# Optimising the performance of frontline implementers engaged in the NTD programme in Nigeria: lessons for strengthening community health systems for universal health coverage

**DOI:** 10.1186/s12960-019-0419-8

**Published:** 2019-11-01

**Authors:** Akinola Oluwole, Laura Dean, Luret Lar, Kabiru Salami, Okefu Okoko, Sunday Isiyaku, Ruth Dixon, Elizabeth Elhassan, Elena Schmidt, Rachael Thomson, Sally Theobald, Kim Ozano

**Affiliations:** 1 Sightsavers, Nigeria Country Office, 1 Golf Course road, PO Box 503, Kaduna, Kaduna State Nigeria; 2 Liverpool School of Tropical Medicine, Liverpool, United Kingdom; 3 Sightsavers, Nigeria Country Office, University of Jos, Jos, Nigeria; 4 University of Ibadan, Ibadan, Nigeria; 5 Federal Ministry of Health, Abuja, Nigeria; 6 Sightsavers, UK, Haywards Health, UK

**Keywords:** Frontline implementers, NTD programme, Optimising performance, Challenges and solutions, Nigeria, Universal health coverage, Health equity, Participatory research methods

## Abstract

**Background:**

The control and elimination of Neglected Tropical Diseases (NTDs) is dependent on mass administration of medicines (MAM) in communities and schools by community drug distributers (CDDs) who are supported and supervised by health facility staff (FLHF) and teachers. Understanding how to motivate, retain and optimise their performance is essential to ensure communities accept medicines. This study aimed to capture and translate knowledge, problems and solutions, identified by implementers, to enhance NTD programme delivery at the community level in Nigeria.

**Methods:**

Qualitative data was collected through participatory stakeholder workshops organised around two themes: (i) identification of problems and (ii) finding solutions. Eighteen problem-focused workshops and 20 solution-focussed workshops were held with FLHF, CDDs and teachers in 12 purposively selected local government areas (LGA) across two states in Nigeria, Ogun and Kaduna States.

**Result:**

The problems and solutions identified by frontline implementers were organised into three broad themes: technical support, social support and incentives. Areas identified for technical support included training, supervision, human resource management and workload, equipment and resources and timing of MAM implementation. Social support needs were for more equitable drug distributor selection processes, effective community sensitisation mechanisms and being associated with the health system. Incentives identified were both non-financial and financial including receiving positive community feedback and recognition and monetary remuneration. The results led to the development of the ‘NTD frontline implementer’s framework’ which was adapted from the Community Health Worker (CHW) Generic Logic Model by Naimoli et al. (Hum Resour Health 12:56, 2014).

**Conclusion:**

Maximising performance of frontline implementers is key to successful attainment of NTD goals and other health interventions. As NTDs are viewed as a ‘litmus test’ for universal health coverage, the lessons shared here could cut across programmes aiming to achieve equitable coverage. It is critical to strengthen the collaboration between health systems and communities so that together they can jointly provide the necessary support for frontline implementers to deliver health for all. This research presents additional evidence that involving frontline implementers in the planning and implementation of health interventions through regular feedback before, during and after implementation has the potential to strengthen health outcomes.

## Background

Neglected Tropical Diseases (NTDs) are responsible for approximately 150 000 deaths a year [1] and cause life-altering morbidity and long-term disability. They limit economic productivity [2, 3] and lead to stigma, discrimination and social exclusion [4–6]. Nigeria has the largest burden of NTDs in sub-Saharan Africa, accounting for 25% of the total burden in the region [7]. As NTDs affect the most disadvantaged and hard to reach populations, often without access to quality health services, they are considered a ‘litmus test’ for UHC and an equity ‘tracer’ [8–10]. One of the main strategies for achieving NTD elimination is the use of safe, single-dose preventative chemotherapy (PC) through mass administration of medicines (MAM), specifically for lymphatic filariasis (LF), onchocerciasis, soil-transmitted helminths (STHs) and schistosomiasis.

In Nigeria, the MAM engages multiple frontline implementers including Frontline Health Facility staff (FLHF), volunteer Community Drug Distributors (CDDs) and teachers. Of all the three categories of people that constitute the frontline implementers, only the frontline health facility staff (FLHF) are trained health workers who have had certificated formal training. Formal training rarely includes a focus on NTDs. CDDs are people selected by the community to assist in medicine distribution who mostly may not have had any formal education. The FLHF are based in local health facilities. They receive and store the medicines and supervise distribution through CDDs and teachers. CDDs distribute medicines (ivermectin and albendazole) for the control of onchocerciasis and LF within communities, while teachers distribute deworming medicines (praziquantel and albendazole) for control of schistosomiasis and STHs within schools. Frontline implementers are an essential link between health services and the endemic communities; they play a role, not only in medicine administration, but also in community engagement which contributes to ensuring equitable access to and acceptability of medicines [11]. Consequently, the recruitment, performance and retention of these frontline implementers are key to the success, sustainability and quality of the NTD programme and its ability to meet control and elimination goals [12].

The performance of frontline implementers within NTD programmes is shaped by the need for a solid knowledge and skills base in relation to NTDs and MAM, confidence to educate communities and the motivation to achieve equitable programme coverage [13–15]. One of the key research questions within NTD programme implementation is how to sustain motivation and improve the performance of frontline implementers which is often influenced by logistics and support from the health system, adequacy of social sensitisation and mobilisation strategies, human resource capacity, financial and non-financial support, inefficient or weak health systems and more [16]. In 2008, a study in south-eastern Nigeria with over 100 CDDs found that attrition increased when there were inadequate supplies of medicines, a lack of supervision and a lack of monetary incentives [12]. Other factors affecting motivation and retention included community selection processes, involvement in other health programmes, incentives, demands of other employment, distances involved in the house-to-house distribution and marital duties. The level of support and acceptance of medicines from community members is also reported as a motivational factor for CDDs in Oyo State, Nigeria [17].

When these frontline implementers leave the NTD programme, there are resulting delays with implementation and increased expenditures [12]. However, while it is acknowledged that frontline implementers are the back bone of NTD programmes, their voices are often missing or ignored in programme planning, reviews and research agendas [16]. One of the key operational questions facing many NTD programmes is how to motivate and retain frontline implementers and optimise their performance?

NTD programme implementation in Nigeria is organised along the 3 tiers of government (federal, state and local government area (LGA)), with the units of operation being the health facilities within LGAs. The central unit head is the national coordinator who is the programme manager at the federal level, while a state coordinator and LGA coordinator manage the project at the state and LGA levels, respectively. Interestingly, funding for implementation of the programme is provided by non-governmental organisations who are partners with the NTD unit of the Federal Ministry of Health, Nigeria, but work in an endemic state of their choice. Hence, different NGO partners work in different states in the country, for example, the NGO supporting NTD programme implementation in Kaduna is different from the NGO supporting implementation in Ogun State. As a result, the level of progress made by each state in the implementation programme is a reflection of several factors which include the NGO supporting them, the funding provided by the NGO and the political will of the state government. Although NTD programme implementation structure is the same across the states, implementation approach (for example length of training for frontline implementers and the amount given as incentives) varies per state and is primarily influenced by the budget provided by the NGO partners. Across both states, MAM takes place annually through a community-based delivery platform for onchocerciasis and lymphatic filariasis and through a school-based delivery platform for soil transmitted helminths and schistosomiasis [18].

The need for an investigation into NTD programme service delivery was based on the fact that the programme was not able to meet its yearly therapeutic coverage targets such as the percentage of people that need to be treated among the at-risk population. Preliminary information from key stakeholders indicated that the way frontline health implementers are supported and managed within the NTD programme could be a factor currently limiting the attainment of coverage goals. Nigeria used the WHO guideline for NTD programme implementation to develop the National NTD master plan and other guidelines. The National NTD master plan gives an overview of the structure of the programme, the diseases endemic in each state and across LGAs and the population affected (supplement 1). There are also standard operational procedures (SOP) for some implementation processes like supply chain management and NTD implementation (supplement 2) and SOP for NTDs (Supplement 3). However, most of these documents are either not in the public domain or only accessible to top programme managers. Most of the frontline implementers are not aware of these documents and so do not access them to guide their role. Furthermore, most of these documents do not contain information that details the expectations or roles of frontline implementers with respect to NTD programme delivery.

This study was designed to understand how to optimise the performance and working conditions of these frontline implementers by capturing and translating their knowledge of the problems and the potential solutions that could be supported by health systems and communities. The study was conducted in Ogun and Kaduna States, Nigeria.

### Theoretical framing

The theoretical framing of this project was adapted from the Community Health Worker (CHW) Generic Logic Model developed by Naimoli et al. [19]. The model theorises that:

*… optimal CHW performance is a function of high quality CHW programming, which is reinforced, sustained, and brought to scale by robust, high-performing health and community systems, both of which mobilize inputs and put in place processes needed to fully achieve performance objectives* [19, p. 1]*.*

**Fig. 1:**
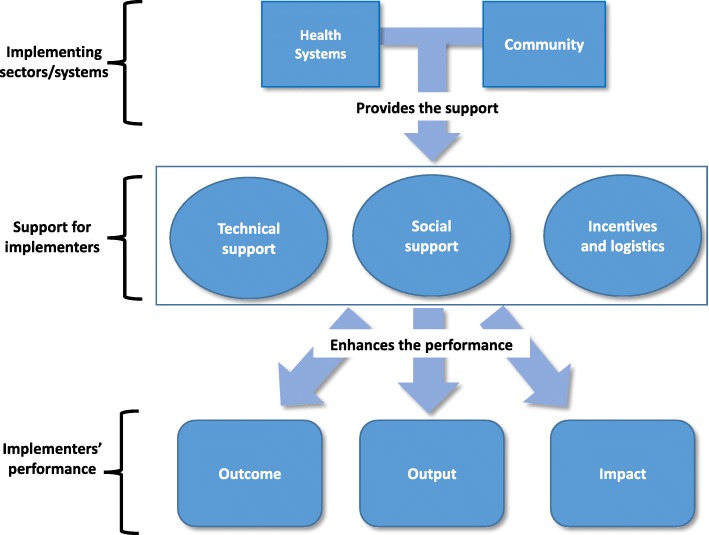
NTD frontline implementer’s framework

We chose to adapt this model and develop the ‘NTD frontline implementer’s framework’ (Fig. 1) as it considers various contextual factors influencing the performance of close-to-community providers such as the frontline implementers in the Nigeria NTD programme: CDDs, teachers and FLHF.

We explore how ‘implementing sectors/systems’, which in this case are health systems and communities (as an extension and part of the health system), can adapt and be better engaged to support frontline implementers and optimise their performance.

The main focus of this paper is on the problems and solutions identified by FLHF, CDDs and teachers. These are explored within three broad ‘support for implementer’ areas: technical support, social support and incentives and how these support mechanisms could lead to changes in implementers’ performance.

NTD ‘implementer performance’ in the model relates to outputs, outcomes and impact. Within NTD programmes, outputs refer to the individual performances of implementers which can be measured directly or indirectly including the knowledge and skills acquired, self-esteem and confidence, satisfaction and motivation to undertake the work. Outcomes are associated with changes occurring among beneficiaries of the NTD programme because of the interaction with the implementers, such as willingness to accept the medicines, strong understanding of the need for medicines and the need to be available during programme implementation. Impact refers to the long-term effect on the population resulting from the interaction with implementers such as reduction in disease morbidity and disease control and elimination.

## Methods

### Study locations and sampling

Kaduna and Ogun States were selected to allow for contextual variation in NTD programme implementation, donor support, progress in disease control and socio-cultural factors. The four most common PC NTDs (LF, onchocerciasis, schistosomiasis and STHs) are endemic in states. Kaduna State is in the northern part of Nigeria and has a long history of support from international non-government organisations (iNGOs). As such, the NTD programme in Kaduna is better resourced and supported technically and has made significant progress toward the control and elimination of NTDs. Ogun State is in the southern part of Nigeria. Here the NTD programme has had inconsistent partner support and is only at the beginning of the implementation of an integrated NTD programme. In total, 12 local government areas (LGAs) (six in both Kaduna and Ogun) were included in the study; the LGAs were purposively selected to ensure epidemiological, programmatic, cultural and geographic diversity. Data collection sites are summarised in Table 1.

**Table 1:** Study locations and reasons for selection

States	LGAs selected	Reasons for selection
Kaduna State	Igabi	• Located in the northern part of Kaduna • It is mainly urban. • Therapeutic coverage for onchocerciasis and lymphatic is higher than the state average • Sociocultural tribe—mainly Hausa • Therapeutic coverage for schistosomiasis is much lower than state average • Therapeutic coverage for STHs is higher than state average
	Ikara	• Located in the northern part of Kaduna. • It is mainly rural • Sociocultural tribe—Hausa Fulani • Therapeutic coverage for onchocerciasis and lymphatic is higher than the state average • Therapeutic coverage for STHs is higher than the state average • Therapeutic coverage for schistosomiasis is much lower than state average
	Kachia	• Located in the southern part of Kaduna. • It is mainly urban. • Has other socio-cultural groups. • Therapeutic coverage for onchocerciasis and lymphatic is higher than the state average • Therapeutic coverage for schistosomiasis and STHs is much lower than state average • Sociocultural tribe—Adara, Gbagi and Ham
	Jema’a (solution finding only)	• Located in Southern Kaduna • It is an urban. • Shares a border with two other states. • The therapeutic coverage for onchocerciasis and lymphatic filariasis is generally higher than the state average. • Therapeutic coverage for schistosomiasis and STHs is much lower than state average. • Sociocultural tribe—Fantswam and Hausa Fulani
	Kagarko (solution finding only)	• Located in Eastern Kaduna • It is largely rural. • Shares a border with two other states. • The therapeutic coverage for onchocerciasis and lymphatic filariasis slightly higher than the state average. • Therapeutic coverage for schistosomiasis is high due to the treatment of adult populations and internally displaced people. • Treatment for STHs did not take place during the 2016 annual treatment round Sociocultural tribe—Koro and Gbagi
	Kabau (solution finding only)	• Located in Northern Kaduna • It is largely urban. • The therapeutic coverage for onchocerciasis and lymphatic filariasis higher than the state average. • Therapeutic coverage for schistosomiasis and STHs lower than state averages. • Sociocultural tribe—Hausa and Fulani
Ogun State	Yewa North	• Located in western Ogun. • It is a rural LGA • Shares a border with Republic of Benin. • An ethnic socio-cultural tribe (Yewa tribe). • The therapeutic coverage for onchocerciasis and lymphatic filariasis is generally higher than the state average. • Therapeutic coverage for schistosomiasis and STHs lower than state averages.
	Ijebu East	• Located in eastern Ogun, mainly rural. • Shares a border with two states. • Home to the Ijebu tribe. • The therapeutic coverage for onchocerciasis is higher than the state average. • Lower for lymphatic filariasis, schistosomiasis and STHs.
	Abeokuta North	• Located in the central Ogun, a mix of rural and urban LGA. • Mainly Egba tribes with some migrant population (Fulani herd’s men). • Therapeutic coverage for onchocerciasis, lymphatic filariasis and schistosomiasis is generally higher than the state average. • STHs have a lower coverage than state average.
	Remo North (solution finding only)	• Located in the eastern part of the state, rural. • Borders another state. • Home of the Remo tribe. • The therapeutic coverage for onchocerciasis is higher than the state average. • Lower for lymphatic filariasis, schistosomiasis and STHs.
	Imeko Afon (solution finding only)	• Located in the western part of the state. • Borders the Republic of Benin. • It is a rural area. • Home of the Yewa tribe. • The therapeutic coverage for onchocerciasis, lymphatic filariasis, schistosomiasis and STHs is lower than state average.
	Ijebu Ode (solution finding only)	• Located in the eastern part of the state. • It is an urban area. • Home of the Ijebu tribe. • The therapeutic coverage for lymphatic filariasis, schistosomiasis and STHs is lower than the state average.

### Data collection

Data was collected through participatory stakeholder workshops organised around two themes: (i) identification of challenges that impacted the ability of frontline implementers to do their job well and (ii) finding solutions to help address these challenges. Three workshops (one each with teachers, CDDs and FLHF) were held in three LGAs per state. There were on average 15 participants per workshop, who were divided into sub-groups and asked to reflect on their current experiences, programmatic challenges and suggested improvements. Small groups fed back to the plenary to allow for broader discussions.

In addition, two extra solution-focused workshops were held with CDDs and teachers separately (each cadre was split into smaller sub-groups based on gender or year(s) of experience with MAM implementation) to further explore in-depth solutions, from three additional LGAs per state with eight to ten participants per workshop. The findings from the first round of workshops informed the development of potential motivational factors and solutions which enhanced the trustworthiness of the study findings by allowing for a process of ‘member checking’ [20]. Participants then worked as co-researchers to further develop the list of factors that would increase their ability to perform well in their roles, specifically related to training, supervision, motivation and incentives. The participatory nature of the workshops allowed implementers to share ideas, compare experiences and comment on the implementation of MAM [21]. Each workshop lasted approximately 3 h. The solution identification workshop stages are shown in Fig. 1. During problem identification workshops with FLHF, participants were also able to generate tangible solutions for change. Consequently, to avoid overburdening this cadre, who are very busy at the health facility, we chose not to conduct solution-focused workshops with FLHF, but rather to draw on the data collected from the first workshop.

### Data analysis

All workshops were recorded and transcribed verbatim. Notes were also taken for quality control and triangulation. Data was analysed thematically using a coding framework developed by the research team. The coding framework was independently applied to the data by five members of the research team and cross-checked between them for consistency. Once all data had been coded, similarities and differences within each code were reviewed to develop thematic charts which were then organised into themes and subthemes [22].

## Results

### Technical support for frontline implementers

As described within the logic model by Naimoli et al. [19], ‘technical support’ includes efforts by health sector actors to ensure sound programme implementation and management. In this study, we found this included (1) training, (2) supervision, (3) human resources and workload and (4) timing of MAM.

### Training

The training of frontline implementers in the studied states followed a cascade structure, where the State NTD team trained local area coordinators, who in turn trained FLHF, who then trained and supervised CDDs. For school-based deworming, the State NTD team and education authorities trained selected teachers, who were then expected to train other teachers in their schools.

This system of cascade training was described as problematic and ineffective. The information about the upcoming training was communicated late, the duration was often too short and many teachers and CDDs received very limited or no training. Some felt that their training was too basic, mainly covering how to calculate the dosage of medicines. Many wanted a more comprehensive understanding of NTDs, the symptoms and manifestations and how to sensitise the community.

We were only informed on dosage and how to measure on the dose pole, we need a structured and effective training. (Participatory meeting with CDDs, Ijebu Ode, Ogun State)

CDDs in Kaduna were unsure how to treat people with physical disabilities and other complex conditions. The messages during the training were inconsistent, and there was no clear treatment guidance. As a result, many CDDs could not determine the treatment dosage, and patients with physical disabilities or complex needs were left out of MAM.

Likewise, FLHF reported minimal training and knowledge on NTD programme implementation and a lack of knowledge on how to handle adverse conditions and stressed that not enough health facility staff are trained to support the NTD programme which could affect their ability to provide adequate training to CDDs.

Teachers reported insufficient numbers of school staff trained to deliver MAM. In Ogun State, only one or two teachers per school were invited, and in Kaduna, only the head teachers were trained. The trained teachers could not always cascade the training, as other teachers thought that the trained teachers had received financial incentives (which was not the case except for travel allowances) and so should be the ones delivering MAM. This significantly increased the workload of the initially trained teachers or resulted in the untrained teachers administering the medicines:

… some teachers did not cooperate with the teachers that went for the training. They said that since two of them went for the training, the two should administer the drug for all the pupils. (Participatory meeting with teachers, Abeokuta North LGA, Ogun State)

Teachers felt unprepared and ill-equipped to manage the challenges related to administering praziquantel. In Kaduna, teachers reported cases of overdose which were attributed to poor training. In Ogun State, there was a reported confrontation between the teachers and the parents of children who had experienced side effects.

Training materials and methods were also criticised by study participants. They wanted to have more interactive and participatory training formats, using role play and practical exercises. Training manuals were not always available in the local language and were difficult to use.

### Supervision

All study participants pointed out that supervision was essential for the effective delivery of MAM. However, many CDDs, particularly in Ogun, reported no supervision during MAM. The only time they communicated with the FLHF was when they reported the data. CDDs in remote communities were particularly concerned about the lack of supervision, as they felt alone and unsupported during MAM. Many CDDs wanted to see their supervisors regularly and argued that this would boost their confidence and increase their communities’ acceptability of MAM:

… if they supervise our work at the state and … LGA level … they will know we are doing it; and the community will know we are not lying … and other community members … will … come forth. (Participatory meeting with CDDs Abeokuta North, Ogun State)

Teachers also reported the lack of support from the health workers, particularly in the management of side effects involving praziquantel. This was a major discouraging factor for the teachers, who wanted health sector representatives to be present in schools during MAM:

Nobody comes from the health sector to supervise. If those from the health sector come and supervise … we will appreciate, and it will mean a lot. (Participatory meetings with teachers, Kauru, Kaduna)

Health workers themselves acknowledged that supervision was often insufficient, largely due to the minimal training received by the FLHF, their poor knowledge of NTDs and insufficient time and other commitments. In Ogun State, for example, only one or two staff per facility were assigned to oversee MAM, while the workload required four or five people:

… after doing all the necessary work in the clinic you will be forced again to do another programme like this onchocerciasis … it is too stressful. (Participatory meeting with FLHF, Abeokuta North LGA, Ogun State)

In addition, some FLHF were unable to regularly visit the communities due to the lack of funds for transportation. CDDs and teachers also complained about limited time to report the MAM data and wanted more days to be able to produce more complete and detailed reports.

### Human resources and workload

All implementers reported insufficient human resources available for MAM. Frontline implementers had to deliver MAM alongside other routine activities, which resulted in high workloads and low morale. In Kaduna, the FLHF reported both insufficient numbers of CDDs and the lack of health personnel to support them:

There is not enough human resources … we don’t have enough CDDs to go around and administer to everyone, we also want more staff as health workers to assist in this Program. (Participatory meeting with FLHF, Ikara LGA, Kaduna State)

Some longstanding CDDs pointed out that their workload had increased significantly, and many of their colleagues had dropped out due to heavy workloads and the lack of incentives to compensate for CDDs’ time. As a result, the remaining CDDs had to cover larger geographic areas and more scattered and isolated communities. CDDs were also asked to deliver medicines more frequently, as the NTD programme had integrated more diseases and expanded. For example, in some communities, CDDs distributed ivermectin and albendazole, and 2 weeks later, they distributed praziquantel. This led to fewer individuals volunteering for the CDD role, while those who continued to volunteer felt demotivated and became progressively disengaged. CDDs in urban settings experienced different types of challenges but also wanted to have more CDDs because many urban dwellings included multiple households or many people living in the same household, as one CDD explained:

CDDs were not enough because you find up to 50 women in a household … you get tired in one household. (Participatory meeting with CDDs, Kaduna North, Kaduna)

### Timing of drug distribution

Teachers reported that MAM sometimes coincided with the exam period, which was busy and stressful. They requested that the NTD programme considered organising MAM in the first 2 weeks of the school term, which was usually a period with less activity.

CDDs requested that either MAM occurs in the dry season or they are provided with bicycles, motorcycles or money to cover the costs of transportation:

Activities are carried out during rainy season and documents are in danger of being ruined, particularly the register … there is a need for them to be given bags, umbrellas and if possible rain coats. (Participatory meeting with CDDs, Kaduna LGA)

### Social support

The findings in this theme were organised into three sub-themes: (1) drug distributor selection, (2) community sensitisation and (3) affiliations with the health system.

### Drug distributor selection

Many study participants argued that the process of selecting CDDs was important to ensure community ownership of and support for MAM. However, in many cases, CDDs were selected by village heads or health facilities due to the lack of time and resources to conduct community meetings. In both states, this selection process resulted in the reduced acceptability of MAM compared with community-selected CDDs, especially in nomadic (Fulani) populations, who did not accept medicines from a non-Fulani CDD:

… more CDDs should be recruited because some Fulani do not accept medicine when non-Fulani CDD goes to them for medication. (Participatory meeting with CDDs, Kauru LGA, Kaduna State)

Some CDDs suggested recruiting more females, particularly in some areas of Kaduna where the cultural norms did not permit men from outside the kinship circle to enter houses.

### Community sensitisation

Community sensitisation was described as a key element of MAM. However, in many cases, the time and resources allocated to community sensitisation were minimal, which impacted on community awareness and acceptability of medicines, particularly in schools:

That awareness … is still very very very low. The awareness [campaign] was very short and … many parents did not allow their children to partake in the deworming exercise. (Participatory meeting with teachers, Imeko Afon LGA, Ogun State)

CDDs and teachers recommended the involvement of a wider range of stakeholders. Teachers recommend involving both school management committees and parent-teacher associations and displaying posters ahead of the deworming campaign. Involving traditional rulers and community leaders and raising awareness via radio, churches and mosques, with education materials being produced in both English and the local languages, were also recommended. Posters were thought to be important, particularly in the nomadic Fulani groups.

### Affiliation with the health system

Both CDDs and teachers wanted to be affiliated with the health system to gain respect in their communities. Study participants suggested having some form of official identification, such as ID cards, branded T-shirts, caps and hijabs with the government logos and information about treatment:

T-shirt will motivate the distribution of medicines because some community members, especially Fulani, only cooperate when they see such identification …. (Participatory meeting with CDD, Kauru LGA, Kaduna)

### Incentives and logistics

Three key sub-themes were identified here: (1) community feedback and recognition, (2) financial remuneration and (3) adequate equipment and resources.

### Community feedback and recognition

Positive feedback from programme beneficiaries was reported to be a major motivating factor; both teachers and CDDs described feeling happy and fulfilled when they received positive feedback from the community.

Study participants also wanted more feedback from the health sector and to be acknowledged as contributors to population health. CDDs wanted appreciation from their supervisors, certificates or preferential treatment at local health centres. Teachers wanted appreciation from the parents, the head teacher and the education authorities:

… thank you or just basic … appreciation would be sufficient … a text message from the higher authority. (Participatory meeting with CDDs, Ijebu Ode, Ogun)

### Financial remuneration

Both CDDs and teachers talked about a need for financial support. For some, this was to cover travel expenses to attend training, collect medicines and access communities. For others, this meant compensation for their time, additional workload and stress related to MAM.

You see, some of us, we want to participate in this thing [MAM], [but] there should be provision for us. Because … we have … work in the school … the work is so much that there should be remuneration to encourage us … and make us happy. (Participatory meeting with teachers, Ijebu East LGA, Ogun State)

CDDs also argued that engaging in MAM without any remuneration was demotivating, particularly when people had to leave their businesses and livelihood activities. Some CDDs preferred to engage with other health programmes, such as the polio campaign where they received better financial remuneration.

### Adequate equipment and resources

All participants talked about the need to improve logistical support for MAM. The key resources mentioned were transportation, bags to carry programme documents and sufficient quantities of registers and measuring sticks. Teachers said that giving medicines to school children required access to clean water, which many schools did not have. They suggested that either the NTD programme should provide clean drinking water at the time of MAM or parents should be instructed to send water with their children to school.

Many frontline implementers reported delays with procurements as well as shortages of medicines. Both FHLF and CDDs reported that the delivery of medicines via the ward focal persons often delayed distribution and requested that drugs be sent directly to the health centres for distribution to the CDDs. Teachers in Kaduna requested that the medicines be delivered at least 1 week before MAM.

CDDs experienced drug shortages because of the incorrect census data. In these cases, CDDs were accused of deliberately missing out individuals or giving out drugs in return for incentives:

It becomes a challenge when the drug finishes … community members start … accusing us that we gave the drug to those who give us more incentives. (Participatory meeting with CDDs Kauru LGA, Kaduna)

A summary of the challenges and solutions proposed by frontline implementers, as well as key variations by context and provider ‘type’, can be viewed in Table 2.

**Table 2:** Summary of challenges and solutions in form of support required for optimal performance of frontline implementers

	Challenges	Solutions
Technical support		
FLHF	• Minimal training and knowledge on NTD programme implementation leave FLHF staff feeling unable to provide supportive supervision to implementers. • Lack knowledge of what to do in case of adverse event in children. • Inadequate human resources result in FLHF feeling overburdened with daily responsibilities at the clinic and limited time to move around for supportive supervision during MAM. • Inadequate number of distributers. • Medicine distribution channel through the focal people results in delay in distribution of medicines. • Medicine arriving during the raining season makes it difficult to reach some endemic communities.	• FLHF staff want knowledge on how to handle children with adverse event during implementation (Kaduna). • FLHF staff request that all health workers be trained on NTD programme implementation rather than selected few (Ogun). • Government should employ more health workers. • More CDDs should be recruited and more health workers to assist in programme implementation. • FLHF staff want NTD medicines to be send to them directly and not through ward focal person to avoid delay in distribution to CDDs (Kaduna). • Medicines should arrive in the state during the dry season when all communities are accessible.
CDDs	• Training time was short and rushed, and in some cases, CDDs were only informed on dosage and how to measure on the dose pole not what is expected in their role. • Training in English hinders understanding of material (Ogun). • CDDs not confident when determining treatment dosage for rare scenarios like people living with physical disabilities that prevent them from standing beside the dose pole. • The CDDs are over-burdened with work load as they had to distribute to large populations and in more than one community (Kaduna). • Time for reporting is too short. • Not enough supervision.	• Duration of training should be 3 h and facilitator should arrive on time. • Use of local language, role play and pictorial training materials during training for better understanding (Kaduna). • Training content to include how to handle side effects and determine medicine dosage for people living with disabilities to increase confidence in medicine administration (Kaduna). • Adequate time should be given for distribution and reporting to allow CDDs to do a thorough job and capture those absent during initial visit (Kaduna). • More CDDs should be recruited based on the population of the community. • Adequate supervision during implementation gives CDDs opportunity to inform supervisors of challenges and get immediate feedback on what to do. • Adequate training and time to fill the recording sheets for reporting.
Teachers	• Inadequate quality of training which is not detailed enough for teachers to understand all they need to know. In Ogun, some teachers were not trained at all. • Some trainers do not have deep knowledge about the diseases and the programme, hence unable to cascade training effectively. • Short notice of training time resulted in many teachers missing the training or arriving late (Ogun). • Training venue not always appropriate for learning. • Training was not interactive or participatory. • Only one or two teachers are trained for medicine distribution. • Lack of supervision will create mistakes. • They have challenge using the reporting tools. • Nobody from the health sector was available to support medicine administration in school. • Reporting time is too tight for them to do a thorough job.	• Have regular training (once/twice every year). • School teachers should be trained rather than head teachers since head teachers can be transferred to other schools anytime (Kaduna). • Use simple training manuals to guide teachers during implementation. Should contain types of food children can eat on the day of administration. • Include training on how to handle side effects to boost confidence. • Training on how to use the record sheet for effective reporting. • More teachers to be trained to distribute medicines, the number should depend on the population of the schools. • Supervision should include access to health personnel from the Ministry of Health and the NGO supporting the programme to ensure staff are doing the right thing. • Supervision should be supportive and not for discipline which will motivate teachers to work faster and easier. • The number of days for reporting should be increased to allow adequate time to submit a detailed report.
Social support		
FLHF	• CDDs imposed on communities by politicians or community leaders are not accepted by the community leading to rejection of medicines. • Acceptability of the programme is poor in some communities including non-indigenous tribes.	• CDDs should be selected from within their community to enhance community acceptability of the medicines. • Traditional rulers to be involved and sensitised about the importance of the programme. • Better sensitisation of tribes by meeting with their leaders or someone that can talk to them in their language, then recruit CDDs from that tribe.
CDDs	• CDDs not from communities they served: rejection of medicines by nomadic Fulani’s. • People refused medicine due to different perceived needs; they are hungry. • Most men in the community (Kaduna) refuse male CDDs access to houses. • People refused the medicine because of side effects. • Time given for medicine administration is short.	• Recruitment of CDDs from communities they serve; recruit nomadic Fulani CDDs to increase uptake of the medicines by the Fulani community (Kaduna). • Sensitisation of community on the importance and benefit of the medicine. • More female CDDs should be recruited to gain access to houses that males CDDs cannot enter. • Traditional rulers to be informed separately by means of a circular so they can use their town criers to spread the news (Ogun).
Teachers	• Some parents do not allow their children to collect the medicine because of socio-cultural beliefs (Kaduna) or they do not trust the source of the medicine (Ogun). • Teachers’ workload increases on the day of medicine administration as they must also teach on those days.	• Sensitisation of parents through Ministry of Education. School-based management committee (SBMC) and PTA should also be involved in sensitisation of parents. • Increase public awareness of the programme using mass media, radio jingles, posters and sensitisation of community leaders, the mosques and churches 2–3 months before implementation of MAM (Kaduna). • Inform and seek the consent of parents for at least 2 weeks before implementation (Ogun). • Post banners in both English and Hausa languages at the school gate a week before implementation to raise awareness among parents. • Teachers assigned to administer medicine to pupils should be free from other responsibilities for that day.
Incentives		
FLHF	• FLHF give their own money to CDDs for transportation, refreshments and materials like exercise books for reporting. • FLHF staff could not travel to supervise CDDs due to lack of transport. • No incentives for CDDs, other health programmes supply incentives. • Lack of incentives discourages CDDs who disengage with the programme when other health programmes offer incentives.	• Adequate transportation allowance or access to a motorbike should be provided for medicine distribution in hard-to-reach areas.
CDD	• CDDs spend their own money to reach some communities, photocopy forms, transportation, purchase stationary and pure water sachets for people. • No incentives from the programme like other health programme demotivate CDDs. • Communities do not provide small incentives as they believe CDDs have been paid by government. • Medicine distribution during raining seasons prevents CCDs from working on their farms. • Lack of medicine result in CDDs being accused of marginalising some community members.	• Adequate transportation allowance, registers and stationary should be provided for CDDs, especially for hard-to-reach areas to prevent out of pocket spending. • Incentives should be provided to encourage implementers, e.g. financial remuneration, provision of uniforms, ID cards, commendation from a higher authority like Ministry of Health, secure employment at the health facility (Ogun) and involvement in other health programmes. • Medicines distribution should be done during dry season (January–March). • CDDs want more medicine to be available to cater for increased population that were not captured during census update (Kaduna).
Teachers	• There is no quality drinking water in schools, so teachers find it difficult to administer medicines to pupils. • Delay in medicine distribution to schools in hard-to-reach areas. Many schools could not get medicine on time; teachers visited the medicine dispensary point several times before they could collect medicine for distribution in their school (Ogun). • Inadequate transportation allowance or delays in receiving payment. • Exam period is a poor time for medicine administration. • Medicine administration disrupts the academic activities as they must suspend lessons to give the medicines. • There are no incentives for teachers’ efforts in medicine administration.	• School authority should make clean water available for pupils taking medicine or pupils could be encouraged to bring clean water from home on the day of medicine administration (Ogun). • Timely provision of the medicines, at least 1 week before implementation day (Kaduna) or on the day of training (Ogun). • Transportation allowance for teachers should be based on distance (Kaduna). • Transport allowance to be paid by cash and not into bank account (Ogun). • Medicine administration should take place within 2 weeks of resumption when academic activities have not commenced fully. • Provide incentives, e.g. financial; commendations from head teachers, parents and Ministry of Education; award of certificate of participation; provision of food on ‘Teachers’ Day’ or during festive period (Christmas and Salah).

NB: The challenges and solutions outlined in the table do not always directly correlate.

## Discussion

FLHF, teachers and CDDs are the critical link in the health system for implementation of the NTD programme in endemic communities. Hence, their motivation for better engagement with communities and prompt addressing of programmatic challenges is key to significantly improve their performance and the success of the NTD programme [13, 16]. In addition, CDDs, teachers and FLHF work across health initiatives and so understanding how they can be better supported within the NTD programme has potential to strengthen health systems and improve UHC.

**Fig. 2:**
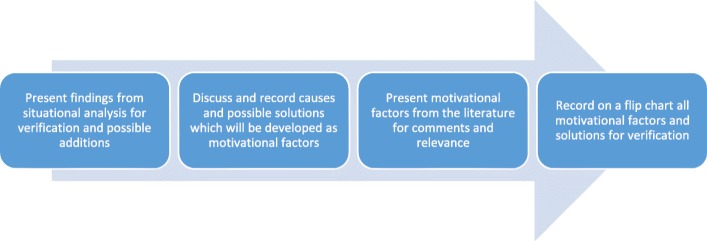
Solution identification workshop stages

Within our discussion, we draw on our adaptation of the Naimoli Framework (Fig. 2) to explore how health systems and communities, as part of the community health system, must collaborate effectively to address the support needs of frontline implementers and put in place processes to fully achieve the objectives of the NTD elimination programme. The ‘implementers framework’ presents a platform to discuss performance criteria that could be improved through understanding and addressing the motivations of FLHF, CDDs and teachers involved in MAM in terms of outputs, outcomes and overall impact. Performance criteria identified from the results of this study are shown in Table 3 and are discussed throughout the discussion. We have indicated in brackets after each heading which performance criteria the section refers to enabling a better understanding of the relationship between the three levels of the model.

**Table 3:** Performance criteria that could be improved through understanding motivations of FLHF staff, CDDs and teachers involved in MAM

Performance criteria	Definition
Output	• Better motivated workforce with job satisfaction • High self-esteem and confidence • Availability and readiness to work • Improvement in competences
Outcome	• Trust established with communities resulting in increased drug acceptance • Communities well informed about the NTD programme and diseases • Reduced number of refusals • Reduced absenteeism • Enhanced management of side effects
Impact	• Increased treatment coverage • Changes in disease status

### Technical support

#### Strengthening capacity of FLHF to implement NTD programme and support CDDs and teachers (output and outcome)

CDDs and teachers need the necessary technical support in the form of training and supervision from the health system to enable them to perform efficiently in their role [23, 24]. As in other studies, our findings emphasised that there are challenges in the quality, duration and content of training that leave gaps in knowledge necessary to successfully and confidently educate communities and manage side effects [25]. Inadequate training leads to poor motivation of frontline providers in NTD programmes and has been reported to negatively impact on adherence of community members to MAM as providers face challenges in convincing community members that they have a good understanding about the medicine they are distributing [24]. Knowledge gaps were exacerbated when treating certain population groups, such as people living with physical disability or other complex treatment cases, consequently affecting equity of programme treatment coverage.

Although National NTD programme guidelines in many countries indicate that health education on NTDs and the treatment programme should be provided to community members by CDDs, only a few received adequate training for this to happen [26, 27]. Adequate and regular training has been shown to be a major motivation for performance and retention of frontline implementers [28–30]. FLHF who are responsible for providing training and supervision for CDDs during programme implementation reveal that they feel ill-equipped to deliver these adequately. This lack of training capacity then cascades down to CDDs, as found in this study, and has a negative impact on training quality, content, style and level of supervisory support.

It is imperative that the health system builds the capacity of the FLHF to deliver on their role by providing them with adequate training on NTD programme implementation. This will enable them to provide adequate training and supportive supervision to CDDs and teachers who are unskilled health workers with direct interaction with the community.

##### Developing or adapting a curriculum and materials to train CHWs

A key output linked to improved performance for NTD programmes is to have a highly motivated, knowledgeable and competent NTD workforce with good self-esteem for MAM. Therefore, it is important that the health system develops a more robust curriculum and training material or guide for FLHF, so staff are able to deliver effective training [31, 32]. FLHF, CDDs and teachers expressed a need for a curriculum that has more interactive, participatory training styles and pictorial training materials to help trainees remember and apply knowledge. Furthermore, teachers want a simple training manual to guide them during implementation, especially as they lack support from the health system. Interactive training curricula that allow providers to engage with the content in practical and similar settings to those they will encounter on the job have also been recommended for CHWs [33]. A lack of sufficient training materials to help FLHF facilitate training and to help CDDs and teachers answer questions and concerns can result in refusal and non-acceptability of the programme by community members, a key outcome within the model [25].

Training duration should be a key consideration in the delivery of such curricula as this was a source of disappointment by CDDs and teachers. WHO recommends that training for CDDs is 1 day long [34]; however, as found here and in other MAM studies, this is often not the case [24]. In Kenya, CDDs mentioned that the training session was brief, hurriedly done and not a full day [23]. A study in compliance with MAM in India by Babu et al. [35] reported that the levels of adherence were higher in areas where the training programme for health workers and drug distributor lasted as long as recommended [35]. Understanding the reasons why training is shortened or inadequate at the different cascade levels is important and should be further researched and addressed. Training length and content could be improved by including specific timings to ensure the training is the recommended duration of a full day, with exercises that clearly indicate how to facilitate an interactive session that also includes role play and opportunities to practise new skills [33, 34].

Consistent evaluation of training programmes as a way to improve and ensure quality and effective training has been recommended in the literature [33]. Other recommendations include regular feedback from frontline implementers that is acted upon to improve the training process and regular monitoring through the use of pre-tests, post-tests and self-assessments [33].

#### Supportive supervision (output)

The need for supportive supervision as a key mechanism to improve the performance of frontline implementers is well reported [12, 31]. The CDDs and teachers here wanted better supervision to enhance their ability to handle side effects, demonstrate to the community that they are supported and thus should be trusted, and discuss challenges and learn through timely feedback on their performance.

Minimal or no supervision and support has been reported in other MAM studies. In a qualitative study in Kenya, nearly half of CDDs reported lack of supervision from health staff as one of the factors responsible for their low motivation [23]. There should also be consideration of how to potentially integrate CDD supervision into the formal health system by better integrating them into the health workforce development and logistics management [19]. This could include a schedule of supervision with adequate tools or checklists that consider individual performance measures and challenges faced by CDDs [19] and could include adapted versions for teacher supervision.

#### Human resources needed for NTD programme implementation (output)

The number of teachers and CDDs needed to implement the NTD programme is high and dependant on two factors: the population of the communities and the distance of one community to another. According to the guideline for implementation of the NTD programme, each community is expected to recommend one CDD or more, depending on the population (ratio 1:100 persons) [36]. In practice, this is not the case as CDDs complained of high workloads and requested for recruitment of more CDDs to support them on the job. In some cases, more than one CDD was selected to serve the community, but this number reduced over time as some CDDs disengaged from the programme. Population to CDD ratio is one of the critical factors found to be significantly associated with high coverage rates as observed in areas where ratio of CDD to population is high [37, 38]. Studies in Nigeria, Yemen, Ghana and Mali have shown that the unrealistically high ratios of populations to be treated to drug distributors [38–41] contribute to decreased motivation and attrition of drug distributors [37]. The challenge of inadequate human resources needs to be addressed if the NTD elimination goal is to be achieved, as potentially many at-risk groups will be left behind, leading to inequalities in accessing NTD medicines. In places with high population, more CDDs should be recruited and more teachers trained to administer drugs during school-based deworming. Recruitment of more CDDs to join the workforce could be a responsibility of the community as this would support in reducing workload. There are also models of community support in supervision structures. This brings different components of Naimoli’s model together (technical and social support) under the responsibility of the community to encourage effective implementation of the NTD programme.

#### Social support (outcome and impact)

Social support provided by communities is achieved when effective sensitisation with community members, leaders and influential community structures, such as religious institutions, takes place [42]. However, all implementers mentioned that poor awareness in communities about the programme and a lack of information about MAM resulted in poor uptake. Studies in Uganda and Tanzania have also found this and demonstrated that compliance with treatment and prevention methods is negatively impacted by poor sensitisation [25, 27, 43]. Research from here and other studies recommends that sensitisation should address negative beliefs, anxieties and local concerns about the medicines and include awareness on the personal risk of NTDs, the need for repeated treatment with the medicine and the reason for using height and weight to identify dosage [25, 27, 42].

Another component of the NTD programme designed to increase community support is for the community to decide when, where and how to distribute medicines through participatory meetings and the selection of CDDs [44, 45]. However, CDDs and FLHF described how a lack of community participation in CDD selection led to a reduction in community ownership of the programme. The critical importance of ensuring the participatory selection of CDDs over preferential selection by community or religious leaders or health system officials has been well documented as critical to programme success and medicine acceptance [27, 46]. Other studies have attributed refusal to the use of a CDD that is not from that community [46, 47]. This is demonstrated as CDDs here stress the importance of recruiting CDDs from Fulani communities who can navigate the cultural beliefs of this group. It is important that CDDs speak the local language, understand the cultures and can build trust [24, 27]. In addition, the gender of the CDD is important for household entry, particularly in northern Kaduna. The use of female and male CDDs will help ensure prompt, equitable and quality health care delivery and also ensure accessibility, equity and trust in health care services [42].

For school-based deworming, teachers reported poor awareness and fear of side effects as the main reasons for parents refusing to allow their children to take part in MAM. This finding is similar to that reported by Musuva et al. [48] in Kenya, where parents mentioned the poor and ineffective sensitisation strategy as a reason for the non-participation of some children, especially the non-enrolled children. Haselow et al. [49] emphasise the need for IEC materials and sensitisation activities that focus on identification and prompt referral to the health sector after side effects. They also mention the need to provide information on the reasons why side effects happen, their frequency and prognosis to stop rumours and allay fears of both the end users and teachers [49]. The teachers in Ogun suggest that consent is taken and awareness raised 2 weeks before MAM so that parents have time to ask questions and understand the need for children to take the medicine.

Teachers’ suggestions of additional methods of sensitising the community include the use of local radio stations, parents’ meetings, meetings with village heads and community chiefs and radio and television announcements, all of which are like those identified in a similar study in Kenya [48].

Understanding how community social support can be created and sustained will facilitate better outcomes and impact within the NTD programme and ensure no one is left behind.

#### Incentives and logistics (outcome and impact)

Incentives are a major motivating factor mentioned by CDDs and teachers. Incentives for CDDs were personal, financial and non-financial [42]. Feedback and recognition from both the community and the health system are personal motivating factors for frontline implementers, coming in the form of awards or certificates of training, to show they are linked with the health system. Feedback and recognition from the community and health system like a letter of recommendation that contributes toward continued professional development, and in some cases to formal integration of the CHW/CDD cadre into the health system, is highly beneficial to improving programme delivery [50].

Reflecting the findings of Njomo et al. [23], implementers mentioned non-financial incentives linked to inadequacy of resources and logistics such as insufficient registers, delay in medicine supply and insufficient medicine as key factors that demotivate implementers. Therefore, it is important that adequate logistics are provided to avoid such problems.

Financial incentives were reasons for insufficient human resources for drug distribution, poor performance and high attrition of distributors. Similar findings have been reported in other parts of the country [12] as well as in other countries like Kenya [42], Mali [38] and Uganda [27], especially because other health intervention programmes, like malaria and HIV, give incentives [51, 52]. As in many other countries, including Nigeria, motivation of CDDs and teachers has increasingly become a challenge, especially for CDDs who temporarily leave their personal occupations to distribute drugs [25].

A number of studies have argued that voluntarism without motivation is not sustainable and therefore recommend the systematic use of multiple incentives based on the different contexts [42]. For example, incentives for a drug distributor in an urban centre may be different from a drug distributor in a rural area. The integration of volunteers into other health and development programmes as a form of motivation and sustainability is also an option that may be explored [53]. However, the question of the capacity of these CHWs to handle the volume of information from different programmes is a concern as they may become inefficient if their task is overwhelming [42]. There is a need to consolidate evidence for decision-makers to help them understand how to provide realistic solutions to the problem of motivating volunteers. Although the study was conducted in six different LGAs in each state, similarities in the perceptions of frontline implementers regarding the challenges and suggested solutions were evident. This supports the generalisability of these study findings to other settings within Nigeria. However, differences which were shaped by the broader socio-cultural context within each state emerged. For example, CDDs in Kaduna clearly requested for the recruitment of additional female CDDs to increase access; however, CDDs in Ogun State did not see this as necessary since they did not have access issues (see Table 2). Additionally, FLHF staff in Kaduna want NTD medicines to be sent to them directly and not through the ward focal person to avoid delay in distribution to CDDs; however, FLHF staff in Ogun do not have this problem as the medicines are sent directly to them and not through ward focal person. Kaduna could learn from the medicine supply chain channel used by Ogun NTD to avoid delay in medicine supply to CDDs.

## Conclusion

Our findings have shown that frontline implementer performance is largely impacted by the support (technical, social, incentives and logistics) provided by health systems and communities during intervention delivery. As NTDs are viewed as a litmus test for universal health coverage, the lessons shared here could cut across other programmes aiming to achieve equitable access to health. It is therefore critical to strengthen the collaboration between health systems and communities so that together they can jointly provide the necessary support for frontline implementers to improve the health of their communities. The provision of supporting factors in a timely and adequate manner is likely to contribute to a better motivated, competent, confident and satisfied health workforce for NTD programme delivery which will strengthen the health system overall. CDDs, teachers and FLHF work across health interventions, and so understanding what optimises their performance is critical to achieving UHC. Outputs linked to the provision of both community and health systems support are then likely to contribute to better programme outcomes including medicine acceptance and disease awareness, which in turn will contribute to the control and elimination of NTDs.

This research highlights a need for consolidated, easily accessible and understandable evidence on frontline implementers’ experiences as well as a concentrated effort to consult health workers, teachers and CDDs in the planning and implementation of health interventions through regular feedback before, during and after interventions.

### Study limitations

A limitation of the study is that the solutions were developed with different groups of individuals to those who identified problems; despite this limiting factor, new challenges were not identified in the solution developments which contribute to supporting the generalisability of findings to the study states. Solutions identified by FLHF could be explored further and are a potential area for future research.

## Supplementary information

**Additional file 1.** Nigeria NTD Multi-Year Master plan (2015-2020)

**Additional file 2.** Standard Operating procedure for Supply chain management for NTD medicine

**Additional file 3.** Standard Operating procedures for Neglected Tropical Disease (NTDs) Elimination in Nigeria

## Data Availability

The datasets generated and/or analysed during the current study are not publicly available due to confidentiality but are available from the corresponding author on reasonable request.

## Abbreviations

CDDs: Community drug distributers
FLHF: Frontline health facility staff
MAM: Mass administration of medicines
NTDs: Neglected tropical diseases
UHC: Universal health coverage
