# Supplementary material for: Optimising the performance of frontline implementers engaged in the NTD programme in Nigeria: lessons for strengthening community health systems for universal health coverage
Source: Hum Resour Health. 2019 Nov 1;17:79. doi: 10.1186/s12960-019-0419-8 (PMC6824027; doi:10.1186/s12960-019-0419-8)
Supplement: Supplementary file 1 — Additional file 1. Nigeria NTD Multi-Year Master plan (2015-2020) [file 12960_2019_419_MOESM1_ESM.pdf]

**Neglected Tropical Diseases**

**Nigeria Multi - Year Master Plan**

**2015 – 2020**

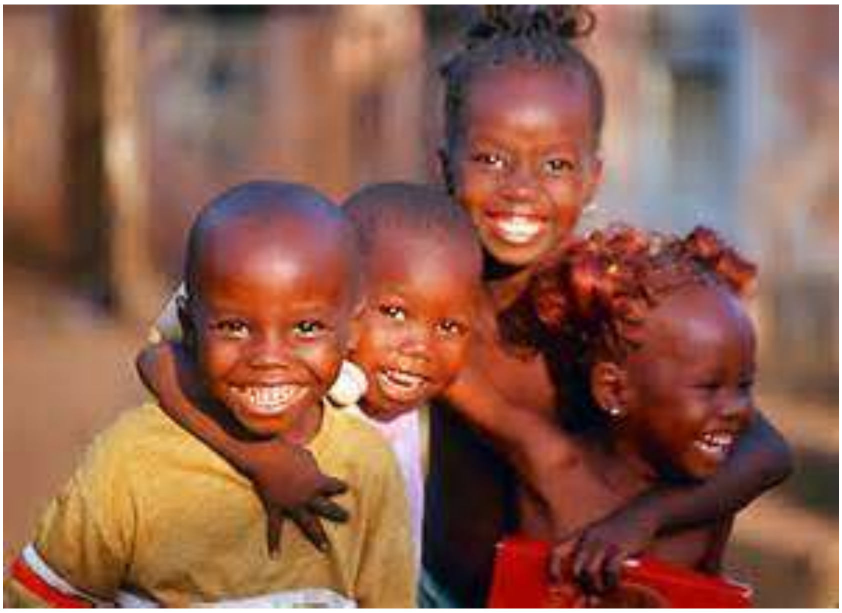

February 2015

## TABLE OF CONTENT


**ACRONYMS/ABBREVIATION** ........ vii

**FOREWORD** ........ xi

**ACKNOWLEDGEMENTS** ........ xii

**INTRODUCTION** ........ 1

**PART ONE: SITUATION ANALYSIS** ........ 5

**1.1 COUNTRY PROFILE** ........ 5

***1.1.1 Administrative structure, demography and community structure*** ........ 5

***1.1.2 Geographical Characteristics*** ........ 8

***1.1.3 Socio-Economic Status and Indicators*** ........ 10

***1.1.4 Transport and communication*** ........ 11

**1.2 HEALTH SYSTEM SITUATION ANALYSIS** ........ 12

***1.2.1 Health System Goals and Priorities*** ........ 12

***1.2.2 Analysis of the Overall Health system*** ........ 12

**1.3 NEGLECTED TROPICAL DISEASES SITUATION ANALYSIS** ........ 18

***1.3.1 Epidemiology and Burden of Disease*** ........ 18

*1.3.1.1* **Onchocerciasis** ........ 18
*1.3.1.2* ***Lymphatic Filariasis*** ........ 19
*1.3.1.3* ***Loasis*** ........ 19
*1.3.1.4* ***Schistosomiasis*** ........ 20
*1.3.1.5* ***Soil Transmitted Helminthiasis*** ........ 21
*1.3.1.6* ***Human African Trypanosomiasis*** ........ 22
*1.3.1.7* ***Dracunculiasis*** ........ 22
*1.3.1.8* ***Trachoma*** ........ 23
*1.3.1.9* ***Leprosy*** ........ 24
*1.3.1.10* ***Buruli Ulcer*** ........ 25
*1.3.1.11* ***Rabies*** ........ 26
*1.3.1.12* ***Leishmaniasis*** ........ 26
*1.3.1.13* ***Yaws*** ........ 26

*1.3.1.14* ***Mycetoma*** ....... 27
*1.3.1.15* ***Dengue*** ....... 27
***1.3.2 NTD Programme Implementation*** ....... *34*
***1.3.2.1 Onchocerciasis:*** ....... 34
***1.3.2.2 Lymphatic filariasis*** ....... 34
***1.3.2.3 Schistosomiasis:*** ....... 35
***1.3.2.4 Soil Transmitted Helminths:*** ....... 35
***1.3.2.5 Trachoma*** ....... 35
***1.3.2.6 Human African Trypanosomiasis:*** ....... 36
***1.3.2.7 Guinea Worm Disease:*** ....... 36
***1.3.2.8 Leprosy*** ....... 36
***1.3.2.9 Buruli ulcer:*** ....... 36
***1.3.2.10 Leishmaniasis:*** ....... 37
***1.3.2.11 Zoonotic and other NTDs:*** ....... 37
***1.3.3 SWOT Analysis*** ....... *40*
***1.3.4 Gaps and Priorities*** ....... *50*
**PART TWO: NTD STRATEGIC AGENDA ....... 52**
***2.1. MISSION AND GOALS:*** ....... *52*
***2.2. NATIONAL MILESTONES*** ....... *60*
**PART THREE: OPERATIONAL FRAMEWORK ....... 67**
**3.1. SCALING UP ACCESS TO NTD INTERVENTIONS AND TREATMENT AND SERVICE DELIVERY CAPACITY ....... 67**
***3.1.1 Scaling up preventive chemotherapy interventions*** ....... *67*
***3.1.2 Scaling up NTD Case management Interventions*** ....... *70*
***3.1.3 Scaling Up NTD Transmission Control Interventions*** ....... *76*
**3.2. PHARMACOVIGILANCE IN NTD CONTROL ACTIVITIES ....... 79**
**3.3. STRENGTHENING CAPACITY AT NATIONAL LEVEL FOR NTD PROGRAMME MANAGEMENT AND IMPLEMENTATION ....... 80**
**3.4. ENHANCING PLANNING FOR RESULTS, RESOURCE MOBILIZATION AND FINANCIAL SUSTAINABILITY ....... 84**
**3.5. STRENGTHNING GOVERNMENT OWNERSHIP, ADVOCACY, COORDINATION AND PARTNERSHIP ....... 86**

3.6. MONITORING AND EVALUATION ........ 90

3.7. POST INTERVENTION SURVEILLANCE AND INTEGRATION WITHIN PRIMARY HEALTH CARE ........ 96

**LIST OF TABLES**

Table 1: National Population Data, Schools and Health Facilities at Distric Level ....... 7

Table 2: Health and Socio-economic Indicators for Nigeria ....... 11

Table 3: NTDs mapping status ....... 34

Table 4.1: Summary of intervention information on existing preventive chemotherapy programmes (as at 2013) ....... 37

Table 5.1: The SWOT Analysis ....... 40

Table 6: Strategic Objectives ....... 53

Table 7: Summary of NTD disease specific goals and objectives ....... 54

Table 8.1: LF Milestones ....... 60

Table 9: Types of mass drug administration ....... 67

Table 10: Activities for strategic priority 1 –Scale up Access to PCT interventions ....... 68

Table 11: Activities for case management interventions ....... 70

Table 12: Intervention packages for Transmission control ....... 77

Table 13: Activities for disease transmission control ....... 78

Table 14: Activities for strengthening pharmaco-vigilance in NTD programmes. ....... 79

Table 15: Activities for Strengthening Capacity at National and Zonal Level and Programme Management. 80

Table 16: Scaling Up Plan ....... 83

Table 17: Enhance planning for results, resource mobilization and financial sustainability of National NTD programmes. ....... 84

Table 18: Strengthening government ownership, advocacy, coordination and partnership ....... 87

Table 19: Strategic Priority 4: Enhance NTD monitoring and evaluation, surveillance and operational research ....... 92

Table 20: Activities for surveillance and sustainability ....... 96

Table 21.1: Summary Budget for federal level activities only ....... 98

**LIST OF FIGURES**


Figure 1: Nigeria Map showing States comprised of LGAs as Implementation Unit ....................................... 5

Figure 2: Geographical Map of Nigeria -Source: Map of Nigeria vegetation. ................................................. 9

Figure 3: Flow of Health Resources ............................................................................................................. 15

Figure 4: Organogram of the Neglected Tropical Diseases Programme ....................................................... 16

Figure 5: Coordination of the Neglected Tropical Diseases Programme ...................................................... 17

Figure 6*: Onchocerciasis Prevalence Map* ................................................................................................. 19

Figure 7: Lymphatic Filariasis endemicity map by LGA in Nigeria ................................................................ 19

Figure 8: Estimated prevalence of eye worm history in Nigeria(Courtesy APOC) ......................................... 20

Figure 9: Schistosomiasis Prevalence Map .................................................................................................. 21

Figure 10: STH Prevalence Map .................................................................................................................. 22

Figure 11: No of Reported Guinea Worm Diseases in Nigeria 1987-2012 ................................................... 23

Figure 12: Trachoma Prevalence Map .......................................................................................................... 24

Figure 13: Chart of New Leprosy Cases in States (2013) ............................................................................. 25

Figure 14: Countries/areas at risk of dengue transmission, 2008 ................................................................. 27

Figure 15: NTDs Co endemicity Map – Nigeria ............................................................................................ 28

Figures 16: PC-NTDs Co-endemicity Maps by State .................................................................................... 28

**ACRONYMS/ABBREVIATION**

| | |
|---|---|
| ACSM | Advocacy Communication for Social Mobilization |
| ADR | Adverse Drug Reaction |
| AFRO | African Regional office of WHO |
| ALB | Albendazole |
| APOC | African Programme for Onchocerciasis Control |
| AU-PATTEC | African Union- Pan African Tsetse and Trypanosomiasis Eradication campaign |
| BU | Buruli Ulcer |
| CATT | Card Agglutination Test for Trypanosomiasis |
| CBM | ChristofelBlinden Mission |
| CDDs | Community Directed Distributors |
| CDI | Community Direct Intervention |
| CDTI | Community Directed Treatment with Ivermectin |
| CHIS | Community Health Insurance Scheme |
| CIA | Central Intelligent Agency |
| CIFF | Children Investment Funds Foundation |
| CL | Cutaneous Leishmaniasis |
| CM | Case Management |
| CSM | Community Self Monitoring |
| CPSS | Community Participatory Surveillance Strategy |
| CWW | Children Without Worms |
| DFB | Damien Foundation of Belgium |
| DFR | Dengue Fever System |
| DRF | Debt Relief Fund |
| DMS | Data Management System |
| DQA | Data Quality Assessment |
| EPI | Expanded Programme of Immunization |
| FCT | Federal Capital Territory |
| FGN | Federal Government of Nigeria |
| FIND | Foundation Innovative New Diagnostic Tests |
| FMC | Federal Medical Centre |
| FMoH | Federal Ministry of Health |
| GAELF | Global Alliance for the Elimination of Lymphatic Filariasis |
| GBUI | Global Buruli Ulcer Initiative |
| GDP | Gross Domestic Product |
| GLRA | German Leprosy Relief Association |
| GSM | Global System for Mobile Communication |
| GWD | Guinea Worm Disease |
| GWE | Guinea Worm Eradication |
| H-ARV | Human antirabies Vaccine |
| HRI | Human Rabies Immunoglobulin |
| HAT | Human African Trypanosomiasis |
| HIV/AIDS | Human Immunodeficiency Virus/Acquired Immune Deficiency Syndrome |
| HKI | Hellen Keller International |
| HMM | Home Management of Malaria |
| HMIS | Health Management Information System |
| HQ | Headquarters |
| HSAM | Health Education, Sensitization, Advocacy & Mobilization |
| HRH | Human Resource for Health |

| | |
|---|---|
| HS | Health Service |
| IAEA | International Atomic Energy Agency |
| ICT | Immuno Chromatographic Test |
| IDM | Innovative (Intensified) Disease Management |
| IDSR | Integrated Disease Surveillance and Response |
| IEC | Information, Education & Communication |
| ILEP | International Federation of Anti-Leprosy Association |
| IST/WA | Inter-country Support Team for West Africa |
| ITN | Insecticide Treated Nets |
| IU | Implementation Unit |
| IVM | Ivermectin |
| J & J | Johnson and Johnson Pharmaceuticals |
| JRF | Joint Reporting Form |
| LEP | Leprosy |
| LEISH | Leishmaniasis |
| LF | Lymphatic Filariasis |
| LFE | Lymphatic Filariasis Elimination |
| LGA | Local Government Area |
| LLIN | Long Lasting Insecticide Treated Net |
| LLTN | Long Lasting Treated Net |
| M&E | Monitoring and Evaluation |
| MAM | Mass administration of Medicine |
| MB | Multibacillary |
| MDA | Mass Drug Administration |
| MDG | Millennium Development Goal |
| MDP | Mectizan Donation Program |
| MDT | Multi-Drug Therapy |
| MITOSATH | Mission to Save the Helpless |
| MIS | Management Information System |
| NAFDAC | National Food and Drug Administration Law and Control |
| NCC | Nigeria Communication Commision |
| NCC-GWDE | National Certification Committee on Guinea Worm Disease Eradication |
| NCD | Non-Communicable Disease |
| NECT | Nifurtimox-Eflornithine Combination Therapy |
| NEGSD | National Economic Growth and Sustainability Development |
| NGDOs | National Non Governmental Developmental Organizations |
| NGOs | Non Governmental Organizations |
| NHA | National Health Account |
| NHIS | National Health Insurance Scheme |
| NIGEP | Nigerian Guinea Worm Eradication Programme |
| NITR | Nigeria Institute of Trypanosomiasis Research |
| NLCP | National Leprosy Control Program me |
| NLFEP | National Lymphatic Filariasis Elimination |
| NLR | Netherlands Leprosy Relief |
| NMEP | National Malaria Elimination Programme |
| NOCP | National Onchocerciasis Control Programme |
| NPC | Nigeria Population Census |
| NPHCDA | National Primary Health Care Development Agency |
| NSHDP | National Strategic Health Development Plan |
| NTBLCP | National Tuberculosis & Leprosy Control Programme |
| NTCP | National Trachoma Control Programme |
| NTD | Neglected Tropical Diseases |

| | |
|---|---|
| ORG | Organization |
| OOPE | Out of Pocket Expenditure |
| PB | Paucibacillary |
| PCD | Partnership for Child Development |
| PCR | Polymerase Chain Reaction |
| PCT | Preventive Chemotherapy |
| PHASE | Preventive Chemotherapy, Health Education, Access to Portable water, Sanitation, Environmental Manipulation |
| PHC | Primary Health Care |
| POD | Prevention of Disability |
| PTC | Prevention and Transmission Control |
| RTI | Research Triangle Initiative |
| SAFE | Surgery, Antibiotics, Facial Cleaness, and Environmental Management |
| SAE | Severe Adverse Event |
| SCH | Schistosomiasis |
| SDPs | Service Delivery Points |
| SMOH | State Ministry of Health |
| SS | Sentinel Site |
| SSI | Sight Savers International |
| STBLCP | State Tuberculosis & Leprosy Control Programme |
| STHs | Soil Transmitted Helminthes |
| TAS | Transmission Assessment Survey |
| TCC | The Carter Center |
| THE | Total Health Expenditure |
| TIPAC | Tool for Integrated Planning and Costing |
| TOT | Trainer of the Trainer |
| TRA | Trachoma |
| UNDP | United Nations Development Program |
| UNICEF | United Nations Children’s Fund |
| VBHW | Village Based Health Workers |
| VDC | Village Development Committee |
| VL | Visceral Leishmaniasis |
| VPF | Vitual Poverty Fund |
| WASH | Water Sanitation Hygeine |
| WATSAN | Water and Sanitation |
| WHA | World Health Assembly |
| WHO | World Health Organization |
| YGC | Yakubu Gowon Center |

## LIST OF CONTRIBUTORS

| | | | |
|---|---|---|---|
| Dr. Bridget Okoeguale | FMOH | Mrs Mercy Agosa | FMOH |
| Dr. U. N. Onyebuchi | FMOH | Mrs. Leticia Nwafor | FMOH |
| Mrs Ifeoma Anagbogu | FMOH | Mr.Afolayon Gabriel | FMOH |
| Dr. Y. A. Saka | FMOH | Mr Agi Maiangwa | FMOH |
| Dr.Obiageli J. Nebe | FMOH | Mr. Faladun Olumide | FMOH |
| Dr. James .I. Balami | FMOH | Dr. Nnamdi Orazulike | FMOH |
| Mr. Chukwu Okoronkwo | FMOH | | |
| Mr. Emmanuel Davies | FMOH | Olaniyi Oluwafisayo | FMOH |
| Mrs.Adenigba Mary | FMOH | Dr. Jibrin B. Suleiman | FMOH |
| Mrs. Rita Urude | FMOH | Dr. Nicholas O. Alor | FMOH |
| Mr. Jacob Solomon | FMOH | Dr. OlusholaOgunmola | FMOH |
| Mrs. Audrey Nyior | FMOH | Dr. Uzoma Nwankwo | FMOH |
| Dr. Nicholas Olobio | FMOH | Mr.Tukur Ali | FMOH |
| Mr.Emeka Uzoma | FMOH | Mr. Shehu Jibrin | FMOH |
| Ms Omolola Olojede | FMOH | Dr.Teyil Wamyil | FMOH |
| Mrs.DorcasMernyi | FMOH | Dr LamineDiawara | WHO/IST/WA |
| Mr.IkennaNwoye | FMOH | Prof TchuemTchuente | WHO/IST/WA |
| Mr. Michael Igbe | FMOH | Dr. Ngozi Njepuome | WHO Consultant |
| Mr. RotimiAdegunju | FMOH | Dr Dorcas Alusala | WHO Consultant |
| Mrs. Ebosele Monica M | FMOH | Dr Sam-Wobo Sammy | WHO Consultant |
| Mr.Adekunle Adeyemo | FMOH | Mr. John Dada | Malaria Consortium |
| Mrs. V. S Augustine | FMOH | Mr. Chris Ogoshi | HANDS |
| Mr.Bitrus Freeman | FMOH | Mr. Benjamin Nwobi | RTI/ENVISION |
| Funmi Areola | FMOH | Mr. Samson Rufus | RTI/ENVISION |
| Mr. Gideon Ntuen | FMOH | Mr. Joseph Kumbur | HKI |
| Mr.Falaja Oladipo | FMOH | Mrs.Jamila Iriogbe | MITOSATH |
| Mr.Ekpenyong Edem | FMOH | Mr.Adamani Williams | Sightsavers |
| Ms Remilekun Adekoya | FMOH | Mrs. Martha Damina | Sightsavers |
| Mr. Ibrahim I. Daguleng | FMOH | Mrs.Safiya Sanda | Sightsavers |

## FOREWORD

Neglected Tropical Diseases (NTDs) are communicable diseases associated with poverty and prevalent in areas that have poor sanitation, inadequate or no safe water sources and substandard housing conditions.

The NTDs are estimated to affect over one billion people in the world, majority of who are living in developing countries. In Nigeria, it is estimated that 122 Million persons are at risk of one or more of these NTDs.

The NTDs include Lymphatic Filariasis (LF), Onchocerciasis, Schistosomiasis, Soil Transmitted Helminths (STH), Human African Trypanosomaisis (HAT), Guinea Worm Disease (GWD), Trachoma, Leishmaniasis, Leprosy, Buruli Ulcer (BU), Dengue Fever,and Rabies among others. These NTDs are endemic in Nigeria with children mostly at risk of Schistosomiasis and Soil Transmitted Helminthes (STH). NTDs have been reported to cause debility in humans, blind or maim, permanently curtailing human potentials and impairing economic growth. They also impair child development and growth, intellectual development and educational outcomes, thereby limiting national productivity.

Nigeria has made tremendous progress in curtailing the effects and transmission of some of these NTDs especially with regards to onchocerciasis, leprosy and GWD. Recently, Nigeria was certified guineaworm-free by WHO. However, the NTD burden in the country is still heavy. The first edition of the Nigeria NTDs Multi-Year Strategic Plan developed in 2012 clearly spelt out mechanisms and processes for an integrated approach to the control, elimination and eradication of NTDs in Nigeria. While major progress in the implementation of the Plan has been recorded in the last two years, a lot still needs to be done. This revised edition is to address the gaps identified in the implementation of the initial plan and has been updated to highlight the road maps and targets enunciating strategies for sustainable integrated interventions for NTDs control and elimination by 2020, in line with the WHA (66.12) and WHO Regional Committee (RC 63) Resolution on NTDs.

The Federal Ministry of Health and Government of Nigeria, in collaboration with various stakeholders and partners, is set to achieve the targets for control, elimination and eradication of the NTDs in an integrated and cost-effective manner. It is envisaged that the use of the revised Plan will facilitate implementation of the NTDs control, elimination and eradication strategies more effectively.

I wish to appreciate the inputs of all stakeholders who contributed in various ways to update the National NTDs Multi-Year Strategic Plan. I enjoin all partners and stakeholders to make concerted effort to achieve the successful implementation of this Plan.

I hereby reaffirm the support and commitment of the Government of Nigeria to ensure that the global targets for NTDs control and elimination are met in Nigeria.

Dr Khaliru Alhassan
Honourable Minister for Health
Federal Republic of Nigeria.
April 2015

## ACKNOWLEDGEMENTS

The Government and people of Nigeria sincerely appreciate the effort, unflinching support and commitment of all partners and stakeholders in the fight against the Neglected Tropical Diseases (NTDs) in Nigeria and the world over. These partners include the financial, logistics and medicines donors including partners such as the World Health Organization that provide technical and other forms of assistance.

The Government of Nigeria appreciates the truly dedicated and committed team in the Federal Ministry of Health NTDs Division who have worked tirelessly to ensure that this strategic plan is updated and fully implemented in order to achieve the control and elimination targets for various NTDs. The efforts of the Consultants who provided needed technical assistance to the NTDs team to produce this document are most commendable and appreciated.

I wish to thank the line Ministries, Departments and Agencies for their inputs so far and their expected contributions in implementing the provisions of this document.

On behalf of the Federal Government of Nigeria, Ministries of Health (MOH), the entire NTDs Teams nationwide and the voiceless rural poor affected by these NTDs, I thank everyone who has contributed to the review and update of the NTDs National Multi-Year Plan of Action, 2015 - 2020.

Dr Bridget Okoeguale

Director, Public Health
Federal Ministry of Health, Nigeria
April 2015

## INTRODUCTION

The Neglected Tropical Diseases (NTDs) Programme Master plans are essential components for effective planning and implementation of sustainable NTD programmes. The Plan provides programme goals and objectives, as well as a six - year detailed strategic plan based on extensive situation analyses and addresses all components of the NTD programmes considered relevant to Nigeria. It enhances synergies among various NTD initiatives such as Guinea Worm Eradication (GWE) and Lymphatic Filariasis Elimination (LFE); provides the basis for integrated or linked NTD project plans and includes costing and financing requirements for effective NTD programme implementation and performance.

A tool developed by the World Health Organization (WHO) and currently driven by Research Triangle Initiatives Institution (RTI/Envision), TIPAC (Tool for Integrated Planning and Costing) is used for costing as the Master Plan provides a platform for integrated planning and costing and for resource mobilization for the NTD programme. It also enhances partner coordination and alignment with national priorities. The NTD Master Plan includes scenarios and strategies for financial sustainability and management that are closely associated with the health sector budgeting and planning cycles in Nigeria. This promotes strong linkages with other non - health sectors and disease control programmes in the country.

The following will be brought into perspective in the implementation of this Master Plan:

- Reflection of lessons learnt and how they affect the new approach
- Strategies are comprehensive, and linked to national priorities, targets and goals to cover all NTDs; preventive chemotherapy and case-management NTDs
- Planning based on national strategic priorities rather than a disease or an initiative is fostered.
- Activities with other health interventions and within the NTD programme to solve shared problems integrated and consolidated
- Costs financing of the NTD Programme in order to ensure financial sustainability, and links the NTD programme to health sector planning and financing mechanisms;
- The Master Plan provides a strong base for the country's annual NTD work plans.
- Implementation of the Plan promotes partnership and collaboration.

The national NTD Master Plan forms the basis for harmonized implementation and performance monitoring of all NTD interventions in the country. The Plan aims to provide all partners and stakeholders working on NTDs in Nigeria with a harmonized tool that will facilitate integration, partnership and collaboration and therefore effectively manage available resources while reducing wastage. The Plan will also facilitate the achievement of the 2020 NTDs elimination targets and goals as defined in WHA resolution 66.12 and the Regional Committee Resolution. It is guided by the following strategic priorities:

- to scale up access to NTD-related interventions
- to enhance planning for results, resource mobilization and financial sustainability of national NTD programmes;
- to strengthen advocacy, coordination and national ownership;
- to enhance monitoring, evaluation, surveillance and research.

Progress in implementation of planned activities as well as the programme performance and output will be monitored regularly and evaluated at appropriate intervals. The strategic plan will be the framework for partner coordination, harmonization and alignment. The content is expected to enhance commitment and accountability, transparency, evidence based and verifiable plans of all stakeholders to enhance effective and sustainable resource mobilization.

Stakeholders will regularly review progress and lessons learnt from previously implemented and ongoing activities, based on which the national Plan may be reviewed and updated as required in order to meet the 2020 goals. Impact assessments where needed may be conducted during the course of events

The preparation and updating of this NTD Master Plan is part of a process that includes analysis, development of the NTD programme plan, approval and scaling up of the national NTD Strategic and Operations Plans. The preparation of the original Master Plan comprised the following steps which included regular reviews and consultations with the National NTDs Steering Committee, partners and stakeholders in country and outside, as well as consideration of outcomes of various monitoring and evaluation activities.

The PHASE activities are emphasized in this revised version of the Plan and adequately described and covered in the Plan. These are as follows:

P - Preventive chemotherapy

H - Health Education

A - Access to clean water

S - Sanitation Improvement

E - Environmental manipulation

This Master plan comprises three main components:

- Situation analysis;
- The NTD strategic agenda and
- The Operational Framework.

The situation analysis cover the NTDs, the health system and the external profile. The NTDs strategic agenda covers the mission, vision, strategic goals, programme objectives and focus, strategic milestones, priorities and objectives. The operational framework covers the NTDs multi year budget for strategic activities and sub activities while addressing the strategic priorities and objectives. The States Plans were pooled and incorporated in the revised national Plan.

## EXECUTIVE SUMMARY

Nigeria is a sub-saharan country located on Latitude 8 E and Longitude 10 N making it a tropical country. It spans a land mass of 923,768sq km that stretches across the northern Sahelian climate through derived Savannah to the Mangrove swamp forest in the southern limit where it is bound by the Atlantic Ocean. It is further bound by three countries namely: Cameroon in the east, Benin in the west and in the north by Niger. The climate varies from arid in the north to tropical in the middle belt while the landscape is lowlands in the south merging into the central hills and plateau mingled with mountains in the southeast and plains in the north.

Nigeria is made up of six geo-political zones comprising 36 States, a Federal Capital Territory (Abuja), and 774 Local Government Areas (LGAs). The country operates a Federal system of government with the Executive, Judiciary and a bicameral Legislative arm (the Senate and House of Representatives). The Federal Government of Nigeria (FGN) is headed by an elected President. Each federating unit (State) has three arms namely: Executive, Judiciary and Legislative. The State Government is headed by an elected Governor. Each LGA is governed by an elected Chairman and a Legislative Council. The Federal structure as outlined in the Nigerian Constitution provides for some level of administrative as well as financial autonomy for each State in the federation.

Nigeria is a country with many ethnic groups that confers on it the diversity of its cultural settings. There are about 350 ethnic groups, the predominant ones being the Hausa in the North, the Yoruba in the South West and the Ibo in the South East. There are three main languages, Hausa, Igbo and Yoruba and most communities have settlement pattern characterized by nucleated villages with satellite hamlets and dispersed settlements with generally large family compounds of many related families. In all the regions, an average community size is approximately 2,500-12,000 persons with approximately 30-50 percent of the population living in hamlet-sized settlements.

The health care delivery system in Nigeria consists of both orthodox and traditional health care delivery systems. Both systems operate side by side but with minimal collaboration. The goal of the national health policy is to bring about a comprehensive health care system based on PHC that is promotive, protective, preventive, restorative and rehabilitative to every citizen of the country within the available resources so that individuals and communities are assured of productivity, social well being and enjoyment of living. The Federal Ministry of Health prioritized the NTDs and included them among the forty (40) communicable and non-communicable diseases and conditions for Integrated Disease Surveillance and Response (IDSR).

Currently, the Federal Ministry of Health are addressing the following NTDs based on outcomes of mapping surveys, case searches or high-suspicion index: **Preventive Chemotherapy NTDs** (Lymphatic Filariasis, Onchocerciasis, Schistosomiaisis, Soil Transmitted Helminths, and Trachoma) and **Case-Management NTDs** (Leprosy, Buruli Ulcer, HAT, GWD, Lymphoedema, Trichiasis, Rabies, Leishmaniasis, Yaws, Dengue and Mycetoma). The PC-NTDs pose a huge disease burden on the population. About 50 million persons are at risk for Onchocerciasis while over 114 million Nigerians are at risk of lymphatic filariasis. 43 million and 35 million persons need treatments for schistosomiasis and for STH respectively. Nearly 20 million persons are at risk for trachoma.

The strategic goal of the NTD Programme is to reduce morbidity, disability and mortality via the control, elimination and eradication of targeted NTDs and contribute to poverty alleviation, increased productivity and better quality of life of the affected people in Nigeria. Its focus is to progressively reduce morbidity, disability and mortality due to NTDs using integrated and cost-effective approaches with the view to eliminating NTDs in Nigeria by the year 2020. The Programme's Strategic Priorities are:

- Strengthen government ownership, advocacy, coordination and partnerships.
- Enhance planning for results, resource mobilization and financial sustainability of national NTD programmes for NTD management, control and elimination..
- Scale-up access to interventions, treatment and system capacity building.
- Enhance NTD monitoring and evaluation, surveillance and operational research

The operational framework component of this NTD master plan describes how Nigeria will in practice implement the planned activities. It explains what the country's capacity needs are, how resources will be mobilized, how potential risks will be addressed, the scale-up strategy, verification and assessment of disease elimination and how the sustainability of the project achievements will be ensured. Mass drug

administration, case management and transmission control are the main strategies that will be used to achieve the stated goals and objectives in the control of NTDs in Nigeria.
The major activities that have been planned include procurement of medicines, conduct of mapping surveys, capacity building of personnel at various levels, conduct of MDA, surveillance, integrated vector management, and advocacy sensitization and resource mobilization campaigns. Others include development of structures in the non-CDI States, development of tools and guidelines for NTD implementation, programme review meetings, supervision, monitoring, impact assessment, establishment of an integrated data management system, as well as the strengthening of NTD Focal Points at Zonal, State and LGA Levels.

The total cost for the implementation of this six-year plan,covering both national and State level activities, is forty-one billion, four hundred and eighty million, eighty-six thousand, six hundred and eighty-nine Naira (N 41,480,086,689) with Government and other partners contributing.

## PART ONE: SITUATION ANALYSIS

### 1.1 COUNTRY PROFILE

Nigeria is a sub-saharan country located on Latitude 8 E and Longitude 10 N making it a tropical country. It spans a land mass of 923,768sq km that stretches across the northern Sahelian climate through derived Savannah to the Mangrove swamp forest in the southern limit where it is bound by the Atlantic Ocean. It is further bound by three countries namely: Cameroon in the east, Benin in the west and in the north by Niger. On its North-eastern extremity is the Lake Chad that is central to Niger, Cameroun and Nigeria.The climate varies from arid in the north to tropical in the middle belt while the landscape is lowlands in the south merging into the central hills and plateau mingled with mountains in the southeast and plains in the north.The population of the country (over 170 million) easily makes it the most populous country in the African continent bringing along with it the challenges of underdevelopment and the attendant large burden of socio-economic challenges and burden of diseases including high maternal and child morbidity and mortality.

#### 1.1.1 Administrative structure, demography and community structure

**Political and Administrative structure of the country**

Nigeria is made up of six geo-political zones comprising 36 States, a Federal Capital Territory (Abuja), and 774 Local Government Areas (LGAs). The country operates a Federal system of government with the Executive, Judiciary and a bicameral Legislative arm (the Senate and House of Representatives). The Federal Government of Nigeria (FGN) is headed by an elected President. Each federating unit (State) has three arms namely: Executive, Judiciary and Legislative. The State Government is headed by an elected Governor. Each LGA is governed by an elected Chairman and a Legislative Council. The Federal structure as outlined in the Nigerian Constitution provides for some level of administrative as well as financial autonomy for each State in the federation. Most of the country's revenue is centrally generated and shared among the three tiers of government on an agreed and Constitutional revenue allocation formula. In addition, States and LGAs are autonomous and each generates independent, internal revenues. Each tier of government prepares its own annual plan and budget. Figure 1 below shows the States of the Federation comprised of varied number of LGAs which are the NTD implementation Units

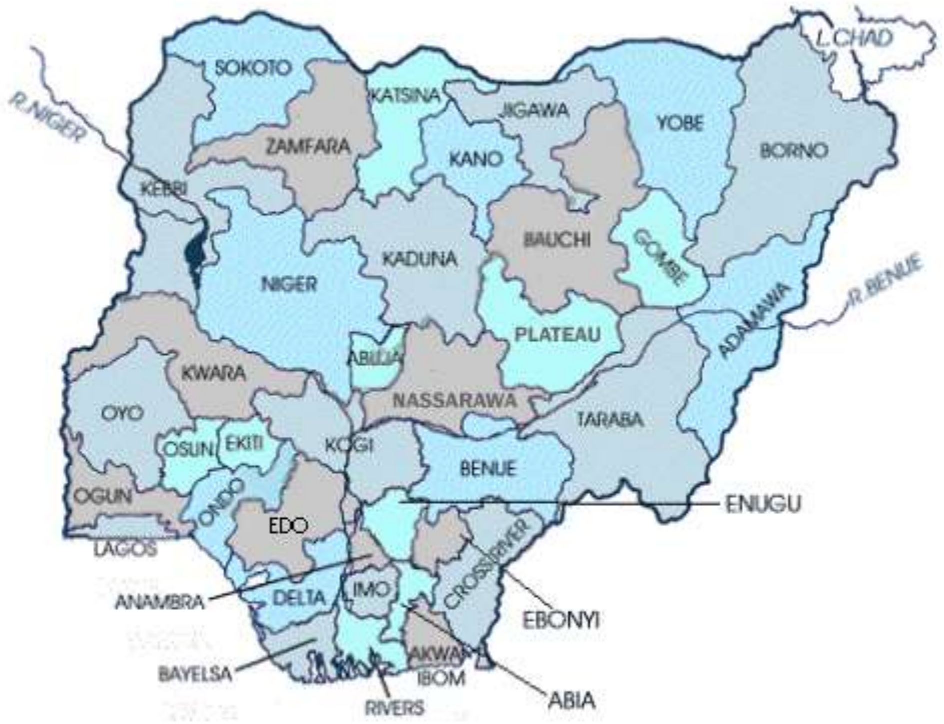


Figure 1: Nigeria Map showing States comprised of LGAs as Implementation Unit

**Census pattern and data**

In 2006 Nigeria's population was 140,431,790 (NPC, 2006) with a projected population of over 170 million in 2014 based on a 2.5% growth rate. The male: female ratio is 1:1. About 55% of the population resides in the rural areas (NDHS 2008). The population distribution is as follows: 0-6months 4%, under 5 years is 20%, under 15 years is 47.6% and 15 years and above is 29.4%. The rural: urban population is at ratio of 45:55. The country has nearly 36,000 registered health facilities.

**Social organisations in communities and settlement patterns**

Nigeria is a country with many ethnic groups that confers on it the diversity of its cultural settings.There are about 350 ethnic groups,the predominant ones being the Hausa in the North, the Yoruba in the South West and the Ibo in the South East. There are three main languages, Hausa, Igbo and Yoruba and most communities have settlement pattern characterized by nucleated villages with satellite hamlets and dispersed settlements with generally large family compounds of many related families.

In all the regions, an average community size is approximately 2,500-12,000 persons with approximately 30-50 percent of the population living in hamlet-sized settlements. Majority of the population of NTD-endemic communities depend on subsistent agriculture, handicraft including mat and basket weaving, pottery, blacksmithing and house building/repairs. However, during the months of reduced agricultural activities, many young men in most communities migrate to the towns and cities for dry season economic endeavors.

The traditional leadership structure in most parts of the north is hierarchical in nature with large areas being under the authority of a District Head who is responsible to the Emir. Under the District Head are Village Heads, and beneath them are Ward Heads who relate directly with heads of households in the communities. In the southern parts of Nigeria on the other hand, villages are run by Councils of respected Elders of the locality. Most communities have Village Development Committees (VDCs) and other types of self-help groups, such as town unions, farmers associations, women societies, traders' associations, vigilante groups and other occupational groups. The community-level NTD programme keys into some of these groups for social mobilization and community monitoring.

Communication channels are many and varied, some being the traditional methods of communication down to the households. Town announcers and musicians are mainly used to communicate information to the people on most issues. The radio is also a very wide spread medium, using local languages. 85% of the populace can be reached using the Radio. Literacy in the local predominant languages is quite widespread, but English is poorly understood in many rural areas of most States especially in the north where enrolment into formal education is low.
Major activities in the communities are centered on key religious and cultural festivals. Major religions in Nigeria are Christianity, Islam and traditional religion. Some communities have festivals during the planting season at the beginning of the rains and/or at harvest time during October-December. Marriages and other festivals generally take place during the dry season, after the harvest. Implementation of NTDs programme activities will be deliberately done to avoid any conflict with the agricultural or festival schedules.

Most communities support development projects such as construction of health facilities and schools, maintenance of community roads, and maintenance and provision of water points. They also have a system for mobilizing the population, especially the youth, to actively participate in these activities. Access to most NTD-endemic parts of the States will be dependent on the use of 4-wheel drive vehicles and motorcycles during both rainy and dry seasons largely due to difficult and unpaved nature of the road networks which is sandy in the north and flood-prone and muddy in the southern parts.

As with other aspects of society, the roles of women are often governed by socio-cultural and ethnic characteristics. In the north, Islamic practices are common including less formal education for the women, early/teenage marriages and confinement to the household with minimal exceptions for social events. It is noteworthy that women play few roles in the leadership structure of these communities. In the south, women traditionally have economically important positions in inter-regional trade and farming as major income-generating sources with concomitant and significantly influential positions in traditional systems and local organizations.

Table 1: National Population Data, Schools and Health Facilities at Distric Level

| **States** | **Number of villages or communities*** | **Total Population** | **Under 5 years old children** | **Children 5–14 years old** | **Number of primary schools** | **Number of health facilities** | | |
|---|---|---|---|---|---|---|---|---|
| | | | | | | **Referal** | **Health centers** | **Total** |
| Abia | 1,284 | 3,539,276 | 707,855 | 990,997 | 958 | 25 | 664 | 689 |
| Adamawa | 9721 | 3,956,524 | 791,305 | 1,107,827 | 1,668 | 32 | 600 | 632 |
| Akwaibom | 2741 | 4,895,803 | 979,161 | 1,370,825 | 1,318 | 20 | 368 | 388 |
| Anambra | 1238 | 5,222,785 | 1,044,557 | 1,462,380 | 1,260 | 12 | 505 | 517 |
| Bauchi | 14722 | 5,840,161 | 1,168,032 | 1,635,245 | 1,908 | 25 | 923 | 948 |
| Bayelsa | 1454 | 2,127,261 | 425,452 | 595,633 | 559 | 13 | 163 | 176 |
| Benue | 2,442 | 5,269,258 | 1,053,852 | 1,475,392 | 2,713 | 25 | 1,217 | 1,242 |
| Borno | 12919 | 5,184,271 | 1,036,854 | 1,451,596 | 1,476 | 55 | 39 | 94 |
| Cross River | 1098 | 3,607,923 | 721,585 | 1,010,218 | 1,043 | 15 | 643 | 658 |
| Delta | 1,455 | 5,118,329 | 1,023,666 | 1,433,132 | 1,509 | 32 | 395 | 427 |
| Ebonyi | 2818 | 2,714,405 | 542,881 | 760,033 | 957 | 3 | 500 | 503 |
| Edo | 1701 | 4,019,256 | 803,851 | 1,125,392 | 1,263 | 441 | 1,767 | 2,208 |
| Ekiti | 422 | 2,976,814 | 595,363 | 833,508 | 878 | 22 | 274 | 296 |
| Enugu | 4292 | 4,067,919 | 813,584 | 1,139,017 | 1,159 | 8 | 456 | 464 |
| Fct | 559 | 1,754,903 | 350,981 | 491,373 | 435 | 23 | 704 | 727 |
| Gombe | 1024 | 2,939,672 | 587,934 | 823,108 | 1,314 | 494 | 473 | 967 |
| Imo | 3194 | 4,914,150 | 982,830 | 1,375,962 | 1,466 | 55 | 852 | 907 |
| Jigawa | 10,619 | 5,430,961 | 1,086,192 | 1,520,669 | 1,683 | 641 | 641 | 1,282 |
| Kaduna | 6242 | 7,576,305 | 1,515,261 | 2,121,365 | 3,409 | 59 | 1,623 | 1,682 |
| Kano | 6565 | 11,718,933 | 2,343,787 | 3,281,301 | 3,450 | 39 | 1,103 | 1,142 |
| Katsina | 10870 | 7,234,136 | 1,446,827 | 2,025,558 | 2,025 | 21 | 1,406 | 1,427 |
| Kebbi | 4736 | 4,044,603 | 808,921 | 1,132,489 | 1,421 | 33 | 708 | 741 |
| Kogi | 2544 | 4,094,381 | 818,876 | 1,146,427 | 2,007 | 61 | 790 | 851 |
| Kwara | 1,375 | 2,961,165 | 592,233 | 829,126 | 1,521 | 21 | 546 | 567 |
| Lagos | 1208 | 11,256,664 | 2,251,333 | 3,151,866 | 2,158 | 29 | 1,689 | 1,718 |
| Nassarawa | 1052 | 2,326,975 | 465,395 | 651,553 | 1,276 | 15 | 708 | 723 |
| Niger | 5,326 | 4,933,320 | 986,664 | 1,381,330 | 2,805 | 20 | 1,769 | 1,789 |
| Ogun | 8176 | 4,655,884 | 931,177 | 1,303,648 | 1,873 | 44 | 475 | 519 |

| | | | | | | | | |
|---|---|---|---|---|---|---|---|---|
| Ondo | 1918 | 4,297,367 | 859,473 | 1,203,263 | 1,650 | 154 | 461 | 615 |
| Osun | 1642 | 4,275,526 | 855,105 | 1,197,147 | 1,686 | 48 | 1,056 | 1,104 |
| Oyo | 8,831 | 6,983,128 | 1,396,626 | 1,955,276 | 2,618 | 35 | 1,527 | 1,562 |
| Plateau | 2,577 | 3,969,776 | 793,955 | 1,111,537 | 1,837 | 16 | 1,366 | 1,382 |
| Rivers | 2455 | 6,475,854 | 1,295,171 | 1,813,239 | 1,080 | 49 | 363 | 412 |
| Sokoto | 6,463 | 4,617,050 | 923,410 | 1,292,774 | 2,118 | 22 | 727 | 749 |
| Taraba | 6543 | 2,848,011 | 569,602 | 797,443 | 1,679 | 18 | 1,004 | 1,022 |
| Yobe | 8451 | 2,899,349 | 579,870 | 811,818 | 950 | 18 | 499 | 517 |
| Zamfara | 3430 | 4,071,101 | 814,220 | 1,139,908 | 1,058 | 1,018 | 3,150 | 4,168 |
| Total | **164,107** | **174,819,199** | **34,963,840** | **48,949,376** | **60,188** | 3,661 | 32,154 | 35,815 |

Source: *National Population Commission (Population Census 2006), †States' NTD Strategic Plans

Close to 3 million children aged 6-14 years had never attended any school. This represents 8.1 per cent of population of children in that age group. An additional one million children (3.2%) of this same age group are dropping out of school. The adult literacy rates for males are higher than those of females (NBS, 2010). The implication of these figures on the elimination of NTDs is that schistosomiasis interventions which target school-age children will not be reached if treatments are conducted only in schools. However, in the northern parts of the country where out of school children attend *islammiya* classes, distribution of medicines will be undertaken using the *islammiya* teachers. Similarly, the number of females that dropped out of school, perhaps for marriage, is higher than males. There is a correlation between education and good health-seeking behavior. An educated mother can take better care of the health-needs of her familys ince she would be the first to know if a child is sick.

### 1.1.2 Geographical Characteristics

Nigeria has a 2014 projected population of over 170million based on a 2.5% population growth. It lies on the West Coast of Africa between latitude $4^0$ and $14^0$ N and longitude $5^0$ and $14^0$ E. It occupies a land mass of approximately 923,768 square kilometers sharing international borders with the Republics of Niger and Chad to the North, Cameroon to the East, Benin to the West and the Atlantic Ocean to the south (See Figure 2). Nigeria is the most populous country in Africa. The Federal Capital Territory is centrally located in Abuja where the national seat of government is domiciled.

There are two main seasons, rainy season from March to October and dry season from November to February. There are wide climatic variations in the different regions of the country. In the coastal areas, the seasons are not sharply defined and diurnal temperatures rarely exceed 32°C but humidity is very high thereby making nights hot and discomforting . Inland, there are two distinct seasons: a wet season from April to October, with generally lower temperatures, and a dry season from November to March, with midday temperatures that surpass 38°C but relatively cool nights, dropping as low as 12° C . The Jos and Mabilla plateaus, however, have a temperate climate.

Average rainfall along the coast varies from about 180cm in the west to about 430cm in certain parts of the east. Inland, it decreases to around 130cm over most of central Nigeria with a mere 50cm in the extreme north. Two principal wind currents affect Nigeria. The northeast wind brings with it the harmattan which is hot and dry and carries a reddish dust from the desert causing high temperatures during the day but cool at nights. The southwest wind brings cloudy and rainy weather. A greater part of the country consists of a low plateau intersected by two main rivers, the Niger and Benue. Along the coast is a belt of mangrove forests and the vegetation gradually thins into the Sahel savannah in the far north corresponding to average rainfall that ranges in the respective regions. Beyond the coast, lowlands follow the valleys of the Niger and Benue; otherwise the land gives way to a broad, hilly, forest belt that gradually rises to the rocky terrain of Jos and Bauchi plateaus. These plateaus are in the region of savannah which stretches to the semi-desert Sahel zone in the extreme north. In the east is the Adamawa plateau, which borders Cameroon and in which is Nigeria’s highest point, Dimlang (Vogel Peak) that is 2,042m above sea level.

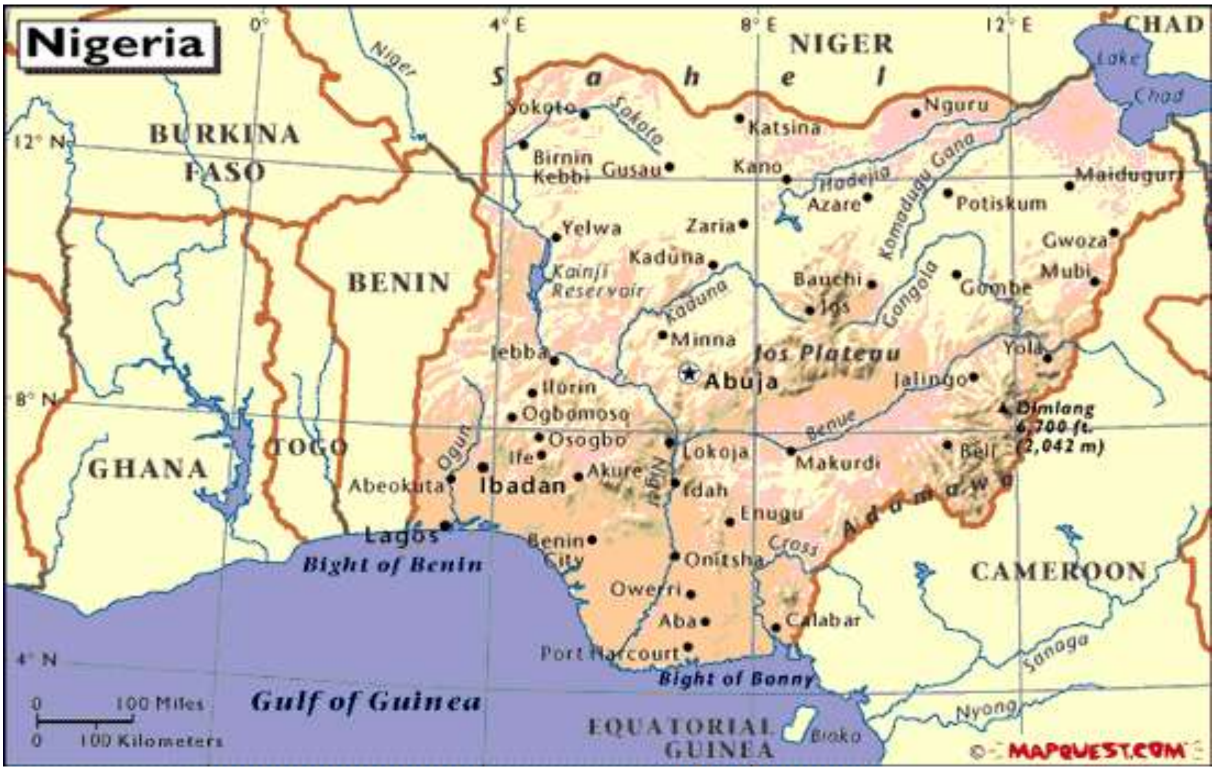


Figure 2: Geographical Map of Nigeria -Source: Map of Nigeria vegetation.

Climate change, global warming caused by the greenhouse effect, and the resulting increase in global temperatures, are possibly causing tropical diseases and vectors to spread to higher altitudes in mountainous regions, and to higher latitudes that were previously spared. In the context of NTDs, this is significant because the physiological activity of both vector and parasite are sensitive to climatic changes (Patz, 2000; McMichael, 2006). For example, dengue and lymphatic filariasis are transmitted by mosquitoes, which require standing water and a warm ambient temperature to breed. Global warming will be accompanied by alterations in the hydrologic cycle (Patz,2000; Zhou X *et al,* 2008), which will impact on rainfall and water availability. Similarly, *Onchocerca volvulus* is transmitted by the black fly, which breeds in fast flowing water bodies. Peak biting density is observed during the wet season (Patz, 2000), indicating a possible increase in the density of transmission of certain NTDs during extreme rainfall. Conversely, there is evidence to suggest that climate change may actually reduce transmission of NTDs in some regions. Heavy rainfall can reduce mosquito populations by flushing larvae from their habitat (McMichael, 2006).

Ecological modifications have been recognized as major threats to the emergence and re-emergence of infectious diseases (Patz, 2000, McMichael, 2004), because they facilitate the breeding of vectors and transmission of parasites. A study by Brou (2008) conducted in Côte d'Ivoire noted that the areas within a few kilometres of hydro-agricultural dams and irrigated rice fields contained the country's highest prevalence of Buruli ulcer. Assuming that confounding factors have little part to play, the continuing development of hydrological systems to advance agriculture may bring about an increase in Buruli ulcer as well as schistosomiasis prevalence, if control measures are not put in place. Schistosomiasis transmission is usually seasonal primarily due to the variation in temperature and irrigation cycle. Transmission can take place in large lakes, rivers, ponds and streams, and these are abundant in Nigeria. Moreover, man-made water bodies (dams, irrigation schemes and canals) abound in Nigeria to service the agricultural sector which increase predisposition to schistosomiasis. Deforestation is carried out for a variety of reasons such as road-building, human re-settlement and farming. Road-building enables non-immune populations, including loggers, construction workers and tourists, to come into contact with indigenous parasites against which they have no immunity, thereby becoming a reservoir for NTDS and facilitating their transmission.

The world population is projected to increase from the present figure of 6.8 billion to beyond 9 billion by 2050. This threatens the control of NTDs because it creates conditions which are conducive to

transmission of infectious disease (United Nations, 2008), as well as increasing the number of people at risk of contracting NTDs. Fertility rates remain highest in poor rural areas of developing countries (United Nations, 2007) which already carry the bulk of the disease burden attributable to NTDs, therefore the impact of population growth will be amplified here. This impact is mediated through a number of factors. For example, developing countries whose health systems are already stretched beyond capacity will be unable to scale up efforts to prevent and treat diseases. Population growth is an important driving force behind migration and outbreaks of civil conflict, which, themselves exacerbate the burden of NTDs (United Nations, 2007).

The urban centres in Nigeria are growing very fast. Abuja, the Federal Capital city, and some of the State capitals have experienced phenomenal growth as a result of migration. Rapid urban growth have resulted in problems of urban congestion or overcrowding, poor housing, poor environmental sanitation, unemployment, crimes and other social vices. Urbanization, secondary to population growth, may increase the transmission of NTDs because of cross-country spread of parasites and the favourable conditions for transmission which ensue in cities. A projected rise in urbanization from 45% in 1995 to 61% by 2030 (McMichael, 2006), threatens to increase the burden of NTDs, in particular because much of this will occur in developing countries like Nigeria where the populations of urban slums will swell disproportionately. Some NTDs like dengue are now emerging/re-emerging as diseases of public health importance in the country and will add to the burden of the existing NTDs. Dense habitation and pools of stagnant water (in car tyres and disused containers) provide additional opportunities for some vectors such as mosquitoes to breed and bite. Similarly, poor hygiene and overcrowding are conducive to the transmission of *Chlamydia trachomatis* (trachoma), a leading cause of blindness throughout the developing world (Wu P-C *et al*, 2009). Urbanization is closely and positively correlated to HIV prevalence (United Nations, 2007), thereby facilitating the spread of NTDs by increasing the immunocompromised population (WHO, 2010). Lastly, urban growth diverts public spending and resources away from rural areas (United Nations, 2007) therefore those suffering from NTDs who remain outside the cities are denied the healthcare they require to manage their condition.

### 1.1.3 Socio-Economic Status and Indicators

The petroleum sector is the main stay of the Nigeria economy being the 6$^{th}$ largest exporter of crude oil in the world, and contributes lower than 10.76% to the annual GDP (NBS, 2014), 75% to government revenue and accounting for virtually all foreign exchange earnings. Recently, the country has begun to exploit the vast deposit of Liquefied Natural Gas. The West African gas pipeline, at an estimated cost of $260m (US), is planned which will supply natural gas from the Excarvos Field to Togo, Benin and Ghana. Other sources of income include agriculture, solid minerals and recently, tourism. Agriculture contributes about 30.9% of the GDP (CIA World Factbook, 2014). The cash crops include cocoa, rubber, coffee, cotton and palm kernels. The food crops include cassava, maize, sorghum, yam, millet, rice and wheat. Nigeria is blessed with arable land, although land cultivation is still mainly in the traditional methods while mechanized is gradually being introduced. Nigeria is also endowed with diverse mineral resources such as tin, coal, gold, lead, columbite, and iron among others. Despite all these Nigeria is ranked the 140$^{th}$ poorest country out of 184 surveyed countries (IMF, 2013) as it is yet to harness all available resources to create wealth and improve the socio-economic status of its citizenry. Over 70% of Nigerians live below the poverty line, particularly women and children in the rural areas, without access to quality and affordable health services and other basic social services. Nigeria is ranked 152 out of 187 countries in the 2014 UNDP Human Development Index. The GDP Per Capita (2013 PPP) is $2,800 (CIA World Factbook, 2014) with an HDI of 0.504 compared with the global HDI of 0.624.

The socio-economic burden of the endemic NTDs in Nigeria will aggravate the current situation if not managed in an integrated manner. This is crucial considering that NTDs, an extremely diverse group of parasitic, viral and bacterial infections are prevalent in conditions of extreme poverty with the majority of sufferers poor and voiceless. Table 2 below shows the socio-economic indices for the country.

Table 2: Health and Socio-economic Indicators for Nigeria

| Health Indicator | National |
|---|---|
| *Total fertility rate (children per woman) | 5.5 |
| GDP per capita (current US$) | 2,800 ( 2013 PPP US$) |
| Life expectancy at birth, total (years) | 54 |
| Use of modern contraception (married women) | 10% |
| *Infant Mortality Rate (per 1,000 live births) | 69 |
| Neonatal Mortality Rate (per 1000 live births) | 37 |
| *Under Five Mortality Rate (per 1,000 live births) | 128 |
| *Maternal Mortality Ratio (per 100,000 live births) | 560 |
| Children fully immunized | 25% |
| Population with access to safe water | 64% |
| % stunted (Under 5) | 36% |
| Population with access to sanitation | 28% |
| Government Expenditure on Health ( 2011) | 6.7%of total budget |
| Health Budget as % of GDP (2007) | 4.78% |
| % of population below poverty line | 70% |
| Adult Literacy rate (Both sexes) (% Aged 15 and above) | 51% |
| Access to potable water | 58.9% |
| Prevalence of HIV, total (% of population ages 15-49) | 4.6% |
| Female Literacy Rate ( 2007) | 58% |

Sources: IMMCH Strategic Document 2007, *NDHS 2013, World Development Indicators, MDG Report 2010, UNGASS Country Report 2008,, World Health statistics 2014

### 1.1.4 Transport and communication

The main transport system in Nigeria is road transportation. Others include air transportation where there are airports (mainly in the State capitals), sea transportation in the riverine areas and less commonly, rail. There are several kilometers of paved roads in Nigeria. The broken rail system has undergone substantial rehabilitation with improved services in some parts of the country. River Niger, the major river that traverses the Northwest, Central part of the country to the Southern parts,is presently being dredged to improve transportation by water ways. NTD-endemic areas are widely distributed across the entire country and some of these areas are hard-to-reach because of the peculiar topography such as difficult terrains and creeks. Implementation of NTD activities is at the community level many of which are in the rural, hard-to-reach areas with narrow, unpaved roads and swamps sometimes. In some remote communities only foot paths exist and can only be accessed by motorcycles or bicycles. In the riverine areas, canoes and boats are used to travel to the communities. At present, most of the project vehicles are worn out and are in desperate need of replacement or simply not available for project implementation leading to added expense and tedious task of hiring vehicles for project implementation in order to ensure that communities do not go without regular treatment.

The introduction of Global System for Mobile communication (GSM) utilizing different service providers such as MTN, Glo, Etisalat, Airtel, Starcomms, Visafone, Multilinks, Zoom, etc has greatly improved communication in Nigeria in most communities. Also, mobile pay phones are widely available in the urban areas. However, there are challenges with their services occasioned by weak and inconsistent signals and reception especially in some rural areas. This has complemented the long-standing analogue telephone system that has been in the country in the last fifty years. The number of GSM subscribers in 2012 is 69.01 per 100 people, and internet users 28.4 per 100 people. The country is yet to achieve full network coverages for mobile telephone with an overall teledensity of 97.6 per 100 people in 2014.There are many public and private Television and Radio Stations and these can be accessed across the nation, especially the radio services. There are government-owned radio and television stations in all the States and the Federal Capital Territory. The accessibility of TV services is largely dependent on the supply of energy in the power sector which falls short of the overall national demand thereby causing periodic power outages and interruption of services. For some remote communities, TV and radio network coverages are minimal.

Postal services are always available across the country with improving service delivery while Courier services continue to be provided by both the public and private sectors. The existing transport and communication systems, in the few areas where they are readily available, are moving in the right

direction. With adequate planning and availability of funds, they can be relied upon for NTD programme implementation including disease surveillance. The NTD offices at all level need to be equipped with communication facilities.

## 1.2 HEALTH SYSTEM SITUATION ANALYSIS

### 1.2.1 Health System Goals and Priorities

The goal of the National health policy is to bring about a comprehensive health care system based on PHC that is promotive, protective, preventive, restorative and rehabilitative to every citizen of the country within the available resources so that individuals and communities are assured of productivity, social well being and enjoyment of living.

The Federal Ministry of Health prioritized the NTDs and included them among the forty (40) communicable and non-communicable diseases and conditions for Integrated Disease Surveillance and Response (IDSR). These diseases were selected on the basis of one or more of the following:

- Causes of high morbidity and mortality in the country (for example, malaria, pneumonia, tuberculosis, and HIV/AIDS, SARI);
- Have epidemic potential (for example, CSM, measles, VHF and Cholera);
- Surveillance required internationally (for example, plague, yellow fever, cholera, SARS, human influenza caused by a new subtype);
- Have available effective control and prevention interventions for addressing the public health problem they pose (e.g., Onchocerciasis, Schistosomiasis. Trypanosomiasis);
- Can easily be identified using simple case definitions; (e.g., Dracunculiasis)
- Diseases targeted for elimination and eradication (e.g., leprosy, polio and guinea worm)

**Top Ten Killer Disease Conditions**
The WHO Statistical Information System (WHO SIS, 2012) identified the following disease as the main causes of morbidity and mortality in Nigeria:
HIV/AIDS, Lower Respiratory Disease (Pneumonia, bronchitis), Malaria, Diabetes, Measles, Peri-natal Conditions, Tuberculosis, Cerebrovascular Diseases (including Stroke), Coronary Heart diseases, Lung Caners.

The process of the development of the initial version of the NTD Master Plan followed after the adoption of the National Policy on NTD by the National Council of Health which is the highest policy making organ for health in Nigeria. The preparation and subsequent updating of the Master Plan have been a collaborative effort of stakeholders including the NTD Programme Officers of the three tiers of government, Development Partners, NGDOs and the NTD Steering Committee. It is envisaged that this document will provide the template for adaptation and implementation at the lower levels with effective coordination by the National NTD office.

### 1.2.2 Analysis of the Overall Health system

The health care delivery system in Nigeria consists of both orthodox and traditional health care delivery systems. Both systems operate side by side but with minimal collaboration. Orthodox health care services are provided by both the private and public sectors. The public health service is organized into primary, secondary and tertiary levels. While the Constitution is silent on the roles of the different levels of government in health services provision, the National Health Policy and just recently, the Health Bill assign responsibilities for primary health care to local governments, secondary care to States and tertiary care to the federal level. The NPHCDA, a federal parastatal, is currently engaged in primary health care services development and provision as part of its mandate. It is responsible for coordination and leadership, while the LGAs are responsible for implementation of the health programmes.

In 2007, the FMOH estimated a total of 17,068 health facilities in Nigeria (Private 7,373, Religious 330, Community 249, Federal 151, State 1,385 and LGAs 7580). Of this total 85.8% are primary health care facilities, 14% secondary and 0.2% tertiary. The private sector owns 38% of these facilities and they provide 60% of health care in the country. Recent data indicates an increase in the number of health facilities to over 35,000 but the breakdown of this number was not seen.The federal-owned tertiary facilities provide specialist services which are mostly not available at the secondary and primary levels, with the teaching hospitals also providing training for health workers and research.

However, the health system in Nigeria is in a deplorable state with an overall health system performance ranking of 187th out of 191 member States by the World Health Organization (WHO). Primary Health Care (PHC) is the bedrock of the national health system but it is in a prostrate state. It will require political will, funding capacity, skilled workforce and essential drugs to deliver quality service.

***i. Service Delivery:***

The Nigerian health service delivery system is based on primary health care and includes among other things:

- Education concerning prevailing health problems and the methods of preventing and controlling them
- Promotion of food supply and proper nutrition
- Maternal and child care, including family planning
- Immunization against the major infectious diseases
- Prevention and control of locally endemic and epidemic diseases
- Provision of essential drugs and supplies.

There is a three-tier system of health care namely: Primary Health Care, Secondary Health Care, and Tertiary Health Care.

***ii. Health Work Force:***

Although Nigeria has one of the largest of human resources for health in Africa they are still inadequate to meet the country's needs both in number, motivation, skills, and distribution. The main challenge for Human Resource for Health (HRH) in Nigeria is the distribution in terms of quantity and mix of health care workers with a skewed distribution towards urban and southern populations. To address the imbalance in HRH provisioning and development a national HRH policy and strategic plan was developed. Specific focus is on building institutional capacities for HRH planning in order to cover issues of optimal distribution of the right quantity and mix of health care workers, performance management, continuing professional development, task shifting and an effective HR information system. Attention is also given to producing more community-focused health care workers and empowering communities to participate in health care delivery for sustainability.

There are 30,391 medical doctors and dentists, 7,581 Pharmacists as at 2011 and 2010 respectively, 128,918 registered Nurses, 90,489 registered midwives, 4,308 public health Nurses and 1,794 peri-operative Nurses as at 2007 as well as 40,491 registered Community Health Officers as at 2009. These numbers translate to 1 doctor to 6,143 persons and 1 Nurse per 1,356 people, but they may not reflect the true current data on the health workforce strength due to factors such as brain drain (Nigeria Annual abstract of statistics, 2012).

With a large population of CDDs (over 120,000) already trained many communities in Nigeria are highly-experienced with participation in health care service delivery.

### iii. Health Information

The FMOH has a Health Management Information System (HMIS) located in the Health Planning Department which collects, collates, analyses and interprets routine data from the health facilities across the country. There is a national policy on HMIS whose goal is to strengthen the National Health System such that it will be able to provide effective, efficient, quality, accessible and affordable health services that will improve the health status of Nigerians through the achievement of the health related MDGs. Health information from the health facilities are captured using HMIS tools and forwarded from the Local Government Areas through the State Ministries of Health to the Planning Department of the FMOH. The findings form the basis for policy development, review and strategic planning for health intervention. Already most of the NTDs in Nigeria are captured in the HMIS but these are facility-based data at all levels. There is need to update the HMIS forms to include all the NTDs.

Another major source of information on health is from the Integrated Disease Surveillance and Response System (IDSR) which has forty priority diseases including epidemic-prone diseases and some NTDs. Through this system information on these diseases are reported using the different IDSR forms. The Epidemiology Division of the Public Health Department of the Ministry is responsible for the IDSR activities and carries out surveillance on the 40 priority diseases of public health importance and provides weekly reports. The reporting channel also is from health facilities as in the HMIS. This is supplemented by surveys. However, information is often limited in scope and seldom includes data from the private sector. There is need to establish community surveillance.

### iv. Medical Products

The National Agency for Food and Drug Administration and Control (NAFDAC) is the primary regulatory body in charge of control of drug quality and standards in Nigeria and is charged with the mandate to regulate and control quality standards for foods, medicines, cosmetics, medical devices, chemicals, detergents and packaged water imported, manufactured locally and distributed in Nigeria including those used for NTDs. The burden of these endemic NTDs resulting in situations of ill health provide the compelling need for medicines in order to modify the functioning of the body and restore it to normal. There is also a centralised procurement, supply storage, and distribution system in place for medical products in the country which ensure standardization.

### v. Health Financing

Health Financing in Nigeria is from a variety of sources that include budgetary allocations from Government at all levels (Federal, States and Local), loans and grants, private sector contributions and out of pocket expenses. Federal government health expenditure was estimated to have grown three fold from N47.02billion in 2003 to N130.76billion in 2005, and 262 Billion in 2014 while the estimated expenditures for the same period by states grew from N48billion to N78.8billion and that of LGAs nearly doubled from N28.63 billion to N44.64billion.

Household out-of-pocket expenditure remains by far the largest source of health expenditure in Nigeria (about 69%) and in absolute terms, going by available data, increased from N489.79 billion in 2003 to N656.55 billion in 2005. The estimated health expenditure of private firms grew from N20.32 billion in 2003 to N29.67billion in 2005. The contribution from the development partners to health sector in Nigeria is estimated to have increased from N48.02billion in 2003 to N78.78 billion in 2005.

In terms of contribution from different levels of Government, the NHA 2003-05 estimates that the Federal Government contributes above a tenth of the total sum (12.1%), State Governments 7.6%, and LGAs 4.5%. The Household Out-Of-Pocket Expenditure (OOPE), by far remains the largest source contributing to over two thirds (68.6%) while Private Firms contribute (3.1%) and Development Partners (4.1%) as

illustrated in Figures 4 and 5 below. This underscores the huge economic burden of health care expenditure on households, especially the poorer households who are mostly affected by the NTDs. The responsibility to lessen this burden therefore rests with the Government playing a stewardship role to ensure provision of quality and affordable health services to Nigerians in tackling the scourge of the NTDs. For the year 2014, government allocation to the NTDs in the Budget Appropriation was a mere N120 million with less than a quarter being released.

The financing agents of health care in Nigeria, through whom funds are channelled to providers include public agents (Federal ministries and agencies, SMOHs, Hospital management boards, LGAs health departments), National Health Insurance for the formal sector and pilot community health insurance schemes, NGOs and Faith based organizations, private firms' medical units and direct expenditure by households. Their financing contribution to providers is as illustrated in Figure below.

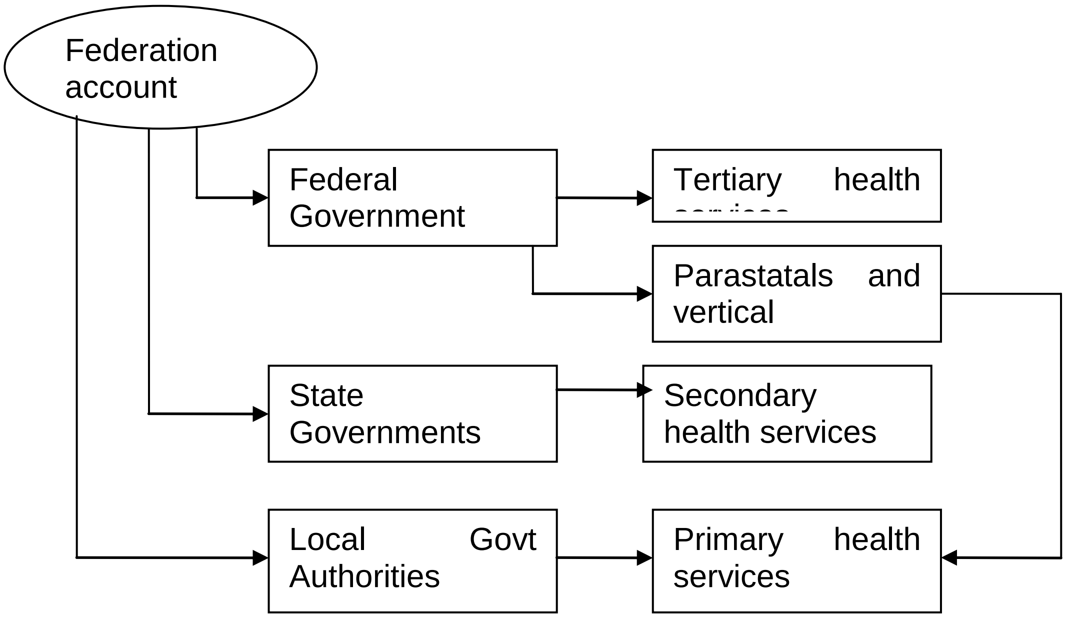


Figure 3: Flow of Health Resources

Fluctuating public funding, problems with management, political interference and poor coordination with State and local governments limit the effectiveness of federal Parastatals and programmes. Even when programmes are well supported they can contribute to fragmentation and duplication, with different programmes operating in the same LGA under different administrative and reporting arrangements-all, making different demands on the same health staff.

***vi. Leadership and Governance***

The Federal Ministry of Health, headed by the Minister, provides the leadership and direction for the implementation of the activities in the health sector. He is assisted by the Minster of State for Health. The Permanent Secretary is the accounting officer and provides the administrative leadership in the Ministry. The Ministry consists of various departments headed by Directors. The NTD Division is domiciled in the Department of Public Health and is saddled with the responsibility of implementing activities for the control, elimination and eradication of the NTDs (see the organogram of the Federal Ministry of Health below). There is a national policy on NTDs which provides the guiding principles for the development and implementation of programmes that will control/eradicate/eliminate these diseases. Its policy statement states that the Federal Ministry of Health will coordinate the control of Neglected Tropical diseases (NTDs) in collaboration with NGDOs, UN agencies, other relevant stakeholders and the private sector. The National Health Act (2014) provides policy directions for effective health leadership and governance. It

addresses issues relating to all building blocks of the health system and gives guidelines for improving health service delivery at all levels.

At the national level there is an NTD division headed by a national coordinator that is in-charge of the day-to-day management of the NTD secretariat, and is assisted by the various programme-specific managers/coordinators. There is a technical committee, the NTD Steering Committee, which oversees programme implementation in the country. Relevant line ministries and government agencies are represented in the Steering Committee which provides the platform for collaboration of the Ministry of Health and other relevant government agencies for the implementation of NTD programme activities. There is also a national NTD task force made up of implementing partners that assists the NTD secretariat in the coordination of programme activities.

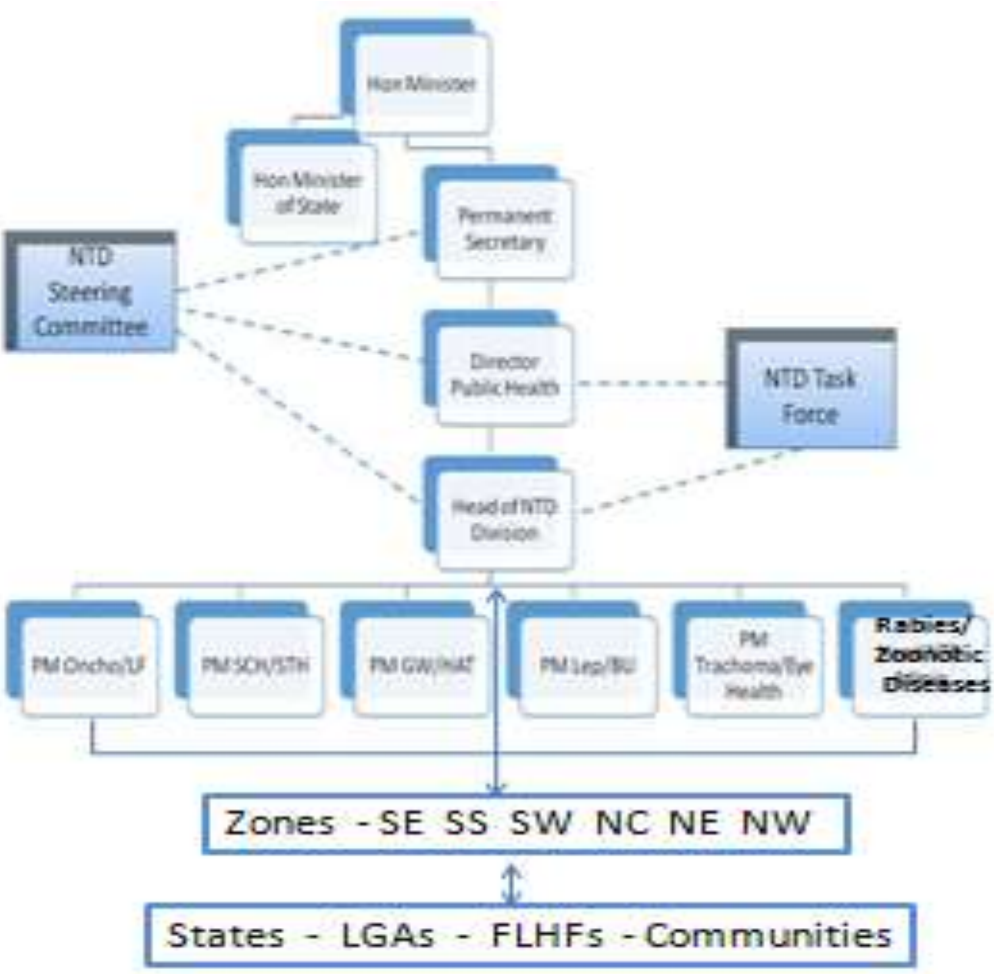


Figure 4: Organogram of the Neglected Tropical Diseases Programme

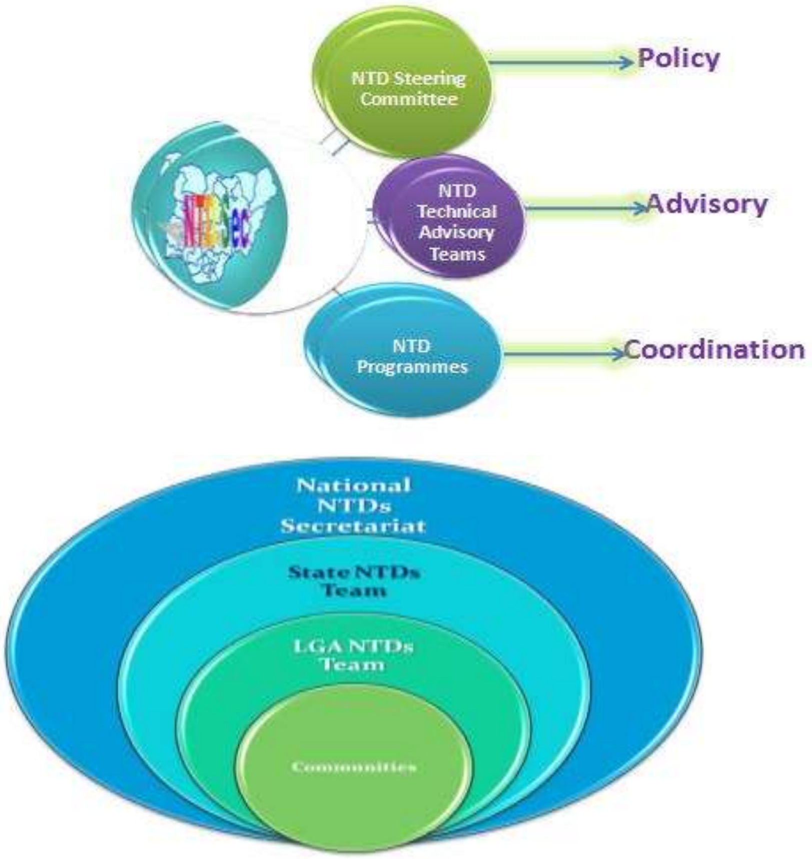


Figure 5: Coordination of the Neglected Tropical Diseases Programme

Primary Health Care: Provision of health care at this level is largely the responsibility of Local Governments with the support of State Ministries of Health and within the overall National Health Policy. Private medical practitioners also provide health care at this level. The local government areas are thus responsible for managing the bulk of Service Delivery Points (SDPs).

Secondary Health Care: This level of health care provides specialized services to patients referred from the primary health care level through out-patient and in-patient services of hospitals for general medical, surgical, pediatric patients and community health services. Secondary health care is available at the district, divisional and zonal levels of the States. Adequate supportive services such as laboratory, diagnostic, blood bank, rehabilitation and physiotherapy are also provided.

Tertiary Care: This level consists of highly specialized services provided by teaching hospitals and other specialist hospitals which provide care for specific diseases such as orthopedic, eye, psychiatric, maternity and pediatric cases. Care is taken to ensure an even distribution of these hospitals. Also, appropriate support services are incorporated into the development of these tertiary facilities to provide effective referral services. Similarly, selected centers are encouraged to develop special expertise in advantage modern technology to serve as a resource for evaluating and adapting these new developments in the context of local needs and opportunities.

To further the overall National Health Policy, governments of the Federation work closely with voluntary agencies, private practitioners and other non-governmental organizations to ensure that the services provided by these other agencies are in line with those of government.

## 1.3 NEGLECTED TROPICAL DISEASES SITUATION ANALYSIS

### 1.3.1 Epidemiology and Burden of Disease

The World Health Organization (WHO) has prioritized 17 neglected tropical diseases that result from four different causative pathogens:

- Protozoa (chagas disease, human african trypanosomiasis [sleeping sickness], Leishmaniases)
- Bacteria (buruli ulcer, leprosy [hansen disease], trachoma, yaws)
- Helminth (cysticercosis/taeniasis, dracunculiasis [guinea-worm disease], echinococcosis, foodborne trematodiases, lymphatic filariasis, onchocerciasis [river blindness), schistosomiasis, soil-transmitted helminthiases), and
- Virus (dengue and chikungunya, rabies)

Though not initially listed as one of the NTDs mycetoma, a chronic, progressively destructive morbid inflammatory disease usually of the foot, is believed to be a significant health problem especially in some countries located in the mycetoma belt. Except for chagas disease the other NTDs are known or suspected to be prevalent in Nigeria. However, infections from cysticercosis/taeniasis, echinococcosis, and foodborne trematodiases are believed not to be of significant public health concern.
Currently, the Federal Ministry of Health are addressing the following NTDs based on outcomes of mapping surveys, case searches or high-suspicion index:

1. **Preventive Chemotherapy NTDs:** Lymphatic Filariasis, Onchocerciasis, Schistosomiaisis, Soil Transmitted Helminths, and Trachoma.
2. **Case-Management NTDs:** Leprosy, Buruli Ulcer, HAT, GWD, Lymphoedema, Trichiasis, Rabies, Leishmaniasis, Yaws, Dengue and Mycetoma

#### *1.3.1.1* Onchocerciasis

Onchocerciasis has been mapped and is prevalent in all States of Nigeria although endemcity is low in such States as Lagos, Sokoto, Bayelsa and Rivers. It is estimated that about 50 million persons in over 40,000 communities are at risk in Nigeria. Until recently, it was a major cause of blindness in many rural communities across the nation.

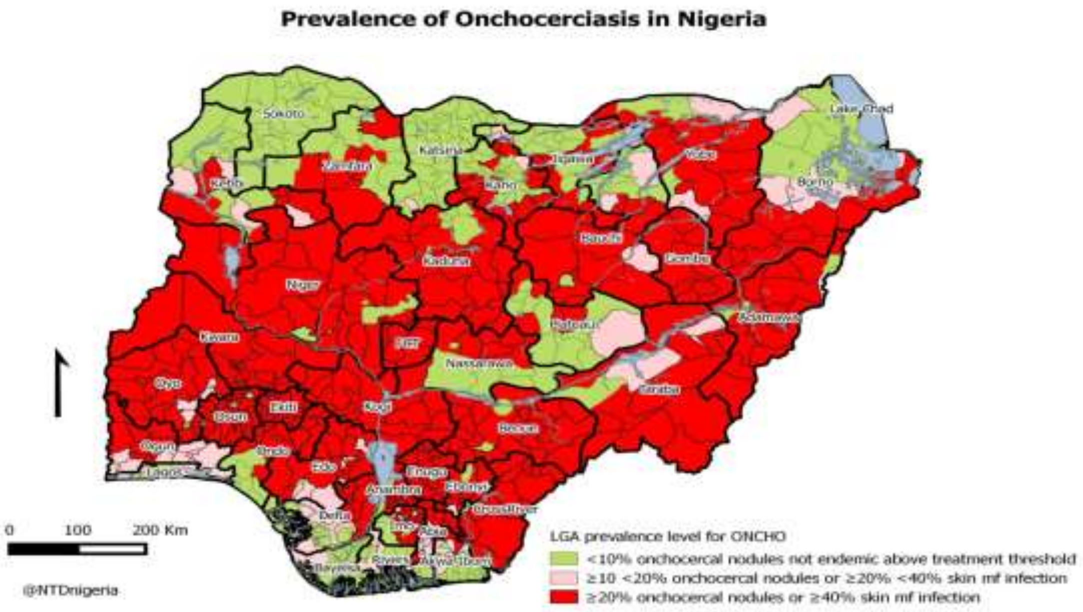


Figure 6*: Onchocerciasis Prevalence Map*

### *1.3.1.2* ***Lymphatic Filariasis***

Globally, Nigeria is ranked 3rd highest with LF disease burden. Over 114 million Nigerians are at risk of the disease. In 2003, the NLFEP started LF mapping in the country and so far 35 States and Federal Capital Territory (FCT) have completed mapping in all their LGAs using Immuno Chromatographic Test (ICT) cards. LF prevalence has been determined in 761 out of 774 LGAs of 36 States and FCT. Out of the mapped LGAs, 574 LGAs are endemic and 187 LGAs are non-endemic. As at 2013, 239 lymphodema and 290 hydrocele cases have been reported from mapping surveys carried out in the country.

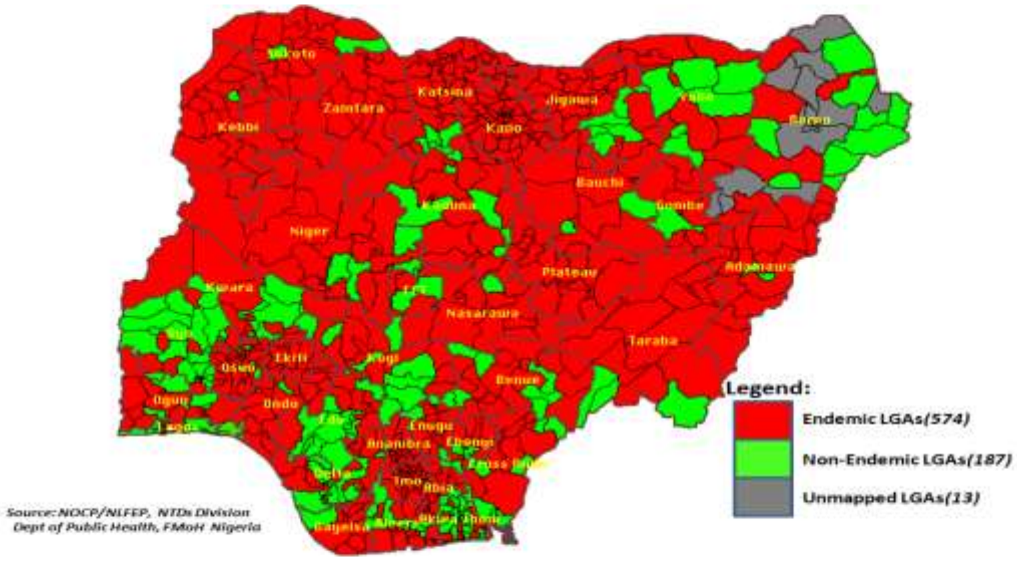


Figure 7: Lymphatic Filariasis endemicity map by LGA in Nigeria

### *1.3.1.3* ***Loasis***

*Loa loa* is a filarial parasite transmitted by the vector *Chrysops* which is mainly found in the rain forest zones of West and Central Africa. Serious adverse events have been reported, sometimes fatal, in areas where there are high levels of microfilaremia and in which Ivermectin is being distributed for the control of Onchocerciasis. For the elimination of LF, ivemectin and albendazole medicines are used. The Mectizan

Drug Donor and WHO recommend that countries where LF and Onchocerciasis are co-endemic with loaisis should carry out survey to determine prevalence of L.loa before treatment scale up.

LF and onchocerciasis are endemic in Nigeria. The Rapid Assessment Procedures for Loa loa (RAPLOA) is the tool used for L.loa study in the country. As at 2014, L. loa prevalence has been known in 147 LGAs of 16 States. Out of this, 19 LGAs in 10 States have at least a community with history of eye worm >40%. RAPLOA will be carried out in the remaining 195 ivermectin naïve LGAs by 2015.

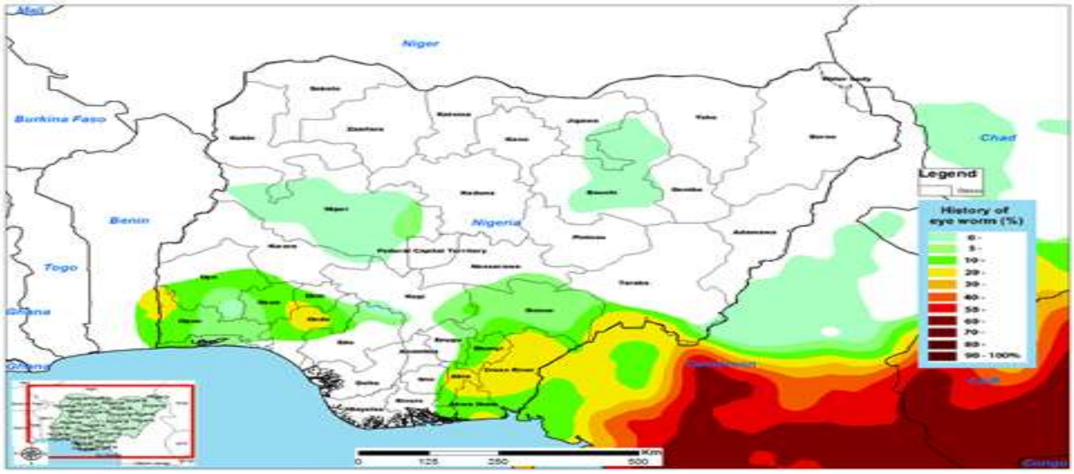


Figure 8: Estimated prevalence of eye worm history in Nigeria(Courtesy APOC)

### *1.3.1.4* ***Schistosomiasis***

Schistosomiasis (or blood fluke infection) is caused by trematodes belonging to the genus *Schistosoma*. Three types of this worm cause human schistosomiasis, two of which occur in Nigeria. These are *Schistosoma. haematobium* which causes urinary Schistosomiasis and *Schistosoma mansoni* that causes intestinal Schistosomiasis.

In Nigeria Schistosomiasis is a disease of considerable and growing importance due to inadequate potable water and activities related to water resource development schemes for irrigation, fishing and hydro-electricity. Generally, the disease mainly affects the rural poor and the vulnerable age group. School children are the major victims of the disease. Schistosomiasis can cause diseases such as cancer of the bladder, anaemia, liver dysfunction etc. Nigeria has the highest burden of this disease in Africa. About 116 million out of the estimated 555 million Africans are at risk as at 2006 (WHO 2008).

Integrated epidemiological mapping has been conducted in a total of 724 LGAs in 32 States and Federal Capital Territory. The States are: Plateau (17 LGAs), Nassarawa (13 LGAs), Lagos (20 LGAS), Ekiti (16 LGAs), Ondo (18), Sokoto (23 LGAs), Niger (25 LGAs), Benue (23 LGAs), Anambra (21 LGAs), Ogun (20) Zamfara (14), Enugu (18 LGAs), Ebonyi ( 13 LGAs), Osun (31), Oyo (33), Imo (27), Abia (17),Edo (18), Delta (25), Cross River(18), Rivers(23), Bayelsa (8), Jigawa (27 LGAS), AkwaIbom (31), Kaduna(23), Kebbi (21), Katsina (34), Kano (44), Kwara (16), Taraba (16), Gombe (11), Bauchi (20), Kogi (21) and Federal Capital Territory (6 LGAs). Currently Borno, Adamawa and Yobe (2 LGAs) States are the only States where Schistosomiasis has not been completely mapped, due to security challenges in the North east zone of the country. Provisional prevalence ranged from 0%- 84% in the LGAs mapped.

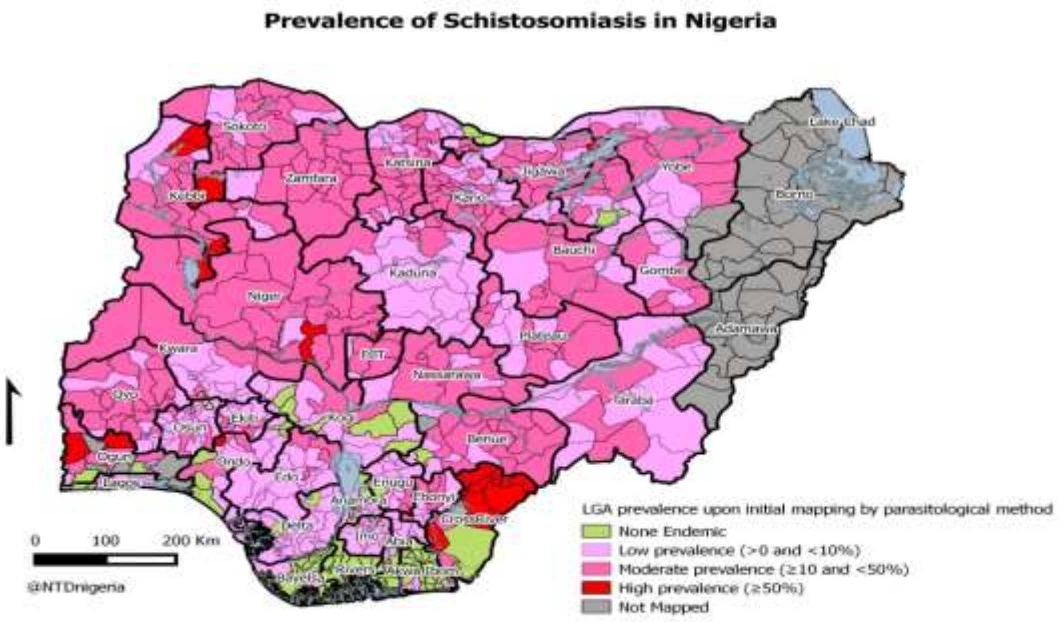


Figure 9: Schistosomiasis Prevalence Map

### *1.3.1.5 **Soil Transmitted Helminthiasis***

STH's are among the Neglected Tropical Diseases which are endemic in Nigeria and the country is among those with the highest burden of this disease in Africa. The causative agent of soil transmitted helminthiasis include any of the following worms; *Ascaris lumbricoides, Trichuris trichiuria, Ancylostoma duodenale and Necator americanus.* It affects mainly children- causing anemia, Vitamin A deficiency, malnutrition, loss of appetite, retarded growth, reduced ability to learn, etc in them.

Integrated mapping for Soil Transmitted Helminths has been conducted in 708 LGAs in 32 States. The States are: Plateau (17 LGAs), Nassarawa(13 LGAs), Lagos (20 LGAS), Ondo (18), Sokoto (23 LGAs), Niger (25 LGAs), Benue (23 LGAs), Anambra (21 LGAs), Ogun (20) Zamfara (14), Enugu (18 LGAs), Ebonyi ( 13 LGAs), Osun (31), Oyo (33), Imo (27), Abia (17),Edo (18), Delta (25), Cross River(18), Rivers(23), Bayelsa (8), Jigawa (27 LGAS), AkwaIbom(31), Kaduna(23), Kebbi (21), Katsina (34), Kano (44), Kwara (16), Taraba (16), Gombe (11), Bauchi (20), Kogi (21) and Federal Capital Territory (6 LGAs). Currently Borno, Ekiti, Adamawa and Yobe (2 LGAs) States are the only States where STH has not been completely mapped, due to security challenges in the North east zone and political instability in Ekiti State. Prevalence ranged from 0%- 87% in the LGAs mapped.

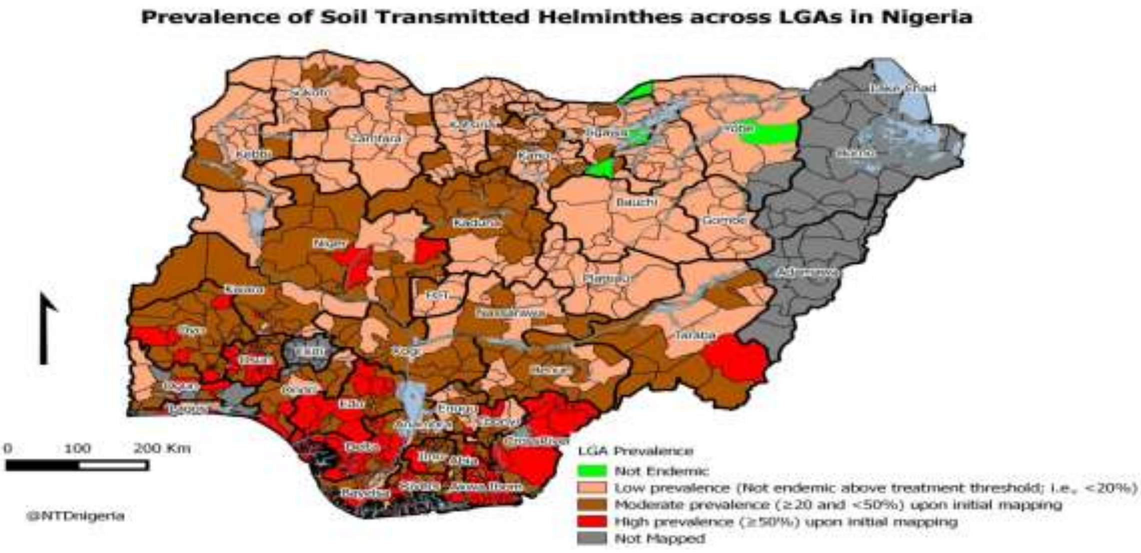


Figure 10: STH Prevalence Map

#### *1.3.1.6* ***Human African Trypanosomiasis***

In 1960 HAT cases were reported from several endemic foci across the country. As a result of interventions by the Nigeria Institute of Trypanosomiasis Research (NITR), the number of cases from these foci declined considerably. Between 1989 and 1996, 3,583 persons were examined in Abraka, HAT endemic focus of Delta state out of which 104 and 359 were sero-positive. Only 127 of these cases could be treated with 7 fatalities being recorded. In another study conducted with a total of 4,966 persons screened 10% were sero positive out of which 6% had the disease.

Recent studies in 2006 confirmed that HAT transmission is still active in the Abraka endemic focus and the same may well be the situation in many other parts of Nigeria. In 2011, 3 positive cases were recorded (2 in Delta and 1 in Niger states) More recently, HAT was found to occur in several LGAs in Delta and Niger States of Nigeria. Also some sero- positive were recorded in Edo which calls for a more robust screening.These results are an indication that HAT is still endemic in the country and there is an urgent need to determine the current level of endemicity throughout the country.

#### *1.3.1.7* ***Dracunculiasis***

Nigeria was among the top three endemic countries in the world at the time Guinea Worm Eradication Programme was launched in the country in 1988. The global campaign to eradicate GWD was enunciated within the framework of the United Nations International Drinking Water Supply and Sanitation Decade (1981 – 1990). It is in this context that Nigeria launched the Nigeria Guinea worm Eradication Programme (NIGEP).

Nigeria reported over 653,000 cases of Dracunculiasis (Guinea worm disease (GWD)) during the case search in 1988/1989. Nigeria entered the pre-certification phase and fulfilled WHO criteria for certification, followed with the inauguration of the National Certification Committee on Guinea worm Disease Eradication (NCC-GWDE) in May 2005. NIGEP is a partnership comprising principally of these institutions: The Carter Center (TCC), UNICEF, Yakubu Gowon Center (YGC), WHO. Although NIGEP target elimination dates have been inevitably shifted several times since the first target date of 1995, the concerted collaborative efforts of the NIGEP partnership at all levels has resulted in very significant reduction in cases and number of villages reporting cases in Nigeria in the 20 years of the programme up till 2012 as shown in Figure below. Peak GWD transmission season in the south is during the dry season

in November to transmission season in the north is June to October when stagnant ponds are formed from the rain water.

Figure 11: No of Reported Guinea Worm Diseases in Nigeria 1987-2012

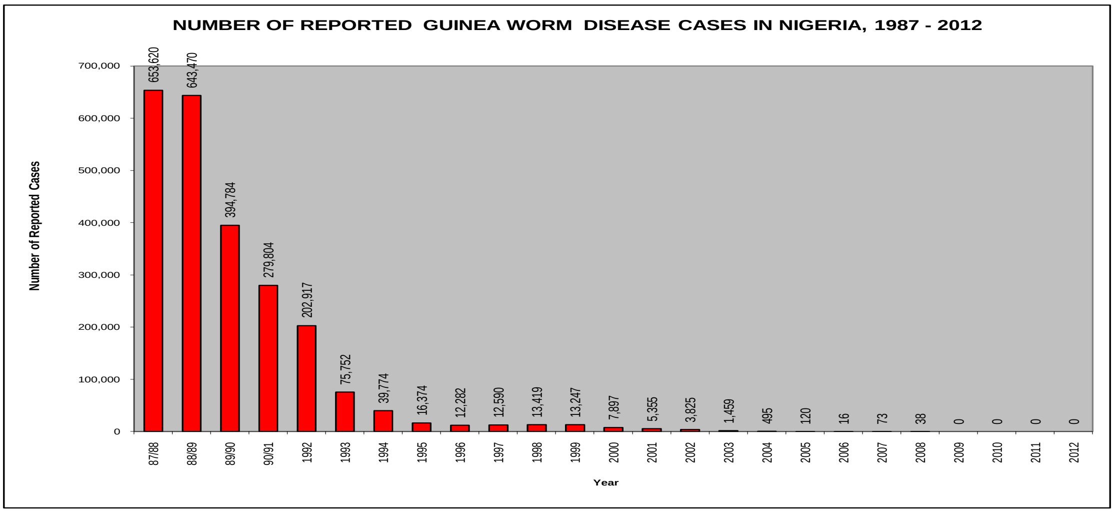


### *1.3.1.8* ***Trachoma***

Trachoma is one of the causes of avoidable blindness only next to cataract. Globally it is the leading infectious cause of blindness responsible for blindness in 1.2 million people worldwide. According to the findings of the Nigeria Blindness and Low Vision Survey (2005-2007) 4.2 % of blindness in Nigeria is due to trachoma. The disease is highly prevalent in the northern part of Nigerian which falls within the trachoma belt. It is found in the most vulnerable communities disproportionately affecting children and women. The repeated infection with trachoma, generally among children, leads to scarring of the tissue lining the underneath of the eyelid. This scarring causes the eyelid to eventually turn inward allowing the eyelashes to rub against and abrade the cornea of the eye. This painful condition called trichiasis (TT) can result in irreversible blindness. The backlog of un-operated trichiasis cases in Nigeria is estimated to be in excess of two hundred and thirteen thousand persons; these people are on the verge of blindness.

Trachoma also has a profound impact on the socioeconomic life of family members and communities being closely associated with poverty and low standard of living characterized by overcrowding, poor environmental and personal hygiene. These conditions are prevalent in most rural communities and urban slums in the developing countries.

Nigeria has nearly finished its entire epidemiologic mapping of all relevant Local Government Areas (LGAs) suspected to have trachoma. Of the 37 States, 22 states have been surveyed and 220 LGAs in 15 States with a population of 57,995,631 in the “trachoma belt” have been found to have trichiasis prevalence of public health significance. However, an estimated 8 million people living in in 123 trachoma endemic LGAs are in need of annual Mass Administration of antibiotics. The only remaining States left to be mapped due to insecurity are Adamawa and Borno. Mapping will be carried out in these two States (44 LGAs) once the security situation improves.

Latest data shows that there is an estimated backlog of 257,286 trichiasis cases with 213,201 patients requiring surgical intervention to meet the UIG for trichiasis.

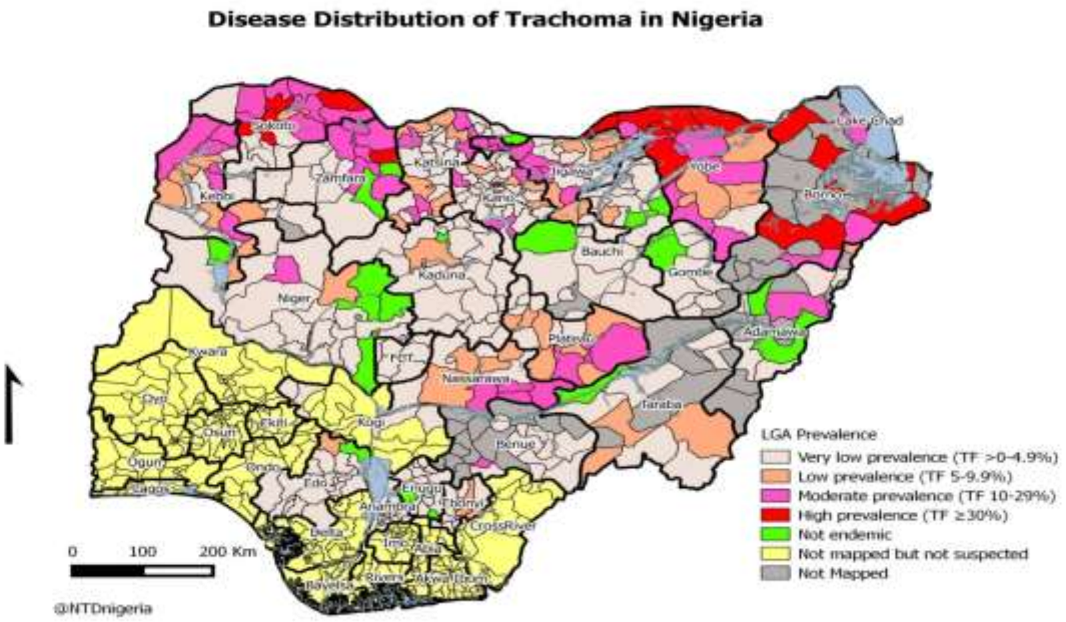


Figure 12: Trachoma Prevalence Map

#### *1.3.1.9* ***Leprosy***

Nigeria is still one of the leprosy endemic countries in the world.

The WHO elimination target of 1 case per 10,000 population has been achieved at the national level and in all Zones from a total of 111,788 patients recorded in 1989. With both prevalence rate and case detection rate below 0.5 per 10,000, Nigeria may well be described as low endemic for leprosy. Despite the fact that Nigeria has reached the elimination target the number of new cases reported yearly in the last five years continues to be high (more than 3,000 cases per year) and there still exists backlog of grade 2 disabilities. About 12% of new cases have grade two deformities, which indicate late patient presentation. The proportion of children with the disease is also high (9.4% in 2005) indicating ongoing transmission of the disease in the communities. It is pertinent to note that at some States are yet to attain the elimination target. In addition, substantial numbers of persons affected by leprosy require physical and socioeconomic rehabilitation. A total of 6,906 cases remained on the leprosy registers nationally at the end of 2008. Leprosy cases are reported from all states in the federation including FCT.

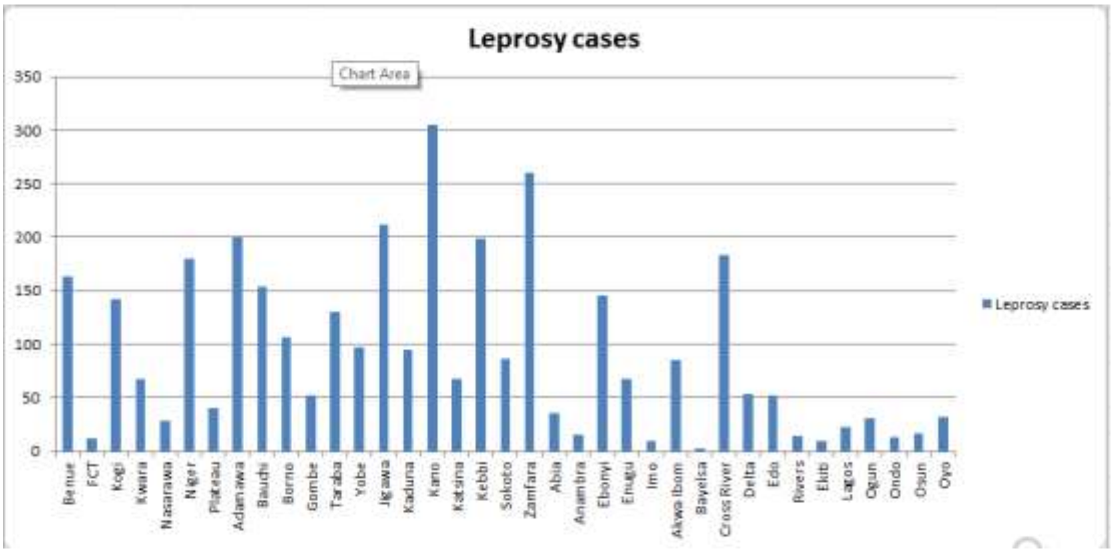


Figure 13: Chart of New Leprosy Cases in States (2013)

A total of 3385 cases were detected in 2013, 93%of them were classified the infectious MB cases and 34% were females. This gives a case detection rate of 0.20 per 10,000 population or 2 cases in every 100,000 Nigerians. The proportion of children among new cases detected is 9% nationally in 2013 (see figure 10). Despite improvement seen in case finding of leprosy cases over the years, many new cases already with visible deformities (WHO Grade 2 disabilities) continue to report for treatment. Compared to the target of 5%, the national grade 2 disability rate of 13% at the end of 2013 shows the disability rate of new cases is high and new case detection occurs relatively late.

*1.3.1.10* ***Buruli Ulcer***

Buruli ulcer (BU) is a chronic, indolent, necrotizing infectious disease of the skin caused by *Mycobacterium ulcerans*. Even though the disease is curable, it still disables, deforms, debilitates and stigmatizes due to late diagnosis and case management. The distribution of the disease is patchy often in relatively inaccessible areas especially aquatic environments. About 70% of those affected are children below 15 years of age. There is no sex or race preference. The exact mode of transmission is still unknown.

Buruli ulcer was first reported in Nigeria in 1967 by Gray, Kingama and Kok among the Tiv in Benue and Bambur in Adamawa States. The disease was also reported among Caucasians living on the campus of University of Ibadan who were swimming in artificial lake. Another set of 10-15 cases were reported yearly in TB and Leprosy Hospital, Ogoja in Cross River State. Prof. Onuigbo (1970 – 1990) reported 43 cases that came from Enugu in Enugu State and Afikpo in Ebonyi State. Many Nigerians from Ogun, Edo, Oyo and Cross River States have been treated in neighbouring countries. BU has been reported from the following States: Ogun, Akwa Ibom, Anambra, Cross River, Ebonyi, Benue, and Enugu.

Nigeria is a Buruli ulcer endemic country. It is located between two confirmed Buruli ulcer endemic countries (Benin and Cameroon). Located on the same latitude these countries share the same climatic and geographical features such as rivers and marshland. They also share the same rural economic activities as farming, mining and forestry. However, only few cases were reported in medical and scientific papers.

A 2006 assessment to confirm BU suspected cases in Ogun State indicated that of 37 suspected cases examined 9 active and 5 inactive cases were confirmed. Four of the 9 active cases were confirmed by PCR at Institute of Tropical Medicine, Antwerp, Belgium. Another assessment visit to Ogun State was carried out in January, 2009. Two communities were visited and a number of suspected cases were identified. Four cases were confirmed by PCR.

Although most cases of Buruli ulcer occur in poor rural communities,there is need to conduct mapping and strengthen BU data collection to aid effective planning and implementation of activities to control Buruli Ulcer in Nigeria. There is also need to establish Polymerase Chain Reaction (PCR) suites for in-country Buruli Ulcer confirmation.

#### *1.3.1.11* ***Rabies***

Rabies is a neglected zoonotic disease that is transmitted as a result of bites from rabid animals. It is widely distributed across the entire continent. However, the Americas and some western countries such as UK, Australia etc., are reported as being free of the disease. Infected wildlife species, including bats, can transmit rabies to humans, but the total number of such cases remains limited compared with the annual number of human deaths caused by dog-transmitted rabies.

By contrast, canine rabies predominates in most of the developing countries of Africa and Asia, where the greater burden of human rabies falls. More than 90% of cases of human rabies are transmitted by dogs; most deaths occur. Globally, rabies is the tenth leading cause of death due to infections in humans. More than 99% of all human deaths from rabies occur in the developing countries and almost half of those dying from rabies and requiring rabies immunoglobulins and human anti-rabies vaccine (H-ARV) are the target risk age group of children less than 15 years, animal handlers and veterinarians are mostly affected.

Though cases of rabies have been reported in various parts of Nigeria these have not been appropriately documented nor has the annual incidence been recorded.

#### *1.3.1.12* ***Leishmaniasis***

This disease is caused by any of a number of species of protozoa in the genus *Leishmania*. There are several major clinical types of this infection including cutaneous, diffuse cutaneous, muco-cutaneous and visceral leishmaniasis. In Nigeria the type that is common is cutaneous leishmaniasis (CL), with evidence of visceral leishmaniasis (VL) being available. CL, also known as oriental or tropical sore, occurs in various parts of the world, mainly in tropical and subtropical regions. In the African continent, CL due to *L. major,L tropica* and *L. aethiopica* is unevenly distributed from the northern to the southern areas of the continent**.**

A common estimate of the worldwide annual incidence is 600,000 newly reported clinical cases, an overall prevalence of 12 million cases and an estimated population at risk of about 350 million in 88 countries. There is probably an even greater difference between the number of cases actually occurring and the number usually reported due to factors such as discontinuous distribution of transmission sites, numerous cases that are undiagnosed, some misdiagnosed and the number of asymptomatic cases.

Five cases of cutaneous leishmaniasis were recorded in a pilot mapping for the disease conducted in Nasarawa State in 2014. In addition there are articles by researchers on cutaneous, diffuse cutaneous and visceral leishmaniasis. There is need to conduct mapping of the disease across the entire country to obtain baseline data on its burden and spread in the country

#### *1.3.1.13* ***Yaws***

Yaws is one of the chronic bacterial infections (endemic treponematoses) often affecting the skin and presenting skin lesions that are caused by treponemes. It is transmitted primarily through skin contact with an infected person. A single skin lesion develops at the point of entry of the bacterium after 2-4 weeks, and where this is not treated, multiple lesions appear all over the body. The disease occurs mainly in poor communities in warm, humid tropical regions of Africa, Asia, Latin America and Western Pacific. About 75% of those affected are children under 15 years.

### *1.3.1.14* ***Mycetoma***

Mycetoma is a chronic, progressively destructive morbid inflammatory disease usually of the foot but which can affect any part of the body. Infection occurs when the causative organism enters the body through minor trauma or a penetrating injury, commonly thorn pricks. The disease affects mainly young adults especially those who walk barefooted and are manual workers. Mycetoma was first reported in the mid-19th century in the Indian town of Madura, and therefore was initially called Madura foot.

The disease commonly, occurs in developing countries among people of low socioeconomic status with manual workers such as agriculturalists, labourers and herdsmen being the worst affected. The World Health Organization has described mycetoma as a badly neglected disease without any accurate data on its incidence and prevalence. *Mycetoma* is characterized by a triad of painless subcutaneous mass, multiple sinuses and discharge containing grains. It usually spreads to involve the skin, deep structures and bone resulting in destruction, deformity and loss of function, which may be be fatal. Mycetoma commonly involves the extremities, back and gluteal region.

The causative organisms of *mycetoma* are distributed worldwide but are are found more within the 'mycetoma belt'. Though Nigeria is not stated to be within the 'mycetoma belt' that includes the Bolivarian Republic of Venezuela, Chad, Ethiopia, India, Mauritania, Mexico, Senegal, Somalia, Sudan and Yemen, it shares boundaries with Chad which is part of the belt. Therefore, there is need for accurate information on the incidence and distribution of mycetoma in the country.

### *1.3.1.15* ***Dengue***

Dengue, a viral disease, is transmitted by the bite of a mosquito infected with one of the four dengue virus serotypes, and is not known to be transmitted directly from person-to-person. A febrile illness it affects infants, young children and adults with symptoms ranging from mild fever, to incapacitating high fever, with severe headache, pain behind the eyes, muscle and joint pain, and rash appearing 3-14 days after the infective bite. Documented as one of the most rapidly spreading mosquito-borne viral disease in the world with its incidence increasing 30-fold over the last 50 years, there are an estimated 50 million dengue infections annually in both urban to rural settings. There is currently no vaccine or any specific medicine to treat dengue.

Nigeria lies within the 'dengue' belt (see figure below), and Carey DE et al (1971) first reported the isolation of DEN-1, -2 and -3 from samples taken from humans in Nigeria in the 1960s.

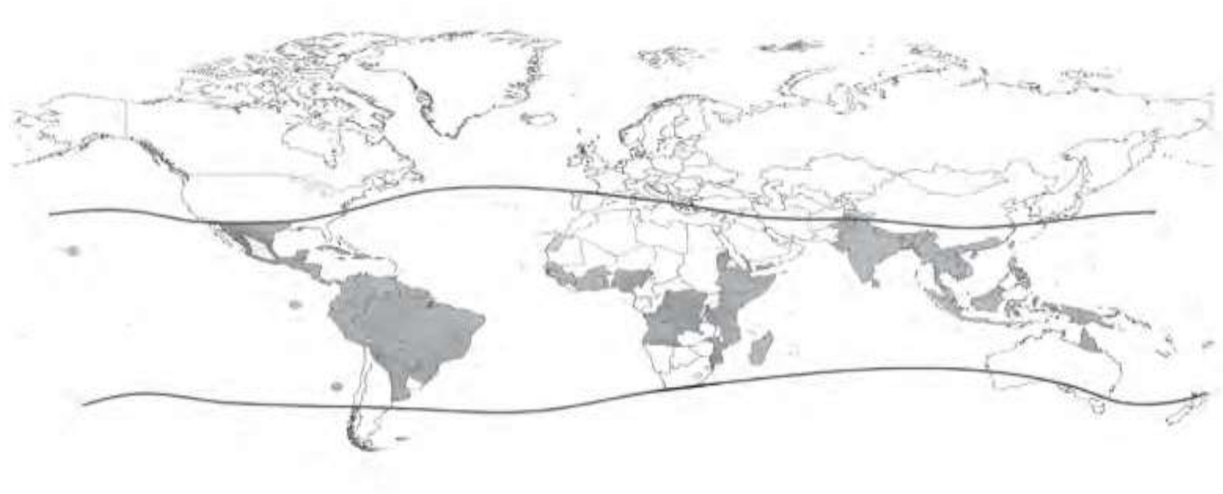

Figure 14: Countries/areas at risk of dengue transmission, 2008

Co-endemicity of Neglected Tropical Diseases in Nigeria

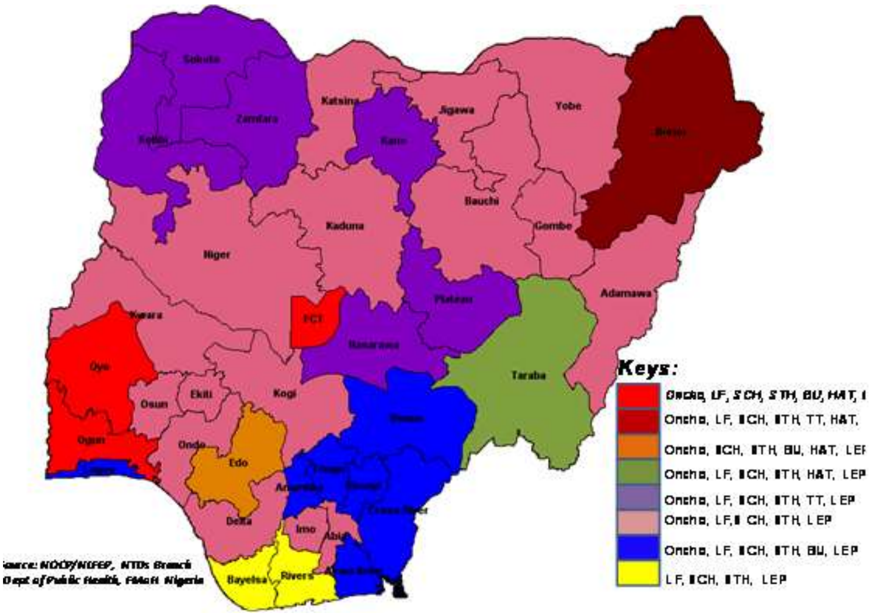


Figure 15: NTDs Co endemicity Map – Nigeria

Figures 16: PC-NTDs Co-endemicity Maps by State

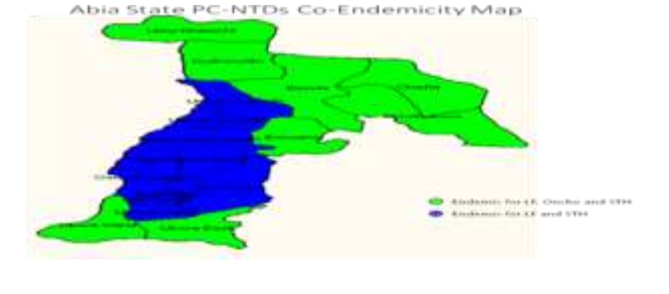


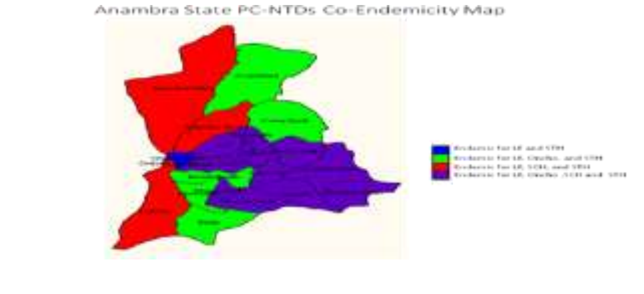


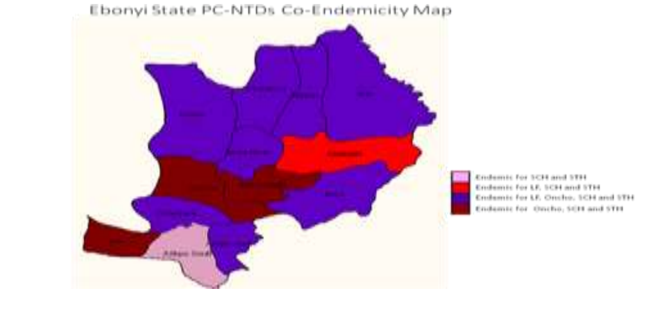


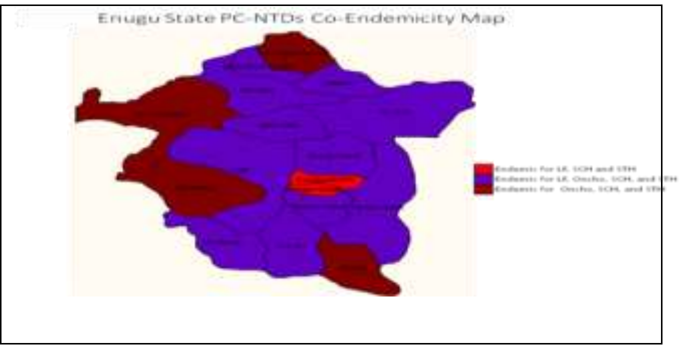


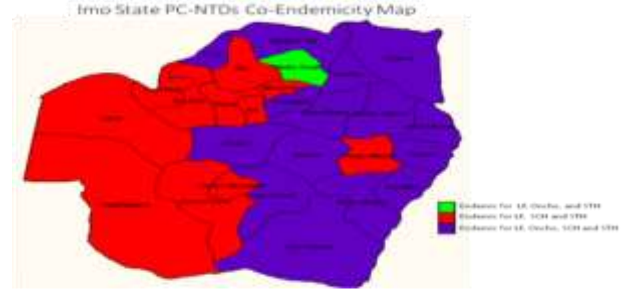


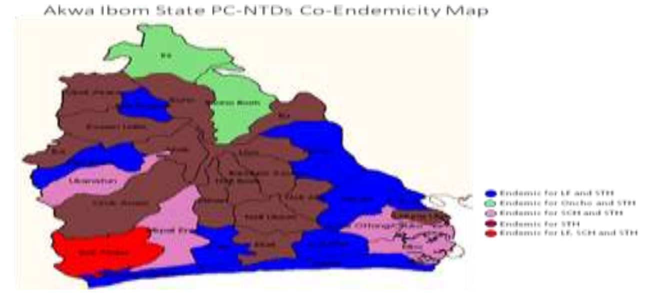

Bayelsa State PC-NTDs Co-Endemicity Map

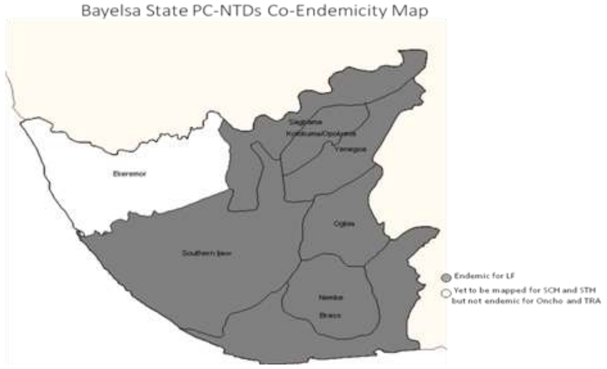


Cross River State PC-NTDs Co-Endemicity Map

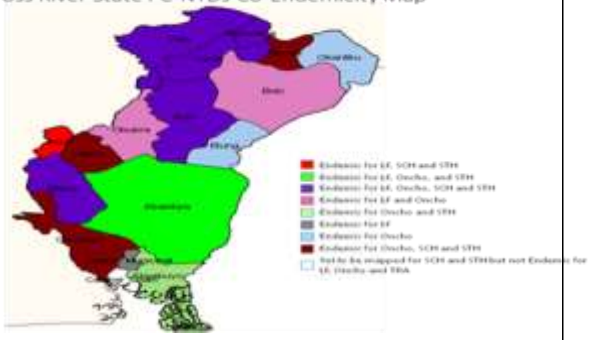

Delta State PC-NTDs Co-Endemicity Map

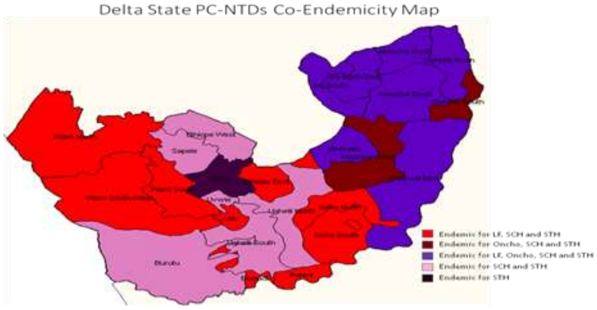


Edo State PC-NTDs Co-Endemicity Map

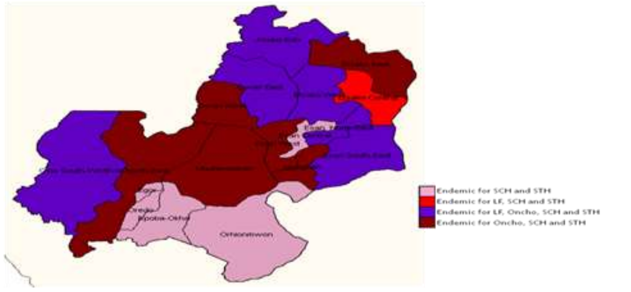


Rivers State PC-NTDs Co-Endemicity Map

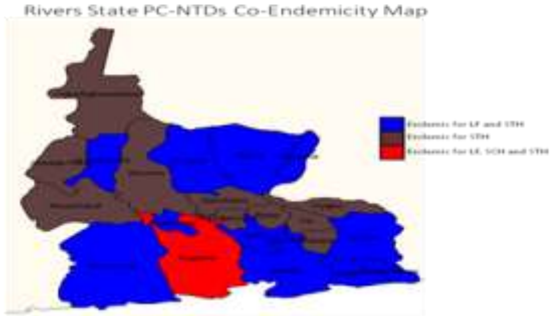

Ekiti State PC-NTDs Co-Endemicity Map

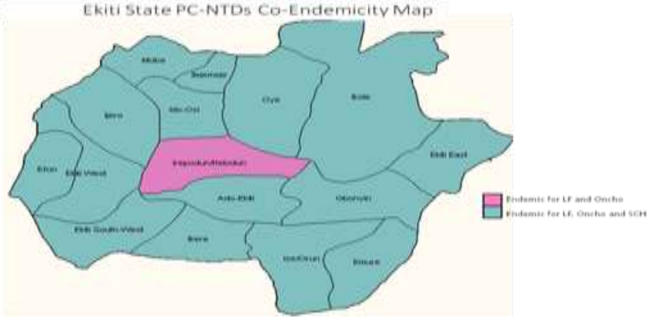

Lagos State PC-NTDs Co-Endemicity Map

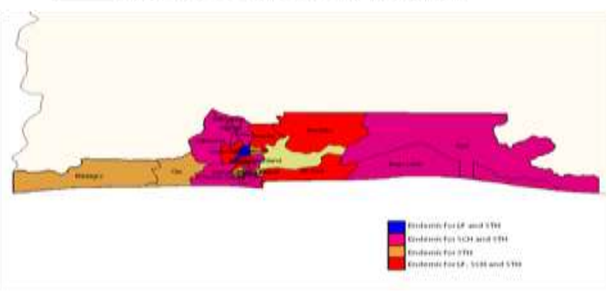

Ogun State PC-NTDs Co-Endemicity Map

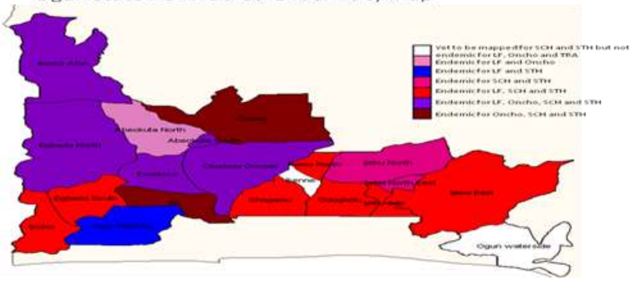

Ondo State PC-NTDs Co-Endemicity Map

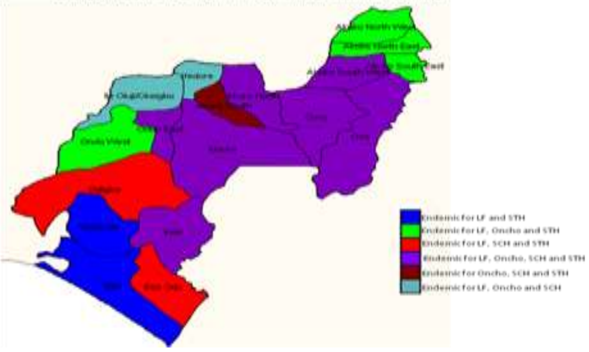


Osun State PC-NTDs Co-Endemicity Map

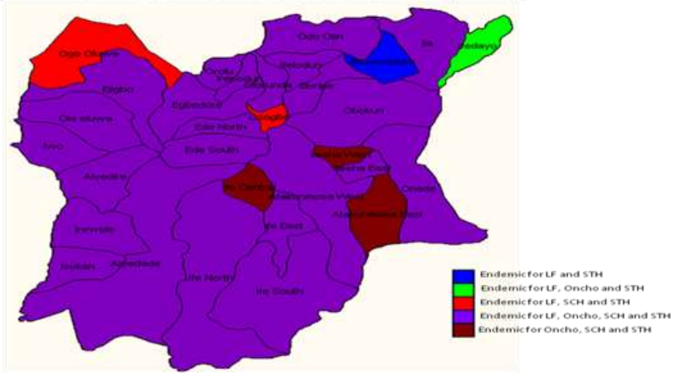


Oyo State PC-NTDs Co-Endemicity Map

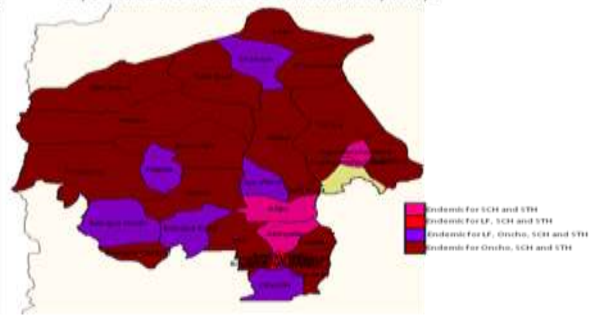


FCT PC-NTDs Co-Endemicity Map

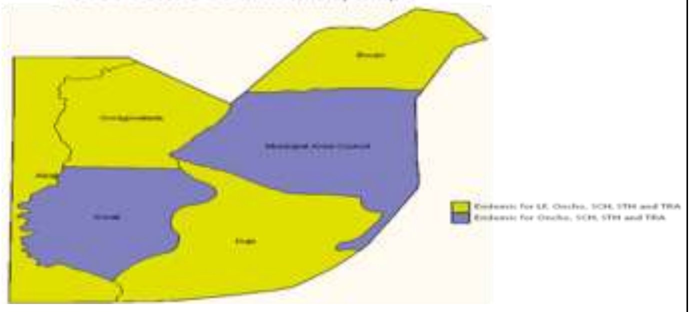

Benue State PC-NTDs Co-Endemicity Map

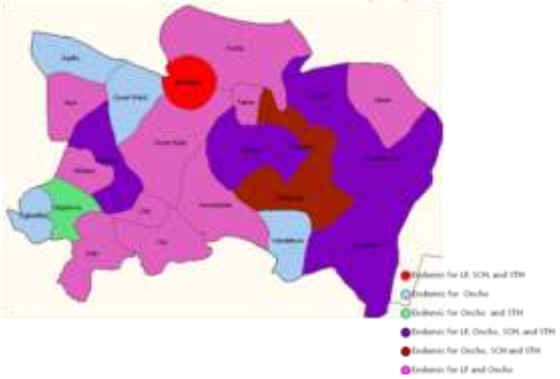

Kaduna State PC-NTDs Co-Endemicity Map

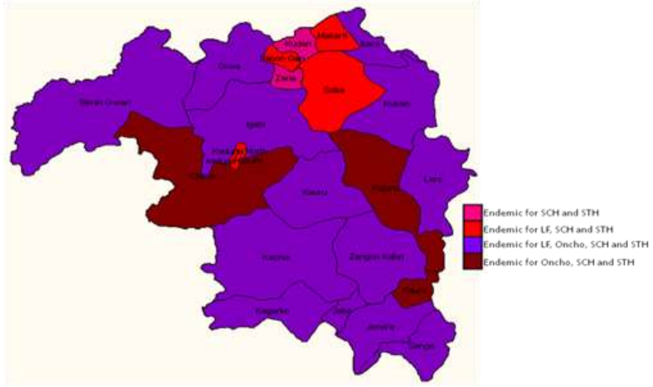

Kogi State PC-NTDs Co-Endemicity Map

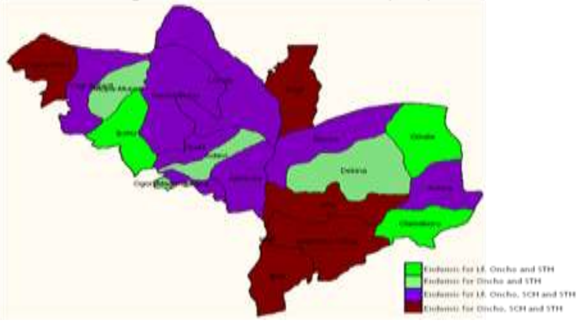

Kwara State PC-NTDs Co-Endemicity Map

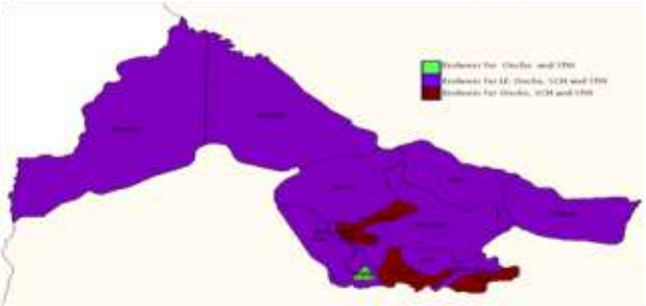

Nasarawa State PC-NTDs Co-Endemicity Map

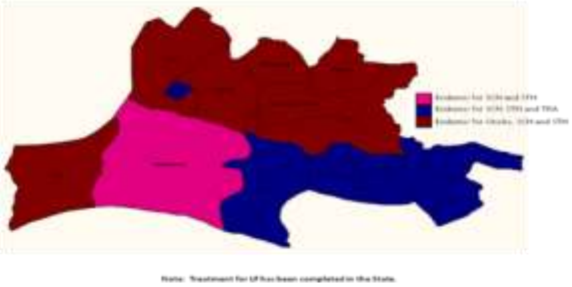

Niger State PC-NTDs Co-Endemicity Map

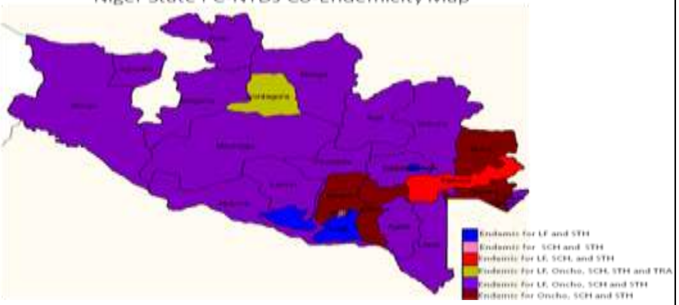

Plateau State PC-NTDs Co-Endemicity Map

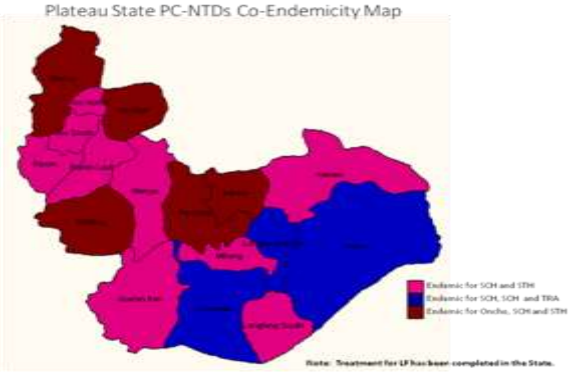

Adamawa State PC-NTDs Co-Endemicity Map

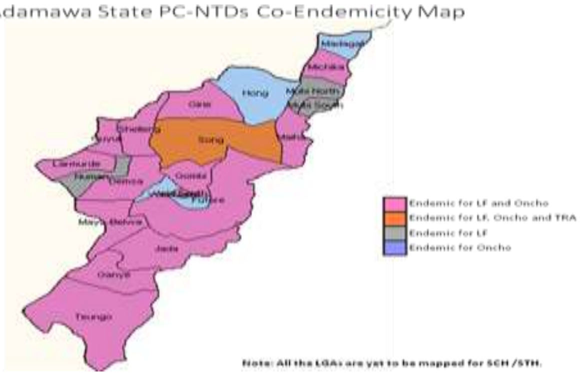


Bauchi State PC-NTDs Co-Endemicity Map

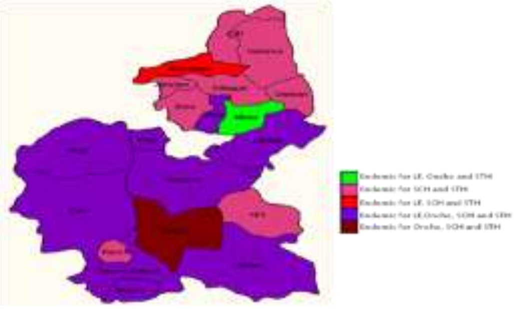

Borno State PC-NTDs Co-Endemicity Map

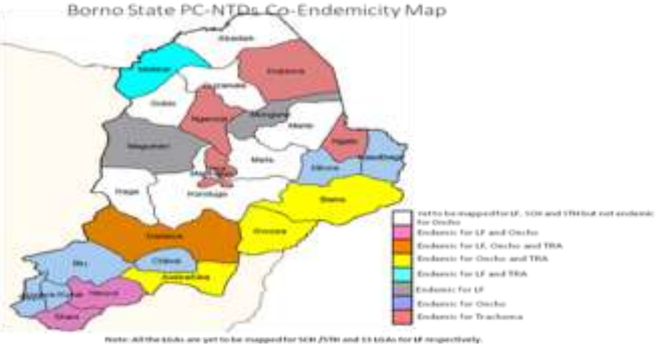

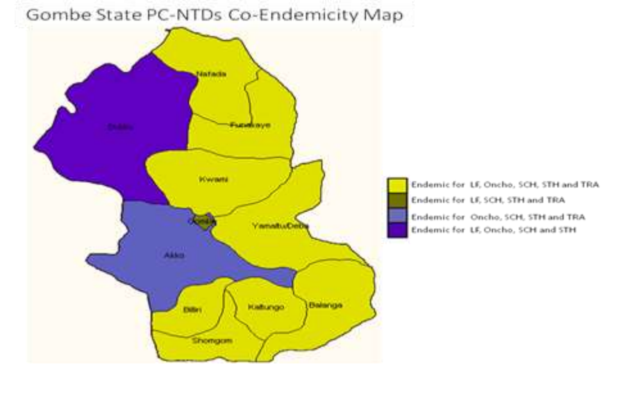
Gombe State PC-NTDs Co-Endemicity Map

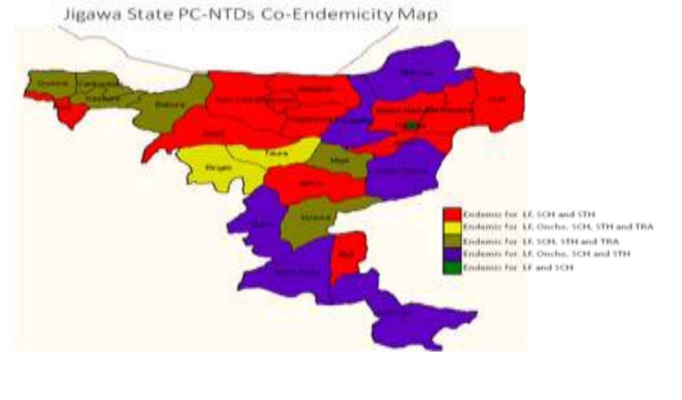
Jigawa State PC-NTDs Co-Endemicity Map

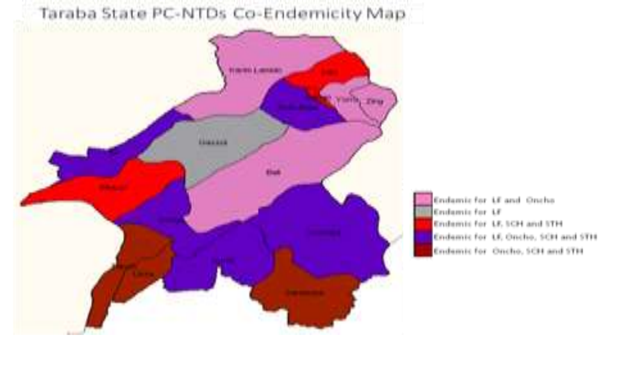
Taraba State PC-NTDs Co-Endemicity Map

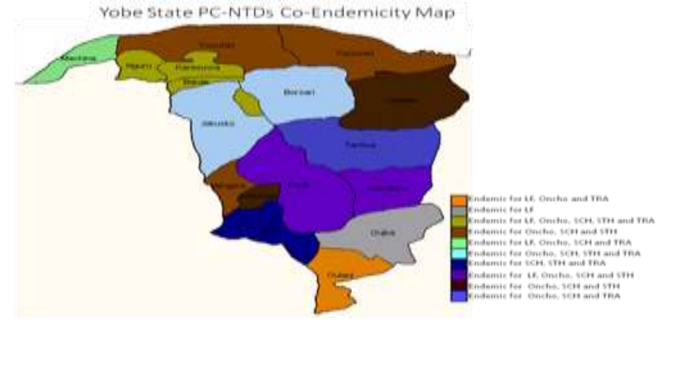
Yobe State PC-NTDs Co-Endemicity Map

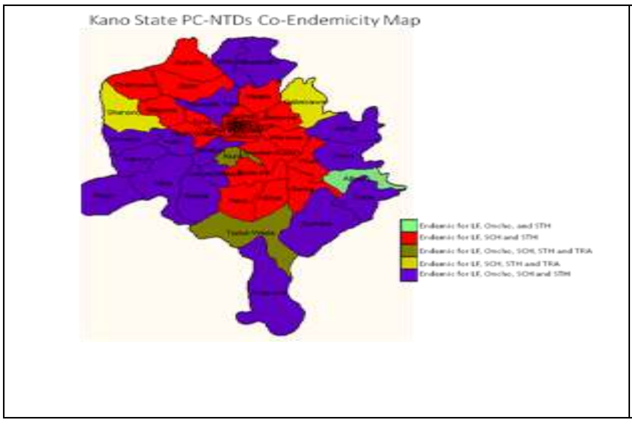
Kano State PC-NTDs Co-Endemicity Map

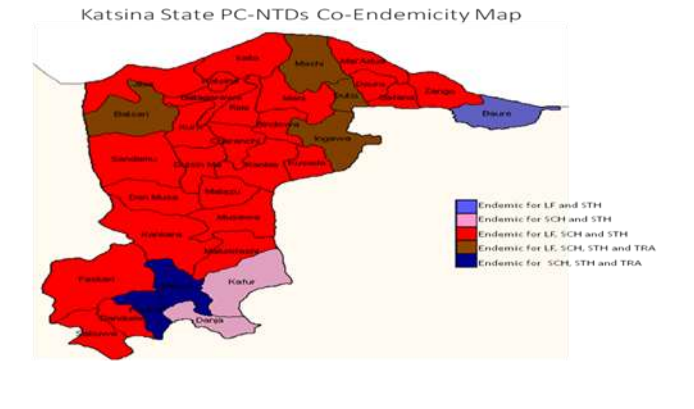
Katsina State PC-NTDs Co-Endemicity Map
Endemic for LF and STH
Endemic for SCH and STH
Endemic for LF, SCH and STH
Endemic for LF, SCH, STH and TRA
Endemic for SCH, STH and TRA

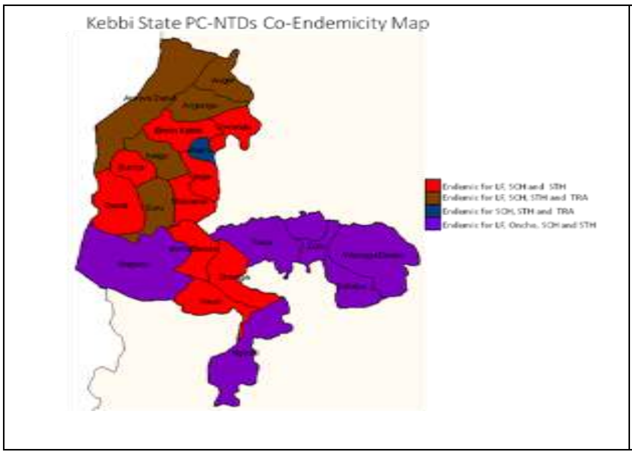
Kebbi State PC-NTDs Co-Endemicity Map

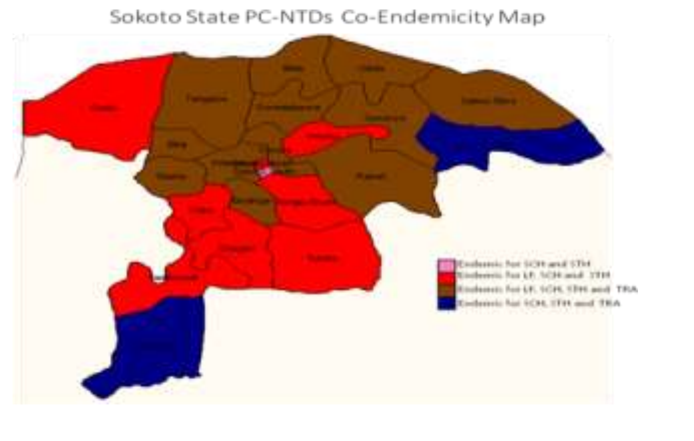
Sokoto State PC-NTDs Co-Endemicity Map

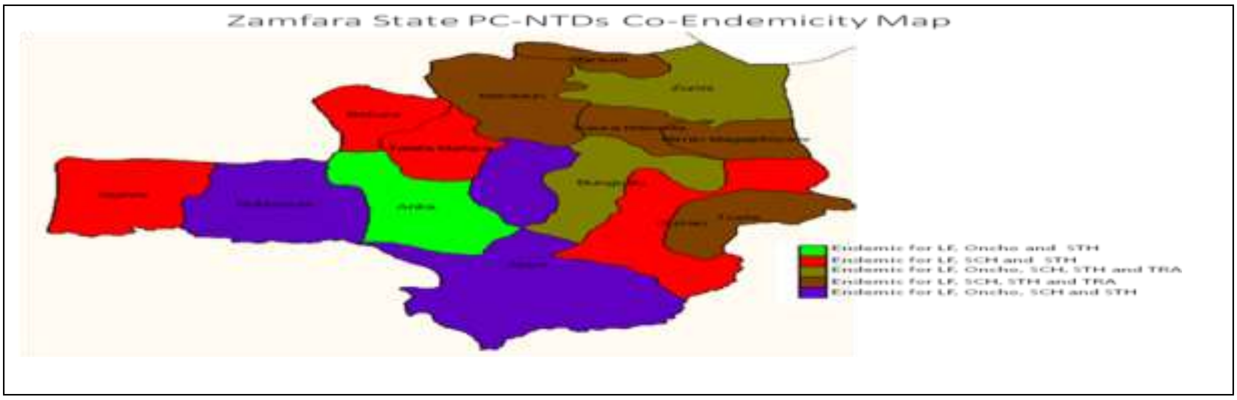
Zamfara State PC-NTDs Co-Endemicity Map
Endemic for LF, Oncho and STH
Endemic for LF, SCH and STH
Endemic for LF, Oncho, SCH, STH and TRA
Endemic for LF, SCH, STH and TRA
Endemic for LF, Oncho, SCH and STH

Table 3: NTDs mapping status

| Endemic NTD | Total No. of LGAs | No. of endemic LGAs | No. of LGAs mapped or known endemicity | No. of LGAs remaining to be mapped or assessed |
|---|---|---|---|---|
| Schistosomiasis | 774 | 603 | 708 | 66 |
| STH | 774 | 456 | 708 | 66 |
| Onchocerciasis | 774 | 0 | 774 | 0 |
| Lymphatic filariasis | 774 | 574 | 761 | 13 |
| Trachoma | 774 | 415 | 120 | 44 |
| HAT | 774 | Not mapped | Not mapped | Not mapped |
| Guinea worm | 774 | 0 | 774 | 0 |
| Rabies | 774 | 774 | 0 | 0 |
| Leishmaniasis | 774 | 3 | 7 | 767 |
| Yaws | 774 | Not mapped | Not mapped | Not mapped |
| Dengue | 774 | Not mapped | Not mapped | Not mapped |
| Leprosy | 774 | Not mapped | Not mapped | Not mapped |
| Buruli Ulcer | 774 | Not mapped | Not mapped | Not mapped |
| Mycetoma | 774 | Not mapped | Not mapped | Not mapped |

Based on information reflected above all the PC-NTDs have been mapped except in Adamawa, Yobe, Borno and Ekiti States. However, only leprosy status is known and there is the need to properly determine the status of leishmaniasis, buruli ulcer, yaws, dengue, mycetoma, and HAT in the country.

### 1.3.2 NTD Programme Implementation

#### *1.3.2.1 Onchocerciasis:*

The National Onchocerciasis Control Programme (NOCP) was established in 1987 with the mandate to reduce prevalence to levels to which it will no longer constitute a public health problem. In 1991, main chemotherapy of communities with Ivermectin (mectizan) was commenced. In 1997, the Community Directed Treatment with Ivermectin (CDTI) strategy was adopted as the main strategy of programme implementation. Over 22,000 health workers and 150,000 Community Directed Distributors (CDDs) have been trained or retrained annually in the past 5 years. 28 of the 32 States and FCT have reached the minimum standard of therapeutic coverage (65% and above). Average treatment coverage during the last 7 years has been about 26 million (78%) annually. 30,454,792 (78%) people in 36,373 (99.3%) communities were treated in 2012. Many hard-to-reach communities are being covered.

The CDTI has provided entry points for other community-based interventions such as Vitamin A supplementation, Guinea worm disease and Measles surveillance, elimination of Lymphatic Filariasis and Control of Schistosomiasis. Impact studies have shown improvement in Ophthalmological, Dermatological and Entomological indicators of treated populations.

Recently there was a shift from control to elimination of transmission necessitating coverage of hypo endemic areas that have the potential of sustaining transmission. This has raised the total at-risk population from about 40 million to 50 million persons.

#### *1.3.2.2 Lymphatic filariasis*

The National Lymphatic Filariasis Elimination Programme (NLFEP) was established in 1997 in response to World Health Assembly Resolution (May, 1997) urging member States to eliminate Lymphatic Filariasis (LF) as a Public Health problem. NLFEP was merged with NOCP in 2007 in order to harmonise implementation of MDA in co- endemic areas. Baseline survey has been done in 107 (95%) of the 113 Sentinel Sites (SS). Transmission Assessment Survey (TAS) is ongoing in 2 of the 36 States in the

country. A total of 558 LGAs are targeted for Mass Drug Administration (MDA) with free donated Ivermectin and Albendazole tablets in 35 States and FCT. As at 2013 MDA is ongoing in 179 LGAs of 18 States and FCT. Treatment has been discontinued in Plateau and Nassarawa States after the TAS 1 results indicated interruption of transmission.

Capacity is being developed for lymphoedema and hydrocele case management with relevant partners/stakeholders.

#### *1.3.2.3 Schistosomiasis:*

The National Schistosomiasis Control Programme was initiated in 1988 and the goal of the programme is to deliver regular anti-helminthic treatment to at least 75% of school-age children in endemic areas in the country in line with WHO recommendation.

The National Programme for Schistosomiasis Control is benefiting from Praziquantel tablets donation from Merck KGAe Germany since 2009. Targeted treatment with Praziquantel tablets is on-going in FCT and 26 States (Abia, Anambra, Delta, Edo, Enugu, Ebonyi, Imo, Cross River, Ekiti, Gombe, Katsina, Kwara, Nassarawa, Ogun, Ondo, Sokoto, Taraba, Zamfara, Plateau Bauchi, Jigawa, Kano, Kebbi, Kogi, Niger and Yobe) and FCT. A total of about 5.5 million persons (29%) were reached with praziquantel tablets in 2013 out of about 19,691,984 persons targeted from the 218 LGAs in States where epidemiological mapping has been conducted. There are plans to scale up from 218 to 302 LGAs in 2015.

#### *1.3.2.4 Soil Transmitted Helminths:*

The Soil Transmitted Helminths (STH) control programme was initiated in 2007. In line with WHO recommendation, the programme has set a target of regular administration of anti-helminthic drugs to at least 75% of school-age children in endemic areas in the country at risk of morbidity.

The National Programme is benefiting from the Johnson and Johnson Mebendazole tablets donation. Targeted treatment with Mebendazole tablets is on-going in FCT and Twenty four States (Delta, Edo, Enugu, Ebonyi, Imo, Cross River, Ekiti, Gombe, Katsina, Kwara, Nassarawa, Ogun, Ondo, Sokoto, Taraba, Zamfara, Plateau Bauchi, Jigawa, Kano, Kebbi, Kogi, Niger and Yobe) and FCT. A total of about 5.4 million school age children (25.4%) were treated with mebendazole tablets out of 21,232,039 targeted in 2013 by the National Programme. This figure is a gross under estimate as the STH Programme is equally benefiting from the Lymphatic filariasis MDA. There are plans to scale up from 217 LGAs to 456 LGAs in 2015.

Some other partners have been implementing school based de-worming programmes using Mebendazole tablets in the country. The data from such campaigns are not fed into the National Programme data base. The National Programme is making efforts to strengthen linkages between the various stake holders so as to have data from other sources fed into the National data base.

#### *1.3.2.5 Trachoma*

The WHO has recommended the SAFE Strategy for the elimination of trachoma as a public health problem.
**S** – Surgery; **A -** Antibiotic use; **F -** Face cleanliness; **E -** Environmental improvements.
*Surgery:* To address the backlog of Trichiasis. Facility and camp based surgeries are conducted. Details and implementation of these surgeries and their outcomes are contained in the Trachoma Action Plan.
*Antibiotics Distribution:* Antibiotic therapy to reduce the community reservoir of infection and therefore stop transmission. Distribution of antibiotics is done from house to house and is community directed under the supervision of the health workers.

*Facial cleanliness:* Facial cleanliness and improved hygiene to reduce transmission. Public health education for prevention of disease is carried out in schools and community centers. People are told about face washing, personal hygiene, water supply, latrines and the need to keep animals separate from living rooms. This is done using IEC materials.
*Environmental Improvement:*Environmental improvements, particularly water and sanitation, to make living conditions better so that the environment no longer facilitates the maintenance and transmission of trachoma. The NTCP is working in conjunction with the NGOs to support local communities to build latrines. The household and school latrines are promoted. The processes of latrine promotion start with the selection and training of masons and provision of cement by the supporting partners. The community supply materials and labour on their part. The mid-term achievement of implementing the SAFE strategy has led to increased uptake of services.
Trachoma control activities began in Nigeria in 2003. MDA started in 2010 in 10 LGAs in 5 States viz:. Kebbi (1LGA), Sokoto (1LGA), Zamfara (1LGA), Nassarawa (4LGAs) and Plateau (3LGAs). Mass administration of medicine using Zithromax is currently on-going in 32 LGAs in 5 States. The States are Jigawa (8 LGAs), Kebbi (2 LGAs), Sokoto (13 LGAs), Yobe (5 LGAs) and Zamfara (3 LGAs).

#### *1.3.2.6 Human African Trypanosomiasis:*
There is an on-going active hospital based surveillance and case management in three sentinel sites for HAT control and management as follows: Obiaruku General Hospital – zero case out of 43 persons tested using ICT method; Eku Baptist Medical Centre – 4 positive cases out of 8 persons tested; Kwale General Hospital – 1 positive case out of 72 persons tested. All the postive cases are being managed.

#### *1.3.2.7 Guinea Worm Disease:*
Nigeria has maintained a zero case status for Guinea worm disease (GWD) since 2009. Nationwide integrated GWD surveillance activities especially at the borders are being maintained. In 2013, Nigeria was certified Guinea worm disease (GWD) free by the World Health Organization. However, nationwide awareness creation for GWD, surveillance and the cash reward are still on till the disease is globally eliminated.

#### *1.3.2.8 Leprosy*
Nigeria has a combined TB and Leprosy programme (NTBLCP) whose implementation was launched in 1989 but became fully operational in1991. Since then, a total of 111,788 leprosy patients have been successfully treated with Multi-Drug Therapy (MDT) with 100% patient coverage achieved in 1995. The implementation of MDT as the strategic intervention for Leprosy elimination resulted in a rapid decline of the number of registered leprosy cases. In 1998, the country achieved the WHO elimination target of less than 1 case per 10,000 populations.

NTBLCP is saddled with formulating policies on the control of the two diseases. It incorporated Buruli ulcer in 2005. The implementation of these policies is done at the State and LGA levels by various STBLCPs. Each of the 36 States and FCT has a State TBL Control Programme Manager also known as the State TBL Control Officer (STBLCO). The STBLCO is the head of the State team and is responsible for programme management and technical guidance to the LGAs. The LGA is the operational level of the programme. The LGA TBL supervisor is responsible for programme management at LGA level. Leprosy and TB services have been integrated into the general health care services.

#### *1.3.2.9 Buruli ulcer:*
Buruli ulcer control programme is still in the emerging phase. It was created and merged with Tuberculosis and leprosy control programme in 2005. The overall goal of BU control programme is to reduce the morbidity, disabilities and socio-economic consequences caused by the disease. However, this control programme has not been vibrant in Nigeria due to dearth of resources. Nevertheless, the programme has conducted a number of advocacy and IEC campaignsas well as sensitization of health workers in selected States.

Sensitization training for GHWs was done in Akwa Ibom, Benue, and Ebonyi in 2011.German Leprosy Relief Association (GLRA) piloted BU case detection and management project in Cross River State and Ogun State. It has scaled-up the pilot project to Anambra State in 2014. Streptomycin and Rifampicin, donated by WHO, have been distributed to Ogun, Anambra, Ebonyi, Cross River, Akwa-Ibom and Kwara States.

#### *1.3.2.10 Leishmaniasis:*

.

This is still at an emergent phase. No control effort has been initiated in the country.

#### *1.3.2.11 Zoonotic and other NTDs:*

The zoonotic NTDs component of the NTD division has developed an approved a plan for human anti-rabies vaccine (H-ARV) and the human rabies Immunoglobulin (HR-IG) distribution. Following the establishment of inter-departmental collaboration in 2007, the zoonotic NTD programme is collaborating with the department of veterinary services in the Federal Ministry of Agriculture on the control and elimination of rabies in Nigeria.

Interventions for the other case management NTDs are yet to be initiated.

Table 4.1: Summary of intervention information on existing preventive chemotherapy programmes (as at 2013)

| NTD | Date programme started | Total No. of LGAs targeted | No. of LGAs covered *(Geographic coverage) | Total population in target LGAs | No. of (percentage) Population Covered | Key strategies used | Key partners |
|---|---|---|---|---|---|---|---|
| **SCH** | 2009 | 661 | 199 (30%) | 43,544,818 | 5,500,000 (13)% | MDA, Health promotion and hygiene education, Provision of portable water and environmental and integrated vector management | WHO, De Worm The World/PCD, NGDOs, Merck, J&J, SS, TCC, HKI,HANDS,MITOSATH, |
| **STH** | 2010 | 482 | 200 (41%) | 44,064,527 | 5,400,000* (12)% | MDA, Health promotion and hygiene education, Provision of portable water and environmental and integrated vector management | WHO, De Worm The World/PCD, NGDOs, Merck, J&J, SS, TCC, HKI,HANDS,MITOSATH, |
| **Oncho** | 1992 | 473 | 430 (91%) | 42,966,736 | 33,482,258 (78%) | MDA (IVM) | APOC, NGDO Coalition in Nigeria |
| **LF** | 1997 | 557 | 179 (32%) | 118,306,096 | 22,735,296 (19%) | IVM+ALB | WHO,MDP,GSK, UNICEF, CNTD, RTI/Envision, TCC,SS,CBM/ |

| | | | | | | | |
|---|---|---|---|---|---|---|---|
| | | | | | | | HANDS, MITOSATH, HKI, Sightcare |
| **Tracho ma** | 2010 | 69 | 51 (74%) | 10,010,68 9 | 5,804,181 (58%) | MDA | Sightsavers, TCF, HKI, CBM, HANDS |

1.3

Table 4.2: Summary information on existing case management programmes

| NTD | Date programe or intervention started | Total No. of LGAs targeted | No. of LGAs covered *(Geographic coverage) | Key strategies used | Key partners |
|---|---|---|---|---|---|
| Schistosomiasis | 1988/2009 | 500 | 49 (10%) | Active case detection and facility management | Sightsavers, TCC, WHO, MITOSATH |
| Lymphatic filariasis | 2000 | 774 | 153 (20%) | -lymphoedema management through simply hygiene of washing affected part at least twice daily with clean water and soap<br>-Hydrocelectomy and use of anti-bacterial and anti-fungal creams for secondary infection, hydrocele surgery | WHO,MDP,GSK, APOC,TCC,SS, CBM/HANDS, MITOSATH, Sightcare |
| HAT | 2006 | 200 | 21 (10%) | Active case detection and facility management | WHO, FIND, AU-PATTEC, NITR, Federal Ministry of Science |
| Guinea worm | 1988 | 774 | 774 (100%) | Surveillance | WHO, YGC, TCC |
| Rabies | 1988 | 774 | 0 | Active case detection and facility management Administration of H-ARV and HR-AG | |
| Leishmaniasis | Not started | 774 | 30 | Active case detection and facility management Administration of topicals and infusions | |
| Leprosy | 1989 | 250 | 150 (60%) | Active case detection and facility management | ILEP, WHO |
| Buruli Ulcer | 2008 | 60 | 5 (8%) | Active case detection and facility management | ILEP, WHO |
| Trachoma | 2001 | 156 | 10 (2%) | Triachiasis surgery, tetracycline, Azithromycin | Sightsavers, TCC, WHO, MITOSATH, Merck |

*Geographical Coverage = No. of LGAs covered by the programme /Total No. of endemic LGAs in the country

### 1.3.3 SWOT Analysis

Table 5.1: The SWOT Analysis

| STRENGTHS | WEAKNESSES | OPPORTUNITIES | THREATS |
|---|---|---|---|
| | **Planning and Coordination** | | |
| Established focal NTD points in all States except Bayelsa State | Inadequate infrastructure and equipment at National and Zonal offices | Availability of a newly endorsed National Health Act that can support NTDs programme | Frequent transfer of NTD staff |
| Presence of a strong NTD advisory body – The NTD Steering Committee | Unavailability of proper NTD structures in 4 non-CDI States (Rivers, Bayelsa, Katsina and Lagos) | Presence of a strong legislative arm of government to make policies in favour of the NTDs programme | Bureaucratic bottle necks that slow planning and implementation processes |
| Availability of revised national multi-year strategic plan, NTD policy and disease-specific guidelines | Absence of State technical advisory committee in most States (The State NTD Task Force) | Availability of a national post 2015 Sustainable Development Goal | Conflicting priority by government at the detriment of NTDs programme |
| Availability of State specific NTD strategic and annual plans for 36 States and FCT | Continued vertical programme planning and intervention at the State and National levels | | |
| Involvement of relevant line ministries in annual review and stakeholders meetings | Inadequate NGDO partners coordination | | |
| Availability of clear strategies, protocols, and guidelines for successful programme delivery | Lack of sustainability plans for NTDs programme | | |
| Availability of various integrated NTDs teams at the national level to carry out specific programme assignments | Absence of regular meetings with policy makers at the national level | | |

| | | | |
|---|---|---|---|
| **Advocacy and Communication** | | | |
| The current use of media messages for promotion of some NTDs elimination and eradication | Lack of advocacy champions for integrated NTDs programme in Nigeria | Availability of experienced past leaders that can serve as NTD champions | Poor communication network in rural communities |
| Availability of advocacy personnel (zonal coordinators and national program managers) and tools to solicit for required financial and administrative support | Lack of comprehensive integrated NTDs advocacy kits and other IEC materials | | Apathy of the public towards the NTD programme |
| Routine advocacy visits conducted by zonal coordinators and national programme managers | Inadequate media publicity of some NTDs | | |
| Availability of a resource mobilization team at the national level to develop NTD resource mobilization guide | Weak resource mobilization team at the national level to perform its duties | | |
| **Scale-up interventions for PCT and Service Delivery Capacity** | | | |
| PCT NTDs mapping conducted in more than 80% of LGAs | Non-completion of PCT NTDs mapping in a few LGAs in the country | Presence of community based organizations (CBOs) in many communities and LGAs | Frequent transfer of NTD staff at State and LGA levels |
| Availability of donated drugs for NTDs | Lack of adequate support staff in Zonal offices | Presence of other community based health programmes with huge financial support in communities | Inadequate number of health workers and staff at the LGA and State level |
| The presence of CDI structures in majority of the LGAs for scale-up interventions | Inadequate number of staff in some NTD programmes at State level | Increasing global commitment for NTDs elimination and eradication goals by 2020 | High attrition of community implementers |
| Presence of Community Implementers in majority of communities | Non retrieval of left over PCT drugs at the community, health facility and LGA level | Presence of strong security organizations such as JTF, local vigilante groups and international agencies like Red cross | Misconception and negative rumors about the NTD programme |
| Availability of trained Health workers in most Health Facilities and LGAs | PC drug wastage in some communities/LGAs | Availability of physical structures that serve as health facilities across the country | Severe Adverse Events from MAM |
| Availability of trained NTD teams at the State level | Late commencement of interventions activities due to late arrival of some PC drugs | | Security challenges in some parts of the country |

| | | | |
|---|---|---|---|
| Availability of integrated NTD training manuals at all levels of implementation | Low commitment of health workers and community implementers | | Natural disasters |
| Annual training and retraining of NTD personnel at LGA and community levels | Diminishing community support in many communities endemic for NTDs | | |
| | Poor preparedness of health facilities and communities (including schools) on the management of SAEs | | |
| | Poor treatment coverage rates for MAM in urban treatments | | |
| | Inadequate number of trained school teachers and community implementers for increased programme coverage | | |
| **Case management of NTDs** | | | |
| Existence of some collaboration with the Leprosy programme (ILEP group) | Lack of baseline data on most case management diseases | Monitoring and supervision of PC NTDs activities to collect information | Competition for scarce government resources for performance |
| Successful eradication of a case management disease i.e Guinea Worm Disease in the country | Lack of support or focus for the elimination of case management NTDs | | |
| | Inadequate knowledge and awareness of case management programmes | | |
| **Support, Financing and partnerships** | | | |
| Strong collaboration and partnership with NGDOs | Inadequate government commitment to release of counterpart funds | Increasing global commitment for NTDs elimination and eradication goals by 2020 | Competing government priorities |
| Presence of a strong and closely knit NGDO coalition group for NTDs in Nigeria | Current partners not working in all parts of the country e.g. Bayelsa and Rivers States | Collaboration with the malaria programme | Old donors fatigue |
| Availability of NTDs programme budget | Weak involvement of line | Availability of a community based | Bureaucratic bottle necks to |

| | | | |
|---|---|---|---|
| line at the national level and some States | ministries in NTD activities. Inadequate inter-sectoral collaboration with line ministries and agencies | unit under the department of public health in the FMOH | delay progress in planning and implementation |
| Establishment of some collaboration between the NTDs and Malaria programme | Lack of a NTD resource mobilization guide | Interest of new partners in the NTDs programme | Over-dependence on donor support |
| Some collaboration with line ministries and agencies e.g Education and WASH | Lack of cross- border collaboration with other NTDs-endemic countries bordering Nigeria | Availability of multi-national organization funding for Health Sector like HIV, TB & Malaria | |
| Partners continuous financial and technical support | | Transitioning government with new and improved agenda | |
| Government support to the provision of human resources for NTDs and their remuneration | | Presence of JTF, local vigilante groups and international agencies like Red cross | |
| **Data Management, Monitoring and Evaluation, Operational research** | | | |
| Availability of integrated reporting MIS tools and guidelines | Inadequate knowledge of the data collection tools at sub-national levels | Creation of M-Health for data management | Loss of historical data through major events |
| Established integrated NTDs data management system | Poor monitoring and supervision due to inadequate funds and logistics | Interest of partners to build a strong monitoring and evaluation system for NTDs programme | Insecurity from communal clashes during monitoring and supervisory activities |
| HMIS, IDSR and NDHS captures some NTDs indicators | Inadequate number of operational vehicles | Existing in-country NTD experts on NTDs monitoring and evaluation | |
| Availability of tools of planning and costing programme activities (TIPAC) | Numerous health programme forms to be completed by the health worker at the health facility level | | |
| Availability of integrated forms for case search of case management diseases | Weak capacity of staff on monitoring and supervision at State and LGA levels | | |
| Existing community monitoring structures for some programmes | Late submission of treatment reports from community to the State level | | |

| | | | |
|---|---|---|---|
| Conduct of evaluations for some project to determine their elimination status | Inconsistent data reporting and poor drug inventory | | |
| | Non availability of State based integrated NTD data management system and trained personnel | | |
| | Inadequate capacity for utilization of TIPAC at sub-national levels. | | |
| | Absence of functional community based surveillance system | | |
| | Non conduct of regular operational researches to address programme challenges | | |
| | Cessation of evaluation activities of some projects due to lack of funds to conduct them | | |

Table 5.2: SWOT Counteracting Table

| **Weakness** | **Strengths counteracting weaknesses** | **Opportunities counteracting Weaknesses** |
|---|---|---|
| Inadequate infrastructure and equipment at National and Zonal offices | Established focal NTD points in all States except Bayelsa State | |
| Non availability of proper NTD structures in 4 non-CDI States (Rivers, Bayelsa, Katsina and Lagos) | Availability of revised national multi-year strategic plan, NTD policy and disease-specific guidelines<br>The presence of CDI structures in majority of the LGAs for scale-up interventions | |
| Absence of State technical advisory committee in most States (The State NTD Task Force) | Presence of a strong NTD advisory body – The NTD Steering Committee<br>Availability of State specific NTD strategic and annual plans for 36 States and FCT | |
| Continued vertical programme planning and intervention at the State and National levels | Availability of revised national multi-year strategic plan, NTD policy and disease-specific guidelines<br>Availability of clear strategies, protocols, and guidelines for successful programme delivery<br>Availability of integrated NTD training manuals at all levels of implementation | |

| | | |
|---|---|---|
| Inadequate NGDO partners coordination | Presence of a strong and closely knit NGDO coalition group for NTDs in Nigeria | |
| Lack of sustainability plans for NTDs programme | | Availability of a national post 2015 Sustainable Development Goal |
| Absence of regular meetings with policy makers at the national level | | |
| Lack of advocacy champions for integrated NTDs programme in Nigeria | Availability of advocacy personnel (zonal coordinators and national program managers) and tools to solicit for required financial and administrative support<br>Routine advocacy visits conducted by zonal coordinators and national programme managers<br>The current use of media messages for promotion of some NTDs elimination and eradication | Availability of experienced past leaders that can serve as NTD champions |
| Lack of comprehensive integrated NTDs advocacy kits and other IEC materials | Availability of integrated NTD training manuals at all levels of implementation | |
| Inadequate media publicity of some NTDs | | Presence of community based organizations (CBOs) in many communities and LGAs |
| Weak resource mobilization team at the national level to perform its duties | Availability of a resource mobilization team at the national level to develop NTD resource mobilization guide | Increasing global commitment for NTDs elimination and eradication goals by 2020 |
| Non-completion of PCT NTDs mapping in a few LGAs in the country | PCT NTDs mapping conducted in more than 80% of LGAs | |
| Lack of adequate support staff in Zonal offices | Availability of various integrated NTDs teams at the national level to carry out specific programme assignments | |
| Inadequate number of staff in some NTD programmes at State level | Availability of trained NTD teams at the State level | |
| Non retrieval of left over PCT drugs at the community, health facility and LGA level | | |

| | | |
|---|---|---|
| PC drug wastage in some communities/LGAs | | |
| Late commencement of interventions activities due to late arrival of some PC drugs | | |
| Low commitment of health workers and community implementers | Availability of trained Health workers in most Health Facilities and LGAs | Presence of community based organizations (CBOs) in many communities and LGAs |
| Diminishing community support in many communities endemic for NTDs | Presence of Community Implementers in majority of communities | Presence of community based organizations (CBOs) in many communities and LGAs<br>Presence of other community based health programmes with huge financial support in communities<br>Availability of a community based unit under the department of public health in the FMOH |
| Poor preparedness of health facilities and communities (including schools) on the management of SAEs | | Availability of physical structures that serve as health facilities across the country |
| Poor treatment coverage rates for MAM in urban treatments | The presence of CDI structures in majority of the LGAs for scale-up interventions | Collaboration with the malaria programme |
| Inadequate number of trained school teachers and community implementers for increased programme coverage | Presence of Community Implementers in majority of communities | |
| Lack of baseline data on most case management diseases | | |
| Lack of support or focus for the elimination of case management NTDs | Existence of some collaboration with the Leprosy programme (ILEP group)<br>Successful eradication of a case management disease i.e Guinea Worm Disease in the country<br>Availability of integrated forms for case search of case management diseases | |
| Inadequate knowledge and awareness of case management programmes | | |
| Inadequate government commitment to release of counterpart funds at all levels | Strong collaboration and partnership with NGDOs<br>Availability of NTDs programme budget line at the national level and some States<br>Partners continuous financial and technical support<br>Government support to the provision of human | Increasing global commitment for NTDs elimination and eradication goals by 2020 |

| | | |
|---|---|---|
| | resources for NTDs and their remuneration | |
| Current partners not working in all parts of the country e.g. Bayelsa and Rivers States | Presence of a strong and closely knit NGDO coalition group for NTDs in Nigeria | Transitioning government with new and improved agenda |
| Weak involvement of line ministries in NTD activities.<br>Inadequate inter-sectoral collaboration with line ministries and agencies | Some collaboration with line ministries and agencies e.g Education and WASH<br>Establishment of some collaboration between the NTDs and Malaria programme<br>Involvement of relevant line ministries in annual review and stakeholders meetings | |
| Lack of a NTD resource mobilization guide | Availability of a resource mobilization team at the national level | |
| Lack of cross- border collaboration with other NTDs-endemic countries bordering Nigeria | | Transitioning government with new and improved agenda |
| Inadequate knowledge of the data collection tools at sub-national levels | Availability of integrated reporting MIS tools and guidelines<br>Availability of tools of planning and costing programme activities (TIPAC) | |
| Poor monitoring and supervision due to inadequate funds and logistics | | Existing in-country NTD experts on NTDs monitoring and evaluation |
| Inadequate number of operational vehicles | | |
| Numerous health programme forms to be completed by the health worker at the health facility level | | |
| Weak capacity of staff on monitoring and supervision at State and LGA levels | Availability of integrated NTD training manuals at all levels of implementation | Existing in-country NTD experts on NTDs monitoring and evaluation |
| Late submission of treatment reports from community to the State level | Established integrated NTDs data management system | Interest of partners to build a strong monitoring and evaluation system for NTDs programme<br>Creation of M-Health for data management |
| Inconsistent data reporting and poor drug inventory | Established integrated NTDs data management system<br>Availability of integrated reporting MIS tools and guidelines<br>Availability of integrated NTD training manuals at all levels of implementation | Existing in-country NTD experts on NTDs monitoring and evaluation |
| Non availability of State based integrated NTD data management system and trained personnel | Availability of integrated reporting MIS tools and guidelines | |

| | | |
|---|---|---|
| | Established integrated NTDs data management system | |
| Inadequate capacity for utilization of TIPAC at sub-national levels | Availability of tools of planning and costing programme activities (TIPAC) | |
| Absence of functional community based surveillance system | Existing community monitoring structures for some programmes | |
| Non conduct of regular operational researches to address programme challenges | | |
| Cessation of evaluation activities of some projects due to lack of funds to conduct them | Conduct of evaluations for some project to determine their elimination status | |
| **Threats** | **Strength Counteracting Threats** | **Opportunities Counteracting Threats** |
| Frequent transfer of NTD staff | Annual training and retraining of NTD personnel at LGA and community levels | Availability of a newly endorsed National Health Act that can support NTDs programme |
| Bureaucratic bottle necks that slow planning and implementation processes | | Presence of a strong legislative arm of government to make policies in favour of the NTDs programme |
| Conflicting priority by government at the detriment of NTDs programme | Involvement of relevant line ministries in annual review and stakeholders meetings<br>Availability of advocacy personnel (zonal coordinators and national program managers) and tools to solicit for required financial and administrative support | Availability of experienced past leaders that can serve as NTD advocacy champions |
| Poor communication network in rural communities | Availability of clear strategies, protocols, and guidelines for successful programme delivery | Presence of community based organizations (CBOs) in many communities and LGAs |
| Apathy of the public towards the NTD programme | The current use of media messages for promotion of some NTDs elimination and eradication | Presence of community based organizations (CBOs) in many communities and LGAs<br>Presence of other community based health programmes with huge financial support in communities |
| Inadequate number of health workers and staff at the LGA and State level | Availability of trained Health workers in most Health Facilities and LGAs | |

| | | |
|---|---|---|
| High attrition of community implementers | | Presence of community based organizations (CBOs) in many communities and LGAs<br>Availability of a community based unit under the department of public health in the FMOH |
| Misconception and negative rumors about the NTD programme | | |
| Severe Adverse Events from MAM | Availability of integrated NTD training manuals at all levels of implementation | |
| Security challenges in some parts of the country | Presence of Community Implementers in majority of communities | Presence of strong security organizations such as JTF, local vigilante groups and international agencies like Red cross |
| Natural disasters | | |
| Competition for scarce government resources for performance | Establishment of some collaboration between the NTDs and Malaria programme<br>Some collaboration with line ministries and agencies e.g Education and WASH | Interest of new partners in the NTDs programme<br>Availability of multi-national organization funding for Health Sector like HIV, TB & Malaria |
| Competing government priorities | | Transitioning government with new and improved agenda |
| Old donors fatigue | Availability of continuous donated drugs for NTDs<br>Strong collaboration and partnership with NGDOs<br>Presence of a strong and closely knit NGDO coalition group for NTDs in Nigeria<br>Partners continuous financial and technical support | Increasing global commitment for NTDs elimination and eradication goals by 2020 |
| Over-dependence on donor support | | |
| Loss of historical data through major events | Availability of integrated reporting MIS tools and guidelines<br>Established integrated NTDs data management system<br>HMIS, IDSR and NDHS captures some NTDs indicators | Creation of M-Health for data management<br>Interest of partners to build a strong monitoring and evaluation system for NTDs programme<br>Existing in-country NTD experts on NTDs monitoring and evaluation |

### 1.3.4 Gaps and Priorities

**Gaps**

From the SWOT counteracting table, major gaps that are current and anticipated which need to be addressed in order for the NTD programme to achieve its 2020 elimination goals include the following:

**Planning and Coordination**

- Non availability of full NTD structures in 4 non-CDI States namely: Rivers, Bayelsa, Katsina and Lagos
- Lack of sustainability plans for NTDs programme post 2020

**Advocacy and communication**

- Poor awareness of the NTD programme among policy makers and community members caused by inadequate media publicity of some NTDs.
- Lack of comprehensive integrated NTDs advocacy kits and other IEC materials

**Scale-up interventions for PCT, Service Delivery Capacity and Case Management of NTDs**

- Non-completion of PCT NTDs mapping in security challenged areas in Borno, Adamawa, Yobe and Ekiti States
- Weak capacity and inadequate number of the FMOH Zonal staff to effectively carryout the supervision and monitoring of LGA and community level activities
- High attrition of community implementers
- PC medicine wastage and lack of a well monitored retrieval system of left-over medicines.
- Low commitment of health workers and community implementers
- Diminishing community support in many communities endemic for NTDs
- Poor preparedness of health facilities, communities and schools on the management of SAEs
- Poor treatment coverage rates for MAM in urban areas
- Inadequate number of trained school teachers and community implementers for increased programme coverage
- Low profile of case management NTDs due to lack of attention to them by government and partners

**Support, Financing and Partnerships**

- Inadequate government commitment to release of counterpart funds at all levels
- Absence of partners to support the NTD programme in some States
- Weak involvement of line ministries in the elimination activities of NTD activities

**Data Management, Monitoring and Evaluation, Operational Research**

- Poor monitoring and supervision due to inadequate funds and weak capacity of staff at the State and LGA levels
- Cessation of evaluation activities of some projects due to lack of funds to conduct them
- Late submission of treatment reports from community to the State level
- Inconsistent data reporting and poor data management
- Non conduct of regular operational researches to address programme challenges
- Misconception and negative rumors about the NTD programme may hinder the elimination progress of NTDs
- Severe Adverse Events from MAM
- Loss of historical data through major events

**Priorities**

In order to address the gaps, the table below identifies programme's priorities for the strategic period of elimination goals

Table 5.3: Gaps and Priorities

| Gaps | Priorities |
|---|---|
| Non availability of full NTD structures in 4 non-CDI States namely: Rivers, Bayelsa, Katsina and Lagos<br><br>Lack of sustainability plans for NTDs programme post 2020 | Creation of NTD structures in Rivers, Bayelsa, Katsina and Lagos States and strengthening of structures in other States<br>Development of sustainability plans for NTDs programme before 2020 |
| Poor awareness of the NTD programme among policy makers and community members caused by inadequate media publicity of some NTDs.<br>Lack of comprehensive integrated NTDs advocacy kits and other IEC materials | Increase the profile of NTDs in all communities, LGAs and States and among policy makers |
| Non-completion of PCT NTDs mapping in security challenged areas in Borno, Adamawa, Yobe and Ekiti States<br><br>Weak capacity and inadequate number of the FMOH Zonal staff to effectively carryout the supervision and monitoring of LGA and community level activities<br><br>High attrition of community implementers<br><br>PC medicine wastage and lack of a well monitored retrieval system of left-over medicines.<br><br>Low commitment of health workers and community implementers<br><br>Diminishing community support in many communities endemic for NTDs<br><br>Poor preparedness of health facilities, communities and schools on the management of SAEs<br><br>Poor treatment coverage rates for MAM in urban areas<br><br>Inadequate number of trained school teachers and community implementers for increased programme coverage<br><br>Low profile of case management NTDs due to lack of attention to them by government and partners | Completion of PCT NTDs mapping in Borno, Adamawa, Yobe and Ekiti States<br><br>Comprehensive capacity building of NTD staff and volunteers at all levels on integrated NTDs<br><br>Creation and strengthening of medicines supply chain management system to address wastage and eliminate medicine pilferage<br><br>Regular spot check visitations to health facilities and communities. Provision of incentives in form of usable IEC materials for health workers and CIs<br><br>Strengthening the capacity of the health facilities and school based clinics to manage adverse effects of the MAMs<br><br>Improve collaboration with all community based organizations within LGAs and communities to ensure full access and coverage of MAM<br><br>Conduct the case search and comprehensive data collection of all relevant case management diseases to have broad baseline information on case management in Nigeria |
| Inadequate government commitment to release of counterpart funds at all levels<br><br>Absence of partners to support the NTD programme in some States<br><br>Weak involvement of line ministries in the elimination activities of NTD activities | Intensify efforts through identified strategies to ensure that NTDs are one of the top priorities of the incoming government<br><br>Strengthen collaboration with line ministries related to the WASH sector and drug law enforcement agencies like NAFDAC |
| Poor monitoring and supervision due to inadequate funds and weak capacity of staff at the State and LGA levels<br><br>Cessation of evaluation activities of some projects due to lack of funds to conduct them<br><br>Late submission of treatment reports from community to the State level<br><br>Inconsistent data reporting and poor data management<br><br>Non conduct of regular operational research to address programme challenges<br><br>Loss of historical data through major events | Strengthen data management systems at all levels<br><br>Strengthen the monitoring and supervision system across all levels and ensure the timely reporting of NTDs data<br><br>Establish and strengthen a self-monitoring treatment system in all communities<br><br>Conduct identified operational research to improve programme performance |

## PART TWO: NTD STRATEGIC AGENDA

### 2.1. MISSION AND GOALS:

**Mission:**

To implement NTD policy and plan in order to deliver effective, efficient, quality and affordable health services to strengthen the national health system and achieve improved health status of Nigerians for accelerated national economic growth and sustainable development.

**Vision:**

To have a country free of Neglected Tropical Diseases.

**Strategic Goal:**

To reduce morbidity, disability and mortality via the control, elimination and eradication of targeted NTDs and contribute to poverty alleviation, increased productivity and better quality of life of the affected people in Nigeria.

**Programme focus**

To progressively reduce morbidity, disability and mortality due to NTDs using integrated and cost-effective approaches with the view to eliminating NTDs in Nigeria by the year 2020.

**Strategic Milestones:**

Quarterly monitoring and evaluation of input and output indicators and final evaluation of programme impact.

**Strategic Priorities:**

- Strengthen government ownership, advocacy, coordination and partnerships.
- Enhance planning for results, resource mobilization and financial sustainability of national NTD programmes for NTD management, control and elimination..
- Scale-up access to interventions, treatment and system capacity building.
- Enhance NTD monitoring and evaluation, surveillance and operational research

Table 6: Strategic Objectives

| **Strategic Priorities** | **Strategic Objectives** |
|---|---|
| Scale-Up Access to Interventions, Treatment & Service Delivery Capacities | Scale up integrated preventive chemotherapy packages |
| | Scale up integrated case-management-based diseases interventions, especially for leprosy, Guinea worm disease, HAT, Buruli Ulcer and endemic Loasis, Leishmaniasis and human rabies prevention |
| | Strengthen integrated vector management and activities for health education, access to clean water, sanitation, and environmental improvement for targeted NTDs |
| | Strengthen capacity for NTD programme management and implementation |
| Enhance Planning for Results, resource mobilization and financial sustainability of National NTDs Programme | Develop integrated multi-year strategic plan and annual operational plans |
| | Enhance resource mobilization approaches and strategies at national and sub-national levels |
| | Strengthen the integration and linkages of NTD programme and financial plans into sector-wide and national budgetary and financing mechanisms |
| | Update national NTD policies and elaborate guidelines and tools |
| Strengthen Government, Ownership, Coordination and Partnerships | Strengthen coordination mechanisms for the NTD control at national and sub-national levels |
| | Strengthen and foster partnerships for NTDs at all levels. |
| | Enhance NTD programme performance reviews for decision making |
| | Strengthen advocacy, visibility and profile of NTD control programmes |
| Enhance NTD Monitoring, Evaluation, Surveillance and Operations Research | Develop and promote an integrated NTD M&E framework and improve monitoring of NTDs, within the context of national health information systems |
| | Strengthen the surveillance of NTDs and response to epidemic-prone NTDs. |
| | Support operational research, documentation and evidence |
| | Improve integrated data management system and support impact analysis for NTD. |

Provided in the table below is the National NTD Disease specific goals and objectives.

Table 7: Summary of NTD disease specific goals and objectives

| PROGRAMME AND GLOBAL GOALS | NATIONAL GOAL | OBJECTIVES | STRATEGIES/ INTERVENTIONS | DELIVERY CHANNELS |
|---|---|---|---|---|
| 1. **Lymphatic Filariasis Elimination**<br><br>Goal:<br>To eliminate LF as public health problem by 2020 | To eliminate LF as a public health problem by 2020. | To reduce and interrupt transmission of LF by 2020.<br><br>To prevent and reduce disability in affected persons through disability alleviation and appropriate management | Annual mass drug administration with Ivermectin and Albendazole medicine to all at risk population<br>.<br>Vector control (LLIN).<br><br>Surveillance activity for areas that are undergoing Transmission Assessment Survey (TAS)<br><br>Lymphodema management.<br><br>Hydrocele surgery<br><br>BCC (Behavioural Change and Communication) | Use of CDI Structure |
| 2. **Onchocerciasis Elimination**<br><br>Goal:<br>To eliminate onchocerciasis with CDI and other effective interventions by 2025. | To eliminate onchocerciasis as a public health problem by 2020 | To achieve 100% geographical coverage and maintain 80% therapeutic coverage in the transmission zones<br><br>To eliminate transmission of onchocerciasis in 10 states by 2017. | Annual treatment with Ivermectin to the population at risk.<br><br>Focal ground larviciding in established isolated vector breeding sites.<br><br>Health education and promotion of behavioural change | Use of CDI Structure |
| 3. **Schistosomiasis** | To eliminate Schistosomiasis as | To complete mapping by 2015 | Mass Drug Administration in the | School Health programme, |

| | | | | |
|---|---|---|---|---|
| **Elimination**<br>Goal: Treat at least 75% of all school aged children and others at risk by 2020 | a public health problem by 2020 | To establish implementation structures in all 36 States and FCT<br><br>To achieve at least 75% therapeutic coverage in implementing LGAs by 2020 | school-aged children<br><br>Health education and promotion of behavioural change<br><br>Improvement in water supply and sanitation<br><br>Foci control of snail intermediate hosts | Community structures (CDI) for non-enrolled school children. |
| **4. Soil Transmitted Helminths Elimination**<br>Goal: Treat at least 75% of all school aged children and others at risk by 2020 | To eliminate Soil Transmitted Helminths as a public health problem by 2020 | To complete mapping by 2015<br><br>To establish implementation structures in all 36 States and FCT<br><br>To achieve at least 75% therapeutic coverage in implementing LGAs by 2020 | MDA with Albendazole/ Mebendazole in the school age population and high risk communities<br><br>Health education and promotion of behavioural change<br><br>Improvement in water supply and sanitation | School Health Programme, Community<br><br>structures (CDI) for non-enrolled schooled children |
| **5. Trachoma Elimination**<br>Goal: Eliminate as blinding disease by 2020. | To control blinding trachoma through the SAFE strategy by the year 2020 | To complete mapping of trachoma by 2015<br><br>To eliminate blinding trachoma through SAFE strategy by 2020 | Health education and promotion of behavioural change<br><br>Improved water supply for personal hygiene<br><br>Personal hygiene reinforcing face washing<br><br>Mass Drug Administration with Azithromycin of entire identified at risk communities<br><br>Surgery of trichiasis cases<br><br>Surveillance activity for areas that are undergoing impact assessment. | Use of CDI Structure |

| | | | | |
|---|---|---|---|---|
| 6. **Leprosy elimination**<br><br>Goal:<br>Early diagnosis and treatment with MDT, elimination of leprosy as a public health problem at national by 2015, and then elimination at sub-national levels | To reduce the prevalence and socio-economic burden associated with leprosy to such a level that it is no longer a public health problem | To ensure early case detection such as the rate of new cases with grade 2 disabilities per 100,000 population is reduced by at least 35% by end of 2015 compared to the baseline at the beginning of 2010<br><br>To provide comprehensive care such that <5% of patients develops disability while on treatment<br><br>To provide health education and counseling to ensure >95% and >85% treatment completion rates among PB and MB patients respectively<br><br>To ensure 100% MDT coverage for all patients in need of MDT<br><br>To ensure that all leprosy rehabilitation needs are mainstreamed into the existing socio-economic and physical rehabilitation services at the State and National levels | Early case finding and proper treatment<br><br>BCG Vaccination<br><br>Strengthen leprosy control activities in areas with high proportions of grade 2 disability and children, among new cases<br><br>Promote the use of community-based rehabilitation to improve the quality of life of persons and families affected by leprosy<br><br>Focused integration of leprosy control into the general care services, this will involve designated skin clinic days in designated facilities<br><br>Increased surveillance for resistant strains of M leprae<br><br>Apply cost-effective advocacy and communication methods to improve community awareness, acceptance and involvement to combat stigma and discrimination against persons and families affected by leprosy<br><br>Institute effective referral system from peripheral health facilities to designated skin clinics and referral centers<br><br>Household contact tracing | Hospital/ Facility - Based case management |

| | | | | |
|---|---|---|---|---|
| | | | Post MDT surveillance.,LT | |
| 7. **<u>Human African Trypanosomiasis Elimination</u>**<br><br>Goal: Eliminate as a public health problem by 2017 | Elimination of HAT from Nigeria by 2020. | To develop and implement systematic surveillance activities as well as integrated intervention packages for Trypanosomiasis prevention and control and ensure that HAT is eliminated from Nigeria by 2020. | 100% mapping for HAT in all target states<br><br>Surveillance and case reporting.<br><br>Health promotion and Education.<br><br>Case detection and management protocols.<br><br>Integration of HAT surveillance and notification into IDSR.<br><br>Training and re-training of HAT focal persons and medical personnel on case detection and management<br><br>Establishing and maintaining sentinel site | Hospital/ Facility - Based case management |
| 8. **<u>Buruli Ulcer Control</u>**<br><br>*Goal:* Early detection and early treatment for effective control; increasing surveillance and control | To reduce the morbidity, disabilities and socio-economic consequences caused by the disease by 2020 | To increase case detection rate of non ulcerative form to 80%<br><br>To provide treatment for all active cases of Buruli ulcer detected by passive case detection<br><br>To provide rehabilitation for 10% of patients with disabilities caused by Buruli ulcer detected<br><br>To train 80% of health workers operating in areas where the disease is endemic<br><br>To encourage scientific research focusing on epidemiology, | Early and community based case detection<br><br>Confirmation of cases<br><br>Case management (antibiotics, surgery and prevention of disabilities)<br><br>Advocacy, social mobilization and partnership<br><br>Strengthening health structures<br><br>Supervision, monitoring and evaluation<br><br>Staff training | Hospital/ Facility - Based case management |

| | | | | |
|---|---|---|---|---|
| | | diagnosis, treatment and prevention of Buruli ulcer | | |
| 9. **Guinea Worm Eradication**<br><br>Goal: Nigeria is now GW free | Strengthen surveillance systems to maintain the guineaworm free status of the country | As a WHO certified GWD free nation, establish mechanisms for containing transmission | Passive surveillance activities all over the country.<br><br>Community participatory surveillance strategy (CPSS)<br><br>Cash reward scheme.<br><br>Rumour investigation, documentation and reporting.<br><br>HSAM (health education, advocacy and social mobilization)<br><br>Provision of safe water supply and rehabilitation of broken down sources in NIGEP target and at risk villages.<br><br>Case management and containment strategy. | Village Based Health Workers (VBHW) through the LGA Coordinators |
| **10.Rabies**<br><br>Goal: To control rabies | To prevent the mortality due to rabies by 100% by the year 2020 | To determine the disease burden of rabies by year 2016<br><br>To prevent the mortality due to rabies by 100% by the year 2020 | Administration of human antirabies vaccine (H-ARV)<br><br>Administration of Human Rabies Immunoglobulin (HRI) | Hospital based care |
| **11.Leishmaniasis**<br><br>Goal: To control leishmaniasis | To reduce the morbidity, disability and mortality due to leishmaniasis | To determine disease burden by year 2016<br><br>To reduce the morbidity, disability and mortality by 80% by year 2020 | Early and community based case detection<br><br>Case management (topicals and infusions, surgery and prevention of disabilities) | Home base and hospital care |

| | | | | |
|---|---|---|---|---|
| **12.<u>Yaws</u>**<br>Goal: To control yaws | To reduce the morbidity, disability and mortality due to yaws | To determine disease burden by year 2016<br><br>To reduce the morbidity, disability and mortality by 80% by year 2020 | Early and community based case detection<br><br>Confirmation of cases<br><br>Case management (antibiotics, surgery and prevention of disabilities) | Hospital/ facility based care |
| **13.<u>Dengue</u>**<br>Goal: To control dengue fever | To reduce the morbidity and mortality due to dengue | To determine disease burden by year 2016<br><br>To reduce the morbidity and mortality by 80% by year 2020 | Case management (treatment based on presenting symptoms) | Home base and hospital care |
| **14.<u>Mycetoma</u>**<br>Goal: To control mycetoma | To reduce the morbidity, disability and mortality due to mycetoma | To determine disease burden by year 2016<br><br>To reduce the morbidity, disability and mortality by 80% by year 2020 | Early and community based case detection<br><br>Confirmation of cases<br><br>Case management (antibiotics, surgery and prevention of disabilities) | Hospital/ facility based care |

## 2.2. NATIONAL MILESTONES

Table 8.1: LF Milestones

| | Indicators | 2015 | 2016 | 2017 | 2018 | 2019 | 2020 |
|---|---|---|---|---|---|---|---|
| 1 | Completed mapping of LF and determined LF endemic areas and the population at risk | 774 (100%) | | | | | |
| 2 | Begun implementation of LF MDA in districts requiring LF MDA including loiasis co-endemic areas | 558 (100%) | | | | | |
| 3 | achieving100% geographical coverage in LF endemic districts | 558 (100%) | | | | | |
| 4 | Major urban areas with evidence of LF transmission under adequate MDA (LGA coverage more than 65%) | 371 (90%) | 411 (100%) | | | | |
| 5 | Conducted more than 5 rounds of MDA in endemic IUs with LGA coverage more than 65% and stopped MDA in LF endemic IUs under WHO criteria | 94 (16%) | 133 (22.7%) | 227 (38.7%) | 360 (61.3%) | 558 (100%) | |
| 6 | Conducted first TAS activities in LF endemic IUs after at least 5 rounds of MDA | 30 (5.1%) | 99 (16.9%) | 129 (22%) | 228 (38.8%) | 357 (60.8%) | 558 (100%) |
| 7 | Conducted and Passed at least 2 TAS activities in IUs | 29 (4.9%) | 29 (4.9%) | 98 (16.7%) | 127 (21.6%) | 225 (38.3%) | 352 (60%) |
| 8 | Started passive surveillance and vector control activities in IUs. | 29 (4.9%) | 29 (4.9%) | 98 (16.7%) | 127 (21.6%) | 225 (38.3%) | 352 (60%) |
| 9 | Proportion and number of IUs where there is full coverage of morbidity- management services and access to basic care | 89 (11.5%) | 173 (22.4%) | 243 (31.4%) | 294 (38%) | 480 (62%) | 774 (100%) |
| 10 | Proportion and number of IUs where 75% of hydrocele cases benefitted from appropriate surgery | 78 (10%) | 147 (19%) | 204 (26%) | 294 (38%) | 480 (62%) | 774 (100%) |

Table 8.2: Oncho Elimination Milestones

| | Indicators | 2015 | 2016 | 2017 | 2018 | 2019 | 2020 |
|---|---|---|---|---|---|---|---|
| 1 | Completed mapping/delineation of oncho and determined oncho endemic areas and the population at risk | 774 (100%) | | | | | |
| 2 | Begun implementation of oncho MDA in districts requiring MDA including loiasis co-endemic areas | 473 (100%) | | | | | |
| 3 | achieving100% geographical coverage in Oncho endemic districts | 468 (99%) | 473 (100%) | | | | |
| 4 | Conducted more than 10 rounds of MDA in endemic IUs with LGA coverage more than 65% | 418 (88%) | 418 (88%) | 418 (88%) | 418 (88%) | 418 (88%) | 418(88%) |
| 5 | Conducted Phase 1a Epid evaluation activities in oncho endemic IUs after at least 10 rounds of MDA | 118 (25%) | 118 (25%) | 177 (38%) | 177 (38%) | 200 (42%) | 230 (49%) |
| 6 | Conducted and Passed epidemiological and entomological assessment in IUs | 0 (0%) | 0 (0%) | 29 (6%) | 29 (6%) | 118 (25%) | 177 (38%) |
| 7 | Proportion and number of IUs where treatment has been stopped | 0 (0%) | 0 (0%) | 0 (0%) | 0 (0%) | 0 (0%) | 177 (38%) |

Table 8.3: SCH Elimination Milestones

| | Indicators | 2015 | 2016 | 2017 | 2018 | 2019 | 2020 |
|---|---|---|---|---|---|---|---|
| 1 | Completed mapping of SCH and determined areas above intervention threshold and the Endemic population | 774 (100%) | | | | | |
| 2 | Begin implementation of school-based/community-based treatments in Endemic LGAs | 302 (46%) | 571 (86%) | 661 (100%) | | | |
| 3 | Achieving100% geographical coverage in SCH Endemic LGAs | 302 (46%) | 571 (86%) | 661 (100%) | | | |
| 4 | Conducted 3-5 years of consecutive treatments in all Endemic LGAs with LGA coverage more than 75% | 77 (12%) | 154 (24%) | 250 (38%) | 400 (61%) | 500 (76%) | 661 (100%) |
| 5 | Conducted first impact assessment activities in at least 50% of SCH Endemic LGAs after at least 3 years of consecutive treatments | 9(24.3%) | 12(32.4%) | 15(40.5%) | 15(40.5%) | 15(40.5%) | 24(64.8%) |
| 6 | Endemic LGAs achieving moderate morbidity control | 481 (73%) | 661 (100%) | | | | |
| 7 | Endemic LGAs achieving advanced morbidity control | 481 (73%) | 661 (100%) | | | | |
| 8 | Endemic LGAs achieving elimination of transmission | 0 (0%) | 0(0%) | 0(0%) | 0(0%) | 77 (12%) | 154 (24%) |

Table 8.4: STH Elimination Milestones 2015-2020

| | **Indicators** | **2015** | **2016** | **2017** | **2018** | **2019** | **2020** |
|---|---|---|---|---|---|---|---|
| **1** | Completed mapping of STH and determined areas above intervention threshold and the Endemic population | 774 (100%) | | | | | |
| **2** | Begin implementation of school-based/community-based treatments in Endemic LGAs | 482 (100%) | | | | | |
| **3** | Achieving100% geographical coverage in STH Endemic LGAs | 482 (100%) | | | | | |
| **4** | Conducted 3-5 years of consecutive treatments in all Endemic LGAs with LGA coverage more than 75% | 77 (16%) | 154 (32%) | 250 (52%) | 350 (73%) | 400 (83%) | 482 (100%) |
| **5** | Conducted first impact assessment activities in at least 50% of STH Endemic LGAs after at least 3 years of consecutive treatments | 9(24.3%) | 12(32.4%) | 15(40.5%) | 15(40.5%) | 15(40.5%) | 24(64.8%) |
| **6** | Endemic LGAs achieving moderate morbidity control | 482(100%) | | | | | |
| **7** | Endemic LGAs achieving advanced morbidity control | 482(100%) | | | | | |

Table 8.5: Trachoma Milestones

| Indicators | 2015 | 2016 | 2017 | 2018 | 2019 | 2020 |
|---|---|---|---|---|---|---|
| Completed mapping of trachoma and determined areas above intervention threshold and the target population | 257 (100%) | | | | | |
| Begun implementation of community-based treatments in target districts | 51 (72%) | 53(75) | 70(100%) | | | |
| Achieved 100% geographical coverage in trachoma target districts | 51 (72%) | 53(75%) | 70(100%) | | | |
| Conducted 3-5 rounds of treatments in target districts with LGA coverage more than 75% | 27 (42%) | 42 (65%) | 52 (80%) | 70 (100%) | | |
| Conducted first impact assessment activities in trachoma target districts after at least 3 rounds of treatments | 16(25%) | 31 (44%) | 34 (49%) | 64 (91%) | 64 (91%) | 70(100%) |
| Started passive surveillance in IUs. | | 16 (25%) | 31 (44%) | 34 (49%) | 64 (91%) | 70(100%) |
| Proportion and number of target districts where there is full coverage of case-management services | 146 (61%) | 180 (76%) | 197 (83%) | 237 100(%) | | |
| Target LGAs achieved elimination of blinding trachoma | 16 (25%) | 31 (44%) | 34 (49%) | 64(91%) | 64(91%) | 70 (100%) |

Table 8.6: IDM MILESTONES ,2015-2020

| Indicators | 2015 | 2016 | 2017 | 2018 | 2019 | 2020 |
|---|---|---|---|---|---|---|
| Active Case detection in 100% of Highly endemic LGAs | 9 (50%) | 18 (100%) | | | | |
| Passive case detection in 100% of other endemic LGAs | 9 (47%) | 19 (100%) | | | | |
| Manage all patients in peripheral health facilities | 18 (26%) | 19 (52%) | 37 (100%) | | | |
| Refer severe and complicated cases for management at district hospitals and reference centres | 18 (26%) | 19 (52%) | 37 (100%) | | | |
| Achieved 100% geographical coverage of SAFE in trachoma target LGAs | 6(41%) | 9 (61%) | 11 (80%) | 14 (100%) | | |
| Achieved 100% treatment coverage of identified HAT and leprosy cases | 5(13%) | 10 (26%) | 14 (39%) | 37 (100%) | | |
| Achieved 100% treatment coverage of identified cases for other CM-NTDs | 5(13%) | 19 (52%) | 24 (65%) | 29 (78%) | 37 (100%) | |
| Started passive surveillance in at least 50% of target LGAs for CM-NTDs targeted for elimination (HAT, Leprosy) | No (%) | No (%) | No (%) | No (%) | 37 (100%) | |
| Started sentinel site surveillance in at least 50% of target LGAs for for CM-NTDs targeted for elimination (HAT, Leprosy) | No (%) | No (%) | No (%) | No (%) | No (%) | 37 (100%) |
| Target LGAs that sustained elimination of leprosy and achieved elimination of HAT | No (%) | No (%) | No (%) | No (%) | No (%) | 37 (100%) |

## PART THREE: OPERATIONAL FRAMEWORK

The operational framework component of this NTD master plan describes how Nigeria will in practice implement the planned activities. It also explains what the country's capacity needs are, how resources will be mobilized, how potential risks will be addressed, scale-up strategy, verification and assessment of disease elimination and how the sustainability of the project achievements will be ensured. Through experiences from implementing the previous master plan, this part explains clearly how the programme outcomes will be attained.

The operational plan has been produced in consultation with all stakeholders in order to allow wide coverage of the programme, harmonize and align available resources, avoid duplication of activities and waste of resources and to yield desired results even within the existing constrained resources for NTD control.

### 3.1. SCALING UP ACCESS TO NTD INTERVENTIONS AND TREATMENT AND SERVICE DELIVERY CAPACITY

This section provides a detailed description of the activities that forms the basis for scaling up of the NTD control programme. Based on the WHO manuals: 'Preventive chemotherapy' and 'Approaches to implementation of integrated NTD programmes', the guidelines on case management diseases, and disease-specific guidelines, the following three packages of interventions are recommended by WHO:

- Preventive chemotherapy;
- Case management/chronic care;
- Transmission control (which includes vector and reservoir control as well as improvements in sanitation and water quality and supply, environmental manipulation).

Mass drug administration, case management and transmission control are the main strategies that will be used to achieve the stated goals and objectives in the control of NTDs in Nigeria. NTDs that share the same strategy have been put together in packages and a summary of the contents of this information in are presented in separate tables.

#### 3.1.1 Scaling up preventive chemotherapy interventions

Preventive chemotherapy is a package of activities for mass distribution of drugs to target populations. A brief description of the interventions and details on the package of activities are shown on the table below. Depending on the types of diseases targeted and their overlaps there will be variations in types and numbers of the drug combinations distributed at a particular time.

The table below provide detail packages of mass drug administration.

Table 9: Types of mass drug administration

| Cross-cutting MDA types | Delivery channels | Timing of treatments | Disease Combination | Requirements | Target (districts) - list | Other mass disease control interventions |
|---|---|---|---|---|---|---|
| MDA1 One annual round of MDA ivermectin and albendazole; | Community-based campaigns/ CDTI. | Annually | Lymphatic Filariasis, Onchocerciasis, | - Training of health personnel;<br>- Training of teachers & community | MDA1= 543 LGAs | EPI campaigns, ITN distribution and re-treatment |

| Cross-cutting MDA types | Delivery channels | Timing of treatments | Disease Combination | Requirements | Target (districts) - list | Other mass disease control interventions |
|---|---|---|---|---|---|---|
| TI, School-based treatment with (PZQ & ALB/MBD).<br><br>T2<br><br>T3 ALB/MBD).<br><br>MDA4 One annual round of MDA with Azithromycin; | School-based campaigns.<br><br>School-based campaigns<br><br>Community Based | Annually<br><br>Bi-annually<br><br>Annually | Schistosomiasis,STH<br><br>Schistosomiasis<br><br>STH<br><br>Trachoma | volunteers;<br>- Social mobilization; Supervision;<br><br>-Production of tools;<br>- Logistics for drug distribution and management. | TI=361 LGAs<br><br>T2=228 LGAs<br><br>MDA3=125LGAs<br>T3= 121 LGAs<br>MDA4=57 LGAs | Home management of Malaria and<br><br>Vitamin A supplement |

<u>Legend</u>

MDA1 = Ivermectin + Albendazole T1 = Praziquantel + Albendazole or Praziquantel +Mebendazole
T2 = Praziquantel only
MDA3 = Ivermectin only (CDTI) T3 = Albendazole or Mebendazole only
MDA4 = Azithromycin only

Table 10: Activities for strategic priority 1 –Scale up Access to PCT interventions

| **Activity** | **Details (sub-activities)** | **Time frame** | **Resources needed** |
|---|---|---|---|
| Strategic objective 1: Scale up an integrated preventive chemotherapy, including access to interventions for `lymphatic filariasis, soil transmitted helminthiasis, onchocerciasis, schistosomiasis and trachoma | | | |
| Procurement of medicines | -Identify reliable sources of NTDs control drug<br><br>-Develop & Submit Reapplications for PCT drugs<br><br>-Procure Mectizan (average of 280 million tablets annually)<br><br>-Procure Albendazole (average of 90 million tablets annually)<br><br>-Procure praziquantel (average of 75 | Annually | Training modules, Personal, Transport, Equipment/materials |

| | | | |
|---|---|---|---|
| Delivery of PCT Medicines | million tablets annually)<br><br>-Procure Mebendazole (average of 30 million tablets annually)<br><br>-Procure Zithromax (average of 20 million tablets annually)<br>-Clear Drugs at Port<br><br>-Store Drugs<br><br>- Conduct feasibility study on the available storage facility at state and LGAs<br><br>-Training on drug supply chain management<br><br>-Coordinate delivery to States, LGAs and Communities | | |
| Conduct Sensitization and mobilization to improve participation | **-**Advocate for inclusion of all NTDs control in curricula of Health training schools<br><br>-Identify major training needs for NTDs control in Nigeria.<br><br>- Build capacity of NTD personnel at all levels of implementation and Surveillance.<br><br>- Carry out refresher training for health workers for management of NTDs disabilities.<br><br>- Training of school teachers for school based de-worming campaigns. | First and second quarter of 2015<br>Annually Subsequently | Training modules, Personal, Transport, Equipment/materials |
| Conduct mapping of NTDs | -Mapping of Schisto/ STH and LF in Adamawa, Borno, Ekiti (STH)and Yobe.<br><br>-Mapping of *Loa loa .in 195 LGAs*<br><br>-Delineation of ivermectin treatment areas in Bayelsa, Rivers, Edo, Ebonyi, Borno, Bauchi, Delta,Lagos, Oyo,Ogun and Akwaibom. | 3rd and 4th quarter | Training, Personal, Transport, Equipment/materials |
| Build capacity for PCT interventions at all levels | **-**Strengthen the existing drug management system at all levels.<br><br>- Maintain an efficient and sustainable drug clearance, storage and distribution | 1st - 4th quarter<br>Annually Subsequently | Personal, Transport, Equipment/materials |

| | | | |
|---|---|---|---|
| | system. | | |
| Conduct MDA for PCT; Report and record PCT activities | - Train personnel for NTDs at State, LGAs and community levels<br><br>-Community Mobilization Meetings & campaigns<br><br>-Distribution of drugs & commodities to community members<br><br>-Registration of Community members<br><br>-Collection of treatment & training data<br><br>-Collation of data<br>-Preparation of periodic reports | Annually | Personal, Transport, Equipment/materials |
| Conduct advocacy Visits to Policy Makers | -Develop plans for advocacy Visits<br>-Secure needed logistics<br>-Meet with Policy Makers<br>-Follow up on commitment secured | Annually | Personal, Transport, Equipment/materials |

### 3.1.2 Scaling up NTD Case management Interventions

The case management package of activities includes identification (active and passive case finding) and management of patients of a specific NTD. Based on the targeted NTDs and the overlap in their requirements, the package of activities is summarized on table 12.

The algorithm in annex 2.16 shows co-endemicity of NTDs controllable mainly by case management interventions in Nigeria. This format is based on groups of states having the same co-endemic CM-NTDs.

Table 11: Activities for case management interventions

| Activity | Details (sub-activities) | Time frame | Resources needed |
|---|---|---|---|
| Strategic Objective 2: Scale up integrated case-management-based disease interventions, especially the following : leprosy, leishmaniasis, buruli ulcer, lymphoedema, trichiasis, guineaworm disease, yaws, rabies, dengue and mycetoma | | | |
| Advocacy, Sensitization/resource mobilization | Engagement of advocacy champions<br><br>Development of advocacy tools<br><br>Advocacy visit to key stake holders<br><br>Sensitization visit to policy makers<br><br>Advocacy and social mobilization in selected States | 2015<br><br>2015<br><br>Annually<br><br>Annually<br><br>Annually | Logistics, Transport, Refreshment, Hall, |
| Strengthening partnership | .Identification of new partners<br><br>.Stakeholders meeting | 2015 – 2017<br><br>Annually<br>Annually | Logistics, Transport, Refreshment, Hall, Stationery |

| | | | |
|---|---|---|---|
| | . Joint activities with stakeholders | | |
| Inter/Intra-sectorial collaboration | . Regular divisional meetings<br><br>. Regular task force meeting<br><br>. Programme review meetings at the states, zones and National<br><br>.Regular Steering committee meetings | Monthly<br><br>Annually<br>Annually<br><br>Annually | Logistics, Transport, Refreshment, Hall, Stationery |
| Health promotion and education | . Integration of NTDs into health education and promotion activities at the FMoH<br><br>. Development, production and distribution of IEC materials<br><br>Production and distribution of branded items for NTDs<br><br>Regular TV/ radio and documentary on NTDs | 2015 -2017<br><br>2015 -2017<br><br>Annually<br><br>Monthly | Logistics, Transport, Refreshment, Hall, Stationery |
| Capacity building (Training and re-training/ procurement, Quality control for drug and commodity supplies) | Training and retraining on the following (epidemiological Survey, CM , treatment, data management, micro planning, and drug supply chain management | Annually | Logistics, Transport, Refreshment, Hall, Stationery |
| Mapping/Epidemiological surveys | Procurement of survey materials<br><br>Conduct epidemiological survey | 2015 - 2017 | Logistics, Transport, Refreshment, Hall, Stationery, Diagnostic materials |
| Surveillance | Training on routine surveillance with the IDSR form<br><br>Prompt rumour reporting and investigation | 2015 - 2017<br><br>Annually | Logistics, Transport, |
| Passive case finding | Training on routine surveillance with the IDSR form<br><br>Procurement and distribution of NTD surveillance materials | 2015 - 2017<br><br>Annually | Reporting forms |
| Active case finding | Procurement and distribution of necessary tools for case search<br><br>Promote integration with other disease control programme | Annually<br><br>Annually | Logistics, Transport, Stationery |
| Promotion of WASH | Production of IEC materials<br><br>Advocacy for safe water supply<br><br>Advocacy to line Ministries | Biannually<br><br>Annually<br><br>Annually | Logistics, Transport, Refreshment, Hall, Stationery |
| Supervision | Training of M & E personnel | Annually | Logistics, Transport, Refreshment, Hall, |

| | | | |
|---|---|---|---|
| | Development, production and distribution of supervisory tools<br><br>Conduct in-process and end-process supervisory visits | Annually<br><br>Annually | Stationery |
| Medical treatment | Procurement or application for donation of CM drugs (Anti rabies vaccine, Efflonithine, MDT, NECT, Penicillin, Rifampicin and Streptomycin, Tetracycline Eye Ointment, Zithromax, Peadeatric Oral Suspension, Meglumine antimoniate, Sodium stibogluconate, Lipsonal amphotericin, Paromomycin, Pentamidine, Miltefosine)<br><br>Delivery of IDM drugs | Annually<br><br>Annually | Logistics, Transport, Stationery, Drugs, Personnel |
| Surgery | Identify cases for surgery<br><br>Conduct selected surgeries for hydrocele, BU, Leprosy, and trichiasis | Annually<br>Annually | Logistics, Transport, Surgical equipment's,, Stationery, Personnel |
| Prevention of disability | Training and retaining of Health personnel to Improve suspicion index<br><br>Improved Publicity<br><br>Equip selected health facilities to act as centers for prompt case management | Annually<br><br>Quartely<br><br>Biannually | Personnel, Logistics, Stationery, Consumables |
| Integrated vector management/ reservoir control | Collaboration with NMCP on distribution of nets and other disease programmes on vector control<br><br>Molluscicicling of water bodies<br><br>Inter sectorial collaboration with other line ministries especially Water Resources to modify dams and reduce vectors | Annually<br><br>2017 -2020<br><br>Annually | Personnel, Logistics, Transport, equipment's and materials |
| Data management/quality assessment | Training and retraining of personnel at all levels<br><br>Procurement of computers and hard drives<br><br>Production and distribution of data collection forms | Annually<br><br>2015<br><br>2015 | Logistics, Transport, Refreshment, hall, personnel, equipments and materials Stationery |
| Impact studies | Identification of study sites<br><br>Production of tools for impact studies<br><br>Training of personnel<br><br>Conduct impact studies | Annually<br><br>2015<br><br>Annually<br><br>Annually | Personnel, training modules, Logistics, Secretariat, Refreshment, Hall, Stationery |
| Operational research | Identification of research needs | Annually | Personnel, Materials, |

| | | | |
|---|---|---|---|
| | Training of personnel | Annually | logistics, equipment's an d materials |
| | Procurement of research materials | Annually | |
| | Conduct operational study | Annually | |

The case management and chronic care is showcased in table 12. The targeted NTDs are listed, with the requirements. Some of the items cut across the listed NTDs.

Table 12: Package 3.2: Case management and chronic care.

| **Cross-cutting interventions** | **NTDs targeted** | **Requirements** | **Other non-NTD opportunities for integration** |
|---|---|---|---|
| Advocacy, Sensitization/resource mobilization | GW, Leprosy, Yaws, HAT, BU, Trichiasis, Human antirabies vaccine (HARV), Rabies Immunoglobulins,Ecch, Cyst, LF (hydrocele and lymphoedema), Other Zoonotic Diseases | Advocacy champion, IEC Materials, personnel, | Capacity building for the legislative |
| Strengthening partnership | GW, Leprosy, Yaws, HAT, BU, Trichiasis, Human antirabies vaccine (HARV), Rabies Immunoglobulins,Ecch, Cyst, LF (hydrocele and lymphoedema), Other Zoonotic Diseases | Logistics, Stationery, Meetings ,,follow up, | Enlightenment |
| Inter/Intra-sectorial collaboration | GW, Leprosy, Yaws, HAT, BU, Trichiasis, Human antirabies vaccine (HARV), Rabies Immunoglobulins,Ecch, Cyst, LF (hydrocele and lymphoedema), Other Zoonotic Diseases | Meetings, Stationary, | Sharing of data and information |
| Health promotion and education | GW, Leprosy, Yaws, HAT, BU, Trichiasis, Human antirabies vaccine (HARV), Rabies Immunoglobulins,Ecch, Cyst, LF (hydrocele and lymphoedema), Other Zoonotic Diseases | IEC materials, Radio/ TV jingles | Health promotion week |
| Capacity building (Training and re-training/ procurement, Quality control for drug and commodity supplies) | GW, Leprosy, Yaws, HAT, BU, Trichiasis, Human antirabies vaccine (HARV), Rabies Immunoglobulins,Ecch, Cyst, LF (hydrocele and | Stationery, Hall, training modules, facilitators | Pharmaceutical department drug supply chain training |

| | | | |
|---|---|---|---|
| | lymphoedema), Other Zoonotic Diseases | | |
| Mapping/Epidemiological surveys | Leprosy, Yaws, HAT, BU, Trichiasis, Human antirabies vaccine (HARV), Rabies Immunoglobulins,Ecch, Cyst, LF (hydrocele and lymphoedema), Other Zoonotic Diseases | Diagnostic tools, personnel, vehicles, logistics, drugs | IDSR |
| Surveillance | GW, Leprosy, Yaws, HAT, BU, Trichiasis, Human antirabies vaccine (HARV), Rabies Immunoglobulins,Ecch, Cyst, LF (hydrocele and lymphoedema), Other Zoonotic Diseases | personnel, vehicle, logistics | IDSR |
| Passive case finding | GW, Leprosy, Yaws, HAT, BU, Trichiasis, Human antirabies vaccine (HARV), Rabies Immunoglobulins,Ecch, Cyst, LF (hydrocele and lymphoedema), Other Zoonotic Diseases | personnel, vehicle | IPD |
| Active case finding | Leprosy, Yaws, HAT, BU, Trichiasis, Human antirabies vaccine (HARV), Rabies Immunoglobulins,Ecch, Cyst, LF (hydrocele and lymphoedema), Other Zoonotic Diseases | personnel, vehicle, logistics | |
| Promotion of WASH | GW, Leprosy, Yaws, HAT, BU, Trichiasis, Human antirabies vaccine (HARV), Rabies Immunoglobulins,Ecch, Cyst, LF (hydrocele and lymphoedema), Other Zoonotic Diseases | IEC, Personnel, | National hand washing days |
| Medical treatment | Leprosy, Yaws, HAT, BU, Trichiasis, Human antirabies vaccine (HARV), Rabies Immunoglobulins,Ecch, Cyst, LF (hydrocele and lymphoedema), Other Zoonotic Diseases | Drugs, medical doctors and nurses; Follow-up and supervision. | Capacity building for basic skills at the district level. |

| | | | |
|---|---|---|---|
| Surgery | Leprosy, BU, Trichiasis, LF (hydrocele and lymphodema) | Refresher and retraining of medical doctors and nurses;<br>Surgical kits, dermatome and mesh graft (for skin grafting);<br>Hospital facilities or appropriate basic facilities with good surgical facilities;<br>Follow-up and supervision. | Capacity building for basic surgery skills at the district level. |
| Morbidity Management | Leprosy, BU, Trichiasis, LF, (hydrocele and lymphoedema) | Refresher and retraining of medical doctors and nurses;<br>Surgical kits, dermatome and mesh graft (for skin grafting);<br>Hospital facilities or appropriate basic facilities with good surgical facilities;<br>Follow-up and supervision | Capacity building for basic surgery skills at the district level |
| Prevention of disability | Leprosy, BU, Trichiasis, LF (hydrocele and lymphoedema) | Training of medical doctors and nurses;<br>Surgical kits, dermatome and mesh graft (for skin grafting);<br>Hospital facilities or appropriate basic facilities with good surgical facilities;<br>Follow-up and supervision. | Capacity building for basic surgery skills at the district level. |
| Integrated vector management/ reservoir control | HAT, BU, Trichiasis, LF | personnel, LLITNs, Abate, | LLITN distribution by NMCP |
| Data management/quality assessment | GW, Leprosy, Yaws, HAT, BU, Trichiasis, Human antirabies vaccine (HARV), Rabies Immunoglobulins,Ecch, Cyst, LF (hydrocele and lymphoedema), Other Zoonotic Diseases | training, computers, personnel | HMIS |
| Impact studies | GW, Leprosy, Yaws, HAT, BU, Trichiasis, Human antirabies vaccine (HARV), Rabies Immunoglobulins,Ecch, Cyst, | personnel, logistics and diagnostic kits | |

| | | | |
|---|---|---|---|
| | LF (hydrocele and lymphoedema), Other Zoonotic Diseases | | |
| Operational research | GWD,Leprosy, Yaws, HAT, BU, Trichiasis, Rabies,Ecch, Cyst, LF Complications, Other Zoonotic Diseases | personnel, logistics and diagnostic kits | Academia/published research works |

### 3.1.3 Scaling Up NTD Transmission Control Interventions

NTD transmission control activities are cross-cutting for both vector-borne diseases and other diseases. In effect, transmission control interventions are complementary to preventive chemotherapy and case management and, as such, they need to be conducted in all NTD endemic areas. These also include PHASE activities as presented in tables 12 and 13

- P - Preventive chemotherapy
- H - Health Education
- A - Access to clean water
- S - Sanitation Improvement
- E - Environmental manipulation

Table 12: Intervention packages for Transmission control

| Cross-cutting interventions | Targeted NTDS | Requirements | Other non-NTD opportunities for integration |
|---|---|---|---|
| Mosquito and sand-fly control:<br>• Insecticide treated nets;<br>• Indoor residual spraying;<br>Snail control<br>• Mapping of water bodies<br>• Use of mollucscide for treatment of water bodies<br>• Introduction of predators e.g ducks into water bodies<br>Environmental management.<br>• Provision of motorize boreholes/potable water in rural communities<br>• Provision of VIP latrine<br>• Campaign on the vaccination of intermediate host of rabies | Lymphatic filariasis,<br>Onchocerciasis,<br>Leishmaniasis,<br>Schistosomiasis,<br>STH,GWD, HAT<br>Rabies | LLINs<br>DDT<br>Plastering of walls<br>Mollucside<br>Aerial larviciding for water bodies | Malaria vector control;<br>Integrated vector management (IVM).<br>Water Agency (RUWASA)<br>Line ministries such as SUBEB<br>Ministry of Environment, Ministry of Education, Ministry of water resources, Ministry of science and technology, Veterinary department of ministry of Agriculture |

Summary of key activities planned to be carried out for transmission control package as defined above.

Table 13: Activities for disease transmission control

| Activity | Details (sub-activities) | Time frame | Resources needed |
|---|---|---|---|
| Strategic objective 3: Strengthening integrated vector management and other "PHASE" interventions for the targeted NTDs. | | | |
| Provision of mollucscide, IRS and outdoor sprays | Forecast and procure mollucside, IRS/outdoor sprays<br>Distribute spray equipment and chemicals | 2016 and 2018 | Transport for distributors<br>Procure IRS/outdoor sprays and mollucscides. Training of health workers on the use of mollucscides |
| Mosquito and sand-fly control:<br>• Insecticide treated nets; | Training of NTDs health team and community volunteers on the use of LLINs, IRS and personal hygiene | Anually | LLINs and health education,<br>Training of Health workers |
| Provision of VIP Latrines | Conduct advocacy to Ministries of Education, Environment and LGA<br><br>Collaborate with SUBEB in the building of VIP latrines and toilets in selected schools/villages | 2016 to 2020 in tranches | Procurement and distribution of VIP latrine |
| Provision of motorized boreholes/potable water in rural communities | Collaborate with water agency (RUWASA) in the provision of boreholes and portable water in rural communities | 2016 – 2020 in tranches | Advocacy visit and mobilization |
| Use of predators in the reduction of intermediate host (snail) | Encourage and collaborate with LGAs and local communities to purchase Ducks and Geese | 2015 – 2020 in tranches | Purchase of Ducks and Geese |
| Campaign on basic hygiene (hand,/face washing, proper waste disposal etc | Conduct discussion in mass media on environmental control. Liaise with RUWASA on Sanitation.<br>Production of IEC materials on WASH<br>Advocate proper hand wash and hygiene among school children | 2015 – 2020 | Radio Airtime/hr<br>TV Airtime/hr<br>Development and printing of IEC material, sensitization visit on hand wash culture in schools |
| Campaign on proper care and vaccination of pets such as Dogs and Cats | Campaigns using media houses etc | Annually | Sensitization and procurement/distribution of rabies vaccine |

### 3.2. PHARMACOVIGILANCE IN NTD CONTROL ACTIVITIES

This section provides information and details on preparedness of the national Pharmaco-vigilance system and NTD programme management to ensure satisfactory reporting and management of side effects and adverse events that may be linked to NTD interventions under the programme setting. In reference to the following guideline: WHO (2009 –Draft). *Management of serious adverse events following preventive interventions for the control of neglected tropical diseases: practical advice for national programme managers*, Geneva, World Health Organization. Geneva, this section also describes how NTDs control activities can be incorporated into the already existing pharmacovigilance system, particularly showing the list of existing forms used for reporting serious adverse effects.

The Nigeria Pharmaco-vigilance Programme was established in 2003. It is coordinated by the National Pharmaco-vigilance Centre (NPC) which is located in NAFDAC and collaborates with the Uppsala Monitoring Centre (UMC) and other national centers worldwide. NPC is responsible for monitoring the safety of all medicines in Nigeria. The National Pharmaco-vigilance Centre will be assisted as the case requires by a National Advisory Committee comprising of experts from various fields of health care. The National Pharmaco-vigilance Centre is responsible for providing reporting forms, collecting, evaluating and communicating the findings from ADR reports to the management of NAFDAC, who may communicate same to council for ratification.

NAFDAC uses the findings from the reports for making regulatory decisions on how to prevent or minimize the risk of ADRs in Nigeria. NAFDAC, through the National Pharmaco-vigilance Centre, may communicate their findings, recommendations and directives to appropriate organisations or individuals. These include, but are not limited to health professionals, pharmaceutical manufacturers, public health programmes within the Federal and State Ministries of Health, other public and private health institutions, the media and the public. On the integrated NTDs programme, the pharmacist occupies the unique position as the logistician for medicines to ensure that accurate forecasts are received from the various NTD specific programmes. He/she prepares the ordering and bidding of tenders for drug supplies to the various NTD programmes as well as ascertaining that medicines supplied to are of good quality, safe and efficacious as well as serve as the programme's focal/contact point for every adverse drug reaction observed. Reports of adverse drug reaction and events are submitted using the standardized forms through appropriate national channels. The pharmacist liaises with the National Pharmaco-vigilance Centre on feedbacks from the field as part of the programme's contribution to the National Pharmaco-vigilance Monitoring System. He requires appropriate training to function effectively and serve as a trainer for programme officers at lower levels of operation.

The activities to be implemented are identified in table below

Table 14: Activities for strengthening pharmaco-vigilance in NTD programmes.

| Activity | Details (Sub-activities) | Timing/ Frequency | Resources needed |
|---|---|---|---|
| Strategic Objective : Strengthening the reporting and response to severe adverse events (SAEs) by leveraging on-going efforts to strengthen pharmacy-vigilance systems in States | | | |
| Adopt Guidelines & Forms for Management of Adverse Drug | Adopt guidelines for management and referral systems of Adverse Events | 2015 - 2016 | Secretariat and logistic support; printing |
| | Adopt forms for reporting of | January – | Secretariat and logistic |

| | | | |
|---|---|---|---|
| Reaction/ Events | Adverse Events | December, 2016 | support; printing |
| Hold consultative meetings with, Food and Drugs Services Dept., NAFDAC and other key stakeholders for the inclusion of NTDs in the Pharmaco-vigilance system | Introduction of and harmonization of ADR&E reporting forms | Fourth Quarter 2016 | Secretariat and logistic support; printing |

### 3.3. STRENGTHENING CAPACITY AT NATIONAL LEVEL FOR NTD PROGRAMME MANAGEMENT AND IMPLEMENTATION

This section describes what is needed to strengthen the management and operational capacities of the NTD programme staff at various levels. The following guiding principles were used to achieve this:

- Leadership and governance: Structural reform within the health sector relating to NTD programmes and harmonization, alignment, oversight and regulation of interventions.
- Health workforce: Strengthening programme staff capacity (training needs to enhance human resources) for the purpose of the control activities. The category (e.g. laboratory diagnosis), level, type or number of training events required, and units involved in training is indicated;
- Medical products, vaccines and technologies: establish and enforce norms, standards, policies, reliable procurement practices for drug quality.
- Full contact details of NTD programmes at national level.
- An overview of existing capacity at national level for NTD Programme management and implementation against each of the points above. An indication is given on what improvement will be made where these are seen necessary.

A summary table is provided on the key activities planned to be implemented and the resources estimated needed (see table 15)

Table 15: Activities for Strengthening Capacity at National and Zonal Level and Programme Management

| Activity | Details (Sub-activities) | Timeframe | Resources needed |
|---|---|---|---|
| **Strategic Objective 4: Strengthen capacity at national level for NTD programme management and implementation.** | | | |
| **TRAININGS** | | | |
| Training at national & zonal levels on PCTs & IDMs interventions | • Meetings with State Policy Makers & briefing of focal points<br>• Hold national training on development of State NTD plans<br>• Transmission Assessment Survey (TAS) training at national and zonal levels<br>• Monitoring, Evaluation, Supervision and Surveillance training | Annually | Telephone & Internet services, Secretariat and logistic support, transport, refreshments, hall and DSA |

| **Activity** | **Details (Sub-activities)** | **Timeframe** | **Resources needed** |
| --- | --- | --- | --- |
| | • Training of Health personnel on mHealth at national and zonal levels<br>• Trainings at national & zonal levels on NTD Data base management and TIPAC tools<br>• Training on LMIS (Logistic Management Information System)<br>• Integrated training of national and zonal level officers on diagnosis of Rabies, Leishmaniasis, mycetoma and Yaws.<br>• Training on rabies vaccine handling and distribution | | |
| Training on PCT interventions at State, LGA and community levels | • Training on maintenance of an efficient and sustainable drug storage and distribution system.<br>• Monitoring, Evaluation, Supervision and Surveillance training at the State, LGA and community level<br>• Training on LMIS (Logistic Management Information System) at the State, LGA and community level<br>• Training of Health personnel on mHealth at State and LGA levels<br>• Refresher training for health workers for management of NTDs disabilities.<br>• Re-orientation of NTD staff at State and LGA<br>• Training of school teachers for school based de-worming campaigns<br>.<br>• Conduct training workshop on resource mobilization & management for NTD technical office<br>• Integrated training of state,LGA and health facility level officers on diagnosis of Rabies, Leishmaniasis, mycetoma and Yaws | Annually | Personal, Transport, Equipment/materials |

| **Activity** | **Details (Sub-activities)** | **Timeframe** | **Resources needed** |
|---|---|---|---|
| | • Training on rabies vaccine handling and distribution | | |
| Training at national & zonal levels on resource mobilization & programme management | • Training at zonal level on resource mobilization & programme management | 2015 - 2018 | Telephone & Internet services, Secretariat and logistic support, transport, refreshments, hall and DSA |
| Structural strengthening of National officers of the NTD programme and National reference laboratories for NTDs | • Training of National officers of the NTD programme and National reference laboratories for NTDs | 2016 - 2018 | |
| Training on development of LGA NTD work plans at State level | • Communication with participants Meetings with State Policy Makers & briefing of focal points | Annually | Telephone & Internet services, Secretariat and logistic support |
| WHO International NTD Program Managers Training | • Participation at WHO International NTD Program Managers Training | Annually | Telephone & Internet services, Secretariat and logistic support |
| Training on Proposal and Report writing | • Training on Proposal and Report writing | Annually | Telephone & Internet services, Secretariat and logistic support |

Based on identified priority areas for initiating programme activities in sections above, a description of how expansion of NTD control activities will progress to attain total coverage by 2020 is represented in table 16 below (scaling up plan/scaling down plan).

Table 16**:** Scaling Up Plan

| NTD | Total No Of Districts Requiring Mda | Total At Risk Population | 2015 No Of Districts And Total Population To Be Treated | 2016 No Of Districts And Total Population To Be Treated | 2017 No Of Districts And Total Population To Be Treated | 2018 No Of Districts And Total Population To Be Treated | 2019 No Of Districts And Total Population To Be Treated | 2020 No Of Districts And Total Population To Be Treated |
|---|---|---|---|---|---|---|---|---|
| PCT IMPLEMENTATION(MDA) | | | | | | | | |
| LF | 557 | 118,306,096 | 544<br>96,156,050 | 530<br>96,332,719 | 483<br>89,825,637 | 469<br>89,389,194 | 435<br>85,593,896 | 262<br>54,991,921 |
| Oncho | 473 | 42,966,736 | 473<br>36,065,592 | 473<br>36,973,415 | 473<br>37,904,120 | 473<br>38,858,281 | 473<br>39,832,267 | 396<br>33,255,108 |
| Schisto | 661 | 43,544,818 | 561<br>37,365,731 | 380<br>24,913,947 | 609<br>41,728,087 | 380<br>26,170,947 | 609<br>43,855,271 | 380<br>28,964,459 |
| STH | 482 | 44,064,527 | 417<br>39,190,422 | 482<br>43,681,552 | 482<br>44,754,129 | 482<br>45,853,608 | 482<br>47,715,470 | 466<br>46,312,823 |
| Trachoma | 69 | 10,010,689 | 52<br>9,903,021 | 60<br>10,102,041 | 51<br>8,701,638 | 25<br>3,639,880 | 21<br>3,253,731 | 8<br>465,234 |
| IDM IMPLEMENTATION | | | | | | | | |
| HAT | 0 | 150000 | not known | not known | not known | not known | not known | not known |
| Leishmaniasis | not known | not known | not known | not known | not known | not known | not known | not known |
| Buruli Ulcer | not known | not known | not known | not known | not known | not known | not known | not known |
| Leprosy | none | 174,000,000 | not known | not known | not known | not known | not known | not known |
| Yaws | not known | not known | not known | not known | not known | not known | not known | not known |
| RABIES | not known | not known | not known | not known | not known | not known | not known | not known |
| Mycetoma | | | | | | | | |

## 3.4. ENHANCING PLANNING FOR RESULTS, RESOURCE MOBILIZATION AND FINANCIAL SUSTAINABILITY

This section describes how the existing and planned systems for planning NTD activities at all levels continuously observed the prevailing trends in Health priorities, Financing and donor strategies. This led to the identification of best strategies of NTD resource mobilization, partnership building and financial sustainability. The objectives primarily aimed at generating adequate funding as well as establishing enabling environment that would suit resource mobilization for implementation of National master plan 2015 – 2020

Below is the description of the guiding principles for the achievement of the set objectives:

- Formulation of an annual operational plan for the control, elimination and eradication of target NTDs at national and sub – national levels in the country
- Incorporation of NTDs in planning at national and sub – national levels
- Development of resource mobilization strategies for NTDs Programme in Nigeria
- Production and use of evidence for resource mobilization
- Establishment of reliable processes and systems to support mobilization
- Institution of good communication channels and information flow mechanisms
- Ensuring enabling and supportive environment
- Establishment of good accountability systems for resource monitoring and control.

As described in the above guiding principles, a summary table is provided below on the key planned activities for implementation to achieve the four strategic objectives for enhancing planning for results, resource mobilization and financial sustainability of National NTD programmes.

Table 17: Enhance planning for results, resource mobilization and financial sustainability of National NTD programmes.

| Activity | Details (Sub-activities) | Timeframe/ Frequency | Resources needed |
|---|---|---|---|
| **Strategic Objective 1: Support States to develop integrated multi-year strategic plans and develop gender-sensitive annual operational plans for the control, elimination and eradication of targeted NTDs.** | | | |
| Hold national training on development of State NTD plans | Venue identification; Mobilize resources; Identify Facilitators; Hold training meeting | January – March 2015 | Secretariat and logistic support |
| Conduct Stakeholders' Meetings in States for adoption of State NTD plans | identification of key stakeholders; Venue Identification; Hold meeting | January – March 2015 | Secretariat and logistic support |
| Revise National annual operational plan for NTDs control to make it gender-sensitive | Identification of gaps; Holding of meeting | Annually | Secretariat and logistic support |
| Revise National NTDs Master Plan | Identification of gaps; SWOT analysis and Holding of meetings | January – March 2015 | Secretariat and logistic support |
| Conduct National Stakeholders Meeting for adoption of the NTDs Master Plan | Venue identification, Identification of key stakeholders and holding of meeting | March 2015 | Secretariat and logistic support |
| Development of Strategic frame work for Elimination | Meetings with Fed. Min. of Agric., academia and Stakeholders, | January – December | Secretariat and logistic support |

| | | | |
|---|---|---|---|
| of Rabies in West Africa (RIWA) | production of framework, dissemination | 2015 | |
| **Strategic Objective 2: Enhance resource mobilization approaches and strategies at national and sub-national levels for NTD interventions.** | | | |
| Develop an NTD Resource Mobilization Guide | Identification of Facilitators & Resource; Development of Guide; Production of Guide | January – April 2015 | Secretariat and logistic support; printing |
| Conduct training workshop on resource mobilization & management for NTD technical officers | Venue identification; Mobilize resources; Identify Facilitators; Hold training meeting | January – April 2015 | Secretariat and logistic support |
| Hold sensitization and resource mobilization workshops for identified prospective funders including philanthropists | identification of key partners; Venue Identification; Hold meeting; Follow up visits on pledges | Annually | Secretariat and logistic support |
| Conduct advocacy Visits to, State and National Policy Makers | Develop plans for advocacy Visits; Secure needed logistics; Meet with Policy Makers; advocacy on counterpart funding at National and State levels, Follow up on commitment secured | Annually | Secretariat and logistic support |
| Regular donor coordination forum at National and State level | Identification of actual and potential NTDs donors (private and public), quarterly meetings, donor forum, Develop mechanism on counterpart funding, regular reports on donor commitment | Annually | Secretariat and logistic support |
| Develop NTD partners mapping | Mapping to reveal NTDs orphans States, Production of maps and sources from partners to work in the orphans States. | January-June 2015 | Secretariat and logistic support |
| Identification of potential partners for NTDs control at all levels | Identification of partners(eg Countdown group) and their areas of operation, Hold meetings with potential partners, Venue identification | Annually | Secretariat and logistic support |
| Conduct visits to Banks, Oil Companies & Multi-national Companies | Identify & select Organizations to be Visited; Develop plans for Visits; Secure needed logistics; Meet with key Managers; Follow up on commitment secured | Annually | Secretariat and logistic support |
| Identification and mobilization of local NGOs, CBOs and religious groups | Venue identification, Hold meeting | Annually | Secretariat and logistic support |
| Conduct rallies, awareness campaign and community seminars | Venue identification, hold meetings, production of T-shirts, aprons, flyers, fact sheets etc. | Annually | Secretariat and logistic support |
| | | | |
| **Strategic Objective 3: Strengthen the integration and linkages of NTD programme and financial plans into sector-wide and national budgetary and financing mechanisms.** | | | |
| Sensitize FMOH Policy | Develop Memos; Sensitize Policy | Quarterly | Secretariat and |

| | | | |
|---|---|---|---|
| Makers on strengthening linkages & enhancing integration with other divisions & departments | Makers at Routine Meetings; Make & Share Reports | | logistic support |
| Visit Line Ministries & Agencies to buy into the NTD Master Plan | Identify & select Ministries/Agencies to be Visited (FMOE, FMOWR, FMOA, National Bureau of Statistics, etc) ; Develop plans for Visits; Secure needed logistics; Meet with key Policy Makers; Follow up on commitment secured | 2015 – 2016 | Secretariat and logistic support |
| Hold Meeting with Other Community-Based Programmes | Contact Reps of Programmes( Malaria, NPHCDA, Fed. Min. of Water resources, community environmental health officers) identified; Hold meeting; follow up on recommendations | Annually | Secretariat and logistic support |
| Engagement and sensitization of Joint Military Task force, Red Cross, Local community vigilante and emergency rescue committee | Identification of Joint Military Task force, Red Cross, Local community vigilante and emergency rescue committee | Annually | Secretariat and logistic support |
| Develop structures in the non-CDI State and LGAs for scale up | Identification of non-CDI States and LGAs, holding Trainings | January – December 2015 | Secretariat and logistic support |
| **Strategic Objective 4: Support States to develop and update national NTD policies and elaborate guidelines and tools to guide effective policy and programme.** | | | |
| Develop Tools for NTD implementation | Collect existing Programme-specific tools; Identify Gaps; develop integrated tools; produce tools | January – December 2015 | Secretariat and logistic support; printing |
| Circulate Revised NTD Policy to States | Hold dissemination meeting, Plan for distribution; distribute documents to States | January – December 2015 | Secretariat and logistic support |
| Conduct State-level meetings on adaptation of guidelines & tools | Venue identification; Mobilize resources; Identify Facilitators; Hold workshops | January – December 2015 | Secretariat and logistic support |
| Revise National Guideline & Tools | Assess Guideline & Tools; Identify Gaps; Revise Documents; Field-test Documents; Produce Documents & Share with Partners | 2017 | Secretariat and logistic support; printing |

### 3.5. STRENGTHNING GOVERNMENT OWNERSHIP, ADVOCACY, COORDINATION AND PARTNERSHIP

This section describes how NTD control would be streamlined at the sector level to establish effective longer - term multi - sectoral involvement at various operating levels, as well as to be responsive to the larger National goals. The existing interaction among the key stakeholders is the basis for describing how streamlining of activities will be effected. Emphasis is made on stakeholders to ensure that required advocacy for NTD control programmes is provided at all levels so that their relevance is kept high on the agenda. The objectives are primarily aimed at strengthening government, ownership, advocacy,

coordination and partnership generating as described in the guiding principles below for National master plan 2015 – 2020 activities:

- Streamlining and in corporation of NTD control in strategic National and sub- national Health plans
- Incorporation of NTD control activities in Health facilities, social services, education and training, employment, local government resources and opportunities
- Regular NTD discussions and knowledge updates in the country coordinating mechanisms at all levels
- Regular reviews of NTD programs involving the steering committee, task forces, secretariats and stakeholders
- Strengthening partnerships and seeking opportunities for collaboration
- Coordination and oversight control activities within the existing structures of the Ministry of Health
- Media engagement
- Community involvement and participation in NTD activities

With the stated guiding principles above the summary table 18 has been developed on the key activities planned for implementation in other to achieve the strategic objectives in strengthening government ownership, advocacy, coordination and partnerships.

Table 18: Strengthening government ownership, advocacy, coordination and partnership

| **Activity** | **Details (Sub-activities)** | **Timeframe/Frequency** | **Resources needed** |
|---|---|---|---|
| **Strategic objective 1: Strengthen coordination mechanism for the NTD control programme at national and sub-national levels.** | | | |
| Establish, equip and manage NTD Focal Points at zonal, State & LGA Levels | Advocacy Meetings with State Policy Makers, Identification & briefing of focal points | Jan – July 2015 | Secretariat and logistic support |
| Strengthen NTD Secretariat at National level | Identification of training and resource gaps; Procurement of relevant logistics; Re-orientation of NTD staff | Annually | Vehicles, Generators & Office equipment (desktop computers, laptop computers, Laser Jet Printers, Photocopiers, Scanners, multimedia projectors, Computer accessories, stationery, fax machine, Telephone services & Maintenance, Internet services & maintenance) |
| Update guidelines (Resource mobilization, Standard Operating Procedure (SOP) for school and Community based De-worming activities, Case management and PCT, Management of SAEs | Holding meetings of NTD Steering Committee & ad-hoc sub-committees; Production of guidelines; Distribution of guidelines to States/LGAs | January – December 2015 | Secretariat and logistic support; printing |

| Activity | Details (Sub-activities) | Timeframe/Frequency | Resources needed |
|---|---|---|---|
| and M and E), for NTD Implementation | | | |
| Develop annual NTD Work plans with inputs from partners | Development of Programme-Specific work Plans; Holding of NTD Branch Meetings; Integration of Programmes' Work plans | Annually | Secretariat and logistic support |
| Hold NTDs partners forum | Venue identification, holding of meetings ,invitation of partners, identification of venue | Annually | Secretariat and logistic support |
| Create task force for PCT-NTDs and IDM-NTDs at State level | Identification of task force members, Hold meetings | Annually | Secretariat and logistic support |
| **Strategic Objective 2: Strengthen and foster partnerships for the control, elimination and eradication of targeted NTDs at national, LGAs and community levels.** | | | |
| Hold bi-annual NTD Steering Committee Meetings at National level | Sourcing for Funds; Identification of Venue; Invitation of Participants; Holding of Meeting | Twice annually | Secretariat and logistic support |
| Mobilize additional Partners at National & Zonal levels | Identification of partners; production of Invitation letters; sensitization meeting with partners | 2annually | Secretariat and logistic support |
| Inclusion of NTDs into the 17 Sustainable Development Goals (SDGs) | Meeting with global partners for NTDs to be part of post MDGs 2015 | January-June 2015 | Secretariat and logistic support |
| Strengthen collaboration with other community based health programmes like RBM, EPI, School Feeding programme | identification of other community based health programmes; sensitization visits; mobilization meetings; production of materials | 2015 – 2017 | Secretariat and logistic support; printing |
| **Strategic Objective 3: Enhance high level reviews of NTD programme performance and the use of lessons learnt to enhance advocacy, awareness and effective implementation.** | | | |
| Hold National Annual review meeting of NTDs programme managers and all stakeholders | Sourcing for Funds; Identification of Venue; Invitation of Participants; Holding of Meeting | Annually | Secretariat and logistic support |
| Coordinate Zonal & State Review Planning Meetings on NTDs | Meetings with Zonal Coordinators; identification of funding sources; holding of State & zonal reviews | Annually | Secretariat and logistic support |
| Hold State Policy Makers Meeting | Sensitization briefs production; Visits to Policy makers; holding of assessment meetings with policy makers | Annually | Secretariat and logistic support |

| Activity | Details (Sub-activities) | Timeframe/Frequency | Resources needed |
|---|---|---|---|
| **Strategic Objective 4: Strengthen advocacy, visibility and profile of NTD control elimination and eradication interventions at all levels.** | | | |
| Development and production of advocacy kits on NTDs | Holding of Meetings for development of Kits, Field-testing of kits; production & distribution of finalized copies, meeting on the production of Rabies blue print for control and elimination in Nigeria. | 2015, 2016, 2017 | Secretariat and logistic support; printing |
| Development of IEC | Holding meetings to develop IEC materials on PCT and IDMs NTDs, Pre-testing, production and distribution of materials, production of pictorial PCT MDA algorithm | 2015-2016, 2018 - 2019 | Secretariat and logistic support; printing |
| Sensitization meetings with policy makers, line ministries, and other stakeholders on the beneficial synergy of integration at the federal, state, LGAs and community levels (including Village health Committees) | Identification of key line Ministries/agencies; sensitization visits; mobilization meetings; production of materials, | Annually | Secretariat and logistic support; printing |
| Advocacy meeting with Top Management of each line ministries | Develop a power point presentation on the role and support of line ministries to NTDs, using line ministries as venues for specific NTDs meeting, invitation of line ministries to attend NTD steering committee and other NTD meeting at National and State level, report dissemination | Annually | Secretariat and logistic support; |
| Presentation of National Master Plan for ratification by steering committee, approval by HMH, and adoption by National Council on Health | Production of national Master Plan, Submission to FMOH policy makers; Presentation at National Council on Health and stakeholders dissemination meeting. | December 2015 | Secretariat and logistic support; printing |
| Sensitization meetings with Nigerian Custom Services | Production of briefs; advocacy & sensitization visits | January – December 2015 | Secretariat and logistic support |
| Conduct sensitization meetings at State and | Production of Briefs; Advocacy Meetings with NUT Executive | Annually | Secretariat and logistic support |

| Activity | Details (Sub-activities) | Timeframe/Frequency | Resources needed |
|---|---|---|---|
| LGA levels for teachers and key community leaders | Committee; Identification of suitable venues & funding sources; holding of sensitization meetings | | |
| Media advocacy and sensitization : Press briefing, talk shows on NTDs (TV & Radio) | Identification of key media organization, production of documentary briefs on NTDs, Production of Media Briefs; Talk shows; press Briefing by HMH; Press Releases; Media chats; Sensitization visits to Media Houses, airing of radio and TV jingles | Annually | Secretariat and logistic support; printing |
| Production of quarterly newsletter on NTDs | Development of Articles; Production & distribution of newsletter | Every Quarter | Secretariat and logistic support; printing |
| Advocacy to developmental partners on flexibility in the use of funds | Identification of key development partners/agencies; sensitization visits; mobilization meetings | Annually | Secretariat and logistic support |

### 3.6. MONITORING AND EVALUATION

This section is one of the most important parts of the plan. It contains information on monitoring and evaluation considerations, including the indicators to being monitored for each disease, a logical framework, and the major M&E activities to be conducted. Also included is the supervision required and the importance of baseline, impact surveys, dossiers and other evaluations; as well as the elimination process for the different NTDs. The M&E activities are allocated at least 5–10% of the total budget of the plan.

- **Monitoring** is the process of continuous observation and collection of data on the NTD programme to ensure that the programme is progressing as planned.
- **Evaluation** is the systematic and critical analysis of the adequacy, efficiency, and effectiveness of the programme and its strategies as well as progress. Evaluation refers to long, mid-term and annual analysis of a performance in relation to the goals, objectives and set targets.

The WHO African Region’s framework for NTD programme monitoring and evaluation is shown in figure 5 below.

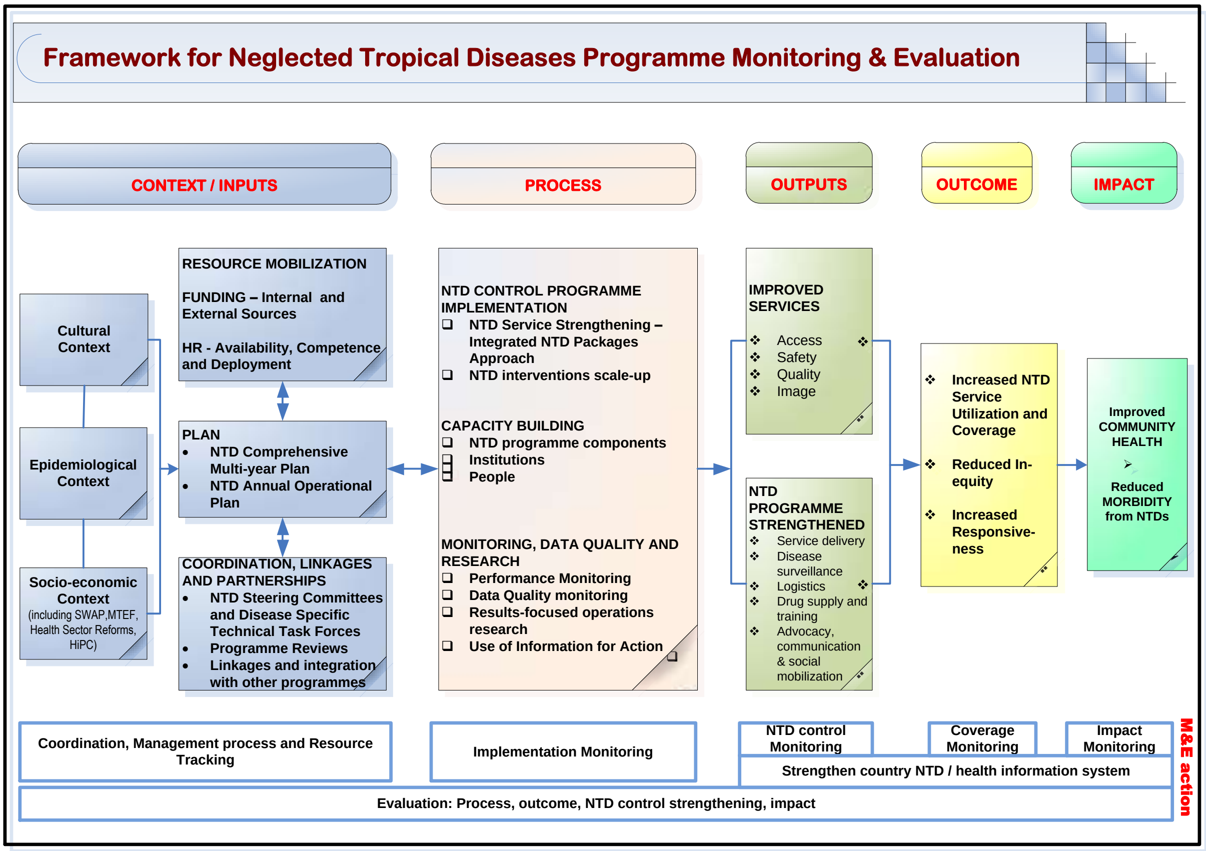


Figure 17: The WHO African Region's M&E structure for NTD

The following guiding principles have been considered in developing the M&E plan:

- Description of the existing system of M&E in Nigeria including the Health Management Information System (HMIS) and Integrated Disease Surveillance and Response (IDSR) system and how NTD information fits in.
- Description of the data flow and periodicity from the peripheral regions to the national level.
- Plan for periodic independent evaluations

In the monitoring system in Nigeria, specific programme indicators are being monitored. Outcomes of monitoring exercises are fed back into the planning process to improve implementation. FMOH has the responsibility of monitoring the adherence to the supervisory guideline already in existence. The SMOH will develop monitoring work plan that is based on FMOH POA. The LGAs will develop work plans that is based on the State work plan while the FLHF should develop a schedule for monitoring. Communities will do community self-monitoring. Retraining of the LGAs Disease Notification Officers(DSNOs) will be carried out for proper reporting of the NTDs in the IDSR reporting format.

Impact assessment studies will be conducted periodically to measure long-term effects of NTDs activities in relation to the set objectives. Outcomes of the studies will be measured against baseline data that have been collected and analyzed. The different aspects that will be measured will include ophthalmologic, entomological, parasitological, epidemiological as well as socio-economic indices.

To ensure that evaluation activities are properly conducted, the FMOH has developed a comprehensive integrated evaluation tool and guideline and generated baseline data on all interventions. These have been done to assess the impact of the interventions. Specific sentinel sites for the evaluation of impact of interventions on the NTDs have also established.
See table 19 for the specific activities and subactivities slated to be carried out.

Table 19: Strategic Priority 4: Enhance NTD monitoring and evaluation, surveillance and operational research.

| Activity | Details (sub-activities) | Time frame | Resources needed |
|---|---|---|---|
| **Strategic objective 1:Develop and promote an integrated NTD M&E framework and improve monitoring of NTDs, within the context of national health information systems**. | | | |
| Finalization of NTDs M & E Framework | Finalization of NTDs M & E Framework | 2015 | Personnel (per diem & transport), venue, Tool, Stationary, Projector, Refreshment |
| Enhance monitoring of National NTDs programme and outcomes | Conduct quarterly Integrated Monitoring visit to States, selected LGAs, health facilities and communities | Annually | Personnel (per diem & transport), Tool, Stationary |
| | Supportive supervision by state NTD Team | Annually | Personnel (per diem & transport), Tool, Stationary |
| | Supportive supervision by LGA NTD Team | Annually | Personnel (per diem & transport), Tool, Stationary |
| | Supportive supervision by Health staff | Annually | Personnel (Transport), Tool, Stationary |
| | Supportive supervision by community base supervisors and monitors | Annually | Personnel, Tool, Stationary |
| | Production of supervision reports | Annually | Personnel , Tool, Stationaries |
| Establishment of Community self Monitoring (CSM) | Training of State, LGA and HFs Workers on CSM | Annually | Personnel (per diem & transport), venue, refreshment, Printing cost, Stationaries |
| | Establishment of community self monitoring | Annually | Personnel (per diem & transport), venue, refreshment, Printing cost, Stationaries |
| | Training of community self monitors | Annually | Personnel (per diem & transport), venue, refreshment, Printing cost, Stationaries |
| | Conduct community self monitoring | Annually | Personnel |
| Monitoring of NTD activities | Production & Distribution of integrated NTDs M & E checklist to States, LGAs and H/Fs | 2015 | Personnel (per diem & transport), venue, refreshment, Printing cost, Stationaries. |
| | Production of supervisory check list by State | 2015 | Personnel , Tool, Stationaries. Printing cost. |
| | Production & Distribution of integrated NTDs data collection / reporting forms and Training Manuals to States, LGAs and H/Fs | 2015 | Personnel (per diem & transport), venue, refreshment, Printing cost, Stationary. |
| | Training of State, LGAs, HFs and community NTD implementers on | Annually | |

| | | | |
|---|---|---|---|
| | the use of data collection reporting tools | | |
| | Development of integrated NTDs monitoring tools. | 2015 | Personnel (per diem & transport), Tool, Stationaries.venue, projector, refreshment. |
| | Conduct treatment coverage survey at state, LGAs, Health facility and community | Annually | Personnel (per diem & transport), Tool, Stationaries. |
| | Adaptation and production of coverage survey tools by state | 2015 and 2017 | Personnel (per diem & transport), venue, refreshment, Printing cost, Stationaries. |
| | Conduct Integrated Coverage Survey by state | Annually | Personnel (per diem & transport), Tool, Stationeries. logistic |
| | Production of Report of coverage survey and feed back by state | Annually | Personnel , Tool, Stationeries. |
| Conduct of impact assessment studies | Conduct impact assessment for PCT NTDs (Oncho, LF, Schisto, Trachoma and STH) | 2015 -2020 | Personnel (per diem & transport), Tool, Stationeries. Logistic, consumables. |
| Conduct of impact assessment studies | Conduct Epidemiological evaluation of Oncho (Phase 1A) | 2015 -2020 | Personnel (per diem & transport), Tool, Stationeries. Logistic, consumables. |
| | Conduct Epidemiological evaluation of Oncho (Phase 1B) | 2015 -2020 | Personnel (per diem & transport), Tool, Stationeries. Logistic, consumables. |
| | Conduct Entomological evaluation of Oncho | 2015 -2020 | Personnel (per diem & transport), Tool, Stationeries. Logistic, consumables. |
| | Conduct cytotaxonomic identification of simulium of species and their distribution and migration pattern | 2015 -2020 | Personnel (per diem & transport), Tool, Stationeries. Logistic, consumables. |
| | Conduct Trachoma impact assessment survey | 2015 -2020 | Personnel (per diem & transport), Tool, Stationeries. Logistic, consumables. |
| | Conduct LF Transmission Assessment survey (TAS I & II) | 2015 -2020 | Personnel (per diem & transport), Tool, Stationeries. Logistic, consumables. |
| | Conduct impact assessment survey for Shisto / STH | 2015 -2020 | Personnel (per diem & transport), Tool, Stationeries. Logistic, consumables. |
| **Strategic objective 2: Strengthen and foster partnerships for the control, elimination and eradication of targeted NTDs at national, district and community levels** | | | |
| **Activity** | **Details (sub-activities)** | **Time frame** | **Resources needed** |
| Strengthen and foster partnerships for the control, elimination and eradication of targeted NTDs at | Mobilize additional Partners at National & Zonal levels | 2015 - 2018 | Personnel, IEC Kit, transport. |

| | | | |
|---|---|---|---|
| national, district and community levels | | | |
| Strengthen advocacy, visibility and profile of NTD control elimination and eradication interventions at all levels | Sensitization meetings with policy makers, (health related) line ministries, and other stakeholders on the beneficial synergy of integration at the Federal, State, LGAs and community levels (including Village health Committees) | Annually | Personnel, IEC Kit, transport, venue, logistic, stationary. |
| | Zonal Bi – Annual Review Meeting on M & E | 2015 - 2020 | Personnel (per diem & transport), Tool, Stationery. Logistics, Venue, Refreshment |
| | Media advocacy and sensitization : Press briefings, talk shows on NTDs (TV & Radio) | Annually | Personnel, stationary, media/jingle cost |
| | Production of quarterly newsletter on NTDs | | Personnel, printing cost |
| | Develop integrated IEC Materials on NTD PCT | 2015 - 2016 | Personnel, venue, staionaries, Logistic |
| Enhance high level reviews of NTD programme performance and the use of lessons learnt to enhance advocacy, awareness and effective implementation | Hold National Annual review and planning meetings of NTDs programme managers | Annual | Personnel, venue, staionaries, Logistic. |

**Strategic objective 3:Strengthen surveillance of NTDs and strengthen response and control of epidemic-prone NTDs, in particular dengue and Leishmaniasis**

| **Activity** | **Details (sub-activities)** | **Time frame** | **Resources needed** |
|---|---|---|---|
| Strengthen the surveillance of NTDs and strengthen the response and control of epidemic-prone NTDs, in particular for dengue and Leishmanaisis | Develop & Produce Guidelines & Forms for Management of Adverse Events | 2015 - 2016 | Personnel (per diem & transport), venue, refreshment, Printing cost, stationary. |
| | Strengthen surveillance structures & mechanisms for GW and other IDMs at community levels | 2015 - 2020 | Personnel, tools. |
| Support operational research, documentation and evidence to guide innovative approaches to NTD programme interventions | Conduct Operational Research on NTDs in the areas of Motivation for NTDs drug distributions | 2015 - 2020 | Personnel (per diem & transport) Stationary. Tools, |
| | Conduct Operational on detection of S. Mansonia in urine samples (KAP) | 2015 - 2017 | Personnel (per diem & transport) Stationary. Tools, |
| | Identify and conduct other operational research on NTDs for effective programme implementation at state, LGAs, | Annually | Personnel (per diem & transport) Stationary. Tools, |

| | | | |
|---|---|---|---|
| | Health Facility and Community | | |
| Conduct Cross-border Meetings | Cross border meetings with the neighboring countries to guide against re-infection/importation of NTDs | 2015 - 2019 | Personnel(per diem & transport) Stationary. Tools, logistic, venue |

**Strategic objective 4: Establish integrated data management systems and support impact analysis for NTD at national and State levels as part of the regional NTD data management system and regional NTD plan**

| Activity | Details (sub-activities) | Time frame | Resources needed |
|---|---|---|---|
| Establish integrated data management systems and support impact analysis for NTD at the national level, as part of the regional NTD data management system | Establishment of an integrated data management system | 2014 - 2015 | Personnel (per diem & transport) Stationary. Tools, logistic, venue |
| | Establishment of a central storage for the integrated NTDs data base | 2015 | Personnel (per diem & transport) Stationary. Tools, logistic. |
| | Collation of treatment data from register from community | 2015 - 2020 | Personnel, Stationary. Tools, |
| | Transmission of treatment data using reporting forms at lower levels | 2015 - 2020 | Personnel, Stationary. Tools. |
| | Data entry at state level | 2015 - 2020 | Personnel, Stationary. Tools. |
| | Production of periodic report at LGAs and state levels | | Personnel, Stationary. Tools |
| | Monthly validation of data at National Level | 2015 - 2020 | Personnel. |
| | Training of NTDs Data Officers on NTD Data Base Management and TIPAC | 2015 - 2016 | Personnel (per diem & transport), Tool, Stationary. Logistic. Refreshment. venue |
| | Development , Production and Dissemination of Guidelines for NTD Data reporting | 2015 | Personnel (per diem & transport), Tool, Stationary. Logistic. Refreshment. Printing cost. Venue. |
| | Conduct of data quality assessment at state, LGAs, Health Facility and community level | 2015 - 2019 | Personnel (per diem & transport), Tool, Stationary. Logistic. Refreshment. Venue. |
| | Development of data quality assessment plan at State level | 2015 - 2016 | Personnel (per diem & transport), Tool, Stationary. Logistic. Refreshment. Venue. |
| | Production of data quality assessment tool at state level | 2015 - 2016 | Personnel (per diem & transport), Tool, Stationary. Logistic. Refreshment. Printing cost. Venue. |
| | Conduct Data Quality Assessment by State | 2015 - 2019 | Personnel (per diem & transport), Tool, Stationary. Logistic. Refreshment. Venue |
| | Development and sharing of report of Data Quality Assessment by State | 2015 - 2019 | Personnel (per diem & transport), Tool, Stationary. Logistic. Refreshment. |

| | | | |
|---|---|---|---|
| | | | Printing cost. Venue. |
| Supply chain management | Training on drug inventory at state, LGAs, HFs and community levels | Annually | Personnel (per diem & transport), Tool, Stationary. |
| | Monitoring of PCT NTDs Drug Inventory at State, LGAs, Health Facility and community level | 2015 - 2019 | Personnel (per diem & transport), Tool, Stationary. |
| | Sensitization of NAFDAC for inclusion of NTDs drugs on the NAFDAC list during market raids | 2015 - 2019 | Transport, Tool, Stationary. |

## 3.7. POST INTERVENTION SURVEILLANCE AND INTEGRATION WITHIN PRIMARY HEALTH CARE

In order to successfully maintain disease levels below thresholds where they are not of public health significance following intense period of interventions; a strong post-intervention surveillance by the primary health care is, as well as their ability to incorporate the surveillance and residual control activities in routine health care delivery is necessary.

There has been some cross-border collaboration between Nigeria and neighbouring countries for NTDs management and control. Meetings have been held between Nigeria and the Republic of Benin on onchocerciasis control and also regional meetings that have focused on some or most of the NTDs. Such meetings include the Joint Action Forum (JAF) and other regional meetings of the African Programme for Onchocerciasis Control, as well as the meetings organized by the Global Alliance for the Elimination of Lymphatic Filariasis (GAELF). In these later meetings, there has been exchange of information on various aspects of NTDs mapping and management. It is planned that on-coming cross-border meetings, even if primarily for onchocerciasis control, will also be utilized to share data on NTDs and plan for cross-border collaboration.

The activities and related resource needs are included in the table below:

Table 20: Activities for surveillance and sustainability

| Activity | Details (Sub-activities) | Timeframe | Resources needed |
|---|---|---|---|
| ***Strategic Objective :*** | | | |
| Strengthen the surveillance of NTDs and strengthen the response and control of epidemic-prone NTDs, in particular for dengue and Leishmaniasis | Strengthen surveillance structures & mechanisms for GW and other IDMs (BU, Leprosy, HAT, Rabies Leish) at community level. | 2015 - 2020 | Personnel (per diem & transport), Tool, Stationary. Logistic. Refreshment. Printing cost. Venue. |
| | Capacity building for NPHCDA personnel at all levels on NTDs for surveillance structures and activities | 2015 - 2020 | Personnel (per diem & transport), Tool, Stationary. Logistic. Refreshment. Printing cost. Venue. |

Table 21.1: Summary Budget for federal level activities only

| Key Activities | 2015 | 2016 | 2017 | 2018 | 2019 | 2020 | *Total* |
|---|---|---|---|---|---|---|---|
| Mapping | 294,847,470 | - | - | - | 294,847,470 | 235,877,976 | ***825,572,916*** |
| MDA | 2,400,000 | 2,564,693 | 2,732,636 | 2,838,945 | 2,960,993 | 2,368,794 | ***15,866,061*** |
| Capacity Building | 45,250,000 | 39,365,059 | 41,313,215 | 34,728,750 | 4,278,582 | 3,422,866 | ***168,358,472*** |
| Case Management | - | - | - | - | - | - | ***-*** |
| Vector/env mgt | - | - | - | - | 20,508,021 | 16,406,417 | ***36,914,439*** |
| Coordination | 33,163,000 | 34,892,380 | 36,700,283 | 4,524,569 | 4,801,540 | 3,841,232 | ***117,923,003*** |
| Partnerships | 2,961,400 | 3,164,617 | 2,620,370 | 2,722,312 | 2,888,958 | 2,311,166 | ***16,668,823*** |
| Review Meetings | 55,304,500 | 59,099,611 | 62,969,606 | 65,419,348 | 69,423,990 | 55,539,192 | ***367,756,247*** |
| Advocacy | 21,525,000 | 22,285,320 | 18,021,113 | 18,269,890 | 19,732,704 | 15,786,163 | ***115,620,191*** |
| Planning | 9,558,400 | 10,214,317 | 10,883,177 | 11,306,572 | 11,998,703 | 9,598,962 | ***63,560,132*** |
| Resource Mob | 195,000 | 208,381 | - | - | - | - | ***403,381*** |
| Review of Policies | 19,544,800 | - | 10,610,597 | - | 2,445,333 | 1,956,267 | ***34,556,997*** |
| Mon/Super and Eval | 486,650,260 | 518,518,594 | 559,397,395 | 577,045,900 | 432,579,535 | 346,063,628 | ***2,920,255,313*** |
| Surveillance/response | 17,771,000 | 18,662,250 | 19,597,762 | 20,575,818 | 21,603,406 | 17,282,725 | ***115,492,961*** |
| Operational research | 19,233,400 | 20,195,070 | 21,204,824 | 22,265,065 | 23,378,318 | 18,702,654 | ***124,979,330*** |
| Data management | 22,481,000 | 2,076,900 | 25,161,879 | 2,289,782 | 15,499,608 | 12,399,687 | ***79,908,857*** |
| Establishment of Nat. Ref.Lab. | - | 105,000,000 | - | - | - | - | ***105,000,000*** |
| **Totals** | **1,030,885,230** | **836,247,193** | **811,212,856** | **761,986,951** | **926,947,163** | **741,557,731** | **5,108,837,124** |

Table 21.2: Summary Budget for National Activities

| State | Coordination, Partnership & Advocacy | Planning and Resource Mobilization | Scale-up Interventions | M&E, Research | Total by State |
|---|---|---|---|---|---|
| Abia | 153,948,670 | 61,536,566 | 445,186,230 | 185,571,200 | ***846,242,666*** |
| Adamawa | 231,104,970 | 89,494,051 | 790,661,576 | 281,725,000 | ***1,392,985,597*** |
| Akwa ibom | 209,347,309 | 69,361,047 | 548,038,098 | 203,654,600 | ***1,030,401,054*** |
| Anambra | 208,741,245 | 59,784,989 | 461,398,770 | 188,545,600 | ***918,470,604*** |
| Bauchi | 171,251,461 | 57,975,201 | 640,134,053 | 217,208,800 | ***1,086,569,516*** |
| Bayelsa | 153,485,394 | 49,094,071 | 473,026,857 | 179,683,200 | ***855,289,522*** |
| Benue | 179,050,579 | 77,437,273 | 532,383,000 | 219,620,600 | ***1,008,491,452*** |
| Borno | 105,518,583 | 41,034,068 | 822,973,506 | 230,439,000 | ***1,199,965,156*** |
| Cross river | 195,959,433 | 63,933,296 | 500,379,595 | 175,939,800 | ***936,212,125*** |
| Delta | 109,631,264 | 33,840,148 | 431,319,438 | 153,599,800 | ***728,390,650*** |
| Ebonyi | 159,243,538 | 59,506,766 | 477,449,086 | 152,725,800 | ***848,925,190*** |
| Edo | 195,959,433 | 63,933,296 | 500,154,595 | 184,549,800 | ***944,597,125*** |
| Ekiti | 136,716,598 | 46,341,342 | 449,455,541 | 157,659,800 | ***790,173,282*** |
| Enugu | 170,260,651 | 56,767,059 | 523,222,171 | 219,405,800 | ***969,655,681*** |
| FCT | 105,755,517 | 40,401,436 | 329,803,085 | 156,369,600 | ***632,329,638*** |
| Gombe | 175,666,486 | 43,657,452 | 805,240,315 | 255,033,800 | ***1,279,598,053*** |
| Imo | 184,766,139 | 80,556,331 | 587,530,975 | 246,734,000 | ***1,099,587,444*** |
| Jigawa | 149,080,584 | 64,719,906 | 504,064,879 | 178,318,680 | ***896,184,050*** |
| Kaduna | 140,995,523 | 49,017,515 | 636,646,335 | 201,511,000 | ***1,028,170,374*** |
| Kano | 177,578,432 | 60,386,024 | 1,072,886,676 | 343,768,000 | ***1,654,619,132*** |
| Katsina | 164,458,540 | 71,278,730 | 522,945,771 | 161,621,680 | ***920,304,720*** |
| Kebbi | 138,002,433 | 58,240,024 | 523,018,367 | 137,923,800 | ***857,184,625*** |
| Kogi | 147,386,082 | 40,495,731 | 558,125,413 | 141,050,000 | ***887,057,226*** |
| Kwara | 125,725,555 | 46,614,260 | 477,128,960 | 172,038,800 | ***821,507,575*** |
| Lagos | 119,190,874 | 58,140,142 | 467,682,258 | 189,932,100 | ***834,945,374*** |
| Nasarawa | 137,043,653 | 51,589,201 | 526,042,008 | 183,418,800 | ***898,093,663*** |
| Niger | 166,506,583 | 50,907,224 | 998,150,901 | 284,990,800 | ***1,500,555,508*** |
| Ogun | 141,706,705 | 58,010,181 | 633,974,543 | 214,032,800 | ***1,047,724,230*** |
| Ondo | 138,314,333 | 31,501,187 | 492,323,514 | 165,034,600 | ***827,173,635*** |
| Osun | 236,964,463 | 54,977,047 | 538,139,698 | 202,204,600 | ***1,032,285,808*** |
| Oyo | 190,608,852 | 73,035,488 | 524,185,692 | 201,009,200 | ***988,839,233*** |
| Plateau | 166,366,173 | 55,356,142 | 615,890,090 | 207,504,160 | ***1,045,116,565*** |
| Rivers | 161,265,932 | 51,400,084 | 412,171,268 | 188,638,200 | ***813,475,484*** |
| Sokoto | 160,614,941 | 51,681,710 | 617,641,168 | 194,599,000 | ***1,024,536,819*** |
| Taraba | 135,883,465 | 54,233,201 | 564,571,279 | 199,982,800 | ***954,670,745*** |
| Yobe | 170,476,687 | 55,356,142 | 649,797,065 | 215,120,800 | ***1,090,750,694*** |
| Zamfara | 100,330,022 | 49,042,201 | 392,104,931 | 138,692,200 | ***680,169,353*** |
| National HQ | 652,525,261 | 63,963,513 | 1,046,711,888 | 3,345,636,461 | ***5,108,837,124*** |
| **Totals** | **6,567,432,364** | **2,144,600,047** | **22,092,559,596** | **10,675,494,681** | ***41,480,086,689*** |
