# Supplementary material for: Optimising the performance of frontline implementers engaged in the NTD programme in Nigeria: lessons for strengthening community health systems for universal health coverage
Source: Hum Resour Health. 2019 Nov 1;17:79. doi: 10.1186/s12960-019-0419-8 (PMC6824027; doi:10.1186/s12960-019-0419-8)
Supplement: Supplementary file 2 — Additional file 2. Standard Operating procedure for Supply chain management for NTD medicine [file 12960_2019_419_MOESM2_ESM.pdf]

Contents

Foreword ..... Error! Bookmark not defined.

Preface ..... Error! Bookmark not defined.

Acknowledgement ..... Error! Bookmark not defined.

Acronyms 3

1. INTRODUCTION 5

1.1 Purpose of this Manual 5

1.2 Users of the Manual 5

2. OVERVIEW OF THE NEGLECTED TROPICAL DISEASES (NTDs) 6

2.1 Background 6

2.2 Programme implementation structure 6

2.3 Logistics system for medicines 7

3. MEDICINE FORECAST AND REQUISITION PROCESS 8

3.1 Medicine Forecast and Quantification Process 8

3.2 Medicine Requisition Process 9

3.3 Custom clearance and documentation 10

4 STORAGE AND DISTRIBUTION 13

4.1 Visual Inspection 14

4.2 Guidelines for storage of medicines 15

4.3 General Storage Guidelines 16

Good Storage Tasks 17

4.4 Handling Expired/Damaged Medicines 17

4.5 Conducting a physical Inventory 18

4.7 Temperature Control 18

4.8 Guidelines for transportation medicines 18

4.9 Guidelines on drug handling at community level 25

5. INVENTORY MANAGEMENT 26

5.1 Logistics Management Information System 26

5.2 Purpose of LMIS 27

5.3 The Six Rights of LMIS data 27
5.4 Essential features of NTDs LMIS 27
5.5 Logistics Records 28
5.6 Logistics Reports 29
5.7 Logistics Management Information Tools (LMIS) 29
5.8. Job Aids for Logistics Management Information Tools 30
5.9 Completing the Stock Allocation Issue and Received Voucher 30
5.10 Completing the Stock Ledger 37
5.11 Completing the Medicines Returns and Transfer Forms 41
5.12 Completing the Quarterly Stock Status Report 44
5.12 Completing the Stock Expiry Date Tracker: 48
5.13 Feedback Reports 53
6. REVERSE LOGISTICS AND WASTE MANAGEMENT 53
6.1 Reverse logistics of NTDs Medicines 53
6.2 Wastes Management of NTDs Medicines 55

Acronyms

ALB	Albendazole
AZT	Zithromax
CDD	Community Directed Distributors
CI	Community Implementer
FCMS	Federal Central Medical Stores
FEFO	First to Expire First Out
FIFO	First In First Out
FLHF	Front Line Health Facilities
FMOH	Federal Ministry of Health
IVM	Ivermectin
JRSM	Joint Request for Selected PC Medicines
LF	Lymphatic Filariasis
LGA	Local Government Area
LMIS	Logistics Management Information System
MBD	Mebendazole
NGDOs	Non-Governmental Development Organizations
NTD	Neglected Tropical Diseases
Oncho	Onchocerciasis
POS	Paediatric Oral Suspension
PZQ	Praziquantel
RL	Reverse Logistics
Schisto	Schistosomiasis
SCMS	States Central Medical Store
SMOH	State Ministry of Health
SOH	Stock on Hand

SOP	Standard Operating Procedures
STH	Soil Transmitted Helmiths
Tab	Tablet
TEO	Tetracycline Eye Ointment
TIPAC	The Integrated tool for Planning and Costing

1. INTRODUCTION

In many states in Nigeria, the supply chain for Neglected Tropical Diseases Drugs (NTDD) is non-uniform, fragmented and characterized inappropriate storage practices, poor inventory management practices, late reporting and weak reverse logistics system. A weak human resources capacity for supply chain management and absence of standard operating procedures to guide and clarify roles and responsibilities further create operational challenges.

At various fora, stakeholders in the NTDs have expressed the need for a standard operating procedure manual for NTD supply chain management to provide guidance to health workers and program managers in addition to serving as a reference training material, monitoring and support supervision.

1.1 Purpose of this Manual

In view of the strategic role of supply chain in the achievement of NTD Program targets, a Standard Operating Procedures (SOPs) manual provides guidance on ensuring product availability, safety and accountability in addition to ensuring that uniform standards are maintained in the performance of specific tasks which in turn will lead unmistakably to the achievement of the set objectives.

This manual contains a set of SOPs for the management of NTD Medicines and associated commodities from the upstream supply chain (Federal Medical Stores) down to the last mile (communities and schools) where the medicines are required.

1.2 Users of the Manual

This manual is intended to be used by personnel that carry out logistics and programmatic functions relating to NTD Program- from the Federal Central Medical Stores down to the service delivery points (communities and schools) . The following categories of personnel will find this manual extremely useful:

- All personnel and program management staff with logistics and inventory management responsibilities at Federal, State, LGA, Front Line Health Facilities, Community level
- NTD Program Managers and Zonal Coordinators
- State NTD Coordinators
- Local Government Area (LGA) NTD Focal Persons
- Logistics and Store Officers
- Pharmacists
- Community Implementers
- Teachers

2. OVERVIEW OF THE NEGLECTED TROPICAL DISEASES (NTDs)

2.1 Background

Neglected Tropical Diseases (NTDs) are diseases that affect rural poor dwellers of low-income communities where safe water supply, sanitation and hygiene are poor or even totally lacking. Although safe and cost-effective interventions for prevention and control are available, these diseases have continued to cause immense suffering and often life-long disabilities for the affected persons due to neglect, with impact much more than that of HIV/AIDs, Tuberculosis and Malaria put together globally.

Priority NTDs for Nigeria include Onchocerciasis (River blindness), Lymphatic Filariasis (LF (Elephantiasis)), Schistosomiasis (SCH), Soil Transmitted Helminthiasis (STH) and Trachoma - the PCT NTDs (Preventive Chemotherapy NTDs); and Human African Trypanosomiasis (HAT), Buruli Ulcer (BU), Leprosy (LEP) and Guinea worm Disease (GWD) – IDM NTDs (Innovative Disease Management NTDs). There are also the Zoonotic NTDs in Nigeria such as Rabies.

About one billion people worldwide are affected by one or more of these diseases, which are common in areas with poor housing, water supply and sanitation. Nigeria contributes significantly to this global burden.

NTDs contribute to and are perpetuated by poverty, and have gradually disappeared from areas where there is significant improvement in quality of life. In Nigeria where the social indices for health are still not optimal, these diseases continue to thrive.

Medicines used for all PC NTDs chemotherapy are donated by partners, with over 255 million persons that need to swallow these medicines annually nationwide. The NTDs Programme get these donated medicines to the end users in the communities and implement other interventions in order to achieve the global target of elimination of the NTDs by 2020

2.2 Programme implementation structure

The programme implementation is organized along the 3 tiers of government with the units of operation being the health facilities at the local government area (LGA). At the Federal level, there is a Central unit headed by the National Coordinator who is the Programme Manager. The National Coordinator is assisted in her work by professionals in charge of different aspects of the program implementation.

The National Coordinator is thus the head of technical crew at the federal level. A similar structure exits at the state and LGA levels with programme manager known as the State NTD Coordinator and the LGA NTD Coordinator respectively. The line of technical supervision goes from the NC through the State NTD Coordinator and to the LGA NTD Coordinator. The line of administrative authority is as laid down by the different tiers of government.

2.3 Logistics system for medicines

The success of the NTD program rest mainly in the provision of essential medicines, recording and reporting materials which include forms, cards, books, registers etc.) on a regular basis. A reliable system of logistics support is therefore a sine qua non to effective program implementation.

This section describes the flow of medicines and information from Federal Central Medical Stores (FCMS) to State, LGA and facility stores as shown in Figure 1 below. Medicines come into the country from donors/ manufacturers through the sea or airports and are moved to be stored at the Federal Central Medical Stores. The in-country pipeline has five supply levels namely: FCMS, State Stores, LGA stores, the health facilities and the community level. The pipeline is designed to be visible from any point in the system i.e. commodity stock level information and reports are to be accessible and visible from the primary level facilities upward.

NTDs MEDICINES & INFORMATION FLOW

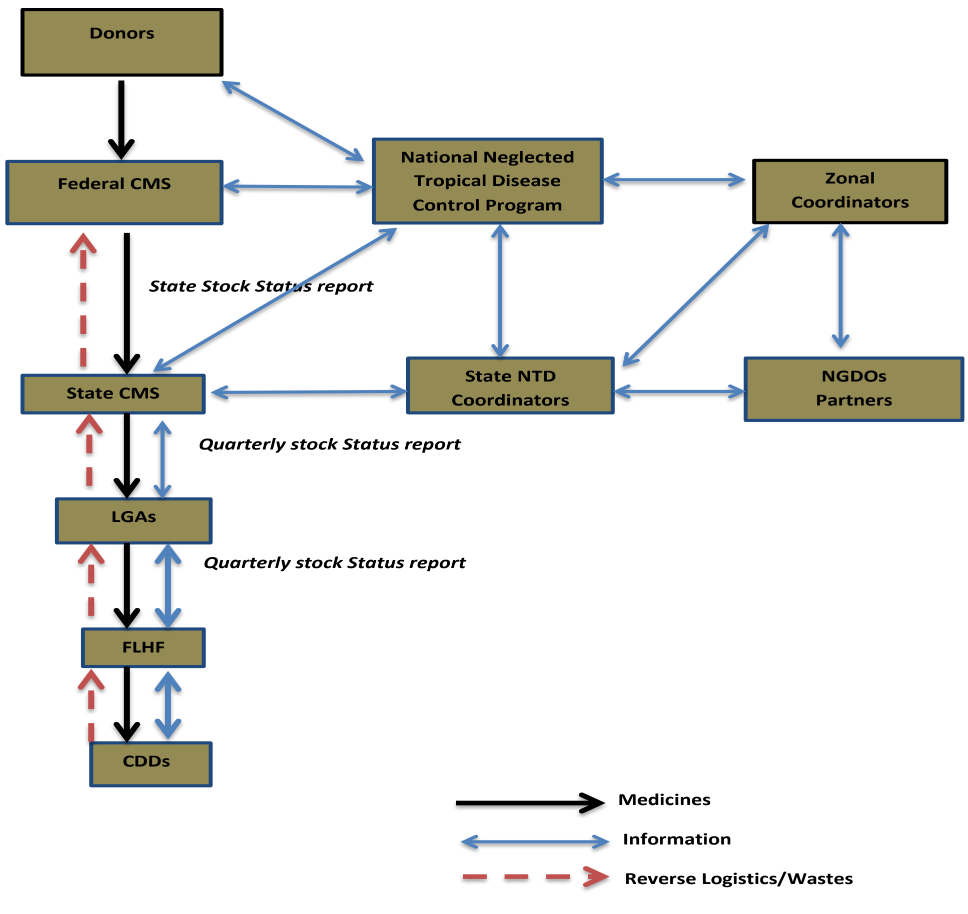

In the forward logistics, the distribution of medicines from one level to the other are captured and documented using transaction records signed by multiple partners thus ensuring accountability at each level. At the end of the Mass Administration of Medicines (MAM), a system of reverse logistics is implemented in which remaining balance of medicines or wastes are returned upwards from community/ schools to Front Line Health facilities to LGA stores and finally to State CMS. The flow of commodity (medicines) and information represent a necessary component of the NTD supply chain.

The products covered on the NTD programme include:

i. Mectizan (IVM)
ii. Albendazole (ALB)
iii. Praziquantel (PZQ)
iv. Mebendazole (MBD)
v. Azithromycin (AZT) (Tabs/POS)
vi. Tetracycline eye ointment (TEO)

3. MEDICINE FORECAST AND REQUISITION PROCESS

This section describes the process of determining the quantities of medicines required, the process for requesting from donors and eventual delivery of the medicines into the country designated medical storage.

3.1 Medicine Forecast and Quantification Process

Quantification is the process of estimating the quantities and costs of the products required for a specific health program (or service), and determining when the products should be delivered to ensure an uninterrupted supply for the program.

The PC NTDs Mass Administration of Medicine programmes use five different medicines: Ivermectin, Albendazole, Praziquantel, Mebendazole and Zithromax®.

- Mectizan for Onchocersiasis
- Mectizan and Albendazole for Lymphatic Filariasis
- Praziquantel for Schistosomiasis
- Albendazole or Mebendazole for Soil Transmitted Helminths
- Zithromax® (tablet& Syrup) and Tetracycline Eye Ointment (TEO) for Trachoma

Forecast and Quantification Process:

- Medicines for the PC NTDs are quantified based on the eligible population in the endemic/target LGAs while the number of cases is considered in drug quantification for IDM NTDs.

- Medicines for Oncho, LF, Schisto and STH (Ivermectin, Albendazole, Praziquantel, Mebendazole) are applied for jointly by the NTDs M & E unit using The Integrated tool

for Planning and Costing (TIPAC). Joint re-application is forwarded by 15th August of every year.

- There is a separate template for Zithromax requisition which is completed and forwarded before the end of February, every year.

- Joint Request for Selected PC Medicines (JRSM) (Ivermectin, Albendazole, Praziquantel, Mebendazole) is generated by TIPAC.

- The Multiplier (average per person) used to generate the total quantity needed for each PC NTD medicine is as below:

  - Ivermectin – 2.8 tablets x 80% of total population of LGA for LF
  - Ivermectin- 2.8 tablets x total population of Oncho-endemic communities for Oncho
  - Albendazole – 1 tablet x 80% of total population of LGA for LF
  - Albendazole- 1 tablet x 100% of target population for STH
  - Mebendazole – 1 tablet X 100% of target population for STH
  - Praziquantel – School Age Children -- 2.5 tablets x target population (28% of total population)
  - Praziquantel – Adult – 3 tablets x 52% of total population of the LGA
  - Zithromax (tablet)– 3 tablets x 80% of target population
  - Zithromax (POS) – 0.333 bottle x 18% of target population
  - Tetracycline Eye Ointment – 2 tubesx 2% of target population

- The multipliers above are multiplied by the target population of each intervention to arrive at the total quantity needed for each medicine.

3.2 Medicine Requisition Process

The process for requesting for NTD medicines are stated below:

- At the end of the treatment cycle estimated balance of each medicine together with Joint Request for Selected PC Medicines (JRSM) is sent to the donors through WHO Nigeria.

- Joint Request for Selected PC Medicines is reviewed by different donors

- Approval is sent by donor for each PC medicine for all endemic States/NGDOs

- In the approval, estimated balance of each medicine at the end of the treatment cycle is deducted from the quantity required.

- UNICEF, WHO and other partners assist in clearing NTD medicines through the clearing agents on arrival at the Port,

- Clearing documents including duty waiver are processed and obtained by the consignee (WHO and UNICEF) from Nigeria Customs Service and Federal Ministry of Finance.

- Before shipment, shipping documents are sent to WHO and UNICEF as the case may be while relevant Programmes are copied.
- PC medicines are shipped by the donors after production
- Prior arrival of PC Medicines at the Port, the Clearing agent is required to submit the following documents to the Federal Central Medical Stores (FCMS) prior delivery:
  - Notification with a letter of intent 72 hours before delivery
  - Certificate of analysis from the donor/Clearing Agent
  - Packing list
  - Way bill
  - Proforma invoice
- PC NTD Medicines on arrival at the Port are cleared by the clearing agents.
- Subsequently, all consignments of Ivermectin, Albendazole, Praziquantel, Mebendazole and Azithromycin for the NTDs Programmes are supplied and effectively managed at the Federal Central Medical Stores (FCMS), Oshodi-Lagos.
- Approved Distribution Plan showing in details the allocations in terms of quantity to the benefiting States is sent by NTDs division, FMOH to the FCMS prior to distribution.
- Allocation Letter is sent by NTDs division, FMOH advising States to collect the Medicines from the Central warehouse.
- State CMS receives the medicines from Federal CMS
- The LGAs collect their PC NTDs medicines from the State CMS while the Front Line Health Facilities collect from the LGAs and issue to the various communities under them.

3.3 Custom clearance and documentation

This section describes the process for the clearance of consignment through customs and ensuring necessary document required for accountability and compliance are met.

Aim:

- The SOPs explains the policies and defines the activities and responsible staff in carrying out the custom clearance of a consignment.
- Ensure that agreed customs clearance policies and procedures are implemented as laid down
- Activities are performed in an orderly efficient and repetitive manner.
- Minimize errors during the execution of this activity
- Serve as training guide for new staff as well as refresher guide for existing staff
- Be the reference guide to carry out an activity in case of doubt or difference of opinion.

Objective:

- Ensure that the custom clearance activities are done accurately and consistently so as to eliminate loss.
- Expediting consignment clearance.
- To carry out the custom clearance operations efficiently in accordance with set out guidelines in order to ensure adequate safety of all stocks.
- Custom clearance operations are done in line with the security management policy and procedures to ensure safety of stock, personnel, and equipment.
- To facilitate processing of orders / deliveries efficiently in line with the set inventory management, receipt, dispatch and distribution policy guidelines.

Procedure Scope:

These SOPs relate to operations at the port of entry, into Nigeria to delivery of the consignment to the warehouse. The outlined procedures should be read and implemented within the framework of the several, laws governing importation, guideline documents, and forms appended which forms part and parcel of these procedures.

Key management issues:

Importation and customs clearance is a specialized area of work. A specialized clearing and forwarding agent shall be contracted. Time periods allowed for each activity must be adhered to. Penalties for poor performance must be instituted. A strict control on subletting of services to other agents other than the one contracted must also be in place.

TheProcess

The major activities in this process are;

- Notifying stake holders of the expected consignments
- Locating the shipment at all stages
- Obtaining all necessary documents relating to the consignment on time
- Timely clearing of the consignment
- Delivery of the consignment to the warehouse

Pro-forma Invoice and certificate of Quality

Follow up to ensure that:

- The shipper (Drug Company and its agents) sends a pro-forma invoice to consignee on time.

- The pro-forma invoice should indicate the product items and their values.
- That the certificate of quality comes along with the product.

Clearing Agent

Follow up the activities of the clearing agent to ensure that they collect the all relevant documents on time, and pay the relevant handling fees.

NAFDAC:

Alert the NAFDAC inspectors of the arrival of the consignments for their timely clearance of the drugs. NAFDAC collects samples for analyses. NAFDAC releases analysis results and releases the goods promptly

Goods released and transported to designate warehouse.

Clearing Agents arrange for the transport to the designated destination of the consignment.

Delivery / Handover to Federal CMS

- Clearing Agents organize all necessary documentation for proper hand over of consignment at Federal CMS. Ensure proper reconciliation of physical presence of items as showed on the packing list before hand over and subsequent signing.
- Open cartons for verification if in doubt.

Required Documents

**Document**	**Source**
Pro-forma Invoice	Shipper + Drug manufacturer
Certificate of Quality	Supplier
Form ‘M’	Opened by consignee’s bank (optional)
CRI	Inspection agent at country of origin
Bill of entry	Prepared by consignee’s clearing agent
Airway bill	Handling company
Clearance Certificate	NAFDAC
Packing List	Supplier-with details of each package
Handover a consignment	Clearing Agents
Delivery note	Clearing Agents

4 STORAGE AND DISTRIBUTION

This section describes the process for receiving and storage of medicines at Central Medical Store. The NTDs Program have adopted the **integrated warehousing model** where all commodities are warehoused in government storage facility (State CMS and government-owned Front Line Health Facility) that are employed in storing other health commodities. This is with a view to strengthen the existing storage infrastructure, avoid parallel storage thus improving product visibility and accountability. It is also expected that ownership and accountability will be improved using the integrated warehousing approach.

The process includes the following steps:

i. National NTDs Secretariat/FMOH informs the Federal Central Medical Store about the NTDs medicines to create space for incoming medicine
ii. Delivery of document related to medicines (certificate of analysis, way bills, invoice etc)
iii. Visual inspection by FCMS staff
iv. Stock taking/Inventory
v. Storage of medicines
vi. National NTDsSecretariat sends distribution plan to FCMS
vii. Allocation letters are issued to states by National NTDsSecretariat
viii. Scheduled collection of medicines by States
ix. State CMS store the medicines collected from FCMS
x. Medicine distribution plan is developed by the State NTD Coordinator for each LGAs, FLHF and Communities (Allocation should be done to the last miles based on the available medicines in the State CMS)
xi. LGAs schedule collection of medicines from State CMS or direct delivery by State CMS to each LGA
xii. LGAs keep the medicines collected from State CMS in Local Government store
xiii. Medicines distributed to Front Line Health Facilities (FLHFs) based on distribution plan developed by the NTDs State Coordinator
xiv. FLHF staff distribute medicines to the communities based on distribution plan developed by the NTDs State Coordinator
xv. Copies of delivery notes (Allocation, Issue and Receipts Vouchers) are maintained at State CMS, LGA Stores, FLHF stores

NTDs Storage and Distribution Flow Chart

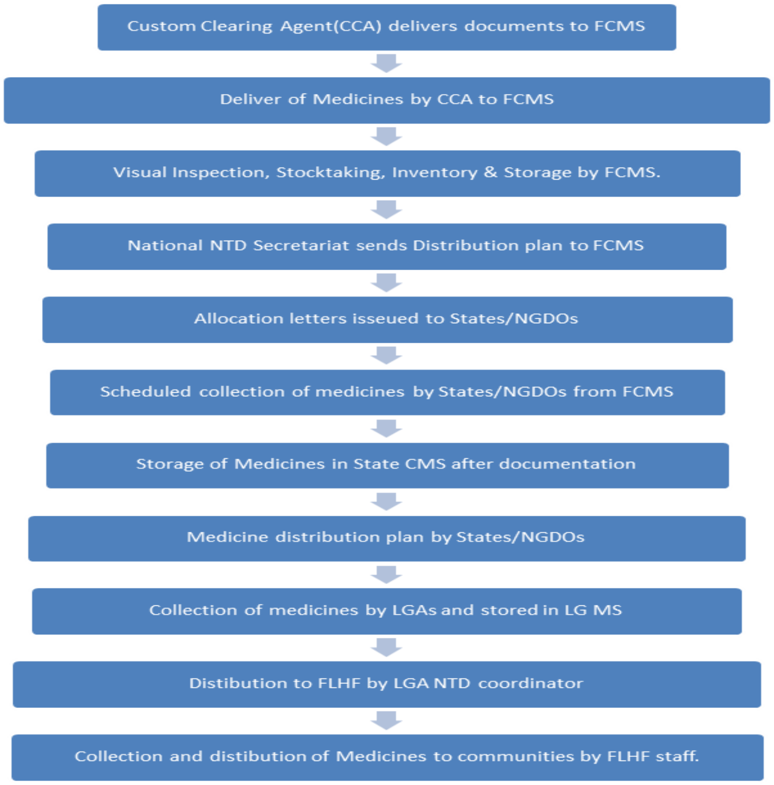

4.1 Visual Inspection

The medicines provided by the various donors to combat neglected tropical diseases must meet quality standards. The quality of the medicines must be maintained throughout the supply pipeline. Visual inspection at all levels is essential.

Upon arrival of shipment at the Central medical store, a thorough inspection must be carried out. This includes matching what is physically received with accompanied document (waybill or delivery note) to ensure that the products that have been shipped match with what is listed on the shipping receipt. When there is more than one product batch in the shipment, it is necessary to crosscheck each batch numbers on the documents to what was received. It is also important to carry out random spot checks to ensure that products are in good condition, labeled correctly, and not expired. If something looks questionable, an investigation is advised. Do not accept any damaged, unlabeled or expired products and make a note on the receipt returned with the driver about what you saw on inspection.

I. How to do a visual inspection

Each level in the supply pipeline must do the following when medicines are received, handled or counted:

1. **Look for visual damage.** Examples include broken boxes, water damage, or oil stains. Are the medicines themselves in good condition? Do they look and smell normal? Visual evidence of damage usually means that at least some of the medicines in a container are no longer usable and should not be given to people.

2. **Check if the medicines are clearly labeled.** If the labels are not legible or are missing, there is a potential danger. If the outer labels have been ripped or smudged during shipping, look at the labels on the containers inside the carton to see if those labels are clear. If there are no labels, or if they are also damaged, you cannot know what is in the container and you cannot administer the medicine to people. Unlabeled medicine should be returned to the sender.

3. **See if any medicines are expired.** The boxes or containers are typically marked with the abbreviation **EXP** (for expiration) followed by a date.For example, **EXP 12/15** would mean that the medicines are good until the twelfth month of 2015. Do not confuse **EXP** with the abbreviation **MFG**, which refers to the date the product was manufactured.

4. **Separate (or "quarantine") unusable damaged or expired medicines.** It is your responsibility NOT to give damaged or expired medicines to people. Do not just "throw them away." Children could find them or they could be sold on the black market. For our purposes, the term *quarantine* means to set aside and not distribute.

5. **You must separate expired medicines from usable medicines.** Clearly mark unusable medicines as expired or damaged and quarantine them immediately. The expired medicines should be returned to the Central Medical Stores so that they can be destroyed.

4.2 Guidelines for storage of medicines

The primary purpose of storage is to ensure stock security and the maintenance of quality of products and packaging throughout the shelf life. Proper store organization is equally important, it helps to conserve storage space and manage the time spent on the following store operations namely: receiving, issuing, physical Inventory and visual inspection. Poor storage conditions usually affect the quality of products stored.

Such poor storage conditions include high temperature, high humidity or wet environments. If medicines are not properly stored, there is the likelihood that they may lose potency even before

their stated shelf lives. A well-organized storeroom also allows proper arrangement of products for easy access and retrieval.

In summary, good storage practices save time, storage space and prevent patients from getting expired or damaged products that could be harmful.

4.3 General Storage Guidelines

Drugs should be protected from sun, heat, light and water. Manufacturer's recommendations / instructions for storing of commodities must be followed. These are usually printed on the product carton and boxes. Good storage practices save time, storage space, preserve quality of drugs and prevent waste.

The following are good storage guidelines:

- Keep the store room regularly cleaned and tidy.(sweep floors, dust the shelve).
- Devise a cleaning schedule giving specific tasks to individuals.
- Ensure there is constant availability of power supply.
- Make sure your store is dry, well-lit and well-ventilated.
- Stock cartons on pallets at least 10 cm (4 inches) off the floor, 30 cm (1 foot) away from the walls and other stacks, and no more than 2.5 meters (8 feet) high.
- Arrange cartons on shelves so that arrows point up. Identification labels and expiry dates should be visible.
- Store products in a manner that facilitates "First Expiry, First Out" (FEFO) counting and general stock management (FEFO).
- Separate externally used from internally used drugs.
- Keep drugs secure at all times.
- Separate damaged or expired drugs without delay and dispose them off according to laid down procedures.
- Inspect the physical structure of the store regularly.
- Control the temperature in the store by using the air conditioners effectively.
- Maintain cold storage for drugs requiring cold chain handling.
- Use thermometers to monitor temperatures and maintain temperature charts hanged next to the thermometer.
- Do not store commodities in direct sunlight.
- Control the light in the store– if light enters through the windows; block direct light by painting the windows white or using curtains.
- Control humidity and prevent water from reaching stored drugs.

- Ensure good drainage channels exist around the store, and no water leakage through the walls, roof or floor.
- Repair leakages as soon as they occur to reduce moisture and water infiltration.
- Containers for some powder, tablet or capsule drugs may be packed in sachets of desiccant. Keep these sachets inside the containers and keep the container closed except when withdrawing the contents for issuing.
- Keep Drugs away from insecticides, hazardous materials, old files, office supplies and equipment.
- Spilled items attract pests. Clean up spills and broken containers immediately.
- Disinfect storeroom regularly and use pesticides to get rid of pests.

Good Storage Tasks

a. Daily and weekly:

- Monitor storage conditions and record the temperature 2 times a day
- Store the drugs off the floor
- Arrange pallets and racks to allow for easy handling and cleaning.
- Monitor store security and safety by proper locking and key management to prevent theft.
- Ensure adequate ventilation and cooling.
- Update stock records and maintain files.
- If cycle counting, conduct physical inventory and update stock keeping records
- Run generator to ensure the system is working correctly; check the level of fuel and add fuel, if needed.

b. Monthly:

- Conduct physical inventory or cycle count, and update stock keeping records.
- Check for signs of rodents, insects, or roof leaks and take corrective action.
- Inspect the infrastructure for damage, including the walls, floors, roof, windows, and doors

4.4 Handling Expired/Damaged Medicines

a) Separate and move all expired or damaged medicines to the space reserved for them and record on the stock card

b) Make entries into the form for returning/transferring medicines (ensure that reference number is inserted)

c) Send the expired/damaged medicines to the next higher level using the returning/transfer form at the end of the quarter or at the point of taking delivery of stock (whichever is applicable)

d) Superior levels will receive and document all expired/damaged medicines and forward same to higher level if applicable

e) The Federal medical store will ensure the collection of all expired/damaged medicines in the pipeline at an appropriate place and time, and arrange for their destruction following due process.

4.5 **Conducting a physical Inventory**

a. All officers having direct custody of medicines shall conduct periodic physical stock counts of medicines in their store.
b. Stock counts at the health facilities and the various stores should be undertaken quarterly.
c. Expired or damaged stocks are removed following the standard procedure (see standard procedures for handling expired/damaged medicines).
d. Usable stock should be counted according to units of issue.
e. Update stock cards by writing date of physical inventory and the words "physical Inventory" in the column for remark.
f. Using a different color ink (red), write the quantity of the product that you counted during stock taking.
g. Discrepancies observed after the physical count exercise should be captured and documented as losses and adjustment in the stock cards.

4.7 Temperature Control

Temperature control is a mandatory exercise at all levels of storage and distribution to ensure that the integrity of NTDs medicines are protected. The temperature of most NTDs medicines at all levels is fixed at a maximum of +24C to ensure proper storage. However, for vaccines, *cold chain* is maintained at all levels.

4.8 Guidelines for transportation medicines

This procedure is required for the movement of NTD medicines and materials from one level to the other, in order to make it available to the end users, in good condition.

The transport and distribution process borders on the availability of PC NTDs medicines and materials, and the process involved in their distribution from the federal, to the state, to the LGA, to the FLHF down to the community levels.

The specification for the vehicle to be used for distribution of NTD medicines at all levels are as follow:

i. Containerized and enclosed vehicle/van
ii. Current driver's license
iii. Current and complete vehicle documents

iv. *Goods on transit* insurance
v. *Vehicle Certification of road worthiness*

The process for the transportation of NTD Medicine is as follows:

1. The National NTDs Logistics Unit communicates details on NTDs medicines to be cleared and delivered to FCMS by the clearing agent. The unit will then communicate to the FCMS on the details of the NTDs medicines expected. Before arrival of the medicines, an email is received by the FCMS on the Certificate of analysis of the NTDs medicines.

2. The clearing agent communicates to FCMS the exact date of delivery of the medicines. They are also prompted to notify the FCMS 72 hours prior to delivery of the medicines.

3. On arrival of the medicines, the vehicle is inspected by the pharmacist to ensure it meets the standard of medicines transportation. The pharmacists also go ahead to inspect the condition of the medicines and take physical inventory, document and warehouse the medicines.

4. The National Logistic unit then sends distribution plan to the FCMS for issuance to the states and partners. There are other guidelines to be met before medicines are released to the state level. These include;

    i. Officers coming to receive must bring an authorization letter and mode of identification or letter of introduction from their states.
    ii. The NTD State Coordinator and State Pharmacist must be present.
    iii. Issuance is dependent on the transportation vehicle meeting the expected standard – containerized and unopened vehicle.

5. On meeting all standards, medicines are released to the transporter for onward delivery to the States, which cascade down to the end users through the relevant levels.

Flow Chart of Transport and Distribution Process

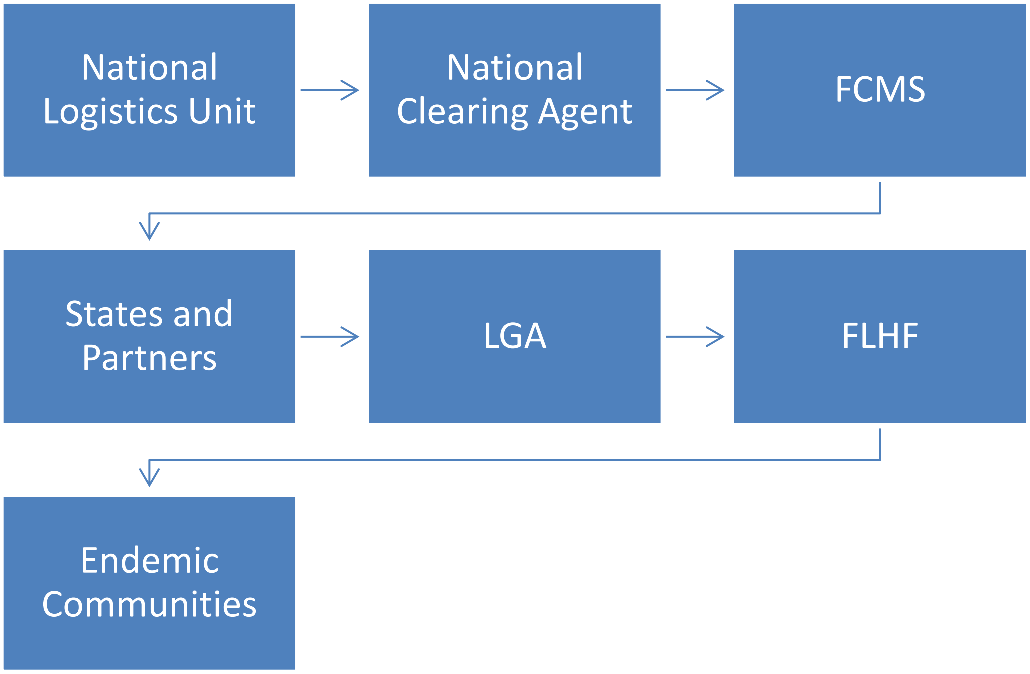
National Logistics Unit
National Clearing Agent
FCMS
States and Partners
LGA
FLHF
Endemic Communities

Roles and Responsibilities (Storage):

**Federal Central Medical Stores**	
Head of Central Medical Store	➢ Receives and store medicines<br>➢ Gives directives to raise Stock Allocation and Issue Voucher (SAIV)<br>➢ Approval of SAIV<br>➢ Prepare quarterly stock report and submit to NTDs Program
Pharmacist/Store Officer	➢ Populate the store ledger<br>➢ Keep accurate records of stock cards<br>➢ Issue the medicines in line with distribution plan.
Store Keeper	➢ Populates the stock card
Zonal Coordinators	➢ Coordinates all the States under the zone with respect to NTD activities<br>➢ Monitor and supervise all NTD projects from all the States i.e, drug distribution and other aspect of NTD administration<br>➢ Provide NTD headquarter with accurate data and information needed about each every state in the zone<br>➢ Conduct NTD zonal review meetings where progress and prospect of all the States across the zone are discussed, identify problems affecting each state project and proffer possible solutions.
**STATE CMS**	
Head of States Central Medical Store	➢ Receives and store medicines<br>➢ Gives directives to raise Stock Allocation and Issue Voucher (SAIV)<br>➢ Approval of SAIV<br>➢ Prepare quarterly stock report and

	submit to NTDs Program.
Pharmacist/Store Officer	➢ Populate the store ledger<br>➢ Receives and stores the medicines from the federal central medical stores<br>➢ Keep accurate records of stock cards as specified in the SOP<br>➢ Issue medicines to LGA stores using the appropriate distribution plan<br>➢ Monitor stock level of each commodity at the State and LGA stores<br>➢ Participate in monitoring and supportive supervision activities during MAM
NTD Coordinator/Pharmacist	➢ Quarterly stock report on NTD Medicine
Store Keeper	➢ Populates the stock card
**LGA STORES**	
LGA NTD Coordinator	➢ Quarterly stock report on NTD Medicine
LGA Store Keeper	➢ Populates the stock card<br>➢ Receive and store medicines
**FLHF STORES**	
FLHF In-Charge	➢ Quarterly stock report on NTD Medicine
Store Keeper	➢ Populates the stock card<br>➢ Receive and store medicines
**Community Level**	
Community Implementers	➢ Conduct census update<br>➢ Dispense medicines to end users<br>➢ Submission of medicine consumption records to FLHF<br>➢ Collate and return unused medicines to FLHF

Roles and Responsibilities (Transport and Distribution):

Human resource required for the transport and distribution process includes the following persons with the following roles and responsibilities:

	Level	Personnel	Roles
	**Federal**	**NTD National Logistic Officer**	• **Communicate details on NTD medicines to be delivered to the FCMS**<br>• **Communicate distribution plan on NTD medicines to be delivered to the States CMS**
		**Clearing Agent**	• **Clear the medicines**<br>• **Deliver medicines to the FCMS**
		**FCMS Pharmacist**	• **Receive, warehouse, document and issue NTD medicines to States**<br>• **Vehicle inspection before receipt and issuance of NTD medicines**
		**FCMS Store Officer**	• **Receive, warehouse, document and issue NTD medicines to States**<br>• **Conduct routine physical inventory of NTD medicines**<br>• **Ensure good warehouse practices are adhered to in accordance with the guidelines**<br>• **Issuance of NTD medicines**
		**Driver**	• **Transportation of the NTD medicines and materials from the sea port to FCMS**<br>• **Ensures the security of NTD medicines while in his custody**
	**State**	**NTD State Coordinator and Team**	• **Coordinate the receipt of NTD medicines from FCMS, and its distribution to the endemic LGAs in the state.**
		**Pharmacist**	• **Work with the NTD Coordinator to ensure the receipt of NTD medicines**

			from FCMS, and its issuance to the endemic LGAs in the state.<br>• Vehicle inspection before receipt and issuance of NTD medicines
		**Store Officer**	• Receive, store, document, and issue NTD medicines to LGAs in the states.<br>• Conduct routine physical inventory of NTD medicines<br>• Ensure good warehouse practices are adhered to in accordance with the guidelines<br>• Issuance of NTD medicines
		**Driver**	• Transportation of the NTD medicines and materials from the FCMS to the State level<br>• Ensures the security of NTD medicines while in his custody
	**LGA**	**NTD LGA Coordinator and Team**	• Coordinate the receipt of NTD medicines from State, and its distribution to the endemic FLHF in the LGA.
		**LGA Pharmacist/ Technician**	• Work with the NTD Coordinator to ensure the receipt of NTD medicines from the state, and its issuance to the endemic FLHF in the LGA.<br>• Vehicle inspection before receipt and issuance of NTD medicines
		**LGA Store Officer**	• Receive, store, document, and issue NTD medicines to FLHF in the LGA.<br>• Ensure good warehouse practices are adhered to in accordance with the guidelines<br>• Issuance of NTD medicines

		**Driver**	• **Transportation of the NTD medicines and materials from the State level to the LGA level**<br>• **Ensures the security of NTD medicines while in his custody**
	**FLHF**	**Officers in charge of the health facility eg. CHO, CHEW, JCHEW**	• **Receipt, documentation, issuance and distribution to the CIs and teachers of endemic communities**
	**Community**	**Community Leaders**	• **Witnesses the receipt and distribution of the endemic medicines to eligible persons at the community level**
		**Teachers**	• **Receipt and distribution of the NTD medicines to the eligible end users (pupils)**<br>• **Registration and record keeping of medicines utilized**<br>• **Collate and return the unused medicines back to the LGA FLHF**
		**CIs**	• **Receipt and distribution of the NTD medicines to the eligible end users**<br>• **Registration and record keeping of medicines utilized**<br>• **Collate and return the unused medicines back to the LGA FLHF**

4.9 Guidelines on drug handling at community level

The following medicine handling procedures should be followed:

i. Hand washing and general cleanliness should be observed by the dispensing personnel.
ii. Tablets should not be handled or dispense by bare hands rather dispensing spoon should be used.
iii. Narrow spoon should be used to remove tablets from the container to prevent wastage or contamination of the medicines.
iv. Tablets should be dispensed into dispensing envelope from the main container and labeled with name of the medicine, batch number and expiry date.

v. The remaining tablets should not be combined with another batch during the return process.
vi. Each container should be finished before opening another container.

Equipment/Tools required for good practices in medicines handling:

1. Dispensing envelopes – small and big size (labeled with space for name of drug, expiry date, batch number)

2. Counting trays and spoons

3. Hand Gloves

5. INVENTORY MANAGEMENT

Medicine Inventory Management System is designed to ensure effective management and monitoring of requisition, allocation, disbursement, receipt and consumption in health facilities across the process. In addition it provides comprehensive reports on medicine consumption patterns and regular updates on expired/ out of stock medicines; this system allows key stakeholders to manage medicine requisitions and allocation. Poor inventory management often leads to waste of financial resources, shortages or overages of others resulting in expiration, and decline in the quality of programme.

Inventory control system refers to the measures employed to manage the supply chain system in order to ensure. In the management of medicines for the NTD programme, it is most important to avoid these two undesirable occurrences: Stock outs and drug expiration. The purpose of an inventory control system is to inform the manager when to order and issue, and how to maintain appropriate stock level to avoid shortages and oversupply.

5.1 Logistics Management Information System

Information is the engine that drives the logistics cycle; without information, the logistics system would not run smoothly. The collection of data for managing a logistics system is a separate activity from the collection of data for other information systems, including health management information systems (HMIS).

Logistics is defined, “as the science and art of getting the right amounts of the right things to the right places at the right time” (Foster 1990). The program managers and storekeepers may have some logistics skills but not many health personnel involved in NTD adequately equipped to handle logistics. It is against this background that a sound knowledge and skills in store management and control form essential tool which all persons involved in the operations of the logistics support system MUST possess.

A logistics management information system (LMIS) is the system of records and reports that you use to collect, organize, and present logistics data gathered across all levels of the system. Most important, an LMIS enables logisticians to collect the data needed to make informed decisions that will ultimately improve customer service.

5.2 Purpose of LMIS

Ultimately, the goal of every public health logistics system is to help ensure that every customer has commodity security. Commodity security exists when every person is able to obtain and use quality essential health supplies whenever he or she needs them.

Information is the engine that drives the entire logistics cycle. We collect information to make decisions; the better information we have, the better decisions we can make. The purpose of LMIS is to collect, organize and report data that will be used to make decisions. However, every system is open to an endless flow of information. Thus, only those data items that will be needed for decision making will be collected and processed. To make logistics decisions, a logistics manager needs three essential data items: a) stock on hand, (b) rate of consumption, and (c) losses and adjustments.

5.3 The Six Rights of LMIS data

The purpose of an efficient and effective reporting system is to provide :

- the **right** (relevant) **information**
- in the **right quantity** (complete) and
- **right quality** (accurate); to
- the **right place** (the next reporting level or decision makers)
- at the **right time** (timely)
- for the **right cost** (not expensive).

5.4 Essential features of NTDs LMIS

Enhanced Visibility

The programme has put in place a mechanism for mapping facilities and personnel to ensure that facility-based logistics data/analyses are communicated to all stakeholders at the state, zone and central levels. The reports are used for routine performance appraisal, planning, stock replenishment, feedback and provision of support which will enhance performance.

Enhanced intelligence

Capacity to see where issues (unwanted outcomes and situations) and strengths (desirable situations and outcomes) arise from. This is done by analysing data in such a way as to gain insight into operations at other work areas.

Enhanced Accountability & transparency

Facilities are expected to document and report all transactions that affect the quantities of stock on order, received, in storage, dispensed or lost.

Enhanced awareness of self-performance profile

The system that provides opportunity for self-appraisal using agreed metrics.

Enhanced Institutional memory

Records and reports are preserved for easy referencing and future planning which ensures data consistency.

5.5 Logistics Records

There are only three things that can be done to any health commodity that is received. It can be stored, moved or consumed. Therefore three types of records are kept to track these activities. The records are:-

- **Stock Keeping Records:** They keep information about products in storage. The primary purpose of stock keeping records is to document information about items in storage. They must contain quantity of stock on hand and the quantity of losses and adjustments.

- **Transaction Records:** They keep information about products being moved. The primary purpose of transaction records is to document information about the movement of stock from one storage facility to another. They do not have to include any of the essential data items earlier mentioned

- **Consumption record:** They keep information about products being consumed. The primary purpose of consumption record is to document information on the quantity of each item dispensed to a patient.

Rationale for Record Keeping

i. Records are instruments of transparency–such that claims can be verified
ii. Records MUST be true to facts on the ground so as not to mislead i.e. they must reflect logistics related events during the quarter, position of things such as medicines at the beginning and at the end of the quarter.
iii. Records must be preserved for a minimum of 5 years

5.6 Logistics Reports

Records are data generated and retained at the facility while reports are data processed into information and sent to a higher level. The primary purpose of a summary report is to capture data items for a specific storage facility and for a specific period of time (usually monthly or quarterly).

Summary reports must contain the three essential data items-stock on hand, consumption and losses and adjustments. Other data items captured include opening balance, quantity received, and quantity issued/dispensed.

i. Opening balance: Stock on hand at the beginning of the reporting period.

ii. Quantity Received: Amount of stock received within the reporting period.

iii. Quantity Issued: Amount of stock issued within the reporting period.

iv. Losses and Adjustments: Losses are quantity of stock removed from the pipeline for any other reason other than consumption by clients/patients (e.g. due to theft, expiration and damage, breakage etc). Adjustments are made when quantities are issued to or received from other facilities at the same level e.g. Loan.

v. Closing balance: Stock on hand at the end of the reporting period.

5.7 Logistics Management Information Tools (LMIS)

The Logistics Management Information System for NTD medicines consist of three records and one report.

The NTD LMIS tools include: (i) Stock Allocation, Issue and Receipts Voucher, (ii) Stock ledger, (iii) Stock returns and transfer form, (iv) Stock status report and Expiry date tracker.

1. **Stock Allocation, Issue and Receipts Voucher:** This is used to track consignment of medicines that are moved from one level to another. It usually accompanies the consignment. It tracks the quantities that are shipped and actual deliveries.

2. **Stock Ledger**: The stock ledger is stock keeping card that keeps information about the movement and quantity of a product in the facility's storage area.

3. **Stock returns and transfer form**: This is a transaction record used to track reversed movement or transfer of medicines from lower level to higher level storage facilities. These are usually unusable stock due to damage, expiry or due to overstocking.

4. **Stock status report and Expiry date tracker:** This is a combined report showing the balance of the medicines and the expiry date.

5.8. Job Aids for Logistics Management Information Tools

A job aid provides step-by-step instructions for completing an activity or a task such as filling a form. The job aids included in this manual are primarily designed to guide health commodity managers to perform a task or complete a form in the logistics system. Typical job aids are characterized by seven features, namely:

• The task to be performed

• The person who is supposed to complete the task

• The purpose of the task

• The timeline to complete the task

• Any materials needed to complete the task

• The step-by-step instructions, which include what to do and how to do it

• A checklist to verify that the task has been completed.

The purpose of a job aid is to improve performance efficiency and effectiveness as well as to standardize practices. If all commodity managers follow the job aids correctly, then the tasks will be done correctly and in the same manner, regardless of who completes the task.

Contained in these manual are the job-aids for performing the following tasks:

- Completing the stock allocation/ Issue and Receipt Vouchers
- Completing the Stock ledger
- Completing the Medicines Returns/ Transfer form
- Completing the Quarterly Stock Status report
- Completing the Stock Expiry Date Tracker

5.9 Completing the Stock Allocation Issue and Received Voucher

This job aid will guide you through the process of completing the form for NTDs stock allocation/issue and receipt voucher. The record is used when a higher level facility is issuing NTDs drugs to a lower level facility. This is a transaction record which is in quadruplicate (4 copies). On completion of the above activity, the copies are distributed as follows:

- White copy: Receiving facility
- Yellow copy: Issuing facility (Proof Of Delivery)
- Green copy: The transporter
- Blue copy: This remains at the issuing facility (tickler copy).

Task: Allocating/issuing and receiving of NTD drugs

Completed by:

Store manager/Pharmacist in charge at the issuing store who approves issue of NTD drugs;

Store officer at the issuing store.

Store officer at the receiving store.

The transporter.

Person witnessing the receipt of the NTDs drugs.

Purpose: To account for the movement of NTDs drugs from a higher to a lower level and also to serve as a proof of delivery (POD).

When to perform: Every time NTDs drugs are allocated/ issued, moved and received at each level of the NTDs supply chain. This is done when NTDs drugs are moved from a higher to a lower level.

Materials needed: Approved distribution plan /allocation letter, Pen, blank Stock Allocation, Issue and Receipt Voucher, calculator.

Note: ***The persons filling out the form should apply a little pressure to ensure that all the carbonated copies are legible***

Step	Action	Notes/Example
**Issuing Facility (FCMS, CMS, LG Store or FLHF)**		
1.	**State:**<br><br>Write the name of the state.	Zamfara
2.	**LGA:**<br><br>Write the name of the LGA that is returning the drugs.	Bungudu
3.	**Issuing facility:**<br><br>Write the name of the facility that is issuing the drugs	CMS, LGA medical store or FLHF
4.	**Receiving facility:**<br><br>Write the name of the facility that would receive the drugs	Furfuri PHC
5.	**Date**:<br><br>Write Date of Issuing drugs	7/11/2014
6.	**Item Description, Strength & Dosage form:**<br><br>Write the description, strength and dosage form of the NTDs drugs.	Zithromax tablet 250mg
7.	**Unit:**<br><br>Write the smallest unit of measure for the NTDs drugs	Unit of NTDs drugs is the smallest unit of measure that can be dispensed to a patient.<br><br>Tablet, Cap, Bottle, Tube.
8.	**Quantity Allocated:**<br><br>Write the quantity of the drug(s) allocated to the facility	600
9.	**Batch No:**<br><br>Write the batch number of the drug(s) to be issued.	EPA22221

Step	Action	Notes/Example
10.	**Expiry Date:**<br><br>Write the expiry date of the drug being issued	12/2015
11.	**Quantity Issued:**<br><br>Write the quantity of NTDs drugs being issued to the facility	600
12	**Remarks:**<br><br>Add any comments regarding the quantity issued	For clarity, write the quantity issued in packs of cartons or Tins.
13.	Detach the first three (3) copies and send with the NTDs drugs to the receiving facility.	The signed yellow copy will be returned to the issuing facility as proof of delivery (POD).
**Receiving Facility**		
14.	**Quantity Received:**<br><br>Write the quantity of NTDs drugs being received.	600
15	**Remarks:**<br><br>Add any comments regarding the quantity received	Complete, 50 damaged, short of 50 etc.
**Signatures**		
16.	**Approved By:**<br><br>Write the Name, Designation, Signature, date and mobile number.	This is filled in by the Store Manager, Store Pharmacist, LG NTDs coordinator or officers-in-charge at issuing facility or their designate.
17.	**Issued By:** Write the Name, Designation, Signature, date and mobile number.	This is filled by the Store pharmacist, Store officer at the issuing store.
18.	**Delivered By:** Write the Name, vehicle registration no, Signature, date and mobile number.	This is filled in by the person responsible for transporting the NTDs drugs.
19.	**Received By:**<br><br>Write the Name, Designation, Signature, date and mobile number.	This is filled in by the person designated to do so at the receiving facility.

Step	Action	Notes/Example
20.	**Witnessed By:**<br><br>Write the Name, Designation, Signature, date and mobile number..	This is filled in by the person designated to do so at the receiving facility.<br><br>**The designates include**: The state NTDs coordinator, The LGA NTDs coordinator, Ward Head and Village head or their representatives

This transaction s has been completed when:
The description, unit, expiry date and batch No. of each NTDs drug has been filled in the Stock Allocation, Issue and Receipt Voucher.<br><br>The Quantity allocated and Issued has been entered on the Stock Allocation, Issue and Receipt Voucher for each item.<br><br>The quantity received has been entered on the Stock Allocation, Issue and Receipt Voucher for each item received;<br><br>Names, Designations, Signatures, dates and phone Numbers have been completed on the voucher by the concerned personnel.<br><br>The yellow copy (POD) of the Stock Allocation, Issue and Receipt Voucher with the Quantity received filled in and signed has been received from the transporter and filed by the issuing store for its records.

STOCK ALLOCATION AND ISSUE VOUCHER

FEDERAL REPUBLIC OF NIGERIA
NEGLECTED TROPICAL DISEASES PROGRAM Serial No: ____________

ALLOCATION / ISSUE/ RECEIPT VOUCHER

State: ________________________ LGA:___________

Name Of Issuing Facility: __________________________ Date:________________

Name Of Receiving Facility: __________________________

For Issuing Facility Use								For Receiving Facility Use	
S#	Item (Description Strength & Dosage Form)	Unit* (e.g Tablet, Bottle, Tube)	Quantity Allocated	Lot/ Batch No.	Exp. Date	Quantity Issued	Remarks	Quantity Received	Remarks
1									
2									
3									
4									
5									
6									
7									
8									
9									
10									

Goods checked & delivered in good condition except items detailed in the above discrepancy remarks				
APPROVED BY:	ISSUED BY:	DELIVERED BY:	RECEIVED BY:	WITNESSED BY:
Name: ____________	Name: ____________	Name: ____________	Name: ____________	Name: ____________
Designation:	Designation:	Designation:	Designation:	Designation:
Signature: __________	Signature: __________	Signature: __________	Signature: __________	Signature: __________
Date: __________	Date: __________	Date: __________	Date: __________	Date:______________
Phone No:	Phone No:	Phone No:	Phone No:	Phone:

5.10 Completing the Stock Ledger

This job aid will guide you through the process of completing the store ledger. The Store ledger is used to track each NTDs drugs in the stores. Each time there is a change in the quantity of the NTDs drugs in the stores, it must be recorded on the appropriate page of the ledger; that is when products are received or issued. The information tracked on the store ledger will facilitate the management of inventory at the facility.

Task:	Completing the Store ledger
Completed by:	Store officer, Store Pharmacist, or Store Manager.
Purpose:	To track the inflow and outflow of NTDs drugs and the balance at every point in time.
When to perform:	Each time there is a transaction (Drug movement like issue, receipt, return, transfer, loss or adjustment etc.) that affects the stock level of a product.

Materials needed: Store ledger, allocation, issue and receipt voucher, return /transfer form, meter rule calculator, red and blue or black pen.

Step	Action	Notes/Example
1.	**State**:<br>write the name of the state	
2.	**LGA**:<br>Write the name of the LGA	Daura
3.	**Nameof Facility**:<br>Write the name of the facility.	MCHC Daura
4.	**Item Description**:<br>Write the drugs name, including the form and strength.	Zithromax tablet 250mg
5.	**Unit of Issue**:<br>Write the unit for the NTDS drug.	Unit of NTDs drugs is the smallest unit of measure that can be dispensed to a patient.<br>Tablet, Cap, Bottle, Tube, piece, etc.
6.	**Date**:<br>Write the date of the transaction.	The date should be the day the transaction was carried out.<br>5/5/2014
7.	**Received from / Issued to**:<br>Write the facility or the person/organization from which the NTDs drugs is coming or is being sent.	FCMS, State Medical Stores, LGA medical store, PHC Samiya.
8.	**Transaction Voucher No**:<br>Write the voucher number of the form that accompanied the NTDs drugs.	1001010

9.	**Batch Number**:<br>Write the batch number of the product.	EPA2222
10.	**Expiry Date**:<br>Write the expiration date of the product.	30/12/2014
11.	**Quantity Received**:<br>Write the quantity of the product that is received.	1,000
12.	**Quantity Issued**:<br>Write the quantity of the product that is being issued.	500
13.	**Losses:** Enter the exact amount of<br><br>Losses to the inventory on this date.<br><br>Explain any losses in the "Remarks"	Losses are quantities removed from<br><br>your stock for anything other than<br><br>dispensing to patients or issuing to
14.	**Adjustments:** Enter adjustments<br><br>either positive or negative, if any.<br><br>Explain any adjustments in the<br><br>"Remarks" column	**Adjustments** are quantities of a product received from any source other than the approved supply chain source.<br><br>Adjustment can either be positive (+) or negative (-): To the facility receiving it is a positive (+) adjustment while to the facility transferring it is a negative (-) adjustment to their stock balances.<br><br>Always use a (+) sign to indicate positive (+) adjustments, and a negative (-) sign to indicate negative adjustments.<br><br>A positive (+) adjustment could be when products are "found" during a physical count.

15.	**Balance**:<br>Calculate and write the new stock balance.	If products were received, add the quantity received to the previous stock balance and write the total.<br><br>If products were returned to your store, add the quantity returned to the previous stock balance and write the total.<br><br>If products were returned by your store, subtract the quantity returned from the previous stock balance and write the total.<br><br>If products were issued, subtract the quantity issued from the previous stock balance and write the total.<br><br>If products were lost or adjusted, add or subtract the quantity from the previous stock balance and write the total.
16.	**Signature**:<br>Sign the store ledger once the stock transaction has been recorded.	xxxxxxxxxx
17.	**Remarks**:<br>Write any comment related to the transaction that may be needed.	Example:<br>Damaged, transferred, or lost

Thetaskiscompletewhen:

- Whenthe state, LGA, facilityname,itemdescription,unitofissue,item code,batchnumber, transaction date, transaction voucher number and expirydatearefilledin.
- Whenthedate,receivedfrom/issuedtocolumns are completed for every transaction.
- Whenoneofthefollowingcolumns is completed:QuantityReceived, losses, adjustments orQuantityIssued.
- Whenthestockbalancehasbeencalculatedandrecorded.
- Whenthepersonrecordingthetransactionsignsthe store ledger.
- When the officer makes remarks where necessary.

FEDERAL REPUBLIC OF NIGERIA

NEGLECTED TROPICAL DISEASES PROGRAM

STORE LEDGER

Serial No.__________________

State: ____

LGA: _____________________

Name of facility: _______________________________

Item Code: _________________

Unit of Issue: _______________

Item Description: ______________________________

(e.g Tablet, Bottle, Tube)

Date	Received from / Issued to:	Transaction Voucher No.	Batch No.	Expiry Date	Quantity Received	Quantity Issued	Losses	Adjustment	Stock Balance	Signature/ Remarks

5.11 Completing the Medicines Returns and Transfer Forms

This job aid will guide you through the process of completing the NTDs drugs return/transfer form from one level to another. Drug return process is the movement of NTDs drugs from a lower to a higher level while drug transfer is the movement of NTDs drugs between facilities at the same level

This is a transaction record which is in quadruplicate (4 copies). On completion of the above activity, the copies are distributed as follows:

- White copy: Receiving facility
- Yellow copy: Returning/transferring facility
- Green copy: The transporter
- Blue copy: This remains at the returning/transferring facility (tickler copy).

**Task:**	Completing the NTDs drugs return/transfer form.
**Completed by:**	The officer at the community, FLHF or LGA that is returning/transferring the NTDs drugs.<br>The officer approving the return/transfer of the NTDs drugs.<br>The person responsible for the transport of the NTDs drugs.<br>The person receiving the NTDs drugs.<br>The person witnessing the transaction.
**Purpose:**	To track the return/transfer of NTDs drugs.
**When to perform:**	Each time NTDs drugs are to be returned/transferred.
**Materials/Tools needed:**	Blank NTDs drugs return/transfer form, calculator, meter ruler and pen.

Step	Actions	Notes/Example
1	**State:**<br>Write the name of the state.	**State:**<br>Niger
2	**LGA:**<br>Write the name of the LGA that is returning/transferring the drugs.	**LGA:**<br>Kontagora
3	**Receiving facility:**<br>Write the name of the facility where the NTDs drugs are to be returned/transferred.	**Receiving facility:**<br>FLHF/LGA/ State CMS
4	**Drugs Returning/Transferring Facility:**<br>Write the name of the facility that is returning/transferring the NTDs drugs.	**Drugs Returning/transferring Facility:**<br>PHC Ibeto, Kontagora LGA store or CMS
For each drug being returned/transferred:		
5.	**Item description, Strength & dosage form:**<br>Write the name and description of the drug.	**Item description, Strength & dosage form:**<br>Zithromax tablet 250mg
6.	**Unit:**<br>Write the smallest unit of measure for the NTDs drug.	**Unit:**<br>Tablet, Cap, Bottle, Tube, etc.
7.	**Batch No. :**<br>Write the batch number of the drugs being returned/ transferred.	**Batch No. :**<br>EPA22221

8.	**Expiry Date:** Write the expiry date of the drugs being returned/transferred.	**Expiry Date:** December 2014 or 12/2014
9.	**Quantity Returned/transferred:** Write the quantity of NTDs drugs being returned/transferred.	**Quantity returned/transferred:** 600 tabs, caps, tubes, or bottles.
10.	**Reason for Return/transfer:** Write the reason for which the product is being returned	**Reason for Return/transfer:** Damaged, expired, unused or redistribution.
12.	**Items return/transfer officer:** The person that is returning/transferring the NTDs drugs writes his/her name and signature, mobile number and date.	Musa Bashir, *MB*, 080xxxxxxxxxxx, 16/11/15
13.	**Items return/transfer Approving Officer:** The person who approves the return/transfer writes his/her name, signature, mobile number and the date.	Moses David, *MD*, 080xxxxxxxxxxxx, 16/11/15
14.	**Transporter:** The Driver transporting the NTDs drugs writes his/her name, signature, mobile number, date and vehicle registration number.	NagogoAkanbi, *NA*, 080xxxxxxxxxxxx, 16/11/15, BDG 114 XY
15.	**Receiving Facility:** The person who receives the returned/transferred NTDs Drugs writes his/her name, signature, mobile number and the date.	Jide John, *JJ*, 080xxxxxxxxxxxx, 17/11/15
16.	**Receiving Witness:** The person who witnesses the receipt of the returned/transferred NTDs Drugs writes his/her name, signature, mobile number and the date.	AmarachiOnyema, *AO*, 080xxxxxxxxxxxx, 17/11/15
17.	**Remarks:** This is written by the Receiving officer to acknowledge the quantity and condition of the returned drugs.	eg complete, incomplete, unlabeled, improperly packaged etc

- **Thistaskincompletedwhen**:
- The names of the State, LGA, facility to which the NTDs drugs were sent and the facility returning/transferring the drugs have been completed.
- The returned/transferred drug is fully described by batch number, expiry date, the quantity returned/transferred recorded and the reason(s) for the transaction stated.
- When the person returning/transferring the drugs signs the form.
- When the transporter signs the form.
- When the approving officer signs the form.
- When the witness to the transaction signs the form. When the receiving officer signs the form.
- When a signed copy of the form is sent back to the facility that returned/transferred the NTDs drugs.

FEDERAL REPUBLIC NIGERIA

NEGLECTED TROPICAL DISEASES PROGRAM

RETURN/ TRANSFER FORM **Serial No:** ______________

State: _______________________ **LGA:** ___________________________

Receiving Facility: ____

Return / Transfer Facility[1]: ____

S #	Item Description, Strength & form	Unit	Lot / Batch No.	Expiry Date	Quantity in units	Reason for Return/ Transfer
1.						
2.						
3.						
4.						
5.						

Item(s) Return / Transfer officer		Item(s) Return / Transfer Approving Officer	
Name		Name	
Signature		Signature	
Phone No		Phone No	
Date		Date	

Transporter

Name of Driver: ____________________________________ **Signature:** _____________________

Phone No:____**Date:**_________________________

Vehicle Reg. No:_____________________________________

Receiving officer		Witness	
Name		Name	
Signature		Signature	
Phone No		Phone No	
Date		Date	

Remarks:____
____

[1] Facility also includes schools, community , medical stores and health facility

5.12 Completing the Quarterly Stock Status Report

This job aid will guide you through the process of completing the stock status report of NTDs drugs. The form is to be completed on a quarterly basis. This form is in duplicates and comes as a booklet. On completion of the form (which is at the first week of a new quarter), the copies are distributed as follows:

- White copy: Next Level in the supply chain e.g. LGA medical store submits report to State CMS
- Pink copy: Retained in the booklet of the facility submitting the report.

Task: Preparing the stock status report for NTDs drugs

Completed by: Store officer, Store manager/Pharmacist in charge at the facility (LGA and SCMS)

Purpose: To provide a report of the quantity issued, quantity received losses, adjustments and stock balance at each level of the supply chain on a regular basis for decision making.

When to perform: First week of a new quarter

Materials needed: Blank stock status report form, store ledger, actual quantities from physical count

Note: ***The persons filling out the form should apply a little pressure to ensure that the carbonated copy is legible.***

Step	Action	Notes/Example
**Reporting Facility (CMS, LGA Store)**		
1.	**Month/Year**<br><br>Write the month and year the report was prepared	April, 2014
2.	**State:**<br><br>Write the name of the state.	Kano
3.	**LGA:**<br><br>Write the name of the LGA.	Filled only for LGA report
4.	**Quarter:**<br><br>Tick the reporting quarter	Quarter 1: Jan – March<br><br>Quarter 2: April – June<br><br>Quarter 3: July – September<br><br>Quarter 4: October – December
5.	**Item Description, Strength & Dosage form:**<br><br>Write the description, strength and dosage form of the NTDs drugs.	Zithromax tablet 250mg
6.	**Opening Balance**<br><br>Write the stock on hand (SOH) at the beginning of the reporting quarter	50,000 Tabs.<br><br>The opening balance is the previous closing balance of the last quarter
7.	**Quantity Received:**<br><br>Write the total quantity of the drug(s) received during the reporting quarter	120,000 Tabs.
8.	**Quantity Issued:**<br><br>Write the total quantity of the drug(s) issued during the reporting quarter	110,000 Tabs.

Step	Action	Notes/Example
9.	**Losses:**<br><br>Enter the exact quantity of losses to the inventory during the reporting quarter.	500 Tabs<br><br>Losses are quantities removed from your stock for anything other than dispensed to patient or issued to other facilities e.g. expired, lost, stolen, pilfered or damaged
10	**Adjustment:**<br><br>Enter adjustments<br><br>either positive or negative done during the reporting quarter	**Adjustments** are quantities of a product received from any source other than the approved supply chain source.<br><br>Adjustment can either be positive (+) or negative (-): To the facility receiving it is a positive (+) adjustment while to the facility transferring it is a negative (-) adjustment to their stock balances.<br><br>Always use a (+) sign to indicate positive (+) adjustments, and a negative (-) sign to indicate negative adjustments.<br><br>A positive (+) adjustment could be when products are “found” during a physical count.<br><br>Adjustments may also be made to correct mathematical mistakes previously made in recording. Be sure to indicate if the adjustment was negative or positive.
11.	**Closing balance:**<br><br>The actual quantity after the physical count at the end of the reporting quarter.	59,500 Tabs.
**Signatures**		
16.	**Reported By:**<br><br>Write the Name of the officer preparing the report.	MallamBukarMusbau.

Step	Action	Notes/Example
17.	**Designation:**<br><br>Write the title/position of the officer preparing the report.	Store Manager, Store Pharmacist, or officer-in-charge of the facility.
18.	**Signature:**<br><br>The officer who prepared the report signs it	xxxxxxxxxxxxx.
19.	**Date:**<br><br>Write the date the report was prepared.	2nd April, 2014

**This report has been completed when:**
1. The month, year, state, LGA and reporting quarter have been filled.<br>2. The item description, opening balance, quantity received, quantity issued and closing balance have been filled.<br>3. When applicable the losses and/or adjustment have been filled.<br>4. The name of the person preparing the report, designation, date and signature has been filled.

STOCK STATUS REPORT

FEDERAL REPUBLIC OF NIGERIA

NEGLECTED TROPICAL DISEASES PROGRAM

STOCK STATUS REPORT

Month.................Year..................

Serial No: ____________

State: ______________ **LGA:** ________________

Quarter 1(Jan – Mar) ( )
Quarter 2(Apr – Jun) ( )
Quarter 3(Jul – Sept) ( )
Quarter 4(Oct – Dec) ( )

Reporting Facility: ________________

S/N	Item Description	Opening Balance	Quantity Received	Quantity Issued	Losses	Adjustment	Closing Balance

Report Prepared By: ________________ Designation: ________________ Date: ________ Signature: ______________

5.12 Completing the Stock Expiry Date Tracker:

This job aid will guide you through the process of completing the expiry date tracker of NTDs drugs. The form is to be completed on a quarterly basis and attached to the stock status report. This form is in duplicates and comes as a booklet. On completion of the form (which is at the first week of a new quarter), the copies are distributed as follows:

- White copy: Next Level in the supply chain e.g. LGA medical store submits report to State CMS
- Pink copy: Retained in the booklet of the facility submitting the report.

Task: Preparing the expiry date tracker for NTDs drugs

Completed by: Store officer, Store manager/Pharmacist in charge at the facility (LGA and SCMS)

Purpose: To provide a report of the quantity, batch number and expiry date of both usable and non usable drugs in the reporting quarter.

When to perform: First week of a new quarter

Materials needed: Blank expiry date tracker, store ledger, actual quantities from physical count

Note:***The persons filling out the form should apply a little pressure to ensure that the carbonated copy is legible.***

Step	Action	Notes/Example
**Reporting Facility (CMS, LGA Store)**		

Step	Action	Notes/Example
1.	**Month/Year**<br><br>Write the month and year the report was prepared	April, 2014
2.	**State:**<br><br>Write the name of the state.	Kano
3.	**LGA:**<br><br>Write the name of the LGA.	Filled only for LGA report
4.	**Quarter:**<br><br>Tick the reporting quarter	Quarter 1: Jan – March<br><br>Quarter 2: April – June<br><br>Quarter 3: July – September<br><br>Quarter 4: October – December
5.	**Item Description (Strength & Dosage form):**<br><br>Write the description, strength and dosage form of the NTDs drugs.	Zithromax tablet 250mg
6.	**Quantity:**<br><br>Write the quantity of the expired drugs and stock on hand by their batches for the reporting quarter.	200 Tabs
7.	**Batch Number:**<br><br>Write the respective batch number for the expired drugs.	EPA22221
8.	**Expiry Date:**<br><br>Write the expiry date of each drug	4th May, 2014.
9.	**Remarks:**<br><br>State whether the drug is expired or damaged	Damaged/expired
**Signatures**		
10.	**Reported By:**<br><br>Write the Name of the officer preparing the report.	MallamBukarMusbau.

Step	Action	Notes/Example
11.	**Designation:**<br><br>Write the title/position of the officer preparing the report.	Store Manager, Store Pharmacist, or officer-in-charge of the facility.
12.	**Signature:**<br><br>The officer who prepared the report signs it	xxxxxxxxxxxxx.
13.	**Date:**<br><br>Write the date the report was prepared.	2nd April, 2014

**This report has been completed when:**
5. The month, year, state, LGA and reporting quarter have been filled.<br>6. The item description, quantity, batch number, expiry date and remarks have been filled.<br>7. The name of the person preparing the report, designation, date and signature has been filled.

STOCK EXPIRY DATE TRACKER

FEDERAL REPUBLIC OF NIGERIA

NEGLECTED TROPICAL DISEASES PROGRAM

EXPIRY DATE SUMMARY

Month.................Year.................   Serial No:

State: ______________   LGA: ________________

Quarter 1 (Jan – Mar) [ ]   Quarter 3 (Jul – Sept) [ ]

Quarter 2 (Apr – Jun) [ ]   Quarter 4 (Oct – Dec) [ ]

S/N	Item Description	Unit	Quantity	Batch No	Expiry Date	Remark

Report Prepared By: ________________ Designation: ________________ Date: ________ Signature: ______________

5.13 Feedback Reports

When data is received from the lower level, it is verified for correctness. Observations made after verification should be sent as feedback to the lower levels. Hence feedback reports are sent from higher levels to lower levels informing them about their performance both good and bad. It also encourages improved performance of the medicine supply chain. Feedback report can be provided through email, phone calls or memo.

6. REVERSE LOGISTICS AND WASTE MANAGEMENT

Reverse logistics refers to process of returning usable surplus supplies (and expired or damaged supplies) from lower level to the next level in the supply chain in order to facilitate redistribution to places where they are needed.

Health Waste management refers to process of managing expired, damaged or other unusable medicines and commodities. This is important to prevent dispensing or usage expired or damaged products with serious health consequences. Efforts should be made by all stakeholders to ensure that medicine are kept safe from unauthorized individual and returned to a safe location for disposal after the end of their life or in case of damage.

6.1 Reverse logistics of NTDs Medicines

The process for returning unused damaged or expired medicines as well as roles and responsibilities of key stakeholders are as follows:

- CDDs/CI/Teachers return unused, damaged or expired medicines to the Front line healthy facility/Zonal Education office after the completion of treatment for the year using the appropriate tool (Reporting forms).

- FLHFS/ZONAL EDUCATION OFFICER receives the returned medicines from the community directed distributors/Community implementers/Teachers in their area and document appropriately send to the LGA medical store under the supervision of the NTDs LGA coordinator.

- NTDs LGA COORDINATOR confirms the receipt of medicines returned to the LGA and facilitates the movement of the medicine to the State central medical store.

- LGA/STATE CENTRAL MEDICAL STORE OFFICERS receives the medicine returned to the store and document appropriately.

- STATE NTDs COORDINATOR confirms receipt of returned medicines from all LGAs on behalf of the State NTDs programme.

Timelines and stock movement procedures

Timelines	Stock Movement Level	Stock Movement Procedures	Reason(s)
Immediately after treatment	CDDs/CIs/Teachers	CDDs/CDIs/Teachers return medicines to front line health facility/LGA education officer	Damaged, expired, not used etc for return of medicine should be stated alongside with other information such as the quantity returned, batch number etc.
One week after treatment	Frontline Health Facility/LGA Education Officers	Frontline Health Facility/LGA Education Officers return medicines to LGA central medical store after due consultation with the LGA NTD Coordinator	Damaged, expired, not used etc for return of medicine should be stated alongside with other information such as the quantity returned, batch number etc.
One week after treatment	LGA medical Store	LGA medical store officer keeps custody of returned products and keeps records.<br><br>Update the stock ledger and also prepare the stock status quarterly report.	Same as above
Two weeks after treatment	State Central Medical Store	Take custody of returned products and keeps records.<br><br>State collates the quantity returned/reasons with the batch	Same as above

		numbers clearly stated.<br><br>Update the stock ledger and also prepare the stock status quarterly report.	

6.1 SOPs on management of waste documentation and destruction.

Management of expired/damaged medicines:

- Gathering/collection of the medicines at the FLHF level.
- Report should be made to the LGA level.
- Verification/stock taking of the medicine at the State Level.
- Transfer of medicine to NAFDAC designated sites by FMOH / CMS from the state central medical stores.
- FMOH arrange for disposal of the medicines

6.2 Wastes Management of NTDs Medicines

Wastes generated in the NTD programme may include, medicines, medicine containers, dispensing envelopes, packaging materials, etc.

Generally, there are four key steps to the management of medicines and other related products, they are:

a) Segregation into various components, including reusable and safe storage in appropriate containers;
b) Transportation to waste treatment and disposal sites
c) Treatment(for medicines that require treatment before final disposal)
d) Final disposition.

Primary packaging and storage takes place where waste is generated. Secondary packaging is used for transportation.Proper segregation of waste generated will greatly reduce the amount of waste that needs expensive treatment. For this reason, items such as cartons, packaging, and non-consumable disposables (e.g., dispensing envelopes) should be segregated from medicines. Expired and damaged medicines should be properly segregated from other wastes in the segregation process, as they may otherwise be resold by waste pickers.
