# Supplementary material for: Optimising the performance of frontline implementers engaged in the NTD programme in Nigeria: lessons for strengthening community health systems for universal health coverage
Source: Hum Resour Health. 2019 Nov 1;17:79. doi: 10.1186/s12960-019-0419-8 (PMC6824027; doi:10.1186/s12960-019-0419-8)
Supplement: Supplementary file 3 — Additional file 3. Standard Operating procedures for Neglected Tropical Disease (NTDs) Elimination in Nigeria [file 12960_2019_419_MOESM3_ESM.pdf]

# Standard Operating Procedures (SOPs) for Neglected Tropical Diseases (NTDs) Elimination in Nigeria

SOPs for PC & IDM NTDs

*Neglected Tropical Diseases Programme, Federal Ministry of Health, Abuja*

# STANDARD OPERATING PROCEDURES (SOPs) FOR NTDs ELIMINATION IN NIGERIA

## *Preventive Chemotherapy (PC) NTDs*

| **Diseases** | **Lymphatic Filariasis (LF)** | **Onchocerciasis (ONCHO)** | **Schistosomiasis (SCH)** | **Soil-transmitted Helminths (STH)** | **Trachoma** |
|---|---|---|---|---|---|
| **Photos** | 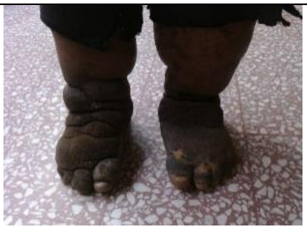 | 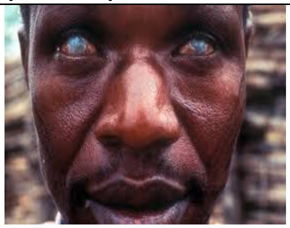 | 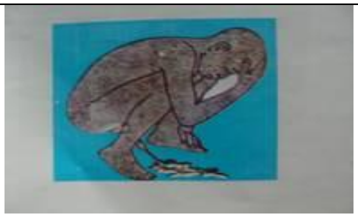 | 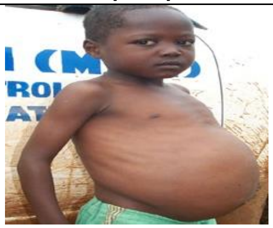 | 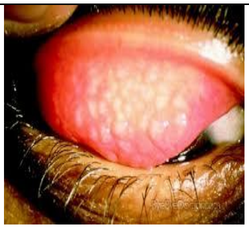 |
| **Disease Definition** | Lymphatic Filariasis (LF) is caused by a thread-like parasitic worm *Wuchereria bancrofti* in Nigeria. The disease is spread from infected person to uninfected person through mosquito bites. The adult worm lives in human lymph vessels and has a life span of 4-6 years. The microfilaria (immature worm) circulates in the peripheral blood with a marked nocturnal periodicity. Major chronic stages of LF are elephantiasis and hydrocoele. | Onchocerciasis is caused by a filarial worm known as *Onchocerca volvulus.* The disease only infects humans and is transmitted through the bite of an infected black fly of the Simulium genus, predominantly found around fast flowing rivers and streams. These flies transmit immature larval forms of the parasite from person to person. The parasites on maturing into adult female worms, can live up to 14 years and produce thousands of microfilariae. | Schistosomiasis, also known as bilharziasis or snail fever, is a primarily tropical parasitic disease caused by the larvae of one or more of five types of flatworms or blood flukes known as schistosomes. There are five types of schistosome:*Schistosoma mansoni, S. mekongi, S. japonicum, S. intercalatum, S. iaematobium,* Eggs are excreted in human urine or faeces and, in areas with poor sanitation, contaminate freshwater sources. The eggs break open to release a form of the parasite called miracidia. Freshwater snails become infested with the miracidium, which multiply inside the snail and after about a month mature into new larvae (cercariae) that the snail ejects into the water. The cercariae, which survive outside a host for 48 hours, quickly penetrate unbroken skin, and travel | Soil-transmitted helminths refer to the intestinal worms infecting humans that are transmitted through contaminated soil ("helminth" means worm): *Ascaris lumbricoides* (sometimes called just "*Ascaris*"), whipworm (*Trichuristrichiura*), and hookworm (*Ancilostoma duodenale* and *Necator americanus*) are the major STH. | Trachoma is a potentially blinding infectious eye disease caused by a micro-organism called *Chlamydia trachomatis* spread by a direct or indirect contact with infected individuals. |

| Diseases | Lymphatic Filariasis (LF) | Onchocerciasis (ONCHO) | Schistosomiasis (SCH) | Soil-transmitted Helminths (STH) | Trachoma |
|---|---|---|---|---|---|
| | | | to the liver where they grow and sexually mature. Mature male and female worms pair and migrate either to the intestines or the bladder where egg production occurs. | | |
| **Signs and Symptoms** | The signs and symptoms of LF are mainly elephantiasis (leg, arm, vulva or breast) and hydrocoele (swollen scrotum). Others may include: fever, headache, pain and chyluria (milky urine). | The adult worms are found in nodules located in the subcutaneous tissue under the skin. Migration of the microfilaria through the skin and eyes is responsible for the major symptoms experienced by infected persons. The conditions include skin lesions (scaly or leopard skin), skin rashes accompanied by intense itching and resultant scratching (causing a condition commonly known as craw-craw), hanging groin, and ultimately, blindness. | Many individuals do not experience symptoms. If present, it usually takes four to six weeks for symptoms to appear. The first symptom of the disease may be a general ill feeling. Within twelve hours of infection, an individual may complain of a tingling sensation or light rash, commonly referred to as "swimmer's itch", due to irritation at the point of entrance. The rash that may develop can mimic scabies and other types of rashes. The disease can also start with blood in urine or stool, anaemia, or problem with growth and development of children, and eventually become life threatening due to health effects such as bladder cancer, kidney and liver problems.<br>Other symptoms can occur two to ten weeks later and can include fever, aching, cough, diarrhoea, or gland enlargement. | People with light soil-transmitted helminth infections usually have no symptoms. Heavy infections can cause a range of health problems, including abdominal pain, diarrhoea, blood and protein loss, rectal prolapse, and physical and cognitive growth retardation. Soil-transmitted helminth infections are preventable and treatable with medication prescribed by health care provider. | Discharge from the eyes, reddening of the eyes, and deformity of the eyelids, inability to see properly, and eventually blindness. |
| **Intervention Measures** | The Local Government Area (LGA) is LF endemic when a selected community of the LGA has at least one (1) positive person in 100 recruits with immunochromatographic test (ICT). The intervention | Endemicity is determined using nodular palpation or skin snipping methods. Currently prevalence of 10% and above of an area identified using REMO principles calls for | LGA is schistosomiasis endemic when the average prevalence of the five selected communities in the LGA is at least one percent (1%) positive using standard diagnostic procedures (Heamastix/microscopy). The | LGA is STH endemic when the average prevalence of the five selected community in the LGA has at least twenty percent (20%) positive using standard diagnostic procedures (Kato- | The strategy for the elimination of blinding trachoma endorsed by the World Health Organization is the SAFE strategy. The acronym SAFE stands for: S- Surgery to treat the |

| Diseases | Lymphatic Filariasis (LF) | Onchocerciasis (ONCHO) | Schistosomiasis (SCH) | Soil-transmitted Helminths (STH) | Trachoma |
|---|---|---|---|---|---|
| | measures for LF after mapping are: Baseline survey, mass distribution of medicines (MDM), surveillance, long lasting insecticide treated nets (LLIN) distribution, health education, hydrocoelectomy and personal hygiene practice for elephantiasis victims. Stakeholder/Partner implementing or supporting LF Programme in the State should have plans for adequate resources for a minimum of six (6) years before starting LF MDM in endemic LGA(s). | the mass distribution of Ivermectin, a microfilaricide that kills 99% of microfilariae. Annual doses of the medicine for a period of up to 20 years reduce drastically the symptoms of the disease and interrupt transmission. Through the Community Directed Treatment with Ivermectin (CDTI) strategy, Community Directed Distributors (CDDs) selected by endemic communities treat eligible persons. Vector control is no longer considered cost-effective. | intervention measures for schistosomiasis after mapping are mass distribution of praziquantel among school age children (enrolled and non enroled) once every two years, praziquantel should be available in dispensaries and cliniucs for treatment of suspected cases in LGA with prevalence ≥1% and <10% by parasitological method (intestinal and urinary schistosomiasis).<br><br>Treat all School Age Children (enrolled and non-enrolled) once every year in LGAs ≥10% and <50% by parasitological method (intestinal and urinary schistosomiasis).<br><br>Treat the entire community once every year in in LGA with prevalence ≥50% by parasitological method (intestinal and urinary schistosomiasis).<br><br>Stakeholders/partners implementing or supporting Schistosomiasis Programme in the State should have plans for adequate resources before starting Schistosomiasis MAM in endemic LGA(s). | katz/microscopy). The intervention measures for STH after mapping, are annual mass distribution of medicines (MDM) in LGA with prevalence ≥20% and <50%, bi-annual mass distribution of medicines (MDM) in LGA with prevalence ≥50%, surveillance, health education, water, sanitation and personal hygiene practice in endemic communities. | blinding stage of the disease (trachomatoustrichiasis)<br>A- Antibiotics to treat active infection<br>F- Facial cleanliness to reduce disease transmission<br>E- Environmental improvement particularly improving access to clean water and sanitation<br>**Decision Making for the Antibiotic Treatment of Trachoma:**<br>Baseline $TF_{1\_9}$≥50% = ≥ 7 rounds of MDA; Baseline $TF_{1\_9}$≥30 - 49.9% = ≥ 5 rounds of MDA; Baseline $TF_{1\_9}$≥10 - 29.9% = ≥ 3 rounds of MDA; Baseline $TF_{1\_9}$≥5 - 9.9% = Targeted treatment, one round of MDA; Baseline $TF_{1\_9}$ <5 = no MDA |
| **Target Populations for Mass Administration** | The target population for LF MDM is 80% of the at-risk population of endemic LGA. The annual projected National | The target population for mass distribution of ivermectin is 80% of the total population at risk in | The target population for Schistosomiasis MAM is School age children and adult in high risk communities of endemic LGA. 28% | The target population for STH MAM is at least 75% of the at-risk population [Pre School Age Children (PSAC), | The target is to treat the entire population. |

| Diseases | Lymphatic Filariasis (LF) | Onchocerciasis (ONCHO) | Schistosomiasis (SCH) | Soil-transmitted Helminths (STH) | Trachoma |
|---|---|---|---|---|---|
| **of Medicines (MAM)** | Population Commission (NPC) figure of the LGA is the at-risk population for LF. | an endemic community. The population at risk is the whole population in identified groups of villages or communities and not necessarily an entire LGA. | of the annual projected National Population Commission (NPC) figure of the LGA is the at-risk population for school age children in low and medium risk LGAs while the entire population is the at-risk population in high risk communities. | School Age Children (SAC) and Women of Child Bearing Age (WCBA)] in endemic LGAs. 28% of the annual projected National Population Commission (NPC) figure of the LGA is the at-risk population for STH. | |
| **Eligibility** | People who can take the LF medicines (Ivermectin and Albendazole tablets) are those that are ≥ 90cm in height or ≥ 5 years of age living in endemic LGA. Those people that cannot take the medicines include those that are <90cm in height or <5years of age, pregnant women, lactating mothers within the first 7 days and seriously sick people. | All persons ≥ 5years in an endemic community is required to use an annual dose of ivermectin with the exception of children below 5 years or less than 90cm in height, pregnant women, lactating mothers within the first 7 days of delivery and severely ill persons. | People who can take the Schistosomiasis medicines (Praziquantel tablets) are those that are between 5 and 15 years of age and not less than 110cm, include adult in high risk LGAs. Those people that cannot take the medicines include pregnant women and children who are ill on the treatment day. This is not because of any danger of side effects, but to prevent the potential misperception that the deworming drug(s) have caused the illness. These children can be given the drug(s) later when they are well again. | People who can take the STH medicines (Albendazole and Mebendazole tablets) are Pre School Age Children (PSAC) 1-4 years, School Age Children (SAC) 5-15 years and Women of Child Bearing Age (WCBA) 16-44 years. Those people that cannot take the medicines include children who are ill on the treatment day. This is not because of any danger of side effects, but to prevent the potential misperception that the deworming drug(s) have caused the illness. These children can be given the drug(s) later when they are well again. | Two types of antibiotics are given for trachoma control: Azithromycin and tetracycline eye ointment (TEO). Zithromax is safe for all people, although in Nigeria children less than 6 months of age and self-reporting preganant women are excluded from Zithromax distribution and are offered TEO. Below are the eligibility criteria for distribution of Zithromax.<br>Children less than 6 months and self-reporting pregnant women: Give 2 tubes of Tetracycline ointment (TEO).<br>Children 6 months – 5 years: Give Pediatric Oral Suspension (POS)<br>Children 5 years and above: Give Zithromax tablet. |

| Diseases | Lymphatic Filariasis (LF) | Onchocerciasis (ONCHO) | Schistosomiasis (SCH) | Soil-transmitted Helminths (STH) | Trachoma |
|---|---|---|---|---|---|
| **Managing Adverse Reactions** | Adverse reactions that may occur when a person takes Ivermectin and Albendazole medicines may include: headache, fever, body aches, dizziness, decreased appetite, malaise, nausea, itching (urticaria), vomiting, wheezing and bronchial asthma. However, in areas where *Loa loa* is co-endemic with LF, there may be severe adverse reactions such as 'encephalopathy'. Any person who experiences any of the above adverse reactions after taking the medicines should go to the **nearest health facility**. In areas where *Loa loa* is co-endemic, treatment with Albendazole medicine and adequate LLIN distribution should be used. Adverse reactions in LF MDM are common. | Adverse reaction from treatment with Ivermectin is rare and reactions can occur in form of headaches, dizziness, body aches, fever, nausea and itching. When these reactions are experienced, the affected persons should be taken to the nearest health facility for the management of the reactions. | Adverse reactions that may occur when a person takes Praziquantel tablet may include: mild headache, fever, body aches, dizziness, decreased appetite, malaise, nausea and vomiting. However, these reactions are rarely experienced provided drugs are taken after meal. Any person who experiences any of the above adverse reactions after taking the medicines should go to the **nearest health facility**. | Adverse reactions that may occur when a person takes Albendazole or Mebendazole tablet may include: mild headache, fever, body aches, dizziness, decreased appetite, malaise, nausea and vomiting. However, these reactions are rarely experienced. Any person who experiences any of the above adverse reactions after taking the medicines should go to the **nearest health facility**. | Zithromax is well tolerated with a low incidence of adverse events. Communities undergoing treatment should be informed in advance that some people will have reactions such as Nausea, vomiting, diarrhea or constipation. Programme beneficiaries should be encouraged to eat their breakfast before they come to take the medicine. They should also drink plenty of liquids such as water, tea or milk before and after treatment. Report any adverse reaction to the appropriate authority immediately it is noticed or the nearest Health Center. Any severe adverse events must be reported immediately to Pfizer through the Ministry of Health. |
| **Elimination Thresholds** | Impact assessment after 5 rounds of effective MDM at sentinel site(s) should be less than 1% mf (microfilaria) or less than 1% CFA (circulating filarial antigen). Thereafter, Evaluation unit (LGA or LGAs) must pass Transmission Assessment Survey (TAS) as in prescribed algorithm. LLIN distributions and other | The disease is said to be eliminated when the prevalence of microfilaria infection of <5% in all surveyed villages or 1% in 90% of surveyed villages is declining or remain the same. This infectivity rate is determined during the phase 2 of a three-phase evaluation. After the | Impact assessment is conducted after 3-5 years of uninterrupted MAM with Praziquantel. Parameters assessed include: 1. Parasitological Indicators (prevalence of infection, intensity of infection measured indirectly by counting worm eggs excreted in faeces expressed as eggs per gram of faeces); all measured against baseline or pre-intervention data. | Impact assessment is conducted after 3-5 years of uninterrupted MAM with Mebendazole/Albendazole. Parameters assessed include: 1. Parasitological Indicators (prevalence of infection, intensity of infection measured indirectly by counting worm eggs excreted in faeces expressed | If TF in children age 1-9 years in a district is 10% and above, it is clear interventions of SAFE are required. If TF in children age 1-9 years is between 5-9.9%, one year of MDA plus F&E may be conducted followed by impact assessment. If the findings are <5% TF, it is likely that |

| Diseases | Lymphatic Filariasis (LF) | Onchocerciasis (ONCHO) | Schistosomiasis (SCH) | Soil-transmitted Helminths (STH) | Trachoma |
|---|---|---|---|---|---|
| | surveillance activities must continue in areas that have passed TAS (i.e. TAS1, TAS2 and TAS3) until the country is verified and certified LF free. | evaluations, the areas to stop treatment are determined based on several factors. | 2. Morbidity indicators (lesion of urinary tract or liver caused by schistosomiasis which will require use of such instruments as ultrasound machine, digital haemoglobinometer, etc.<br>3. Water Sanitation and Hygiene (WASH) Indicators (Knowledge, Attitude and Practices -KAP), presence, adequacy and conditions of safe water supply, environmental control and sanitation; improved school attendance, retention and performance).<br>The impact assessment is conducted in at least five sentinel sites selected per senatorial district. 50% of the sentinel sites should be maintained and used to monitor the impact of intervention over the years while 50% of schools are changed every year. Data should be collected from children of the same age and those in their third year of school are preferred. Reduction in disease transmission is evaluated using first year children who are normally have not been exposed to treatment.<br>SCH is regarded as ***eliminated as a public health problem*** if:<br>Prevalence of schistosomiasis heavy infection in school-aged children in endemic areas is less than 1% (Heavy infection: Egg intensity ≥100 eggs per gram for S. mansoni or ≥ 50 eggs per 10 ml of urine for S. haematobium) | as eggs per gram of faeces); all measured against baseline or pre-intervention data<br>2. Morbidity indicators (Malnutrition, anaemia and its consequences) .<br>3. Water Sanitation and Hygiene (WASH) Indicators (Knowledge, Attitude and Practices -KAP), presence, adequacy and conditions of safe water supply, environmental control and sanitation; improved school attendance, retention and performance).<br>The impact assessment is conducted in at least five sentinel sites selected per senatorial district. 50% of the sentinel sites should be maintained and used to monitor the impact of intervention over the years while 50% of schools are changed every year. Data should be collected from children of the same age and those in their third year of school are preferred.<br>Reduction in disease transmission is evaluated using first year children who are normally have not been exposed to treatment.<br>If after assessment prevalence is <2% no further intervention is needed; if 2- | trachoma does not constitute a public health problem and no specific interventions are needed. After impact assessments if results are $TF_{1\_9} \geq 30\%$ = ≥ 5 rounds of MDA; if $TF_{1\_9} \geq 10$ - 29.9% = ≥ 3 rounds of MDA; if $TF_{1\_9} \geq 5$ - 9.9% = ≥1 round of MDA. WHO has declared the elimination of blinding trachoma to be equivalent to a prevalence of trachomatous inflammation-follicular (TF) in children age 1-9 years to be less than 5% after two years of surveillance. |

| Diseases | Lymphatic Filariasis (LF) | Onchocerciasis (ONCHO) | Schistosomiasis (SCH) | Soil-transmitted Helminths (STH) | Trachoma |
|---|---|---|---|---|---|
| | | | or<br>Reduction of pattern B fibrosis is less than 10% and absence of pattern C fibrosis on ultrasonography for *S. mansoni* and prevalence of bladder lesions less than 10% in *S. haematobium*.<br>***Transmission of SCH is regarded as interrupted*** if: Zero prevalence of Schistosomiasis among school-aged children for three consecutive years and<br>Absence of symptomatic and laboratory confirmed schistosomiasis cases in the health facilities in endemic areas. | 10% then PC once every 2 years; if 10 - 20% PC is annual; if 20 - 50% maintain existing frequency of PC with an additional round targeted at SAC; if >50% then increase frequency of PC to 3 times annually. | |

## *Innovative Disease Management (IDM) NTDs*

| Diseases | Buruli ulcer | Guinea Worm Disease | Human African Trypanosomiasis | Leishmaniasis | Leprosy | Rabies | Yaws |
|---|---|---|---|---|---|---|---|
| Photos | *Non ulcerative forms*<br>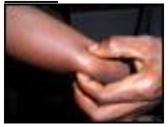<br>*Small ulcers*<br>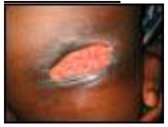<br>*Large ulcers* | 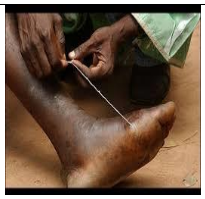<br>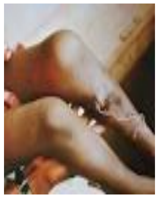 | 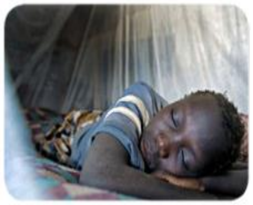 | 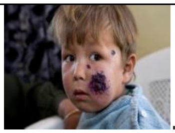.<br>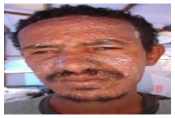<br>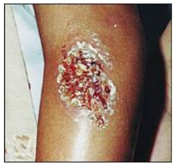 | 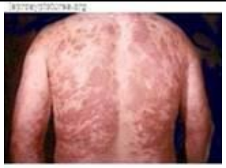<br>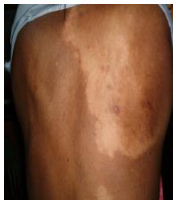 | 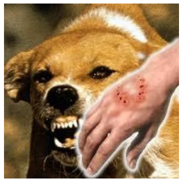<br>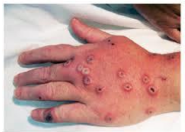 | 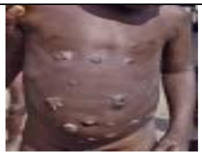<br>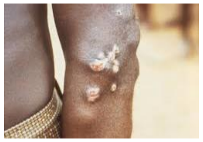 |

| Diseases | Buruli ulcer | Guinea Worm Disease | Human African Trypanosomiasis | Leishmaniasis | Leprosy | Rabies | Yaws |
|---|---|---|---|---|---|---|---|
| | 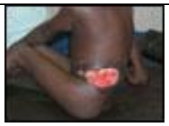 | | | | | | 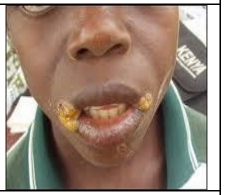 |
| **Case definition** | Buruli ulcer is a chronic debilitating skin and soft tissue infection that can lead to permanent disfigurement and disability. | Any person with a wound and Guinea worm emerging from the wound. | Generally known as sleeping sickness, Human African Trypanosomiasis is a parasitic infection that occurs in sub-Saharan Africa and is transmitted by tsetse flies. It attacks the central nervous system, causing severe neurological disorders or even death. | A parasitic disease that is spread by the bite of sand flies infected with the protozoa Leishmania. There are several forms of leishmaniasis, the most common being cutaneous and visceral leishmaniasis (known as kala-azar). The cutaneous form of the disease causes skin sores and is usually named for a geographic place (for example, Jericho boil, Baghdad button, Delhi sore). Visceral leishmaniasis affects the internal organs of the body and can be fatal. | Leprosy is a slowly progr essing bacterial infection that affects the skin, peripheral nerves in the ha nds and feet, and mucous membranesof the n ose, throat, and eyes. De struction of the nerve en dings causes the the affected areas to lose sensation. Occasionally, because ofthe loss of feeling, the fingers and toes become mutilated and f all off, causing the deformities that are typically associated with the disease. | An acute, infectious, usually fatal viral disease of the central n ervous system that is transmitted by the saliva and bite of infected a nimals and occurs in many mam mals, including dogs, cats, raccoons, and b ats causing madness and convulsions. | Yaws: contagious disease, caused by a bacterium that enters skin abrasions and gives rise to small crusted lesions which may develop into deep ulcers. |
| **Causative Organism** | *Mycobacterium ulcerans* | Guinea worm(*Dracunculu smedinensis*.) | *Trypanosoma gambiense* | Leishmania sp. | *Mycobacterium leprae* | Rabies virus | *Treponema palladium pertenue* |
| **Vector** | _ | Cyclops | Tsetse fly | Sandfly | _ | Scratches and Bites from infected animals like rabid dogs, bats | _ |
| **Diagnosis** | Histopathological staining of Skin lesion and Polymerase Chain reaction | Emergence of the adult worm from boils or skin blisters | Microscopic detection of parasite in body fluids and polymerase chain reaction | Demonstration of parasites in tissues of relevance by light microscopy | one or more hypopigmented, anaesthetic skin patches, typical of leprosy; one or more | Detection of negri bodies (rabies virus) from suspected animals, Polymerase chain reaction on body fluids of | Dark field microscopy of skin lesion and Polymerase Chain reaction |

| Diseases | Buruli ulcer | Guinea Worm Disease | Human African Trypanosomiasis | Leishmaniasis | Leprosy | Rabies | Yaws |
|---|---|---|---|---|---|---|---|
| | | | | | thickened peripheral nerves; or a positive skin smear. | affected animals or humans | |
| **Strategies for Elimination** | 1. Advocacy, Health Education, social mobilization.<br>2. Surveillance, case detection and reporting.<br>3.Chemotherapy using recommended drugs.<br>4. Case containment and Management.<br>5. Safe water supply. | 1. Advocacy, Health Education, social mobilization.<br>2. Surveillance, case detection and reporting.<br>3. Case containment and Management<br>4. Vector control.<br>5. Safe water supply. | 1. Advocacy, Health Education, social mobilization.<br>2. Surveillance, case detection and reporting.<br>3. Chemotherapy using recommended drugs.<br>4. Case containment and Management. .<br>5. Vector control.<br>6. Safe water supply. | 1. Advocacy, Health Education, social mobilization.<br>2. Surveillance, case detection and reporting.<br>3. Chemotherapy using recommended drugs.<br>4. Case containment and Management. .<br>5. Vector control.<br>6. Safe water supply. | 1. Advocacy, Health Education, social mobilization.<br>2. Surveillance, case detection and reporting.<br>3. Chemotherapy using recommended drugs.<br>4. Case containment and Management. | 1. Advocacy, Health Education, social mobilization.<br>2. Surveillance, case detection and reporting.<br>3. Chemotherapy using recommended drugs.<br>4. Case containment and Management.<br>5. Vector control. | 1. Advocacy, Health Education, social mobilization.<br>2. Surveillance, case detection and reporting.<br>3. Chemotherapy using recommended drugs.<br>4. Case containment and Management.<br>5. Safe water supply. |

## Protocol for multiple medicine administration in LGAs/Communities/Schools

| | Co-endemicity scenario | Interventions and frequency of treatment |
|---|---|---|
| **1** | **Where LF is Endemic…….** | |
| **1a** | **……and Oncho is present with Trachoma** | |
| i | If an LGA is endemic for LF + Oncho + Trachoma + SCH + High STH (2ce a year) | Treat with IVM + ALB + AZT (Communities)<br>After 2 weeks, treat with PRQ only (Schools)<br>After 6 months, treat with ALB or MEB in schools |
| ii | If an LGA is endemic for LF + Oncho + Trachoma + SCH + Moderate STH (1ce a year) | Treat with IVM + ALB + AZT (Communities)<br>After 2 weeks, treat with PRQ only (Schools)<br>(ALB would have taken care of 1ce a year treatment for STH) |
| iii | If an LGA is endemic for LF + Oncho + Trachoma + SCH + Low STH (no treatment) | Treat with IVM + ALB + AZT (Communities)<br>After 2 weeks, treat with PRQ only (Schools)<br>Low STH is considered as no STH |
| iv | If an LGA is endemic for LF + Oncho + Trachoma + High STH (2ce a year) and no SCH | Treat with IVM + ALB + AZT (Communities)<br>After 6 months, treat with ALB or MEB in schools (no need to treat with PRQ because no SCH) |
| v | If an LGA is endemic for LF + Oncho + Trachoma + Moderate STH (1ce a year) and no SCH | Treat with IVM + ALB + AZT (Communities)<br>(ALB would have taken care of 1ce a year treatment for STH) |
| vi | If an LGA is endemic for LF + Oncho + Trachoma + low STH (no treatment) and no SCH | Treat with IVM + ALB + AZT (Communities)<br>Low STH is considered as no STH |
| **1b** | **……and oncho is present without Trachoma** | |
| i | If an LGA is endemic for LF + Oncho + SCH + high STH (2ce a year) | Treat with IVM + ALB (Communities)<br>After 2 weeks, treat with PRQ only (Schools)<br>After 6 months, treat with ALB or MEB (Schools) |
| ii | If an LGA is endemic for LF + Oncho + SCH + moderate STH (1ce a year) | Treat with IVM + ALB (Communities)<br>After 2 weeks, treat with PRQ only (Schools) |
| iii | If an LGA is endemic for LF + Oncho + SCH + low STH (no treatment) | Treat with IVM + ALB (Communities)<br>After 2 weeks, treat with PRQ only (Schools) |
| iv | If an LGA is endemic for LF + Oncho + high STH (2ce a year) and no SCH | Treat with IVM + ALB (Communities)<br>After 6 months, treat with ALB or MEB (Schools) |
| v | If an LGA is endemic for LF + Oncho + moderate STH (1ce a year) and no SCH | Treat with IVM + ALB (Communities)<br>(ALB would have taken care of 1ce a year treatment for STH) |

| | | |
|---|---|---|
| vi | If an LGA is endemic for LF + Oncho + low STH (no treatment) and no SCH | Treat with IVM + ALB (Communities) |
| **1c** | **……but Oncho and Trachoma is not present** | |
| i | If an LGA is endemic for LF + SCH + high STH (2ce a year) and no Oncho | Treat with IVM + ALB (Communities)<br>After 2 weeks, treat with PRQ only (Schools)<br>After 6 months, treat with ALB or MEB (Schools) |
| ii | If an LGA is endemic for LF + SCH + moderate STH (1ce a year) and no Oncho | Treat with IVM + ALB (Communities)<br>After 2 weeks, treat with PRQ only (Schools) |
| iii | If an LGA is endemic for LF + SCH + low STH (no treatment) and no Oncho | Treat with IVM + ALB (Communities)<br>After 2 weeks, treat with PRQ only (Schools) |
| iv | If an LGA is endemic for LF + high STH (2ce a year) but no Oncho and SCH | Treat with IVM + ALB (Communities)<br>After 6 months, treat with ALB or MEB (Schools) |
| v | If an LGA is endemic for LF + moderate STH (1ce a year) but no Oncho and SCH | Treat with IVM + ALB (Communities) |
| vi | If an LGA is endemic for LF + low STH (no treatment) but no Oncho and SCH | Treat with IVM + ALB (Communities) |

| | Co-endemicity scenario | Interventions and frequency of treatment |
|---|---|---|
| **2** | **Where LF is <u>not Endemic</u>…….** | |
| **2a** | **……but Oncho is present with Trachoma** | |
| i | If an LGA is endemic for Oncho + Trachoma + SCH + High STH (2ce a year) | Treat with IVM + AZT (Communities)<br>After 2 weeks, treat with PRQ + MEB or ALB (Schools)<br>After 6 months, treat with ALB or MEB (Schools) |
| ii | If an LGA is endemic for Oncho + Trachoma + SCH + Moderate STH (1ce a year) | Treat with IVM + AZT (Communities)<br>After 2 weeks, treat with PRQ + MEB or ALB (Schools)<br>(ALB or MEB would have taken care of 1ce a year treatment for STH) |
| iii | If an LGA is endemic for Oncho + Trachoma + SCH + Low STH (no treatment) | Treat with IVM + AZT (Communities)<br>After 2 weeks, treat with PRQ only (Schools)<br>Low STH is considered as no STH |
| iv | If an LGA is endemic for Oncho + Trachoma + High STH (2ce a year) and no SCH | Treat with IVM + AZT (Communities)<br>Treat with MEB or ALB (Schools)<br>After 6 months, treat with ALB or MEB in schools (no need to treat with PRQ because no SCH) |

| | | |
|---|---|---|
| v | If an LGA is endemic for Oncho + Trachoma + Moderate STH (1ce a year) and no SCH | Treat with IVM + AZT (Communities)<br>Treat with MEB or ALB (Schools)<br>(ALB would have taken care of 1ce a year treatment for STH) |
| vi | If an LGA is endemic for Oncho + Trachoma + low STH (no treatment) and no SCH | Treat with IVM + AZT (Communities)<br>Low STH is considered as no STH |
| **2b** | **……and Oncho is present without Trachoma** | |
| i | If an LGA is endemic for Oncho + SCH + high STH (2ce a year) | Treat with IVM (Communities)<br>After 2 weeks, treat with PRQ + ALB or MEB (Schools)<br>After 6 months, treat with ALB or MEB (Schools) |
| ii | If an LGA is endemic for Oncho + SCH + moderate STH (1ce a year) | Treat with IVM (Communities)<br>After 2 weeks, treat with PRQ only (Schools) |
| iii | If an LGA is endemic for Oncho + SCH + low STH (no treatment) | Treat with IVM (Communities)<br>After 2 weeks, treat with PRQ only (Schools) |
| iv | If an LGA is endemic for Oncho + high STH (2ce a year) and no SCH | Treat with IVM (Communities)<br>Treat with ALB or MEB (Schools)<br>After 6 months, treat with ALB or MEB (Schools) |
| v | If an LGA is endemic for Oncho + moderate STH (1ce a year) and no SCH | Treat with IVM (Communities)<br>Treat with ALB or MEB (Schools)<br>(ALB would have taken care of 1ce a year treatment for STH) |
| vi | If an LGA is endemic for Oncho + low STH (no treatment) and no SCH | Treat with IVM only (Communities) |
| **2c** | **……but Oncho and Trachoma is not present (this applies to SCH and STH only endemic areas)** | |
| i | If an LGA is endemic for SCH + high STH (2ce a year) and no Oncho | Treat with PRQ + ALB or MEB (Schools)<br>After 6 months, treat with ALB or MEB (Schools) |
| ii | If an LGA is endemic for SCH + moderate STH (1ce a year) and no Oncho | Treat with PRQ + ALB or MEB (Schools) |
| iii | If an LGA is endemic for SCH + low STH (no treatment) and no Oncho i.e SCH only LGA | Treat with PRQ only (Schools) |
| iv | If an LGA is endemic for high STH (2ce a year) but no Oncho and SCH i.e High STH only LGA | Treat with ALB or MEB (Schools)<br>After 6 months, treat with ALB or MEB (Schools) |
| v | If an LGA is endemic for moderate STH (1ce a year) but no Oncho and SCH i.e Moderate STH only LGA | Treat with ALB or MEB (Schools) |
| vi | If an LGA is endemic for low STH (no treatment at all) but no Oncho and SCH i.e NTD free LGA | No Mass Administration of Medicines |

Points to note:

1. If an LGA has a high prevalence of $\geq$50% for SCH, it should be remembered that adults in communities are to be treated also. In these cases, community implementers are also to treat with PRQ only 2 weeks after any previous MDA have occurred.
2. Proper mobilization must therefore be carried out in both schools and communities so that SAC enrolled and treated in schools will not use PRQ again in the communities
3. LF co-endemic area with Loa loa- use albendazole medicine and vector control (LLIN distribution and Indoor Residual Spray)
4. Tablet pole is a long piece of wood marked with height intervals corresponding to the number of praziquantel or mectizan tablets needed to treat school age children and adults for schistosomiasis, onchocerciasis and lymphatic filariasis.

**For further information contact:**

Head, Neglected Tropical Diseases,
Department of Public Health,
Federal Ministry of Health, CBA, Abuja
Federal Capital Territory, Nigeria.
